# Frontier-orbital modulation of rhodium single-atom catalysts for enhanced hydrogen evolution

**DOI:** 10.1038/s41467-026-73161-6

**Published:** 2026-05-18

**Authors:** Rouna Jia, Zongyan Liu, Yang Wang, Jingyang Zhao, Zhong Huang, Wen Yue, Haozhi Wang, Kaiping Yu, Mingxin Huang, Yida Deng

**Affiliations:** 1 State Key Laboratory of Tropic Ocean Engineering Materials and Materials Evaluation, School of Materials Science and Engineering, Hainan University, Haikou, China; 2 Key Laboratory of Pico Electron Microscopy of Hainan Province, Hainan University, Haikou, China; 3 School of Information and Communication Engineering, Hainan University, Haikou, China; 4 State Key Laboratory of Deep Earth Exploration and Imaging, School of Engineering and Technology, China University of Geosciences Beijing, Beijing, China; 5 Department of Mechanical Engineering, The University of Hong Kong, Hong Kong, Hong Kong China

**Keywords:** Electrocatalysis, Electrocatalysis, Hydrogen energy, Electrocatalysis

## Abstract

Single-atom catalysts (SACs) are promising for hydrogen evolution due to their maximal atomic utilization and discrete energy levels. Modulating metal-support interactions is key to tailoring their activity and stability, yet achieving precise control and mechanistic insight remains challenging and controversial. Here, we construct a rhodium single-atom catalyst model system, with Rh atoms anchored on a series of MoS_x_Se_2-x_ supports (Rh_SA_-MoS_x_Se_2-x_, 0 ≤ x ≤ 2), enabling gradient modulation of metal-support frontier orbital interactions through systematic tuning the anion composition. The elevated lowest unoccupied molecular orbital (LUMO) of MoS_x_Se_2-x_ support narrows the energy gap with the highest occupied molecular orbital (HOMO) of Rh atoms, strengthening metal-support orbital hybridization to enhance stability and further amending the LUMO of Rh atoms to optimize both the hydroxide and hydrogen adsorption for high activity. The apex Rh_SA_-MoSSe catalyst, with optimal HOMO-LUMO hybridization, achieves favorable hydrogen evolution reaction activity and stability simultaneously. This work offers fundamental insights into the metal-support frontier orbital interaction in SACs and establishes a rational design framework for high activity and stability electrocatalysis.

## Abstract (web summary)

Single-atom catalysts offer high efficiency for hydrogen evolution, but control of metal–support interactions remains challenging. Here, the authors report a rhodium single-atom catalyst platform enabling continuous tuning of metal–support frontier orbital interactions via anion-engineered supports.

## Introduction

Renewable-electricity-driven water electrolysis offers a sustainable hydrogen production pathway^1,2^, yet alkaline hydrogen evolution reaction (HER) suffers from sluggish kinetics due to coupled water dissociation and proton-transfer barriers^3–6^. While volcano relationships link HER activity to hydroxide (OH)^7–9^ and hydrogen (H)^10,11^ adsorption energies, simultaneously optimizing both intermediates remains fundamentally challenging. In the past decades, Rh-based catalysts have shown great potential to substitute Pt/C for alkaline HER by virtue of the much lower energy barrier for water dissociation and approximate H adsorption energy with Pt^12–14^, but their scarcity and high cost greatly hindered large-scale applications. Single atom catalysts (SACs) hold great promise due to their maximal atomic utilization and tunable coordination environment^15–18^, but their activity and long-term stability still require significant enhancement.

Metal-support interactions (MSIs) are crucial for stabilizing single atoms on the support and modulating the *d*-band structure of single-atom sites, while also influencing the local electronic structure of the support in SACs^16–19^. Precise control of MSIs is essential for enhancing catalyst stability and optimizing the binding energies of reactants and intermediates, which govern the catalytic performance of SACs. To date, MSI's research has predominantly focused on the active single-atom metal, with the critical role of support materials often overlooked. Conventional strategies, such as varying the type or increasing the loading of single atoms, increase catalyst costs and face limitations in enhancing activity. Given that the coordination number, chemical bonding, and spatial environment of single-atom sites are dictated by the properties of the support, tailoring the support’s composition and phase structure offers an effective approach to modulate the geometry and electronic structure of SACs^20–22^. This has been demonstrated in heteroatom (e.g., N, P, O, and S) carbon-based SACs, where activity and selectivity are significantly altered by adjusting the extent of heteroatom coordination^23–26^. Compared to relatively inert carbon-based supports, transition metal compounds, with their tunable band structures and covalent bonding capabilities, serve as ideal platforms for stabilizing single atoms^27–31^. However, achieving precise MSIs control through atomic-level compositional gradients remains challenging, impeding a clear understanding of structure-performance relationships. Notably, the role of metal-support frontier orbital hybridization in anion-engineered supports remains largely unexplored, representing a critical gap in the orbital-level design of SACs.

Herein, we establish a Rh SACs platform (Rh_SA_-MoS_x_Se_2-x_, 0 ≤ x ≤ 2) enabling continuous metal-support frontier orbital interactions via anion-engineered supports. The detailed experimental characterization and theoretical simulations suggest that the LUMO of MoS_x_Se_2-x_ substrates exhibits a volcano-type relationship with the S: Se ratio, with MoSSe located at the volcano apex. Moreover, the elevated LUMO of MoS_x_Se_2-x_ promotes Rh-support orbital hybridization for high stability and modulates the LUMO of anchored Rh atoms to optimize Rh-adsorbate interactions for high activity. The Rh_SA_-MoSSe with optimal HOMO-LUMO hybridization simultaneously balances intermediate adsorption-desorption kinetics and reinforces Rh-support orbital hybridizations, leading to long-term stability under industrially relevant fluctuating power conditions (> 300 h). This work resolves the nexus between orbital hybridization and electrocatalysis, offering a universal design paradigm beyond activity-stability compromises.

## Results

### Theoretical design of Rh single-atom catalysts

**Fig. 1:**
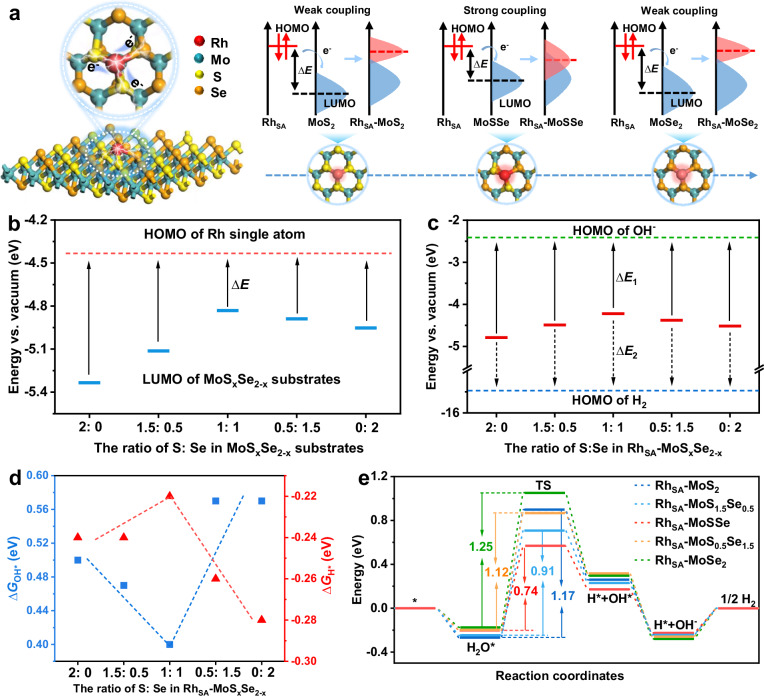
Theoretical design of Rh single-atom catalysts. **a** Schematic illustration of modulating MSIs in Rh_SA_-MoS_x_Se_2-x_ by varying the S: Se ratio of MoS_x_Se_2-x_ supports. **b** Calculated LUMO positions of MoS_x_Se_2-x_ substrates with different S: Se ratios and the HOMO of unsupported Rh atom relative to vacuum. Black arrows highlight the energy discrepancies (Δ*E*) between the MoS_x_Se_2-x_ LUMOs and the Rh atom HOMO. **c** Calculated LUMO positions of Rh single atoms anchored on MoS_x_Se_2-x_ substrates (red solid line) and the HOMO of OH^-^ (green dashed line) and H_2_ (blue dashed line) relative to vacuum. Black arrows highlight the energy discrepancies (Δ*E*_1_) between the Rh_SA_ LUMOs and the OH^-^ HOMO. Black dashed arrows highlight the energy discrepancies (Δ*E*_2_) between the Rh_SA_ LUMOs and the H_2_ HOMO. **d** Correlation between the S: Se ratio and Δ*G*_OH*_, and Δ*G*_H*_ in Rh_SA_-MoS_x_Se_2-x_. (e) Gibbs free energy diagram for the HER process at Rh sites of Rh_SA_-MoS_x_Se_2-x_. Source data are provided as a Source Data file.

To gain a comprehensive understanding of metal-support frontier orbital interactions, we construct a model system of Rh single-atom catalysts (SACs), in which the HOMO of the Rh single atoms (Rh_SA_) can be effectively modulated by tuning the anion composition in the support (Fig. 1a). First principal density functional theory (DFT) calculations were conducted to investigate the MSIs effects based on the MoS_x_Se_2-x_ and Rh_SA_-MoS_x_Se_2-x_ (0 ≤ x ≤ 2) structure models (Supplementary Data 1, Supplementary Figs. 1 and 2). The density of states (DOS) suggests that the incorporation of Rh single-atoms significantly increases the DOS of MoS_x_Se_2-x_ near the Fermi level (Supplementary Fig. 3). Moreover, the band gap of Rh_SA_-MoSSe (0.68 eV) is narrower than those of Rh_SA_-MoS_2_ (0.88 eV), Rh_SA_-MoS_1.5_Se_0.5_ (0.73 eV), Rh_SA_-MoS_0.5_Se_1.5_ (0.70 eV), and Rh_SA_-MoSe_2_ (0.73 eV), and MoS_x_Se_2-x_ substrates, thereby promoting accelerated electron transfer and optimizing the adsorption and desorption of reaction intermediates. The calculated HOMO position of freestanding Rh atom is predicted to be around -4.50 eV, whereas the LUMO of MoS_x_Se_2-x_ substrates exhibit a volcano-type trend with the S: Se ratio, with MoSSe appearing near the volcano apex (Fig. 1b, Supplementary Fig. 4, and Supplementary Note 1). According to the FMO theory^32–34^, the elevated LUMO of MoSSe support narrows the energy gap with the highest occupied molecular orbital (HOMO) of Rh atoms, which promotes orbital hybridizations between Rh atoms and support to lead to a high stability.

When electrons are transferred from Rh to MoS_x_Se_2-x_ substrates, the anchored Rh atoms exhibit an unoccupied or partially occupied state (or LUMO) in Rh_SA_-MoS_x_Se_2-x_. Therefore, regulating Rh-MoS_x_Se_2-x_ orbital coupling may facilitate charge transfer and effectively modulate the LUMO position of anchored Rh atoms. As shown in Supplementary Fig. 4c, the projected density of states (PDOS) analysis suggests the LUMO position of Rh atoms exhibits a volcano-type relationship with the S: Se ratio in MoS_x_Se_2-x_ substrates. The higher LUMO of Rh atoms on MoSSe exhibits smaller energy gaps (∆*E*_1_) with the HOMO of OH^-^ and larger energy gaps (∆*E*_2_) with the HOMO of H_2_ molecules (Fig. 1c), which might directly alter the adsorption energies of

key intermediates through systematic tuning of the S: Se ratio in the support. Moreover, the S: Se ratio in Rh_SA_-MoS_x_Se_2-x_ series exhibits a volcano-type relationship with both the free energy of hydrogen adsorption (Δ*G*_H*_) and hydroxide adsorption (Δ*G*_OH*_), with Rh_SA_-MoSSe located at the volcano apex, displaying optimal adsorption strengths for both species (H and OH) (Fig. 1d and Supplementary Figs. 5 and 6). To further elucidate the impact of MSIs, we evaluated Δ*G*_H*_ and Δ*G*_OH*_ at all possible and thermodynamically stable adsorption sites on both pristine MoS_x_Se_2-x_ and regions surrounding the Rh atoms in Rh_SA_-MoS_x_Se_2-x_. It is worth noting that the introduction of Rh single-atom appears to substantially promote the adsorption of H and OH at Rh sites, synergistically improving both the thermodynamics and kinetics of HER (Supplementary Fig. 7). Gibbs free energy differences for the alkaline HER process in Rh_SA_-MoS_x_Se_2-x_ and MoS_x_Se_2-x_ were calculated in Supplementary Figs. 8–23. H_2_O adsorption at Rh sites in Rh_SA_-MoS_x_Se_2-x_ is thermodynamically barrier-free and more favorable than at S/Se sites in Rh_SA_-MoS_x_Se_2-x_ or MoS_x_Se_2-x_. The rate-determining step (RDS) for alkaline HER in both systems is H_2_O dissociation. Moreover, the water dissociation free energy of Rh_SA_-MoS_x_Se_2-x_ exhibits a volcano-type relationship with the S: Se ratio, with the lowest energy barrier observed at an S: Se ratio of 1: 1 (volcano’s apex). Significantly, the incorporation of a Rh single-atom in Rh_SA_-MoS_x_Se_2-x_ appears to lower the energy barriers for H_2_O dissociation, effectively promoting the water dissociation.

### Synthesis and characterizations of Rh_SA_-MoS_x_Se_2-x_ catalysts

Inspired by these DFT results, we synthesized Rh single atoms anchored on a series of MoS_x_Se_2-x_ with different S and Se ratios to validate the findings. The synthesis strategy for Rh_SA_-MoS_x_Se_2-x_ is illustrated in Supplementary Fig. 24. First, MoS_x_Se_2-x_ with different S and Se contents (denoted as MoS_x_Se_2-x_, 0 ≤ x ≤ 2) were prepared via a hydrothermal method followed by annealing. Then Rh single-atoms were anchored on the MoS_x_Se_2-x_ substrates (denoted as Rh_SA_-MoS_x_Se_2-x_, 0 ≤ x ≤ 2) using an electrochemical deposition method. The Rh loadings were comparable across the samples (Rh_SA_-MoS_2_: 0.37 wt.%, Rh_SA_-MoS_1.5_Se_0.5_: 0.42 wt.%, Rh_SA_-MoSSe: 0.49 wt.%, Rh_SA_-MoS_0.5_Se_1.5_: 0.44 wt.%, and Rh_SA_-MoSe_2_: 0.39 wt.%), as determined by inductively coupled plasma optical emission spectrometry (ICP-OES) (Supplementary Fig. 25). Given that all Rh_SA_-MoS_x_Se_2-x_ samples were synthesized using the same Rh precursor concentration, number of scanning cycles, and scanning rate, the observed variation in Rh loading is attributed to differences in substrates conductivity and the strong metal-support interactions^35^. The principle is as follows: According to the hard-soft acid-base (HSAB) principle^36–38^, the soft acid Rh³⁺ preferentially interacts with the softer base Se over S. Consequently, the higher conductivity of Se than S results in a higher Rh mass loading on MoSe_2_ than MoS_2_. Owing to the built-in electric field caused by the electron transfer from Se to S^39,40^, the MoS_x_Se_2-x_ with both S and Se anions exhibits superior conductivity and stronger metal-support interaction than MoS_2_ and MoSe_2_. In addition, the adsorption energies of Rh atoms on MoS_2_, MoSSe, and MoSe_2_ calculated by DFT are -3.66 eV, -3.91 eV, and -3.85 eV, respectively (Supplementary Fig. 26), indicating the stronger metal-support interaction between the Rh atom and MoSSe compared to both MoS_2_ and MoSe_2_. Therefore, the mass loading of Rh species on MoS_x_Se_2-x_ substrates is in the order of MoSSe > MoS_0.5_Se_1.5_ ≈ MoS_0.5_Se_1.5_ > MoSe_2_ > MoS_2_.

Scanning electron microscope (SEM) (Supplementary Fig. 27) and transmission electron microscopy (TEM) (Supplementary Figs. 28 and 29) characterizations revealed that all Rh_SA_-MoS_x_Se_2-x_ exhibit a similar nanoflower morphology, which promotes the exposure of active sites

and enhances mass transfer. Energy-dispersive X-ray (EDX) spectroscopy elemental analysis confirms the uniform distribution of Rh, Mo, and Se in Rh_SA_-MoS_x_Se_2-x_. As shown in Supplementary Figs. 30 and 31, the X-ray diffraction (XRD) patterns and corresponding Raman spectra indicate that all MoS_x_Se_2-x_ samples maintain the same crystal structures as their parent structures of 2H phase MoS_2_ (PDF#37-1492) and MoSe_2_ (PDF#20-0757) without the impurity phase. Moreover, all main diffraction peaks of MoS_x_Se_2-x_ are shifted toward a small angle with the Se concentration increasing, which is due to the gradual expansion of unit cells upon the substitution of S atoms by larger Se atoms^41,42^. Additionally, the synthesized Rh_SA_-MoS_x_Se_2-x_ possess the same crystal structure as the 2H phase of MoS_x_Se_2-x_^43,44^, and no distinct Rh-containing phases detected after Rh electrodeposition. In addition, high-resolution transmission electron microscopy (HRTEM) results (Supplementary Figs. 32 and 33) further confirm that the crystal structures of Rh_SA_-MoS_x_Se_2-x_ remain well-preserved following Rh decoration.

**Fig. 2:**
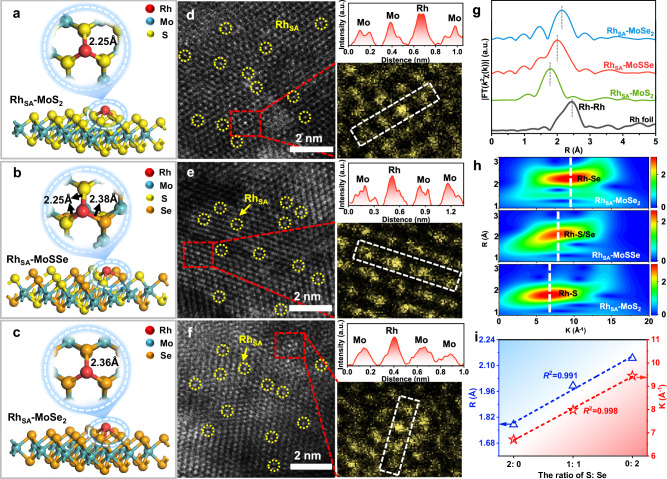
Structural characterization of Rh_SA_-MoS_x_Se_2-x_ catalysts. Structural models of **a** Rh_SA_-MoS_2_, **b** Rh_SA_-MoSSe, and **c** Rh_SA_-MoSe_2_. HAADF-STEM images of **d** Rh_SA_-MoS_2_, **e** Rh_SA_-MoSSe, and **f** Rh_SA_-MoSe_2_, with corresponding intensity profiles derived from the regions marked by red dashed rectangles (yellow dashed circles highlight selected single-atomic Rh sites). **g** Rh K-edge EXAFS spectra in R-space for Rh foil, Rh_SA_-MoS_2_, Rh_SA_-MoSSe, and Rh_SA_-MoSe_2_. **h** WT-EXAFS contour plots of Rh for Rh_SA_-MoS_2_, Rh_SA_-MoSSe, and Rh_SA_-MoSe_2_. **i** Correlation between the S: Se ratio, Rh-S/Se coordination path length, and the k-space positions in Rh_SA_-MoS_x_Se_2-x_. Source data are provided as a Source Data file.

The structural models of the Rh_SA_-MoS_2_, Rh_SA_-MoSSe, and Rh_SA_-MoSe_2_ catalysts are presented in Fig. 2a–c, respectively. The aberration-corrected high-angle annular dark-field scanning TEM (HAADF-STEM) detects the atomically dispersed Rh atoms (bright spots) on the MoS_x_Se_2-x_ nanosheets (Fig. 2d–f). Furthermore, the different intensity profiles along the dashed yellow rectangles in the HAADF-STEM images confirm the single-atom dispersion of Rh atoms. The Fourier transformed extended X-ray absorption fine spectroscopy (FT-EXAFS) spectra of Rh K-edge in Rh_SA_-MoS_2_, Rh_SA_-MoSSe, and Rh_SA_-MoSe_2_ all display a main characteristic peak below 3 Å (Fig. 2g), matching to the Rh-S/Se coordination^22,33^. In contrast to the Rh foil reference, the absence of a metallic Rh-Rh scattering signal rules out the presence of Rh nanoparticles or clusters, confirming the atomic dispersion of Rh species in Rh_SA_-MoS_2_, Rh_SA_-MoSSe, and Rh_SA_-MoSe_2_. The Rh K-edge EXAFS data fitting curves in Supplementary Fig. 34 and corresponding fitting data in Supplementary Table 1 unveiled the mean Ru-S/Se coordination number of the three catalysts was about 3.5, which was consistent with theoretical calculation models. Given the higher ionic radius and relative atomic mass of Se^2-^ compared to S^2–42,45^, the bond distance of Rh-S (2.3 Å) is shorter than Rh-Se (2.5 Å). Furthermore, Wavelet transformed EXAFS (WT-EXAFS) contour plots reveal single intensity maxima for Rh in Rh_SA_-MoS_2_, Rh_SA_-MoSSe, and Rh_SA_-MoSe_2_ at approximately 6.7, 8.0, and 9.4 Å^-1^ (Fig. 2h), corresponding to the Rh-S, Rh-S/Se, and Rh-Se coordination paths^34^, respectively. Notably, both the coordination path lengths and *k*-space positions of Rh-S/Se exhibit a linear correlation with the S: Se ratio in Rh_SA_-MoS_x_Se_2-x_ (Fig. 2i), providing further evidence that Rh single-atoms are covalently bonded to S/Se and that their coordination environment can be precisely tuned by adjusting the Se: S ratio in MoS_x_Se_2-x_ substrates.

**Fig. 3:**
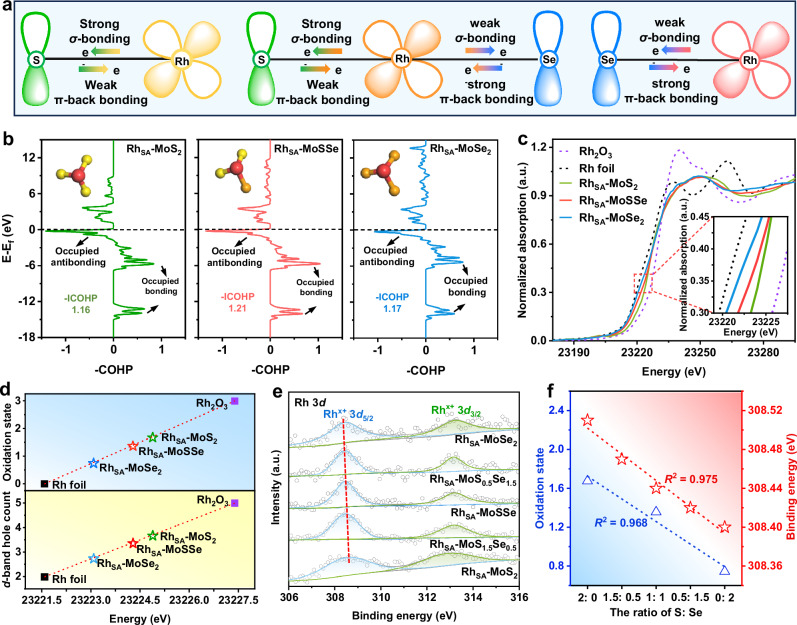
Electronic structure and composition analysis of Rh_SA_-MoS_x_Se_2-x_ catalysts. **a** Schematic depiction of electronic interactions among Rh, S, and Se in Rh_SA_-MoS_x_Se_2-x_. **b** The COHP analysis of Rh-S, Rh-S/Se, and Rh-Se bonds in Rh_SA_-MoS_2_ (left), Rh_SA_-MoSSe (middle), and Rh_SA_-MoSe_2_ (right). **c** Rh K-edge XANES spectra for Rh_SA_-MoS_2_, Rh_SA_-MoSSe, and Rh_SA_-MoSe_2_. **d** Fitted average oxidation states of Rh derived from XANES spectra. **e** Rh 3 *d* XPS spectra for Rh_SA_-MoS_2_, Rh_SA_-MoS_1.5_Se_0.5_, Rh_SA_-MoSSe, Rh_SA_-MoS_0.5_Se_1.5_, and Rh_SA_-MoSe_2_. **f** Correlation between the S: Se ratio, Rh oxidation state, and Rh 3*d*_5/2_ binding energy in Rh_SA_-MoS_x_Se_2-x_. Source data are provided as a Source Data file.

As shown in Fig. 3a, the synergistic electronic interactions among Rh, S, and Se in Rh_SA_-MoS_x_Se_2-x_ are characterized through Rh-S, S-Rh-Se, and Rh-Se coordination units. Owing to the higher polarizability volume and larger covalent radii of Se than S^41,42,45^, the closer size match between S 3*p* and Rh 4 *d* orbitals and the shorter Rh-S bond facilitates more effective orbital overlap, resulting in strong σ-bonding and weak π-back bonding in Rh-S bond. Moreover, the higher polarizability and more diffuse 4*p* orbitals of Se enhance its π-acceptor capacity, leading to stronger π-back bonding and weak σ-bonding in Rh-Se bond. The bonding characteristics and strength of the Rh-S and Rh-Se bonds in Rh_SA_-MoS_x_Se_2-x_ were investigated using crystal orbital Hamilton population (COHP) and integrated COHP (ICOHP) analysis (Fig. 3b). The COHP curves of All three systems show dominant bonding states below the Fermi level, confirming stable Rh-S and Rh-Se bonds^46,47^, with ICOHP values of -1.16 (Rh_SA_-MoS_2_), -1.21 (Rh_SA_-MoSSe), and -1.17 (Rh_SA_-MoSe_2_). Notably, the more negative ICOHP value of Rh-MoSSe indicates a stronger covalent interaction due to the asymmetric structure that polarizes the electron density and enhances orbital overlap. However, the three systems exhibit slightly different anti-bonding characteristics near the Fermi level. Rh_SA_-MoS_2_ displays more pronounced anti-bonding features suggestive of partial occupation of destabilizing states than Rh_SA_-MoSe_2_, while Rh_SA_-MoSSe exhibits an intermediate level that reflects its asymmetric chalcogen environment. Charge density difference (CDD) analyses reveal charge redistribution between Rh single-atoms and coordinated S/Se atoms^48,49^, resulting in a marked reduction of electron density around Rh atoms (Supplementary Fig. 35a). Bader charge analysis quantifies such electron transfer, affording positive values of 0.22 |e| (Rh-MoS_2_), 0.14 |e| (Rh-MoSSe), and 0.01 |e| (Rh-MoSe_2_), which further confirm the electron donation from Rh to the substrates. The substantial transfer in Rh-MoS₂ (0.22 |e | ) signifies strong ionic character, while the negligible transfer in Rh-MoSe₂ (0.01 |e | ) confirms a nearly non-ionic interaction. Thus, the decreasing Bader charges quantitatively capture the transition from polar ionic-covalent bonding in Rh-MoS_2_ to weak covalent bonding in Rh-MoSe_2_, fully consistent with the CDD observations.

X-ray photoelectron spectroscopy (XPS) and X-ray absorption near-edge structure (XANES) analyses were performed to elucidate the electronic structures and chemical compositions of Rh_SA_-MoS_x_Se_2-x_ catalysts. Rh K-edge XANES spectra (Fig. 3c) reveal a positive shift in edge absorption energies of Rh single-atoms with increasing Se content, confirming effective modulation of the Rh electronic structure through variation of the Se: S ratio. Quantitative analysis of the XANES data indicates oxidation states of Rh single-atom in Rh_SA_-MoS_2_, Rh_SA_-MoSSe, and Rh_SA_-MoSe_2_ are +1.67, +1.36, and +0.74, respectively (Fig. 3d). Relative to Rh^0^ foil (4*d*^8^5*s*^1^) and Rh^Ⅲ^_2_O_3_ (4*d*^5^5*s*^1^) standards, the *d*-band hole count for Rh_SA_-MoSSe is estimated to be 3.36, lower than that of Rh_SA_-MoS_2_ (3.67) but higher than Rh_SA_-MoSe_2_ (2.74), reflecting moderate *d*-orbital vacancy induced by *d*-*p* orbital hybridization in Rh_SA_-MoS_x_Se_2-x_^50^. High-resolution Rh 3 *d* XPS spectra (Fig. 3e) show a negative shift in binding energies with increasing Se contents, consistent with XANES results. Both the Rh oxidation state and Rh 3*d*_5/2_ binding energy exhibit a linear correlation with the S: Se ratio in Rh_SA_-MoS_x_Se_2-x_ (Fig. 3f). High-resolution Mo 3 *d*, S 2*p*, and Se 3 *d* spectra further confirm

the successful synthesis of Rh_SA_-MoS_x_Se_2-x_ variants with distinct S:Se ratios^43,50^ (Supplementary Fig. 35b–d). Collectively, these electronic structure characterizations demonstrate that the *d*-band structure of Rh single-atoms can be precisely tuned by modulating the S: Se ratio of MoS_x_Se_2-x_ supports via MSIs. The electronic properties of Rh_SA_-MoS_x_Se_2-x_ and MoS_x_Se_2-x_ were further probed through work function (*W*_f_) measurements using ultraviolet photoelectron spectroscopy (UPS). As shown in Supplementary Fig. 36, *W*_f_ displays a volcano-type dependence on the S: Se ratio for both Rh_SA_-MoS_x_Se_2-x_ and MoS_x_Se_2-x_, with Rh_SA_-MoSSe exhibiting a significantly lower *W*_f_ (3.54 eV) compared to Rh_SA_-MoS_2_ (3.75 eV), Rh_SA_-MoSe_2_ (4.04 eV), and pristine MoS_x_Se_2-x_ substrates (5.51 ~ 5.71 eV). The incorporation of Rh single atoms and the precise tuning of the S: Se ratio in MoSxSe2-x markedly enhance electron transfer capabilities, thereby facilitating the adsorption and activation of reactants.

### Electrocatalytic HER performance of Rh_SA_-MoS_x_Se_2-x_

**Fig. 4:**
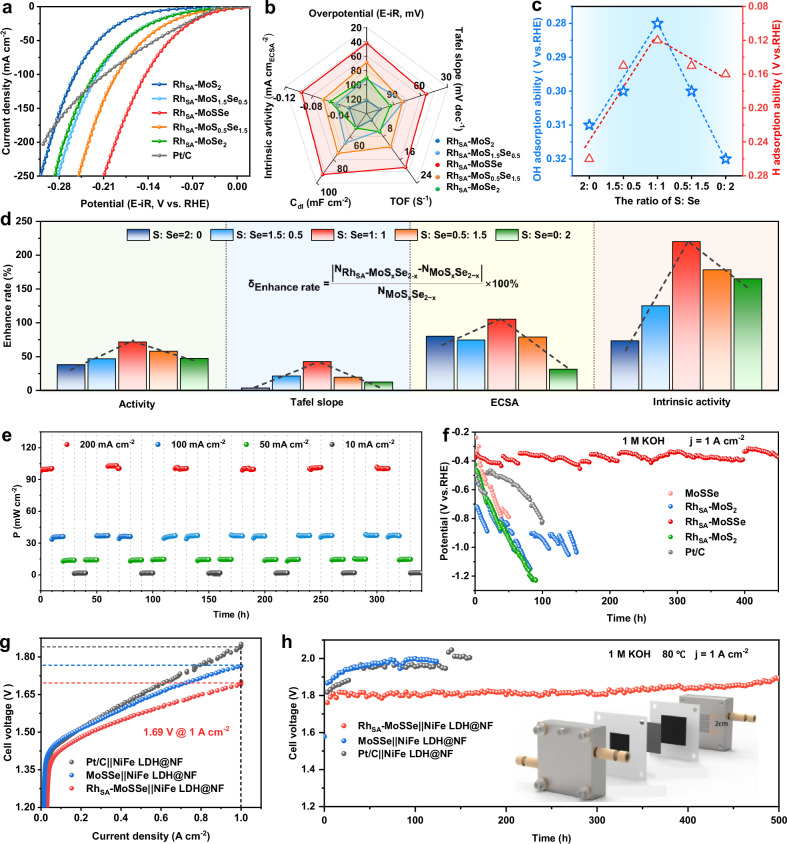
Electrocatalytic HER performance of Rh_SA_-MoS_x_Se_2-x_ measured in Ar-saturated 1 M KOH at 25 °C. **a** HER polarization curves for Rh_SA_-MoS_2_, Rh_SA_-MoS_1.5_Se_0.5_, Rh_SA_-MoSSe, Rh_SA_-MoS_0.5_Se_1.5_, Rh_SA_-MoSe_2_, and Pt/C catalysts, with 95% *iR* compensation. The electrolyte resistance (R_s_) is 1.4 ohm and the electrode surface area is 1 cm^-2^. **b** Radar chart illustrating Overpotential (at 10 mA cm^-2^), Tafel slope, TOF (at −100 mV vs. RHE), *C*_dl_, and intrinsic activity for Rh_SA_-MoS_2_, Rh_SA_-MoS_1.5_Se_0.5_, Rh_SA_-MoSSe, Rh_SA_-MoS_0.5_Se_1.5_, and Rh_SA_-MoSe_2_. **c** Correlation between the S: Se ratio and the H and OH adsorption strengths of Rh_SA_-MoS_x_Se_2-x_. **d** Enhancement rates of Rh_SA_-MoS_x_Se_2-x_ relative to pristine MoS_x_Se_2-x_ in terms of HER activity, Tafel slope, ECSA, and intrinsic activity. **e** Stability evaluation of Rh_SA_-MoSSe under step-change current density of 10, 50, 100, and 200 mA cm^-2^. **f** Chronoamperometric (CP) curves of Pt/C, MoSSe, Rh_SA_-MoS_2_, Rh_SA_-MoSSe, and Rh_SA_-MoSe_2_ at a current density of 1 A cm^-2^. **g** Polarization curve of Rh_SA_-MoSSe | |NiFe LDH@NF, MoSSe | |NiFe LDH@NF, and Pt/C | |NiFe LDH@NF in the AEMWE operated in 1 M KOH at 80 °C. **h** Stability test of Rh_SA_-MoSSe | |NiFe LDH@NF, MoSSe | |NiFe LDH@NF, and Pt/C | |NiFe LDH@NF in the AEMWE at 1 A·cm^-2^. Inset: schematic diagram of an AEMWE. Source data are provided as a Source Data file.

Electrochemical measurements were performed to elucidate the impact of the *d*-band structure of Rh single atoms on alkaline HER performance. As shown in the 95% *iR*-compensated linear sweep voltammetry (LSV) curves (Fig. 4a and Supplementary Fig. 37c and d), Rh_SA_- MoSSe exhibits HER activity, achieving a low overpotential of 46 mV at a current density of 10 mA cm^-2^, outperforming Rh_SA_-MoS_2_ (122.5 mV), Rh_SA_-MoS_1.5_Se_0.5_ (65.5 mV), Rh_SA_-MoS_0.5_Se_1.5_ (70.5 mV), Rh_SA_-MoSe_2_ (91 mV), pristine MoS_x_Se_2-x_ substrates, and commercial Pt/C catalysts. Moreover, the LSV curves of all samples without *iR* calibration were provide in Supplementary Fig. 37a and b. Specifically, Rh_SA_-MoSSe demonstrates a high mass activity of 3.48 A mg^-1^ (normalized to Rh loading) at an overpotential of 100 mV, approximately 50-fold higher than that of commercial Pt/C (0.07 A mg^-1^). Tafel plots were analyzed to probe HER kinetics^51^ (Supplementary Fig. 37c and d), revealing that Rh_SA_-MoSSe possesses a significantly lower Tafel slope (56 mV dec^-1^) compared to Rh_SA_-MoS_2_ (111 mV dec^-1^), Rh_SA_-MoS_1.5_Se_0.5_ (94 mV dec^-1^), Rh_SA_-MoS_0.5_Se_1.5_ (84 mV dec^-1^), Rh_SA_-MoSe_2_ (99 mV dec^-1^), Pt/C (40-121 mV dec^-1^), and MoS_x_Se_2-x_ substrates, indicating accelerated Volmer reaction kinetics. Moreover, we have added the relationship between the computed ∆*G*(H_2_O) and experimental overpotential required to achieve a current density of 10 mA cm^-2^ (*ŋ*_10_) based on the Brønsted-Evans-Polanyi (BEP) relationship^7^. As shown in Supplementary Fig. 40c, a lower ∆*G*(H_2_O) corresponds to a smaller overpotential and thus significantly enhanced alkaline HER activity, suggesting the critical role of water dissociation kinetics in determining the overall alkaline HER performance. In this work, the water dissociation barrier of Rh_SA_-MoS_x_Se_2-x_ is limited to 0.74 eV due to the modulation scope of EMSI in TMD-based supports. Therefore, future high-performance alkaline HER electrocatalysts should be rationally designed by constructing active sites with intrinsically low water-dissociation energy and by optimizing electronic structures to further reduce Δ*G*(H_2_O), thereby promoting water-dissociation kinetics and enhancing alkaline HER activity.

The catalyst's mass activity is fundamentally determined by the product of the electrochemically active surface area (ECSA, normalized by mass) and the specific activity (SA, catalytic current normalized by ECSA)^52–54^. In contrast to the geometrical surface area, the ECSA represents the actual surface area accessible to the electrolyte. The ECSA were estimated via double-layer capacitances (*C*_dl_) measurements derived from cyclic voltammogram (CV) (Supplementary Figs. 38 and 39). Owing to the built-in electric field and in-plane dipole moment caused by the electron transfer from Se to S^39,40^, the MoS_x_Se_2-x_ with both S and Se anions exhibits superior conductivity and hydrophilic than MoS_2_ and MoSe_2_. Moreover, the introduction of Rh increases the number of active sites and enhances charge transfer efficiency. Therefore, Rh_SA_-MoSSe exhibits the highest *C*_dl_ (92.6 mF cm^-2^) compared to Rh_SA_-MoS_2_ (52.4 mF cm^-2^), Rh_SA_-MoS_1.5_Se_0.5_ (65.5 mF cm^-2^), Rh_SA_-MoS_0.5_Se_1.5_ (74.4 mF cm^-2^), Rh_SA_-MoSe_2_ (52.5 mF cm^-2^), and pristine MoS_x_Se_2-x_ substrates, suggesting a greater abundance of exposed active sites. Normalizing activity by ECSA thus offers a more accurate and meaningful metric for evaluating the catalytic properties of diverse electrocatalysts, accounting for variations in size, shape, morphology, topography, and porosity^55,56^. Therefore, polarization curves of Rh_SA_-MoS_x_Se_2-x_ and MoS_x_Se_2-x_ samples, normalized to ECSA, confirm the superior intrinsic activities of Rh_SA_-MoSSe (Supplementary Fig. 40).

The turnover frequency (TOF) of Rh_SA_-MoSSe at -100 mV vs. RHE (19.34 s^-1^) surpasses that of Rh_SA_-MoS_2_ (2.15 s^-1^), Rh_SA_-MoS_1.5_Se_0.5_ (6.05 s^-1^), Rh_SA_-MoS_0.5_Se_1.5_ (9.89 s^-1^), Rh_SA_-MoSe_2_ (6.97 s^-1^), and is competitive with most reported noble metal-based and transition metal dichalcogenide (TMD) catalysts^12,13,17,50,57–62^ (Supplementary Fig. 41d and Supplementary Table 2). Moreover, the faradaic efficiency of Rh_SA_-MoSSe surpasses that of Rh_SA_-MoS_2_ (2.15 s^-1^), Rh_SA_-MoS_1.5_Se_0.5_ (6.05 s^-1^), Rh_SA_-MoS_0.5_Se_1.5_ (9.89 s^-1^), Rh_SA_-MoSe_2_ (6.97 s^-1^), MoS_x_Se_2-x_ substrates, and Pt/C (Supplementary Fig. 41e). Notably, the S: Se ratio in Rh_SA_-MoS_x_Se_2-x_ exhibits a volcano-type relationship with HER activity, Tafel slope, *C*_dl_, intrinsic activity, and TOF, with Rh_SA_-MoSSe at the volcano’s apex displaying optimal overall HER performance (Supplementary Fig. 41a–c). This underscores the effectiveness of precisely tuning the *d*-band structure of Rh single-atoms to enhance HER activity and kinetics. To further quantify the impact of MSIs modulation via the S:Se ratio variation in MoS_x_Se_2-x_ supports, the enhancement rates of Rh_SA_-MoS_x_Se_2-x_ relative to pristine MoS_x_Se_2-x_ substrates were evaluated for activity, Tafel slope, ECSA, and intrinsic activity, based on the following equation:

$${\delta }_{{{\rm{Enhancerate}}}}=\frac{\left|{N}_{{{{\rm{Rh}}}}_{{{\rm{SA}}}}-{{{\rm{MoS}}}}_{{{\rm{x}}}}{{{\rm{Se}}}}_{2-{{\rm{x}}}}}-{N}_{{{{\rm{MoS}}}}_{{{\rm{x}}}}{{{\rm{Se}}}}_{2-{{\rm{x}}}}}\right|}{{N}_{{{{\rm{MoS}}}}_{{{\rm{x}}}}{{{\rm{Se}}}}_{2-{{\rm{x}}}}}}\times 100\%$$

The enhancement rate (δ_Enhance rate_) is defined as the ratio of the performance metrics (activity, Tafel slope, ECSA, and intrinsic activity) for Rh_SA_-MoS_x_Se_2-x_ (*N*_RhSA-MoSxSe2-x_) relative to those for pristine MoS_x_Se_2-x_ (*N*_MoSxSe2-x_). Notably, the enhancement rates for activity, Tafel slope, ECSA, and intrinsic activity exhibit a volcano-type dependence on the S: Se ratio, with the highest enhancement rate observed at an S: Se ratio of 1: 1 (volcano’s apex) (Fig. 4d). Moreover, the Pt and Ru single atoms anchored on MoS_x_Se_2-x_ were also successfully synthesized to demonstrate the generality of the design strategy. The XRD of Ru_SA_-MoSSe and Pt_SA_-MoS_x_Se_2-x_ was shown in Supplementary Fig. 42, confirming that no diffraction peaks of Ru or Pt nanoparticles were observed. Additionally, the SEM and corresponding elemental mapping results reveals the uniform distribution of every element (Supplementary Figs. 43 and 44). Notably, the S: Se ratio in both Ru_SA_-MoS_x_Se_2-x_ and Pt_SA_-MoS_x_Se_2-x_ exhibits a volcano-type relationship with HER activity and Tafel slope, with Ru_SA_-MoSSe and Pt_SA_-MoSSe at the volcano’s apex displaying optimal overall HER performance (Supplementary Figs. 45 and 46).

The strong interaction between OH* species and catalysts surfaces is well-established for accelerating water dissociation in the HER^4,7^. To probe the OH adsorption capacity at CO adsorption sites^9^, CO-stripping voltammetry was employed to assess the water dissociation capabilities of the Rh_SA_-MoS_x_Se_2-x_ catalysts (Supplementary Fig. 47a). Among them, Rh_SA_-MoSSe exhibited the lowest onset potential for CO oxidation (0.28 V), compared to Rh_SA_-MoS_2_ (0.31 V), Rh_SA_-MoS_1.5_Se_0.5_ (0.30 V), Rh_SA_-MoS_0.5_Se_1.5_ (0.30 V), and Rh_SA_-MoSe_2_ (0.32 V), indicating a stronger Rh-OH interaction and enhanced water dissociation kinetics. The H adsorption ability of Rh_SA_-MoS_x_Se_2-x_ catalysts was evaluated through the desorption of underpotential-deposited hydrogen (H_upd_)^9,10,63^. As shown in Supplementary Fig. 47b, Rh_SA_-MoSSe displayed a notably low H_upd_ potential (0.12 V), compared to Rh_SA_-MoS_2_ (0.26 V), Rh_SA_-MoS_1.5_Se_0.5_ (0.15 V), Rh_SA_-MoS_0.5_Se_1.5_ (0.15 V), and Rh_SA_-MoSe_2_ (0.16 V), facilitating H recombination during HER. Furthermore, the S: Se ratio in Rh_SA_-MoS_x_Se_2-x_ catalysts exhibited a volcano-type correlation with both OH and H adsorption strengths, with Rh_SA_-MoSSe at the volcano’s apex demonstrating optimal adsorption capabilities for both H and OH (Fig. 4c), consistent with theoretical predictions. The catalyst’s stability across varying current densities was evaluated through multi-step current experiments, where the potential rapidly stabilized at each step, indicating efficient mass transport at the electrode surface during the HER (Fig. 4e). Long-term durability of Rh_SA_-MoSSe was assessed via chronopotentiometry, demonstrating negligible potential decay over 450 h at a high current density of 1 A cm^-2^, outperforming the commercial Pt/C, MoSSe, Rh_SA_-MoS_2_, and Rh_SA_-MoSe_2_ (Supplementary Fig. 48). The experimental findings are fully consistent with the trends predicted by the above DFT calculations. Moreover, the stability time of Rh_SA_-MoSSe at 1 A cm^-2^ is competitive with most reported noble metal-based and transition metal dichalcogenide (TMD) catalysts (Fig. 4f and Supplementary Table 3)^13,17,51,57–59,64–76^. These findings underscore the catalyst’s robust electrochemical stability and potential for industrial applications. Structural integrity of Rh_SA_-MoSSe post long-term HER testing was investigated using XRD, SEM, HAADF-STEM, and XPS. XRD analysis (Supplementary Fig. 49) confirmed that the crystal structure of Rh_SA_-MoSSe remained intact after extended HER operation. SEM (Supplementary Fig. 50) and HAADF-STEM (Supplementary Fig. 51) images verified that Rh_SA_-MoSSe retained its original morphology and atomic dispersion of Rh after 500 h testing. XPS analysis (Supplementary Fig. 52) further revealed that the oxidation states of Rh, Mo, S, and Se in Rh_SA_- MoSSe remained stable and unaltered post testing. Collectively, these structural characterizations demonstrate that Rh_SA_-MoSSe exhibits HER activity and stability, attributed to the precise tuning of the Rh single-atoms *d*-band structure, which optimizes intermediate adsorption energies and significantly enhances alkaline HER performance.

The anion exchange membrane water electrolyzer (AEMWE) tests were employed to further evaluate the application potential of Rh_SA_-MoSSe catalyst for industrial water splitting. The homemade NiFe LDH@NF as an anode electrocatalyst (Supplementary Fig. 53) was assembled with Rh_SA_-MoSSe and operated in 1.0 M KOH electrolyte at 80 °C. The polarization curves showed the Rh_SA_-MoSSe | |NiFe LDH@NF exhibit a cell voltage of 1.58 V and 1.70 V at current densities of 0.5 A cm^-2^ and 1.0 A cm^-2^ for AEMWE, respectively, which is superior to commercial Pt/C | |NiFe LDH@NF and MoSSe | |NiFe LDH@NF systems (Fig. 4g). Furthermore, the AEMWE of Rh_SA_-MoSSe | |NiFe LDH@NF was continuously operated for over 500 h at a current density of 1 A cm^-2^ (Fig. 4h). The overpotential at 1.0 A cm^-2^ and stability time of were comparable to those of recently reported catalysts for AEMWE (Supplementary Table 4)^9,59,66–70,73,76,77^. The above results confirm the advanced and reliable potential of the Rh_SA_-MoSSe catalyst for industrial water-splitting applications.

### Mechanism insights into catalytic activity

**Fig. 5:**
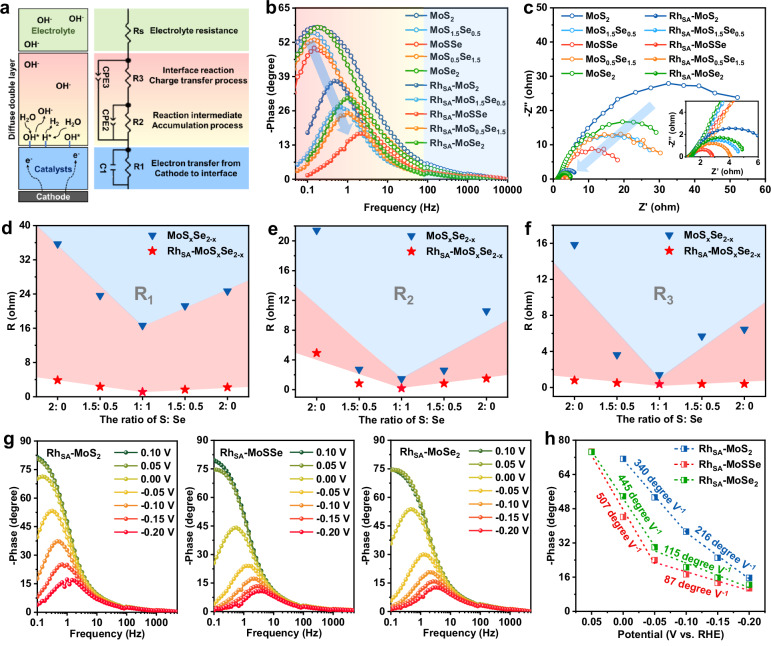
Electron transfer characteristics of Rh_SA_-MoS_x_Se_2-x_ catalysts. **a** Equivalent circuit-fitted EIS date and schematic representation of the mass and charge transfer processes for Rh_SA_-MoS_x_Se_2-x_ and pristine MoS_x_Se_2-x_. **b** Corresponding Bode plots and **c** Nyquist plots of Rh_SA_-MoS_x_Se_2-x_ and MoS_x_Se_2-x_ at −0.1 V vs. RHE. Correlation between the S: Se ratio and the resistance (R) components **d** R_1_, **e** R_2_, and **f** R_3_ in Rh_SA_-MoS_x_Se_2-x_ and MoS_x_Se_2-x_. **g** In situ bode plots for Rh_SA_-MoS_2_, Rh_SA_-MoSSe, and Rh_SA_-MoSe_2_. **h** Frequency dependence of phase changes in Bode plots for Rh_SA_-MoS_2_, Rh_SA_-MoSSe, and Rh_SA_-MoSe_2_. Source data are provided as a Source Data file.

Operando electrochemical impedance spectroscopy (EIS) was conducted to probe charge-transfer kinetics and elucidate HER^78^. The equivalent circuit for both Rh_SA_-MoS_x_Se_2-x_ and pristine MoS_x_Se_2-x_ comprises four components: electron transfer resistance (R) from the cathode to the interface (R_1_, part 1), accumulation of reaction intermediate (Volmer step, R_2_, part 2), charge transfer during the interfacial reaction (Heyrovsky step, R_3_, part 3), and electrolyte resistance (R_s_, part 4) (Fig. 5a). The low-frequency region is primarily associated with the Volmer step, while the high-frequency region reflects electron transfer from the catalyst’s inner layer to surface-active sites, driven by distinct relaxation time. Bode plots at overpotential of 100 mV (Fig. 5b) reveal significantly reduced phase angles at low frequencies for Rh_SA_-MoS_x_Se_2-x_ compared to MoS_x_Se_2-x_ supports, indicating that Rh single-atoms enhance the Volmer step kinetics. Analysis of Nyquist plots and optimized fitting parameters (Fig. 5c–f) shows that Rh_SA_-MoS_x_Se_2-x_ catalysts exhibit substantially lower charge transfer resistances than their pristine MoS_x_Se_2-x_ counterparts. Notably, R_1_, R_2_, and R_3_ display a volcano-type dependence on the S: Se ratio in both Rh_SA_-MoS_x_Se_2-x_ and MoS_x_Se_2-x_, with Rh_SA_-MoSSe at the volcano’s top exhibiting the lowest R_1_, R_2_, and R_3_ values, signifying accelerated HER charge transfer kinetics and a rapid Faradaic reaction at the catalyst-electrolyte interface. Operando EIS measurements at varying applied biases further clarify the charge transfer dynamics and HER mechanism of Rh_SA_-MoS_x_Se_2-x_. Bode plot (Fig. 5g) for Rh_SA_-MoS_2_, Rh_SA_-MoSSe, and Rh_SA_-MoSe_2_ reveal a single phase-angle peak spanning low-frequency (Volmer step) and mid-frequency (Heyrovsky step) regions as the applied bias decreases, suggesting that Rh_SA_-MoS_x_Se_2-x_ follows a mixed Heyrovsky-Volmer and Tafel-Volmer mechanism rather than a singular Heyrovsky-Volmer pathway. Notably, Rh_SA_-MoSSe exhibits a smaller phase angle and a faster phase-angle decrease rate (0.05 to -0.05 V) compared to Rh_SA_-MoS_2_ and Rh_SA_-MoSe_2_ (Fig. 5h), further confirming its superior HER kinetics.

**Fig. 6:**
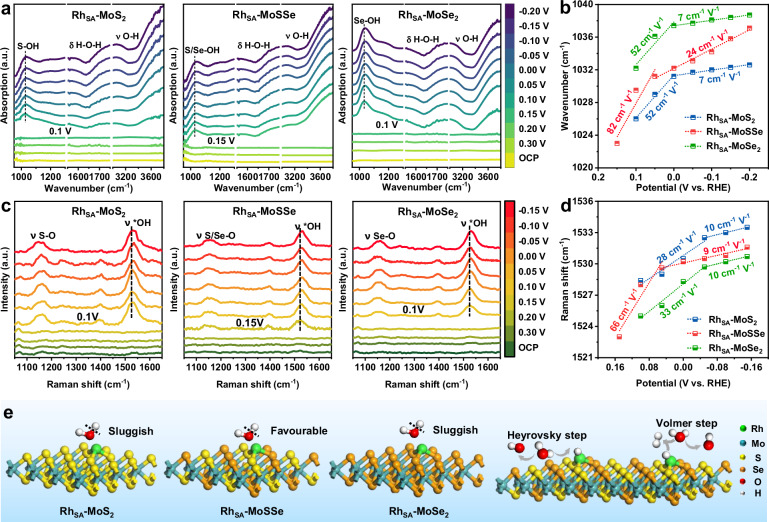
Mechanism insights into catalytic activity. **a** Operando attenuated total reflectance Fourier-transform infrared (ATR-FTIR) spectra of Rh_SA_-MoS_2_, Rh_SA_-MoSSe, and Rh_SA_-MoSe_2_. **b** Frequency dependence of S/Se-OH modes variations in ATR-FTIR spectra at different potentials. **c** In situ Raman spectra of Rh_SA_-MoS_2_, Rh_SA_-MoSSe, and Rh_SA_-MoSe_2_. **d** Frequency dependence of stretching mode variations in Raman spectra at different potentials. **e** Schematic illustration of the water dissociation trends for Rh_SA_-MoS_2_, Rh_SA_-MoSSe, and Rh_SA_-MoSe_2_. Source data are provided as a Source Data file.

To elucidate the enhanced alkaline HER activity of Rh_SA_-MoSSe, operando infrared absorption (IR) spectroscopy and in situ Raman spectroscopy were employed to investigate adsorption site and binding energy dynamics of reaction intermediates under HER operating conditions (Supplementary Figs. 54 and 55). As shown in Fig. 6a, with decreasing bias potential, distinct absorption peaks emerged at approximately 1030, 1621, and 3200 ~ 3600 cm^-1^. The peak at ~1030 cm^-1^ is attributed to S/Se-OH formation, while the broad peak at 3200 ~ 3600 cm^-1^ and the peak located at ~1621 cm^-1^ for Rh_SA_-MoS_2_, Rh_SA_-MoSSe, and Rh_SA_-MoSe_2_ correspond to the O-H stretching and *δ*(H-O-H) bending modes of interfacial water molecules, respectively^63,79^. Moreover, water-related peaks for Rh_SA_-MoSSe appeared at a more positive potential (0.15 V) compared to Rh_SA_-MoS_2_ (0.1 V) and Rh_SA_-MoSe_2_ (0.1 V), indicating that Rh_SA_-MoSSe facilitates water dissociation more effectively. The vibrational Stark effect, which describes potential-dependent shifts in adsorbate vibrational frequencies^3^, reveals that S/Se-OH in Rh_SA_-MoSSe is more responsive to the local electric field than in Rh_SA_-MoS_2_ and Rh_SA_-MoSe_2_, as evidenced by steeper Stark slopes (Fig. 6b). In-situ Raman spectroscopy further probed the catalytic processes in Rh_SA_-MoS_x_Se_2-x_ during alkaline HER. As presented in Fig. 6c, Raman peaks at ~1522 and ~1395 cm^-1^ for Rh_SA_-MoSSe emerged at 0.15 V, corresponding to adsorbed OH species generated during the HER^58^. In contrast, OH-related Raman peaks for Rh_SA_-MoS_2_ and Rh_SA_-MoSe_2_ appeared at 0.1 V, underscoring the stronger OH binding and accelerated water dissociation kinetics of Rh_SA_-MoSSe. Additionally, the Stark slopes for ^*^OH in Rh_SA_-MoSSe indicate greater sensitivity to the local electric field compared to Rh_SA_-MoS_2_ and Rh_SA_-MoSe_2_ (Fig. 6d). These findings suggest that precise modulation of the S: Se ratio optimizes OH adsorption at Rh single atom sites, thereby enhancing H_2_O adsorption and dissociation (Fig. 6e).

## Discussion

In summary, we establish an anion-gradient-engineered Rh SACs platform (Rh_SA_-MoS_x_Se_2-x_) for precisely manipulating metal-support frontier orbital hybridization at the atomic scale. We demonstrate that the elevated LUMO of MoS_x_Se_2-x_ support promotes orbital hybridizations between Rh atoms and support, which not only enhances catalytic stability but also amends the LUMO of anchored Rh atoms to optimize both the OH and H adsorption for high activity. The apex catalyst, Rh_SA_-MoSSe, achieves optimized orbital hybridization, simultaneously balancing intermediate adsorption/desorption kinetics and strengthening Rh-S/Se covalent bonding, thereby delivering favorable HER performance, with a low overpotential of 46 mV at 10 mA cm^-2^ and long-term stability under industrially relevant fluctuating power conditions (> 300 h). This work establishes the fundamental link between orbital hybridization and electrocatalytic energetics, providing a universal design principle to overcome activity-stability trade-offs in single-atom catalysis.

## Methods

### Chemical and reagents

All chemicals were of reagent-grade and used without further purification. Hexaammonium heptamolybdate tetrahydrate ((NH_4_)_6_Mo_7_O_24_·4H_2_O, 99.9%), thiourea (NH_2_CSNH_2_, 99%), selenourea (NH_2_CSeNH_2_, 99%), potassium hexachlororhodate (K_3_RhCl_6_, 99.9), ruthenium trichloride (RuCl_3_, 99.9%), potassium tetrachloroplatinate (K_2_PtCl_4_, 99.9%), ferric nitrate nonahydrate (Fe(NO_3_)_3_·9H_2_O, 99.9%), nickel nitrate hexahydrate (Ni(NO_3_)_2_·6H_2_O, 99.9%), urea (CO(NH_2_)_2_, 99%), and potassium hydroxide (KOH, 95%) were obtained from Sigma-Aldrich. Deionized (DI) water was produced using a Millipore ultrapure water system.

### Preparation of MoS_x_Se_2-x_

MoS_x_Se_2-x_ was synthesized following previously reported methods with minor modification. To prepare MoSSe, 1 mmol of (NH_4_)_6_Mo_7_O_24_·4H_2_O, 1.25 mmol of NH_2_CSNH_2_, and 1.25 mmol of NH_2_CSeNH_2_ were dissolved in 30 mL of deionized water and stirred vigorously for 1 h to form a homogeneous solution. The solution was then transferred to a 50 mL Teflon-lined autoclave and maintained at 230 °C for 20 h. After cooling to room temperature (20-25 °C), the resulting precipitates were filtered, washed with ethanol and water, and dried at 60 °C overnight. Stable MoSSe was obtained by heat-treatment the metastable MoSSe solid solution at 300 °C. Similarly, MoS_2_, MoS_1.5_Se_0.5_, MoS_0.5_Se_1.5_, and MoSe_2_ nanosheets were synthesized using the same procedure, adjusting only the amounts of NH_2_CSNH_2_ and NH_2_CSeNH_2_.

### Preparation of Rh_SA_-MoS_x_Se_2-x_

Rh single atoms anchored on MoS_x_Se_2-x_ substrates (Rh_SA_-MoS_x_Se_2-x_) were synthesized using a universal electrodeposition method in a standard three-electrode system. Initially, 10 mg of each as-prepared MoS_x_Se_2-x_ catalysts was dispersed in 1 mL of ethanol containing 30 μL of Nafion solution and sonicated until a homogeneous ink was formed. Subsequently, 250 μL of the ink was drop-cast onto Ni foam (1×1 cm) and dried at room temperature (20-25 °C), resulting in a catalyst loading of 2.5 mg cm^-2^. A carbon rod and a saturated calomel electrode (SCE) were used as the counter and reference electrode, respectively. The electrolyte consisted of 200 μM Rh precursors and 1 M KOH. Electrochemical depositions were performed by sweeping the potential from 0.10 V to -0.55 V at a scan rate of 5 mV s^-1^ for cathodic deposition, with the process repeated five times. After depositions, the electrode was rinsed with deionized water and used directly for subsequent electrochemical measurements.

### Preparation of Ru_SA_-MoS_x_Se_2-x_ and Pt_SA_-MoS_x_Se_2-x_

The synthetic procedure of both Ru_SA_-MoS_x_Se_2-x_ and Pt_SA_-MoS_x_Se_2-x_ were similar with that of Rh_SA_-MoS_x_Se_2-x_, except that RuCl_3_ and K_2_PtCl_4_ was used instead of K_3_RhCl_6_, respectively.

### Preparation of NiFe LDH@NF

The NiFe LDH grown on Ni foam was synthesized through a hydrothermal reaction. Firstly, the solution was prepared by adding 0.5 mmol Ni(NO_3_)_2_·6H_2_O, 0.5 mmol Fe(NO_3_)_3_·9H_2_O, and 5 mmol CO(NH_2_)_2_ into 30 mL of water and transferred to an autoclave. Subsequently, the cleaned Ni foam was placed into the obtained solution. The hydrothermal reaction was maintained at 80 °C for 4 h. After cooling naturally to room temperature (20–25 °C), the sample was washed with ethanol and ultrapure water several times, then dried in air for further use.

### Electrode preparatory method and electrochemical measurement

Electrochemical measurement was performed using a three-electrode system on a CHI760E electrochemical workstation (Chenhua Shanghai) at room temperature (20-25 °C), a 50 mL non-sealed quartz cell (single compartment) was used. The working electrode was prepared by dispersing 10 mg of catalyst in 1 mL of ethanol containing 30 μL of Nafion solution, followed by sonication to form a homogeneous ink. Then, 250 μL of this ink was drop-cast dropwise onto a 1×1 cm Ni foam and dried at room temperature (20–25 °C), yielding a catalyst loading of 2.5 mg cm^-2^. A carbon rod and Hg/HgO electrode served as the counter and reference electrode, respectively. The 1 M KOH electrolyte was prepared by dissolving 59.1 g of KOH pellets in DI water to a total volume of 1 L. The dissolution has been carried out under stirring at room temperature (20–25 °C) until complete clarity has been achieved. The prepared electrolyte has been stored in a tightly sealed polyethylene bottle at room temperature (20–25 °C). No visible precipitation or change in concentration has been observed during the storage period. The solution has been used within one month after preparation. The pH value of 1 M KOH solution at 25 °C: 13.9 ± 0.07 (Supplementary Fig. 36e). The R_s_ in a three-electrode system at 25 °C: 4 ± 0.8 ohm (Supplementary Fig. 36d). We have converted all measured potentials to the reversible hydrogen electrode (RHE) scale using the following equation: *E*_RHE_ = *E*_measured_ + *E*^0^_reference_ + 0.059 × pH. Where *E*_measured_ is the experimentally recorded potential against the reference electrode, *E*^0^_reference_ is the standard potential of the reference electrode relative to the standard hydrogen electrode (SHE), and pH is the measured pH value of the electrolyte at 25 °C. The reference electrode used throughout the experiments has been a Hg/HgO electrode filled with 1 M KOH solution. Its exact potential relative to SHE has been +0.098 V at 25 °C. The pH of the 1 M KOH electrolyte has been measured as 13.9. Therefore, the conversion has been applied as: *E*_RHE_ = *E*_measured (vs. Hg/HgO)_  + 0.098 + 0.059 × 13.9.

HER activities were evaluated via linear sweep voltammetry (LSV) in 1 M KOH electrolyte at 25 °C, with all data corrected to the reversible hydrogen electrode (RHE) scale with 95% *iR* compensation applied. CO stripping experiments were conducted in 1.0 M KOH solution. The working electrode with catalysts was immersed in the electrolyte, and CO was purged for 30 min to achieve CO saturation. After CO adsorption at open circuit potential, the electrode was transferred to an Ar-saturated 1.0 M KOH electrolyte. CO stripping voltammetry was recorded between 0.02 and 0.8 V (vs. RHE) at a scan rate of 50 mV·s^-1^. Electrochemical impedance spectroscopy (EIS) was performed over a frequency range of 10^5^ to 0.1 Hz with a 5 mV amplitude. Long-term stability was assessed using chronopotentiometry at a constant current density of 10 mA cm^−2^. The electrochemically active surface area (ECSA) was determined by measuring the electrochemical double-layer capacitance (*C*_dl_). Cyclic voltammetry (CV) was conducted in a non-Faradaic region (0.37-0.47 V vs. RHE) at scan rates ranging from 20 to 100 mV s^-1^. The *C*_dl_ was calculated by plotting the current density difference (ΔJ) between anodic and cathodic sweeps at a fixed potential against the scan rate, where the slope represents *C*_dl_. The ECSA was calculated as ECSA = *C*_dl_/*C*_s_, where *C*_s_ is the specific capacitance of the material. A general specific capacitance value of *C*_s_ = 40 uF cm^−2^ in 1 M KOH was used, based on typical commonly reported values for surface area estimation.

Faradaic efficiency (FE) for H_2_ was calculated using the equation: FE = (*n* × *z* × *F*)/*Q* × 100%, where n is the moles of H_2_ measured by gas chromatograph (GC), *z* = 2, *F* = 96485 C·mol^-1^, and *Q* is the total charge passed. H_2_ was quantified by sampling the sealed cell headspace every 10 min and analyzing with a gas chromatograph (GC-TCD, Ar carrier). Ultra-pure argon (Ar, 99.999%) was used as the carrier gas. All measurements were repeated three times independently; data are reported as mean ± SD. Potential side products were monitored: O_2_ was quantified by GC-TCD (no significant amount detected), and H_2_O_2_ was tested using a colorimetric peroxidase assay. No other reducible impurities were present. The TOF values for all Rh_SA_-MoS_x_Se_2-x_ catalysts were derived from the formula TOF = *I* / (2 *F* × *n*), where *I* stands for the current measured during linear sweep scans, *F* is Faraday’s constant (96,500 C mol^-1^), and *n* is the number of moles of active Rh sites. The factor of 1/2 represents the two-electron requirement for the formation of each H_2_ molecule.

### Anion-exchange membrane water electrolyzer (AEMWE) test

AEMWE with a serpentine flow channel was used to test practical applications of RhSA-MoSSe electrocatalysts. The anode electrode was prepared by spraying Rh_SA_-MoSSe or Pt/C inks on Ni foam; the spraying was controlled to achieve a mass loading of 4.0 mg cm^-2^. The homemade self-supporting NiFe LDH@NF was used as an anode electrocatalyst. Ni foam was used as a cathode porous current collector. Sustainion®X37-50 Grade 60 anion exchange membrane (Dioxide Materials Corporation, thickness: 75 μm, dimension: 1.5×1.5 cm) was activated by soaking in a 1.0 M KOH solution for 24 hours under 80 °C before use. The AEMWE test was operated at 80 °C with a peristaltic pump pumping 1.0 M KOH at a flow rate of 40 ml·min^-1^. Polarization curves were obtained from 1 to 1.8 V at a stepwise rate of 5 mV s^-1^. A long-term stability test was conducted at a current density of 1 A·cm^-2^ at 80 °C.

### Structural characterization

X-ray diffraction (XRD) analysis was conducted using a Rigaku Ultima IV diffractometer (Japan). Scanning electron microscope (SEM) images were obtained with a Thermo Scientific Verios G4 UC microscope SEM. Transmission electron microscopy (TEM) was performed on a Thermo Scientific Talos F200X G2 TEM equipped with X-ray energy-dispersive spectroscopy (EDS). Atomic-resolution scanning TEM (AC-STEM) images were acquired using a JEOL JEM-ARM200F aberration-corrected microscope. Inductively coupled plasma optical emission spectrometry (ICP-OES) was carried out on an Agilent 7700 instrument (USA) to determine the composition of materials. X-ray photoelectron spectroscopy (XPS) was conducted using a Thermo Scientific K-Alpha spectrometer (USA) to analyze chemical valence states and surface atomic ratios, with calibration based on the C 1 s peak at 284.8 eV. Raman spectroscopic experiments were performed with a confocal microscope Raman system Xplora Plus (HORIBA France), the excitation wavelength of the semiconductor laser was 532 nm, and the laser power was 100 mW.

### XAFS measurements

Rh L-edge XAFS spectra were collected in transmission geometry at the BL14W1 beamline of the Shanghai Synchrotron Radiation Facility (SSRF, China), which employed a Si(111) double-crystal monochromator. During data acquisition, the SSRF storage ring was operating at 3.5 GeV with a peak current of 210 mA. Spectra were recorded under ambient conditions (room temperature, atmospheric pressure).

### In situ Raman measurements

In situ Raman experiments were performed on an Xplora Plus confocal Raman system (HORIBA France) using a 532 nm laser (100 mW) and a 50× objective. A custom-designed Raman cell was employed, with Rh_SA_-MoS_x_Se_2-x_ deposited on 1 × 1 cm Ni foam as the working electrode, a Pt wire as the counter electrode, and Hg/HgO as the reference electrode. The electrochemical potential was controlled by a CHI760E workstation (Chenhua, Shanghai).

### Operando FTIR measurements

Operando FTIR spectra were recorded using an INVENIO spectrometer (Bruker Optics) equipped with independent DigiTect detectors and Transit channels for rapid mid-IR acquisition. A three-electrode cell was employed, with Rh_SA_-MoS_x_Se_2-x_ on Ni foam (1×1 cm) as the working electrode, Ag/AgCl as the reference, and Pt foil as the counter. Background spectra were taken under open-circuit conditions. The spectral resolution was set to 4 cm⁻¹ for all measurements.

### Calculation details

Density functional theory (DFT) calculations were performed using the projector augmented-wave (PAW) pseudopotential method with the Perdew-Burke-Ernzerhof (PBE) exchange-correlation functionals, as implemented in the Vienna Ab-initio Simulation Package (VASP)^80–86^. A plane-wave cutoff energy of 400 eV was used for electronic structure calculations. The convergence criterion for electronic self-consistency was set to 10^-6 ^eV, and the force convergence threshold for structural optimization was set below 0.01 eV Å^-1^. The basal planes of Rh_SA_-MoS_2_, Rh_SA_-MoSSe, Rh_SA_-MoSe_2_, MoS_2_, MoSSe, and MoSe_2_ were simulated and modeled with vacuum thicknesses exceeding 15 Å. The free energy (*G*) was calculated as *E*_total_ + *ZPE* - *TS*, where *E*_total_ is DFT-calculated total energy, *ZPE* is the zero-point energy, *T* is temperature, and *S* is the entropy.

## Supplementary information

Supplementary Information

Description of Additional Supplementary Files

Supplementary Data 1

Transparent Peer Review file

## Source data

Source Data

## Data Availability

All data generated in this study are provided in the Article and its Supplementary Information. The raw data generated in this study are provided in the Source Data file. Source data are provided with this paper.
