# Supplementary material for: Frontier-orbital modulation of rhodium single-atom catalysts for enhanced hydrogen evolution
Source: Nat Commun. 2026 May 18;17:6523. doi: 10.1038/s41467-026-73161-6 (PMC13376774; doi:10.1038/s41467-026-73161-6)
Supplement: Supplementary file 1 — Supplementary Information [file 41467_2026_73161_MOESM1_ESM.pdf]

Supplementary Information

Frontier-Orbital Modulation of Rhodium Single-Atom Catalysts for Enhanced Hydrogen Evolution

Rouna Jia[1,2], Zongyan Liu[1,2], Yang Wang[1,2 *], Jingyang Zhao[1], Zhong Huang[3], Wen Yue[4], Haozhi Wang[1,2 *], Kaiping Yu[5], Mingxin Huang[5], Yida Deng[1,2 *]

[1] State Key Laboratory of Tropic Ocean Engineering Materials and Materials Evaluation, School of Materials Science and Engineering, Hainan University, Haikou, 570228, China;

[2] Key Laboratory of Pico Electron Microscopy of Hainan Province, Hainan University, Haikou, 570228, China;

[3] School of Information and Communication Engineering, Hainan University, Haikou, 570228, China;

[4] State Key Laboratory of Deep Earth Exploration and Imaging, School of Engineering and Technology, China University of Geosciences Beijing, Beijing 100083, China;

[5] Department of Mechanical Engineering, The University of Hong Kong, Hong Kong 999077, China.

[*]Corresponding authors.

E-mail addresses: yida.deng@tju.edu.cn (Yida Deng), yangwangwy@hainanu.edu.cn (Yang Wang), hzwang001@hainanu.edu.cn (Haozhi Wang).

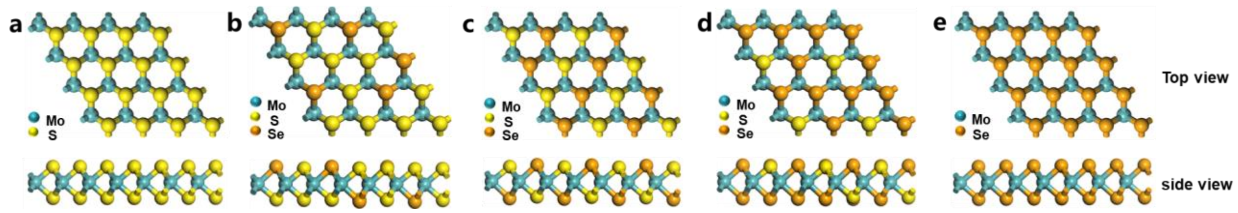

Supplementary Fig. 1. Structure models of MoS_xSe_{2-x} ($0 \leq x \leq 2$). Structure models of (a) MoS_2, (b) $MoS_{1.5}Se_{0.5}$, (c) MoSSe, (d) $MoS_{0.5}Se_{1.5}$, and (e) $MoSe_2$, depicted in top and side view. Blue, yellow, and orange spheres represent molybdenum (Mo), sulfur (S), and selenium (Se) atoms, respectively.

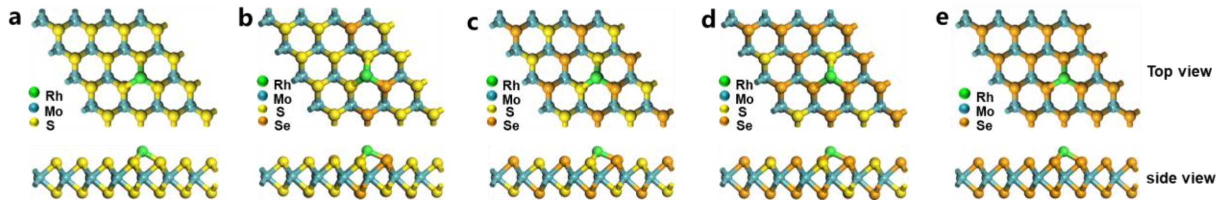

Supplementary Fig. 2. Structure models of Rh_{SA}-MoS_xSe_{2-x} ($0 \leq x \leq 2$). Structure models of (a) Rh_{SA}-MoS_2, (b) Rh_{SA}-$MoS_{1.5}Se_{0.5}$, (c) Rh_{SA}-MoSSe, (d) Rh_{SA}-$MoS_{1.5}Se_{0.5}$, and (e) Rh_{SA}-$MoSe_2$, presented in top and side view. Green, blue, yellow, and orange spheres represent rhodium (Rh), Mo, S, and Se atoms, respectively.

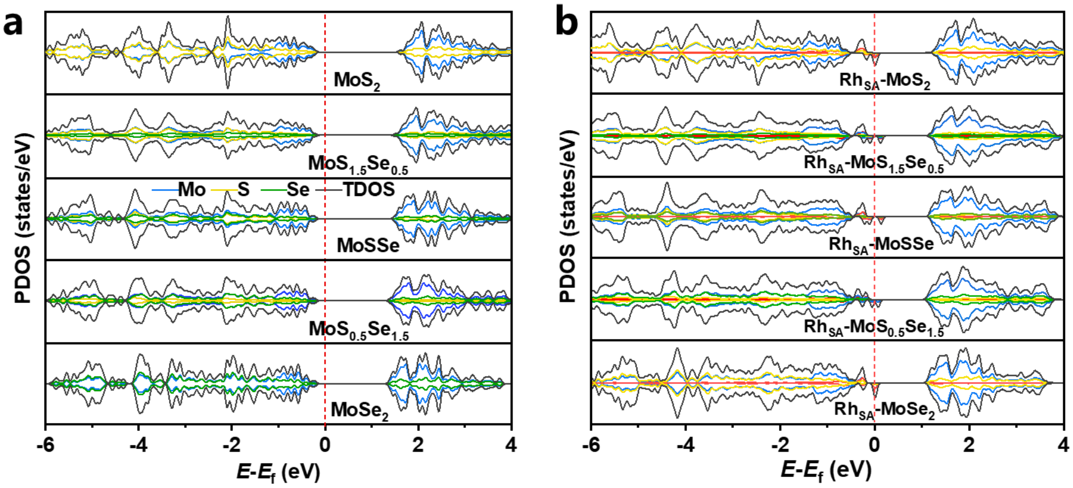

Supplementary Fig. 3. (a) Calculated density of states (DOS) for MoS_2, $MoS_{1.5}Se_{0.5}$, MoSSe, $MoS_{0.5}Se_{1.5}$, and $MoSe_2$. (b) Calculated DOS for Rh single-atom anchored systems: Rh_{SA}-MoS_2, Rh_{SA}-$MoS_{1.5}Se_{0.5}$, Rh_{SA}-MoSSe, Rh_{SA}-$MoS_{0.5}Se_{1.5}$, and Rh_{SA}-$MoSe_2$.

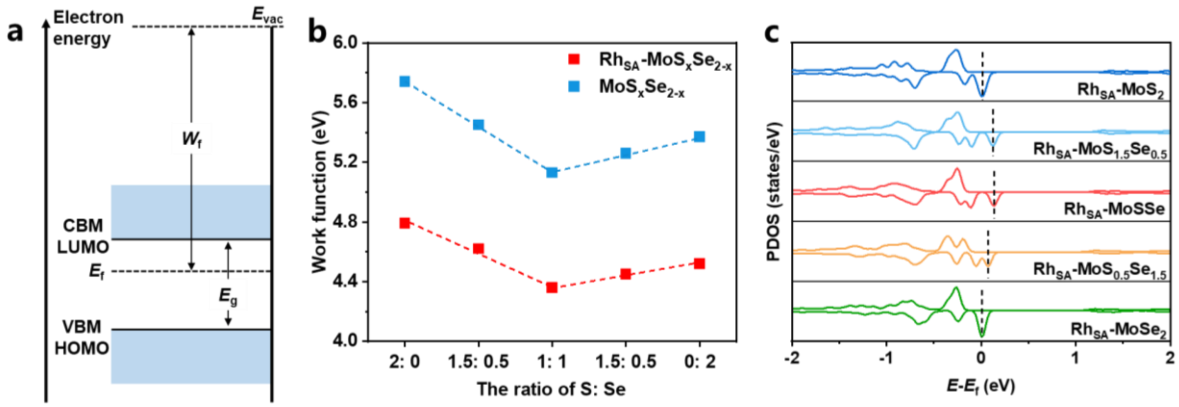

Supplementary Fig. 4. (a) Energy diagram of a semiconductor with flat bands to the surface. Band edges (CBM/LUMO and VBM/HOMO), vacuum level E_{vac}, work function W_f, and energy gap E_g are defined. (b) Dependence of the calculated work function on the S: Se ratio in MoS_xSe_{2-x} and Rh_{SA}-MoS_xSe_{2-x}. (c) Calculated PDOS of anchored Rh atoms in Rh_{SA}-MoS_xSe_{2-x}, where bleak dashed lines denote the LUMO of Rh.

Supplementary Note 1. The conversion from energy relative to the Fermi level (E-E_f) to energy relative to the vacuum level (E-E_{vac}) is a fundamental transformation in computational chemistry and materials science that establishes absolute energy references for molecular orbitals. This conversion is governed by the work function (Φ), which represents the minimum energy required to remove an electron from the Fermi level to the vacuum level, mathematically expressed as $\Phi = E_{vac} - E_f$. The fundamental conversion equation is $E - E_{vac} = (E - E_f) - \Phi$, where the work function serves as the critical bridge between these two energy reference systems. For molecular systems, the HOMO and LUMO energies can be transformed from their relative positions to the Fermi level to absolute energies relative to vacuum, providing a universal reference scale that facilitates comparison across different materials and experimental conditions. This conversion is particularly crucial in organic electronics and photovoltaic applications, where precise energy level alignment between donor and acceptor materials determines device performance.

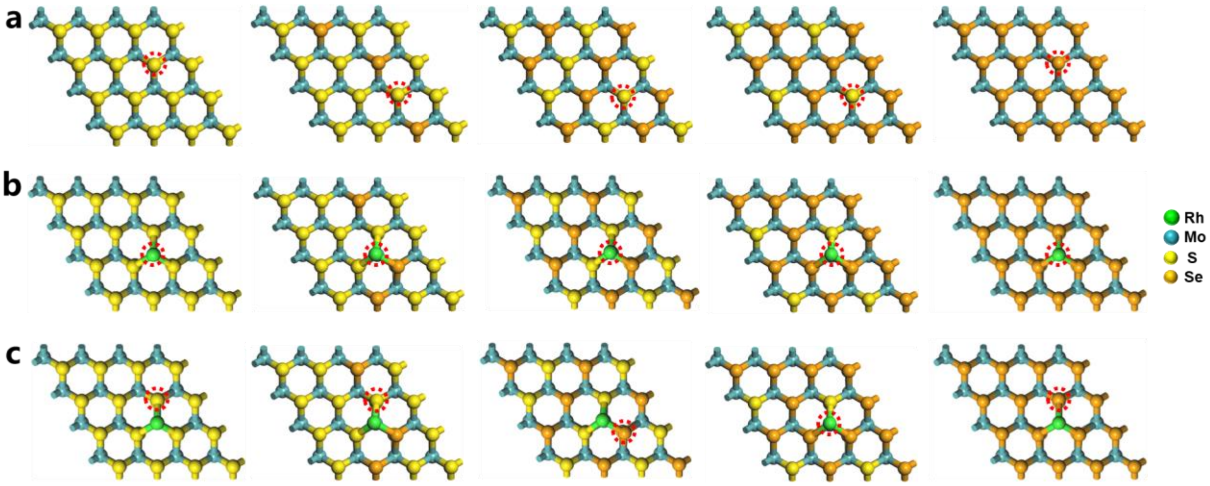

Supplementary Fig. 5. (a) S/Se sites for H adsorption in MoS_xSe_{2-x}. (b) Rh sites and (c) surrounding S/Se sites for H adsorption in Rh_{SA}-MoS_xSe_{2-x}.

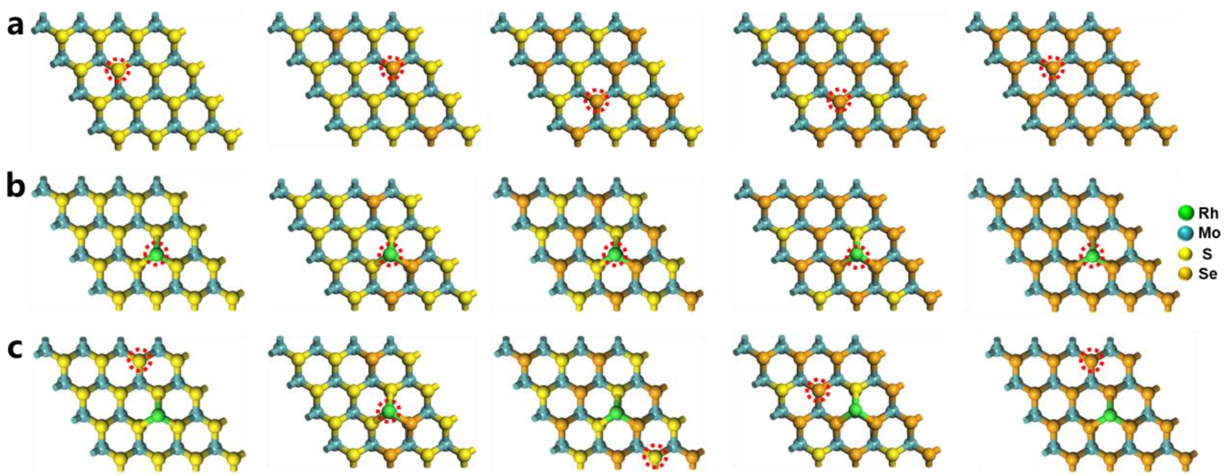

Supplementary Fig. 6. (a) S/Se sites for OH adsorption in MoS_xSe_{2-x}. (b) Rh sites and (c) surrounding S/Se sites for OH adsorption in Rh_{SA}-MoS_xSe_{2-x}.

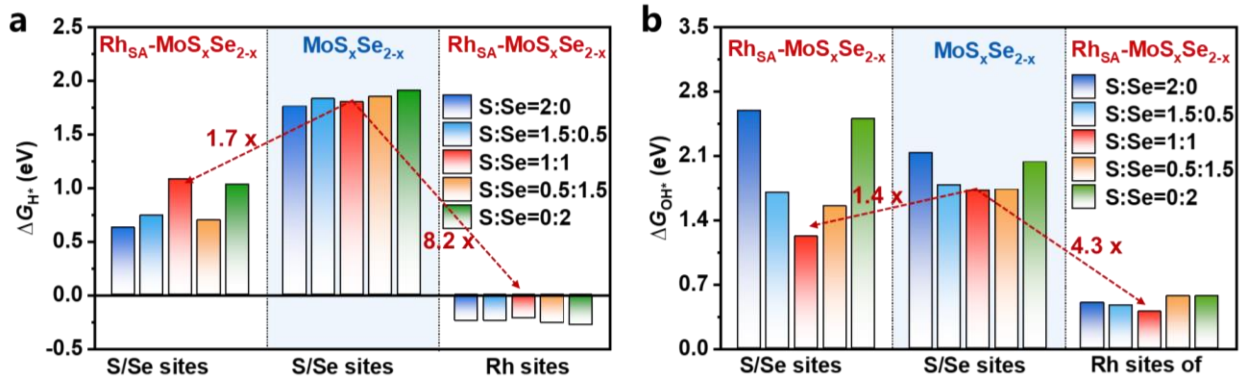

Supplementary Fig. 7. (a) H adsorption strength and (b) OH adsorption strength at S/Se sites in pristine MoS_xSe_{2-x}, Rh sites, and adjacent S/Se sites in Rh_{SA}-MoS_xSe_{2-x}.

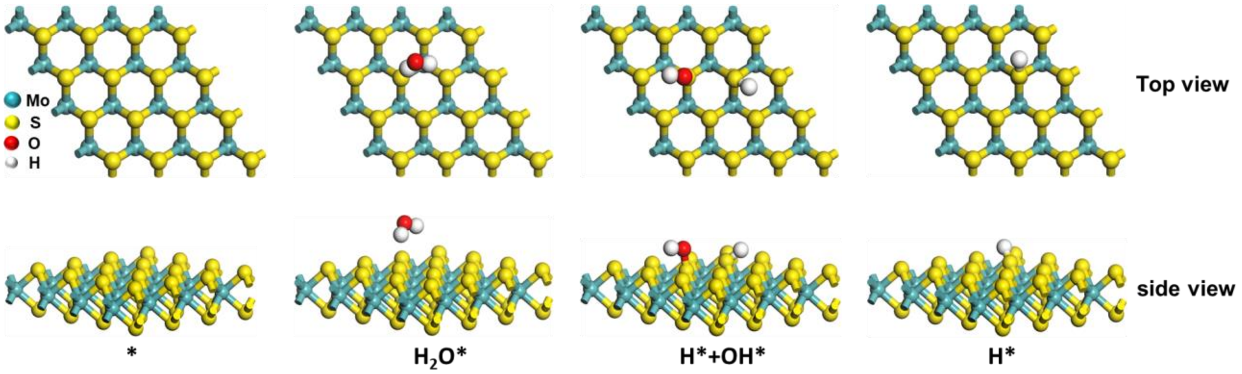

Supplementary Fig. 8. Diagram illustrating the HER at S sites on MoS_2.

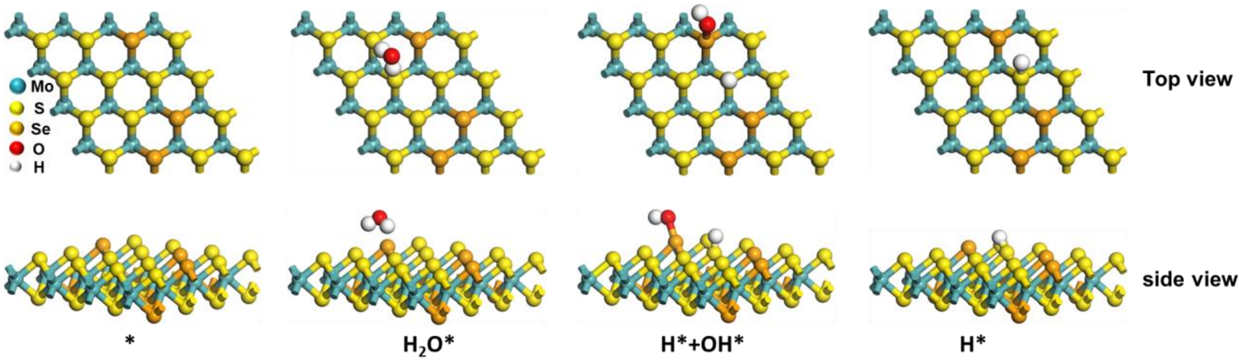

Supplementary Fig. 9. Diagram illustrating the HER at S/Se sites on $MoS_{1.5}Se_{0.5}$.

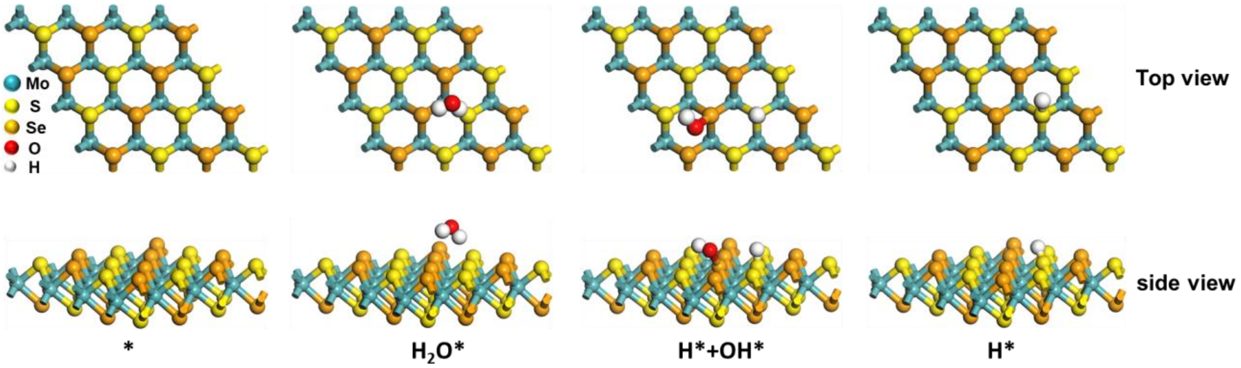

Supplementary Fig. 10. Diagram illustrating the HER at S/Se sites on MoSSe.

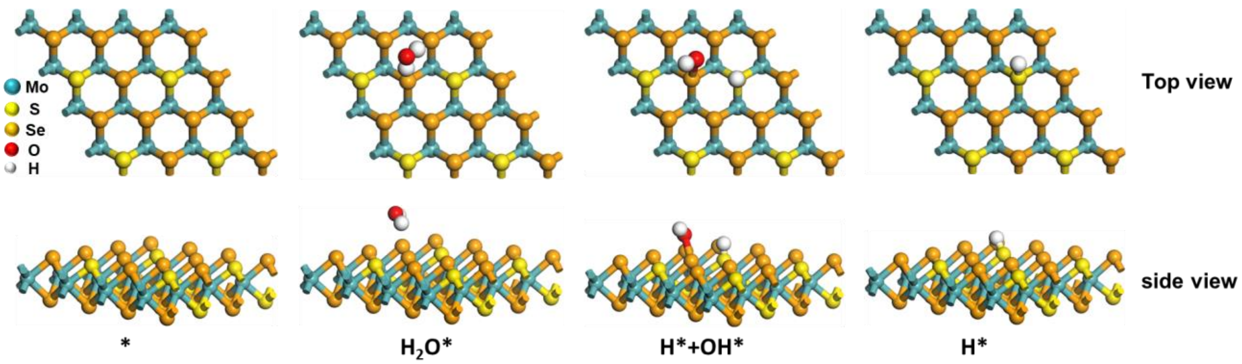

Supplementary Fig. 11. Diagram illustrating the HER at S/Se sites on $MoS_{0.5}Se_{1.5}$.

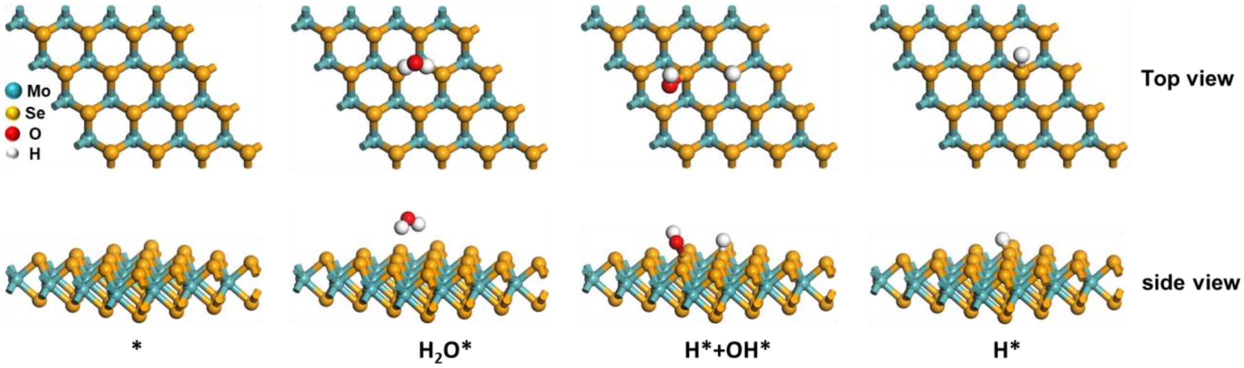

Supplementary Fig. 12. Diagram illustrating the HER at Se sites on $MoSe_2$.

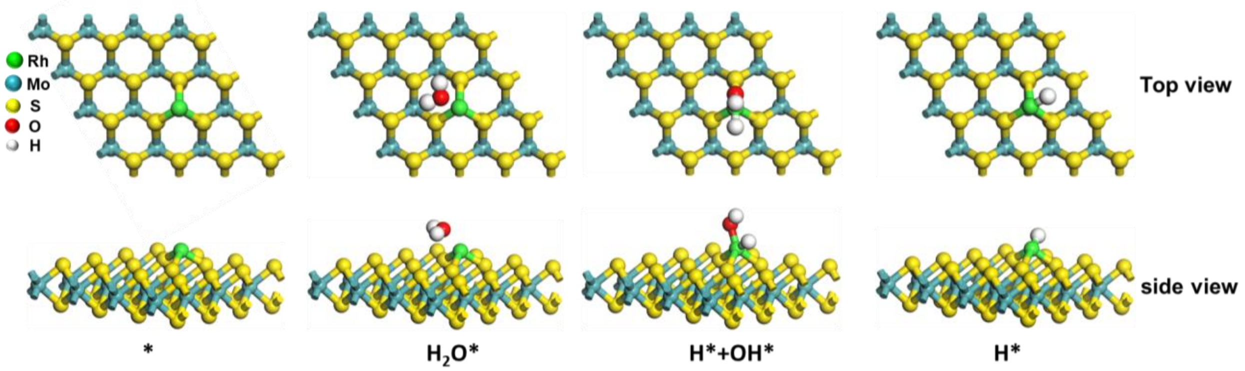

Supplementary Fig. 13. Diagram illustrating the HER at Rh sites on Rh_{SA}-MoS_2.

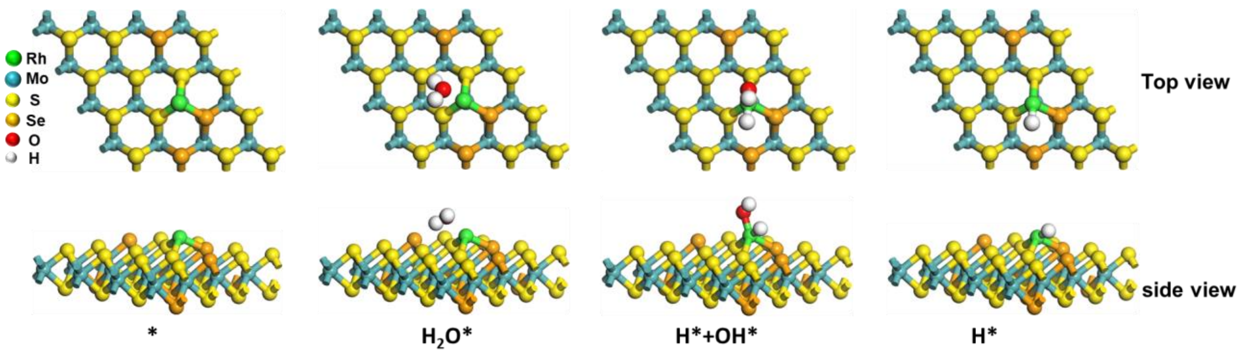

Supplementary Fig. 14. Diagram illustrating the HER at Rh sites on Rh_{SA}-$MoS_{1.5}Se_{0.5}$.

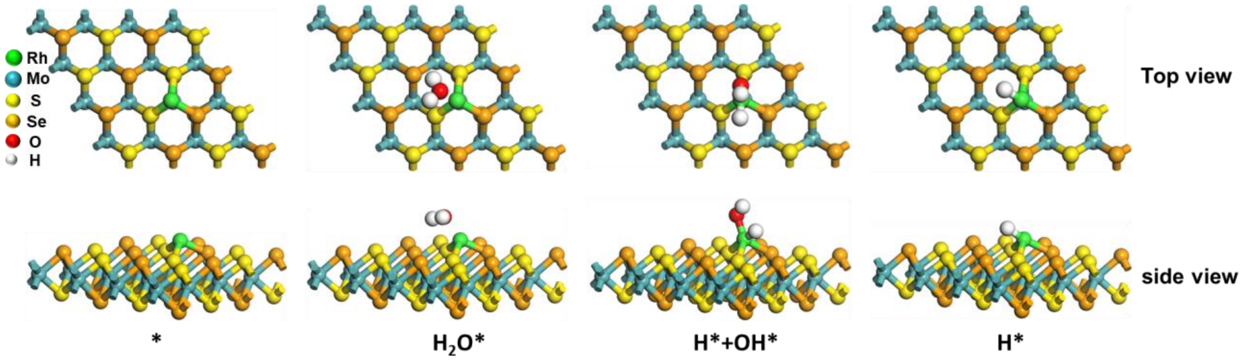

Supplementary Fig. 15. Diagram illustrating the HER at Rh sites on Rh_{SA}-MoSSe.

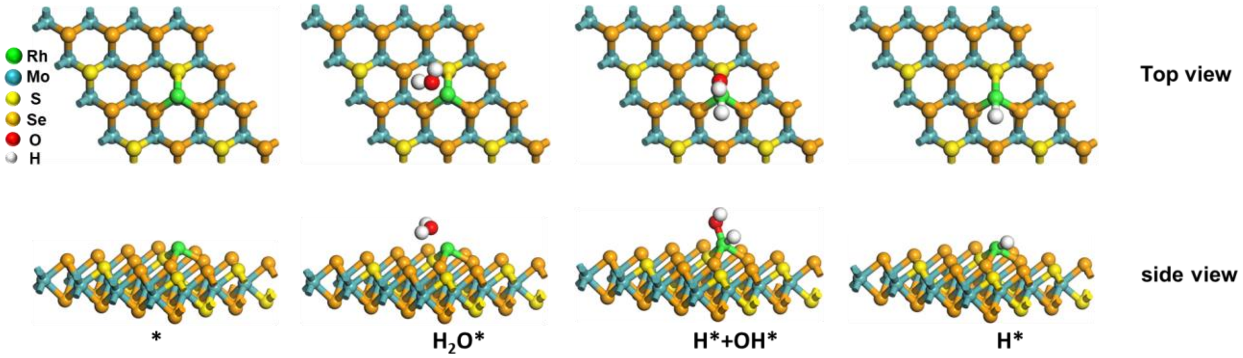

Supplementary Fig. 16. Diagram illustrating the HER at Rh sites on Rh_{SA}-$MoS_{0.5}Se_{1.5}$.

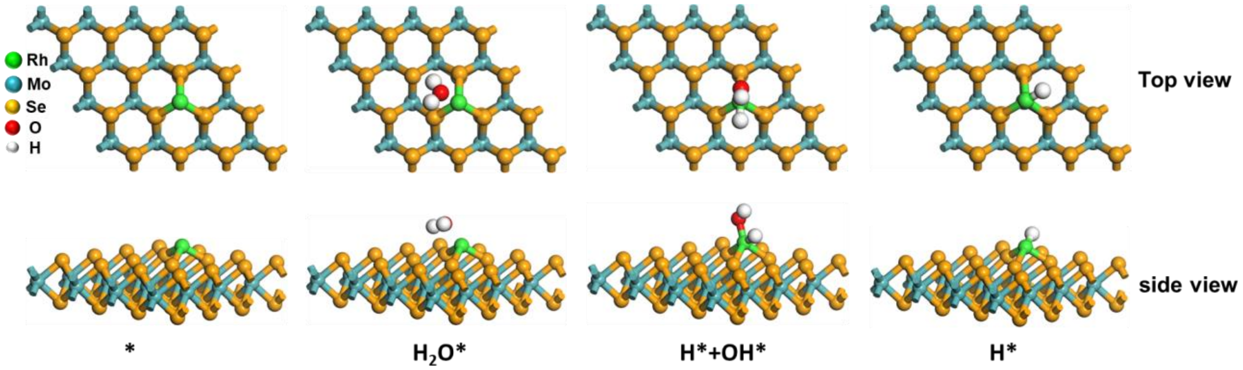

Supplementary Fig. 17. Diagram illustrating the HER at Rh sites on Rh_{SA}-$MoSe_2$.

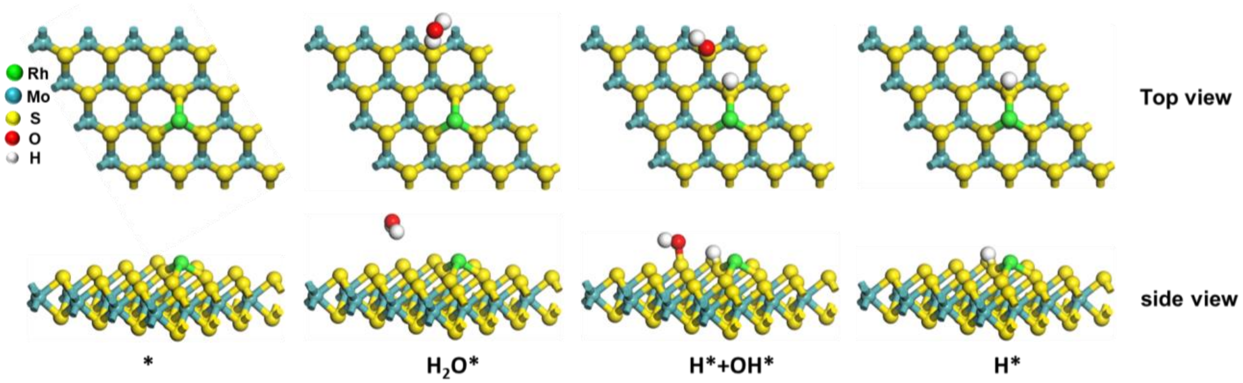

Supplementary Fig. 18. Diagram illustrating the HER at S sites surrounding Rh single-atom on Rh_{SA}-MoS_2.

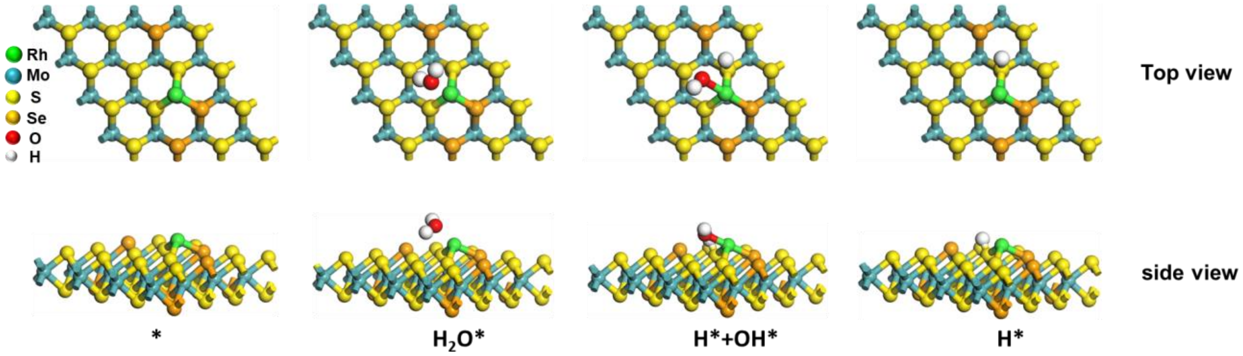

Supplementary Fig. 19. Diagram illustrating the HER at S/Se sites surrounding Rh single-atom on Rh_{SA}-$MoS_{1.5}Se_{0.5}$.

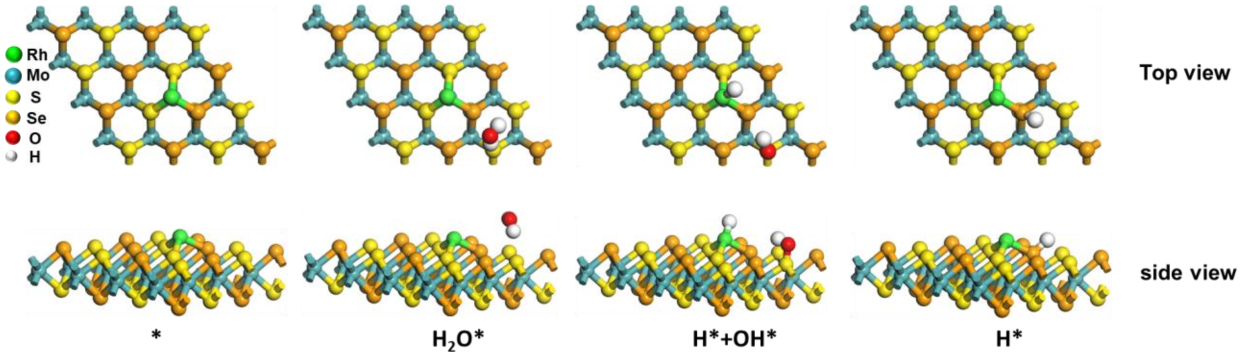

Supplementary Fig. 20. Diagram illustrating the HER at S/Se sites surrounding Rh single-atom on Rh_{SA}-MoSSe.

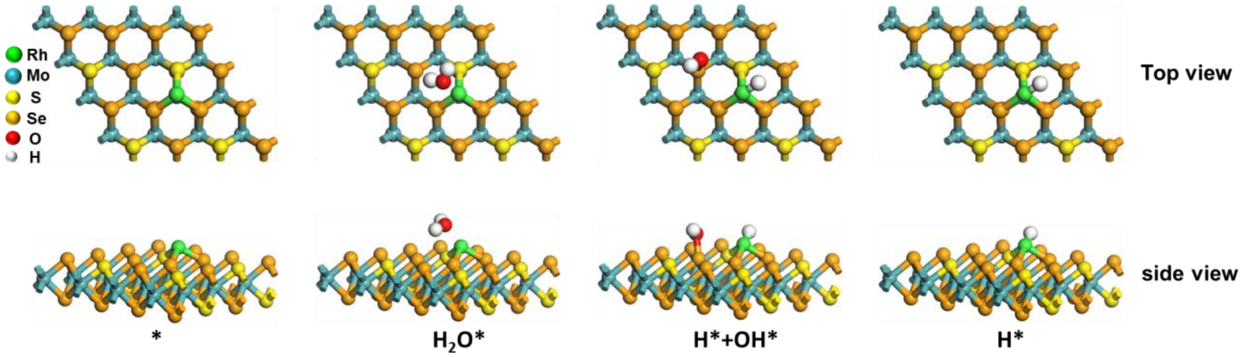

Supplementary Fig. 21. Diagram illustrating the HER at S/Se sites surrounding Rh single-atom on Rh_{SA}-$MoS_{0.5}Se_{1.5}$.

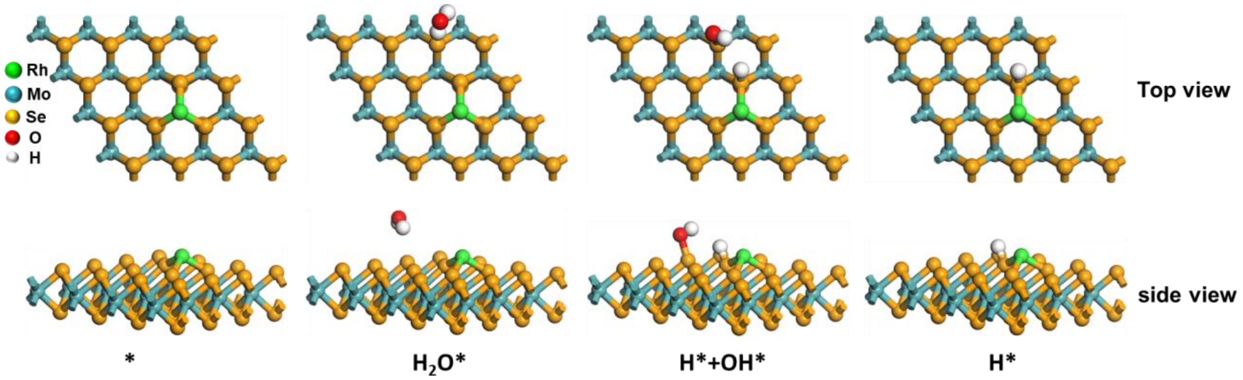

Supplementary Fig. 22. Diagram illustrating the HER at Se sites surrounding Rh single-atom on Rh_{SA}-$MoSe_2$.

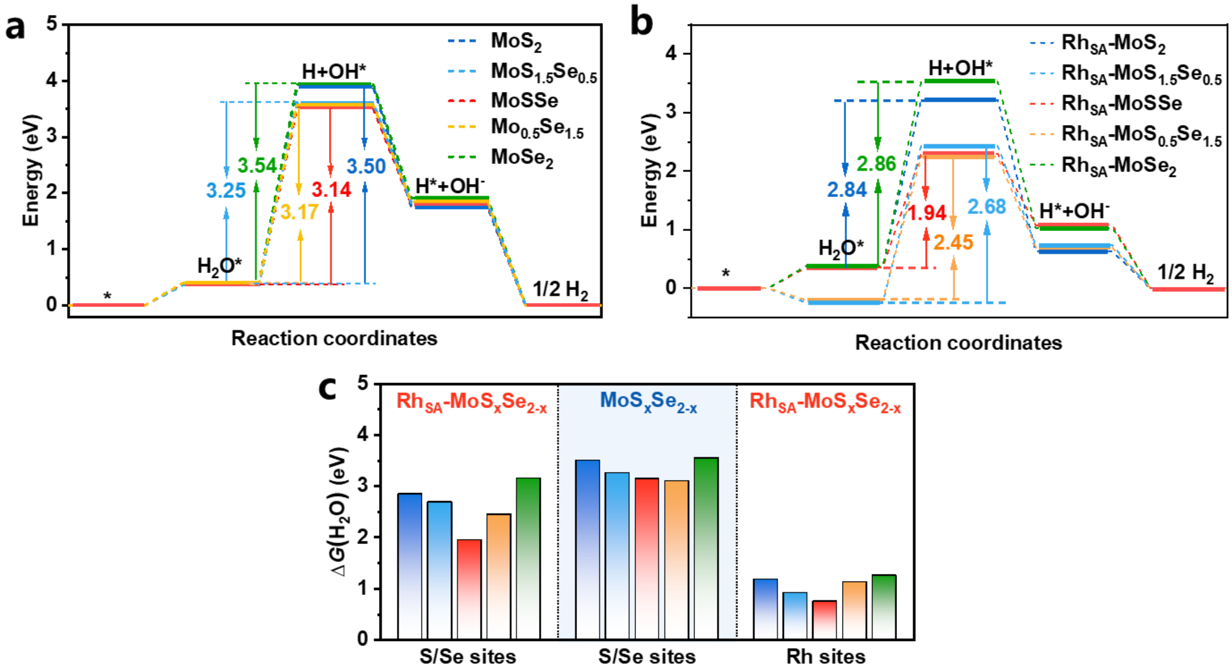

Supplementary Fig. 23. Gibbs free energy diagram for the HER process on (a) MoS_xSe_{2-x} at S/Se sites, (b) Rh_{SA}-MoS_xSe_{2-x} at S/Se sites surrounding Rh single-atom. (c) Water dissociation energies at S/Se sites in pristine MoS_xSe_{2-x}, Rh sites, and adjacent S/Se sites in Rh_{SA}-MoS_xSe_{2-x}.

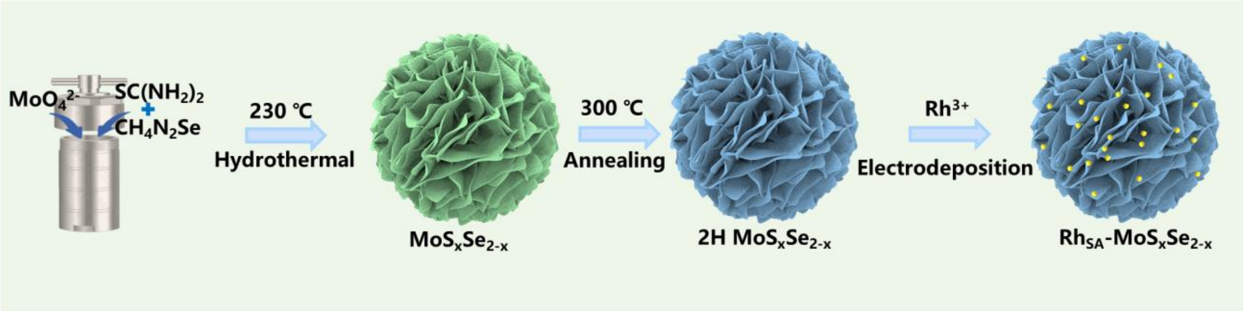

Supplementary Fig. 24. Synthetic pathway for Rh_{SA}-MoS_xSe_{2-x} catalysts.

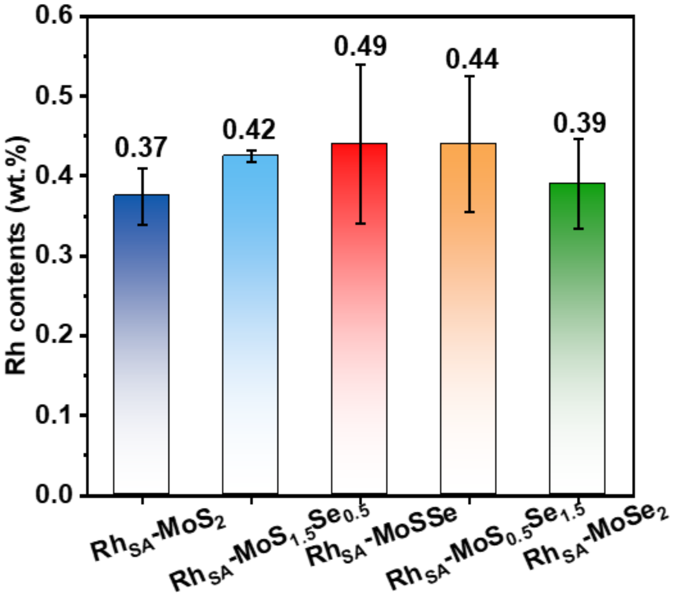

Supplementary Fig. 25. The mass loading of Rh species on MoS_xSe_{2-x} substrates. Error bars represent standard deviation (SD) from two independent measurements.

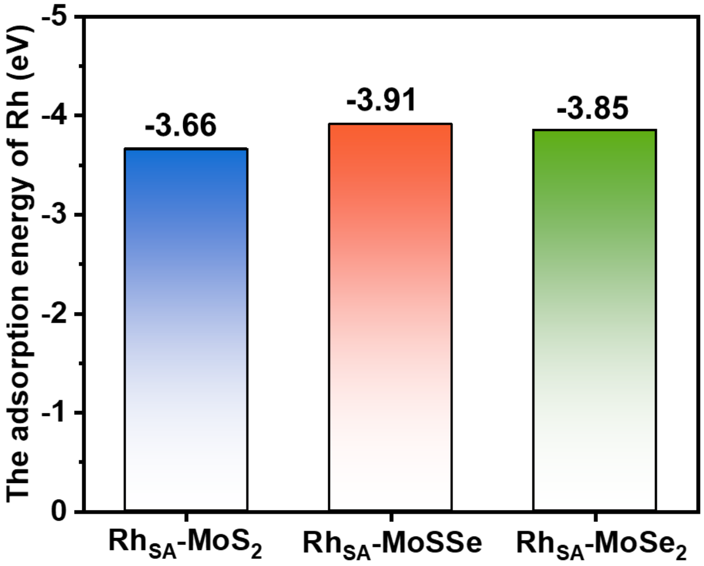

Supplementary Fig. 26. The adsorption energy of Rh on MoS_2, MoSSe, and $MoSe_2$.

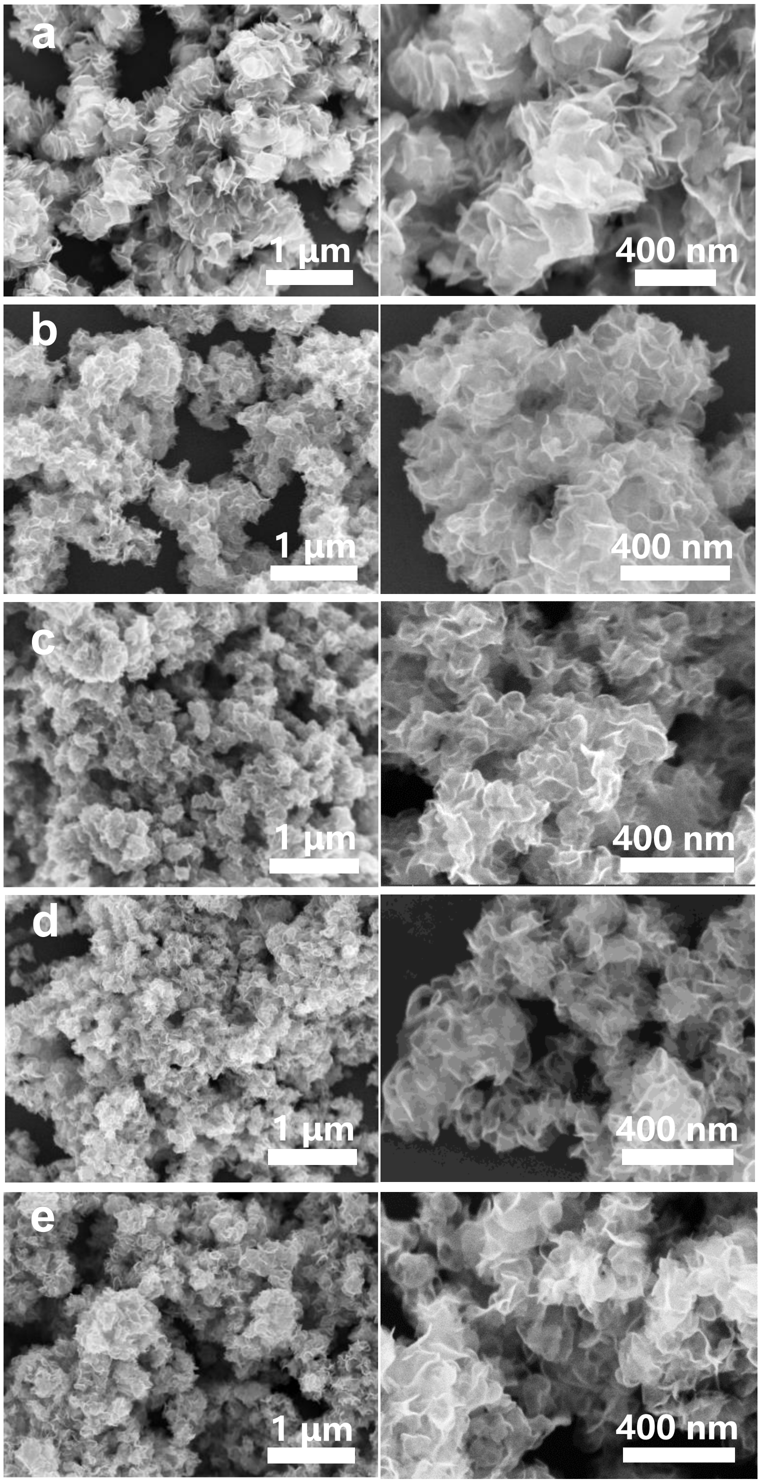

Supplementary Fig. 27. SEM images of (a) MoS_2, (b) $MoS_{1.5}Se_{0.5}$, (c) MoSSe, (d) $MoS_{0.5}Se_{1.5}$, and (e) $MoSe_2$.

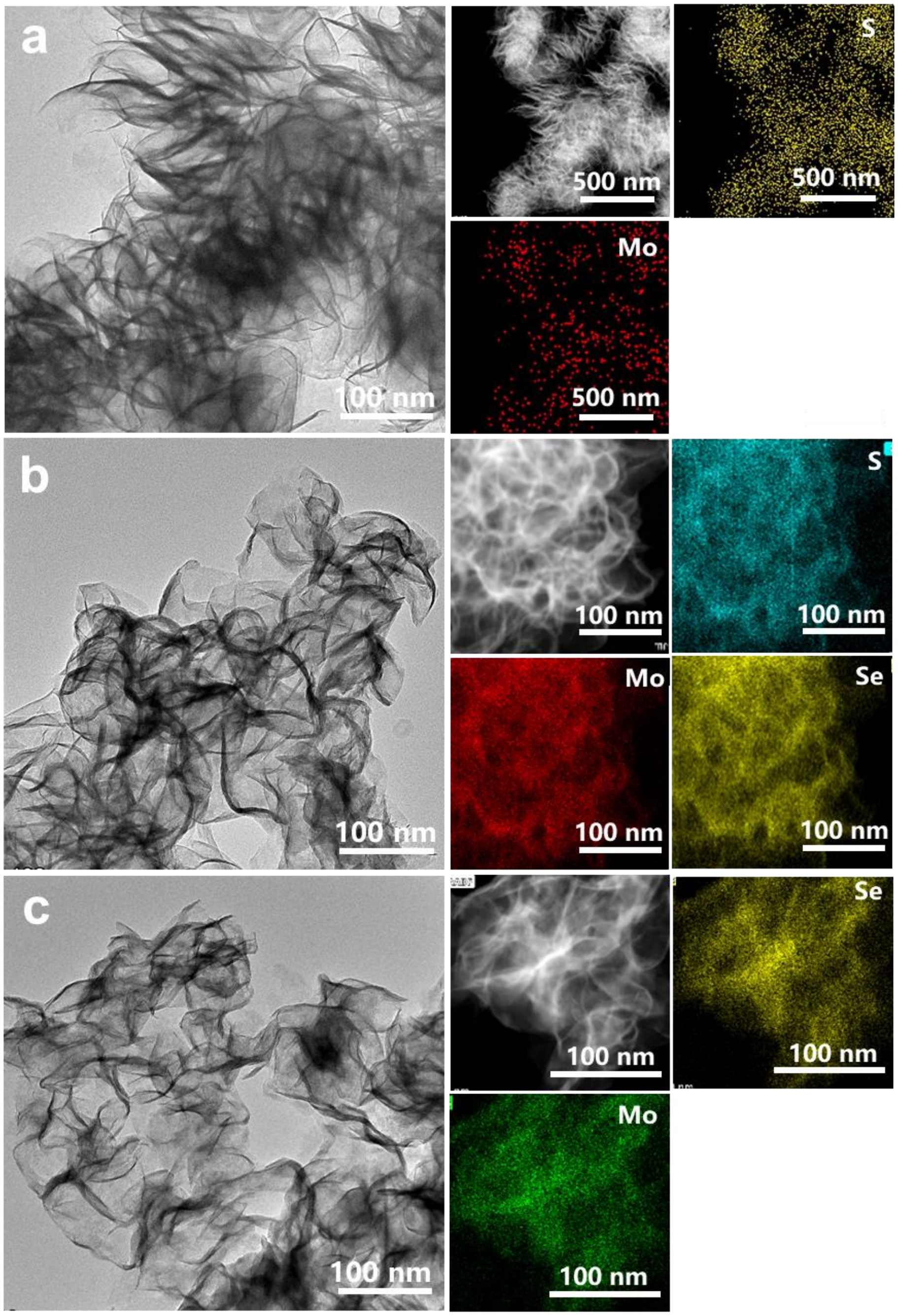

Supplementary Fig. 28. TEM images and corresponding EDS elemental mapping of (a) MoS_2, (b) MoSSe, and (c) $MoSe_2$.

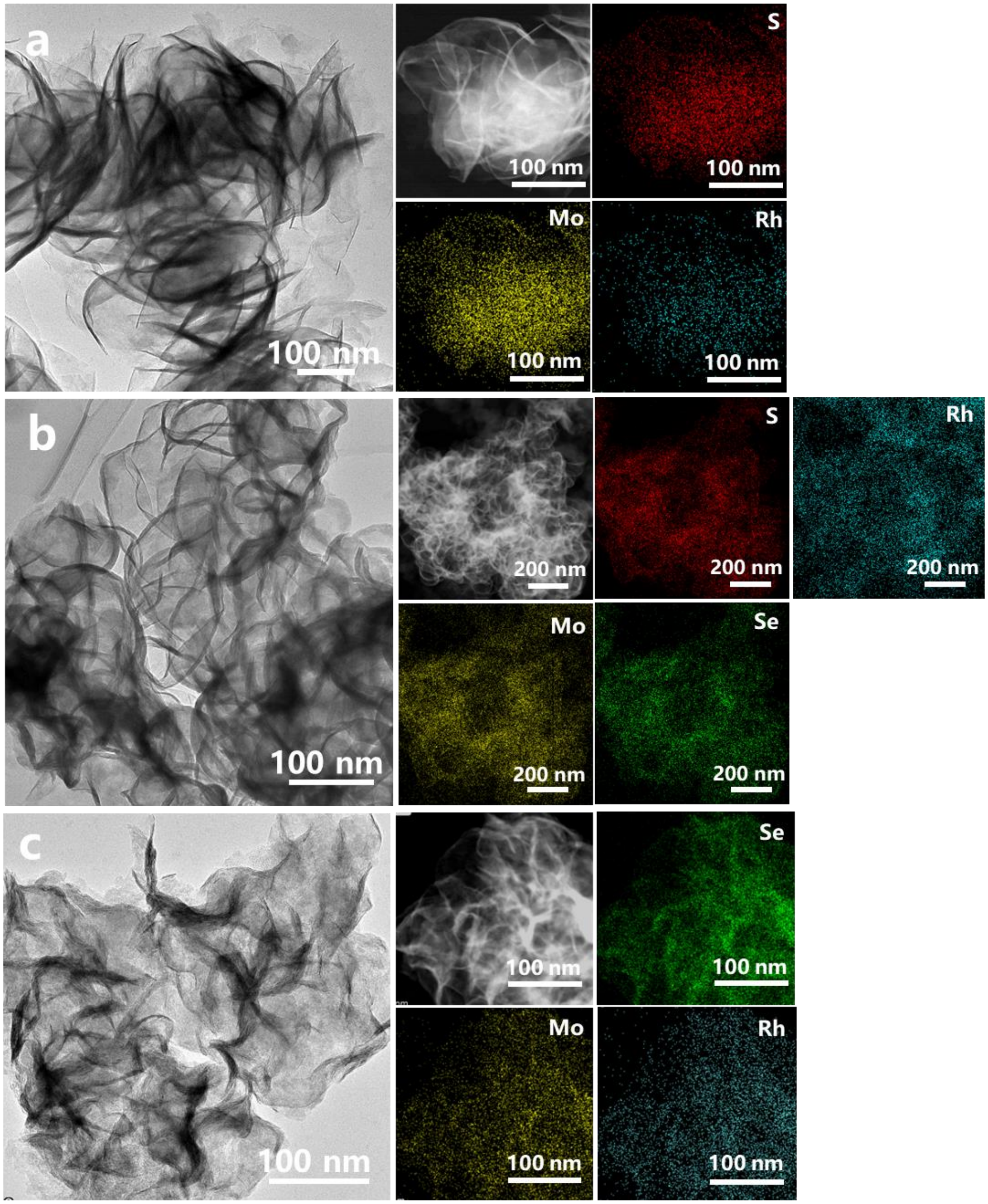

Supplementary Fig. 29. TEM images and corresponding EDS elemental mapping of (a) Rh_{SA}-MoS_2, (b) Rh_{SA}-MoSSe, and (c) Rh_{SA}-$MoSe_2$.

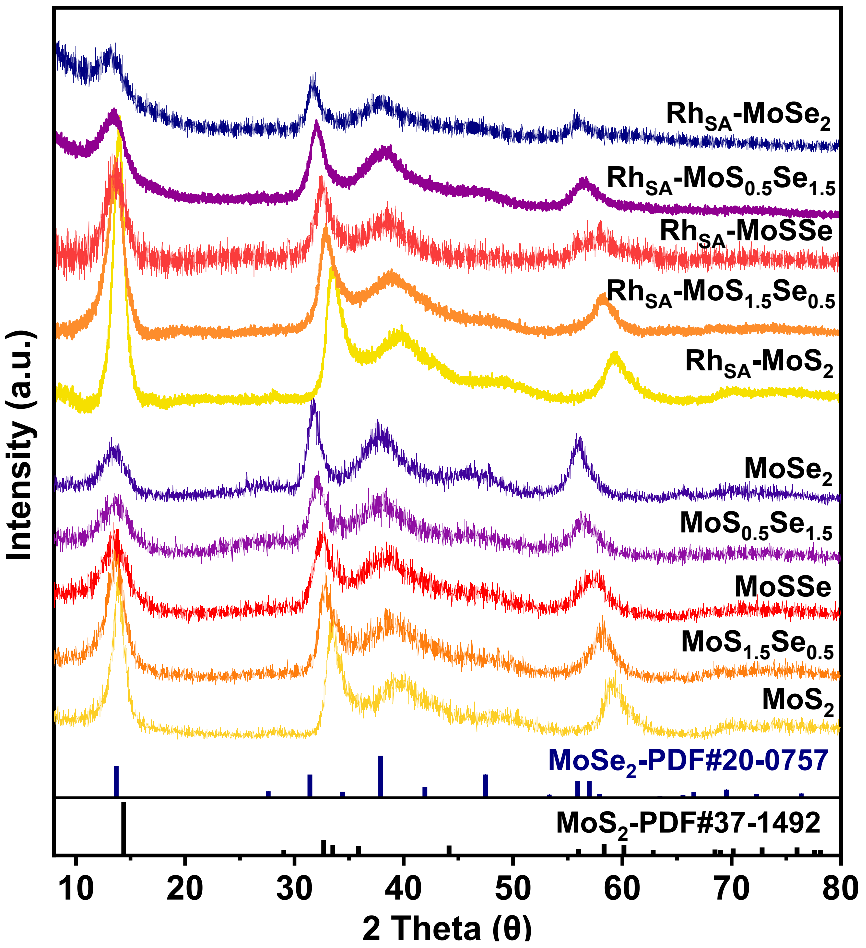

Supplementary Fig. 30. XRD patterns of MoS_2, $MoS_{1.5}Se_{0.5}$, MoSSe, $MoS_{0.5}Se_{1.5}$, $MoSe_2$, Rh_{SA}-MoS_2, Rh_{SA}-$MoS_{1.5}Se_{0.5}$, Rh_{SA}-MoSSe, Rh_{SA}-$MoS_{0.5}Se_{1.5}$, and Rh_{SA}-$MoSe_2$.

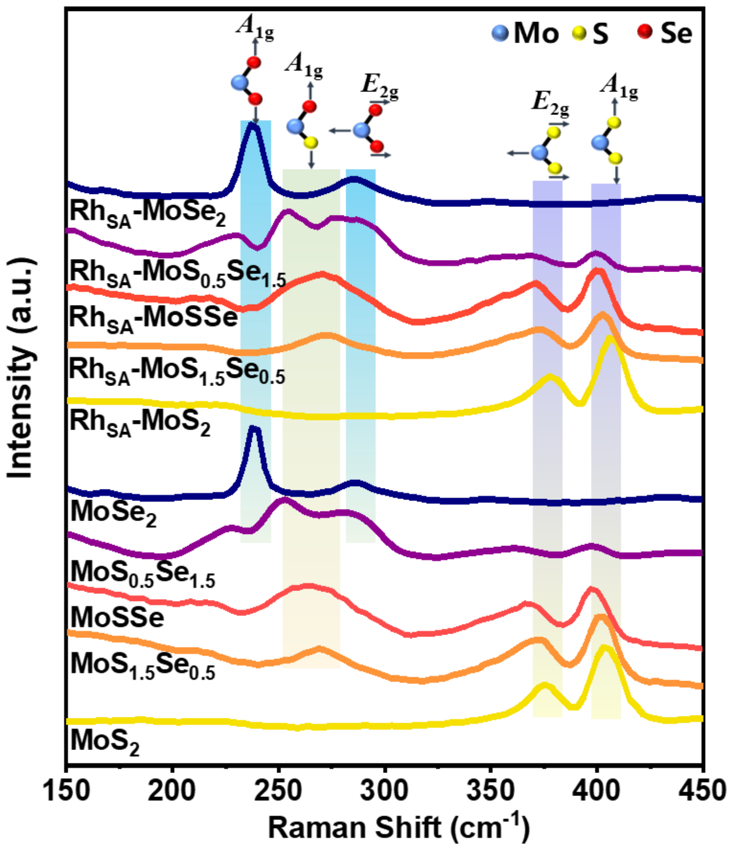

Supplementary Fig. 31. Raman spectra of MoS_2, $MoS_{1.5}Se_{0.5}$, MoSSe, $MoS_{0.5}Se_{1.5}$, $MoSe_2$, Rh_{SA}-MoS_2, Rh_{SA}-$MoS_{1.5}Se_{0.5}$, Rh_{SA}-MoSSe, Rh_{SA}-$MoS_{0.5}Se_{1.5}$, and Rh_{SA}-$MoSe_2$.

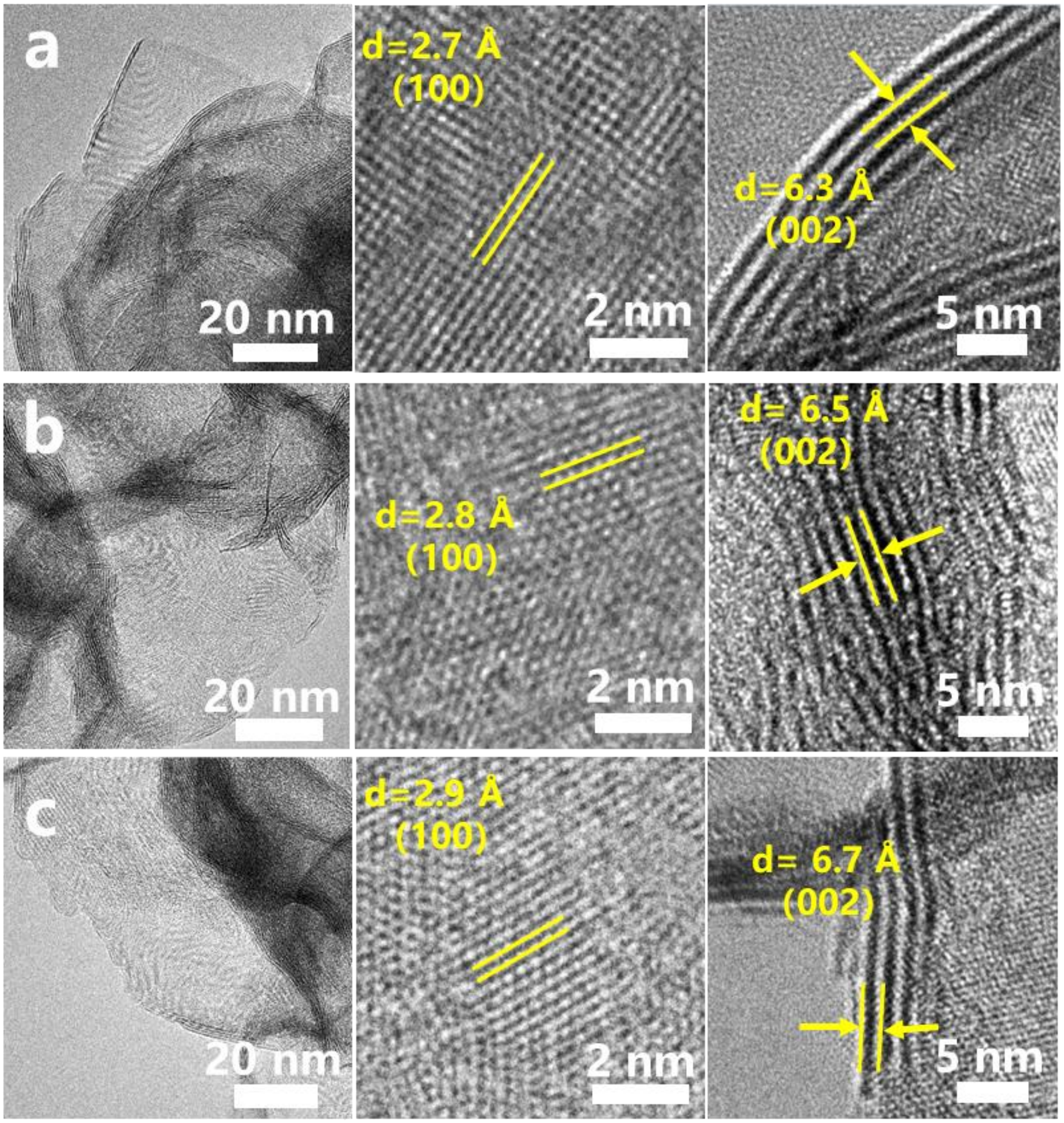

Supplementary Fig. 32. High-resolution transmission electron microscopy (HRTEM) images of (a) MoS_2, (b) MoSSe, and (c) $MoSe_2$.

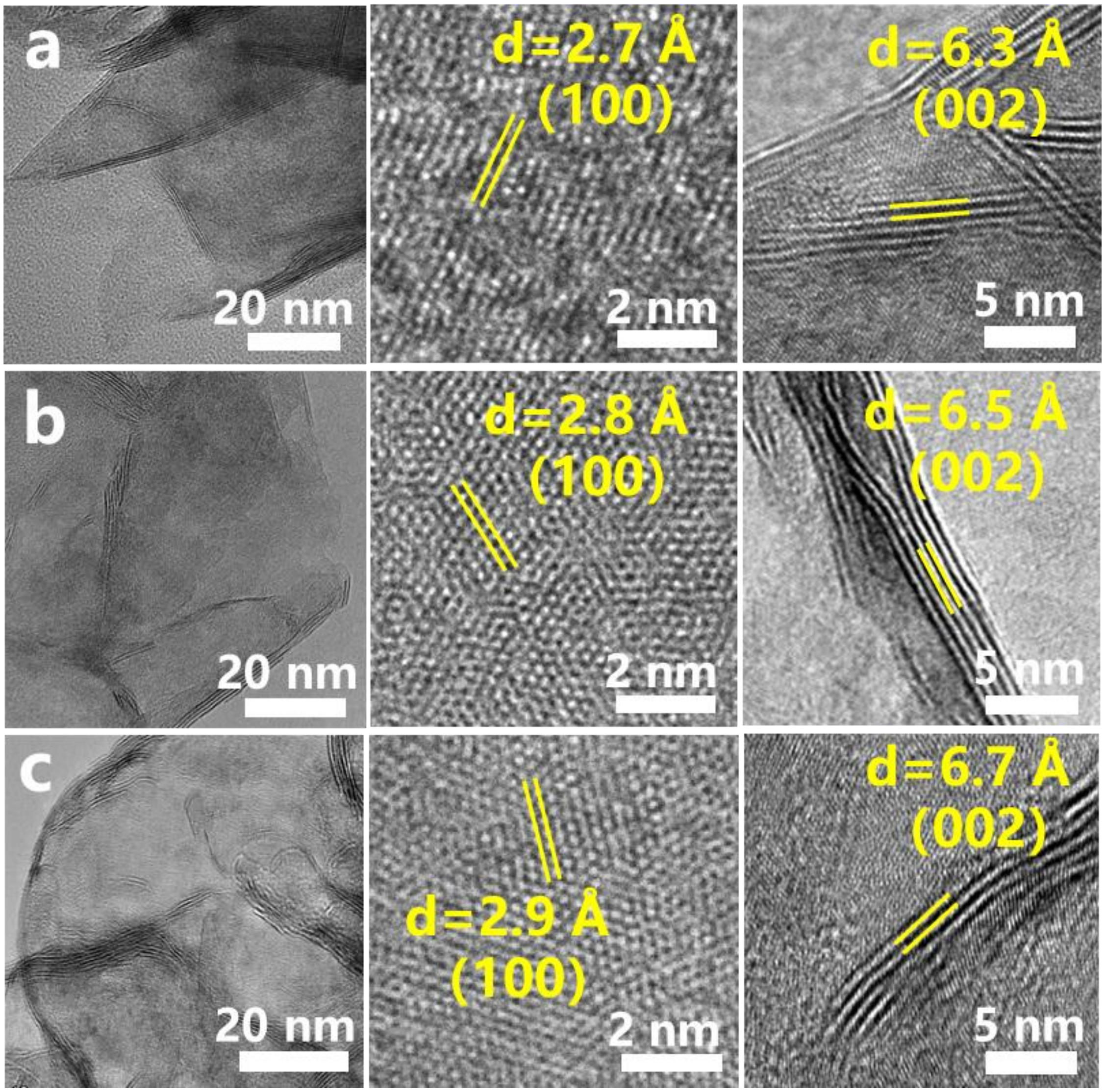

Supplementary Fig. 33. High-resolution transmission electron microscopy (HRTEM) images of (a) Rh_{SA}-MoS_2, (b) Rh_{SA}-MoSSe, and (c) Rh_{SA}-$MoSe_2$.

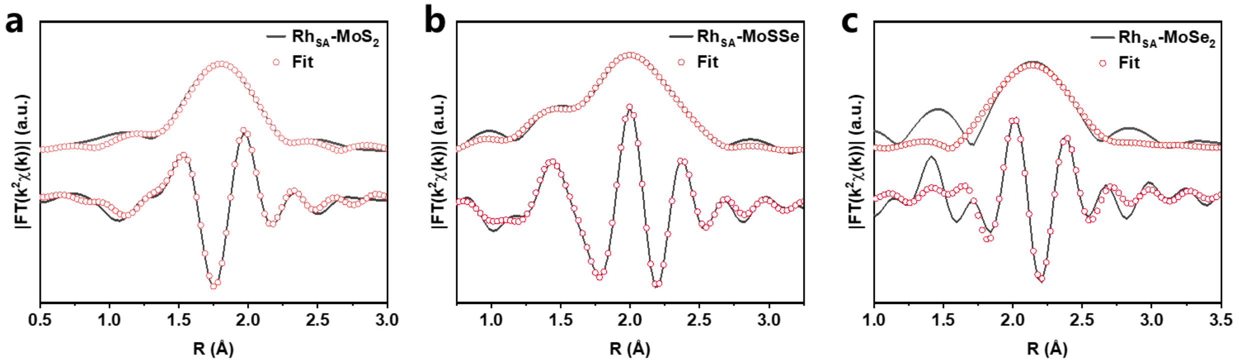

Supplementary Fig. 34. The Rh K-edge EXAFS fitting (red dots) for the Rh$_{SA}$-MoS$_2$ (a), Rh$_{SA}$-MoSSe (b), and Rh$_{SA}$-MoSe$_2$ (c), shown in k^2 weighted R-space.

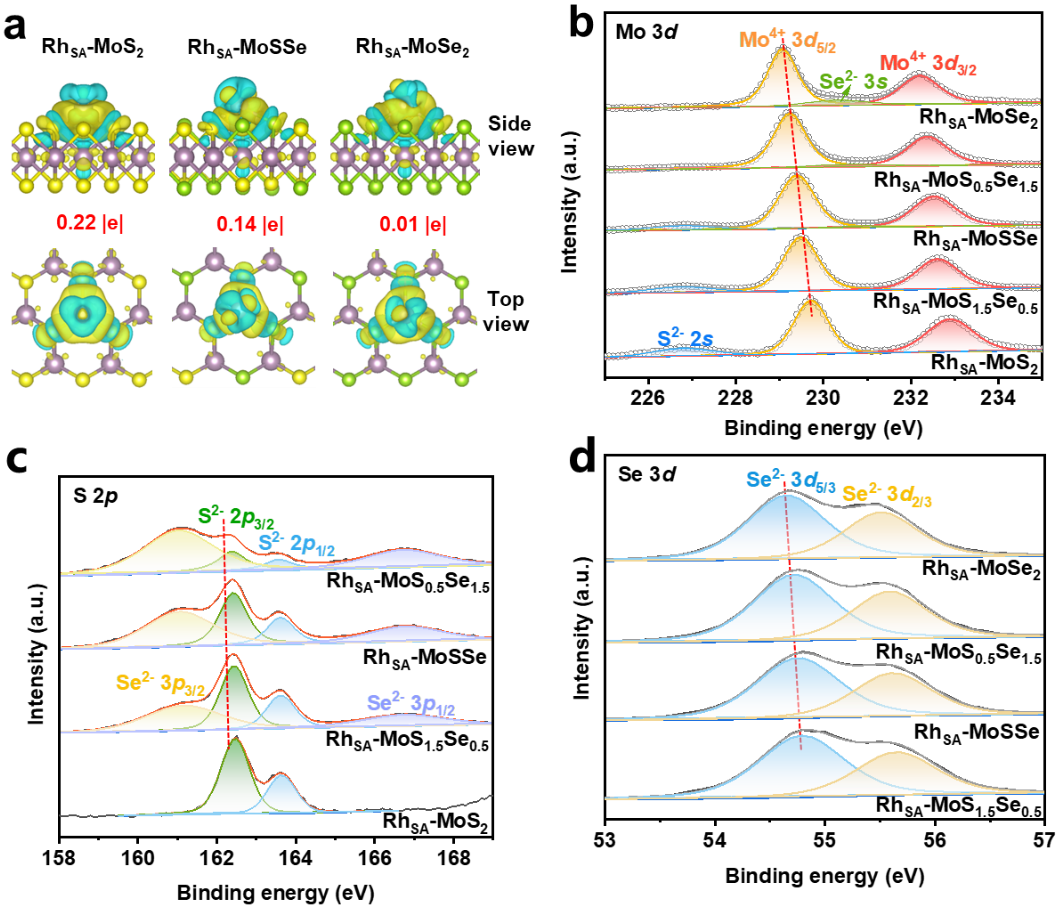

Supplementary Fig. 35. (a) The charge density difference of Rh atoms in Rh_{SA}-MoS_2, Rh_{SA}-MoSSe, and Rh_{SA}-$MoSe_2$, with yellow and blue regions indicating charge accumulation and decrease, respectively. (b) Mo 3*d*, (c) S 2*p*, and (d) Se 3*d* XPS spectra of Rh_{SA}-MoS_2, Rh_{SA}-$MoS_{1.5}Se_{0.5}$, Rh_{SA}-MoSSe, Rh_{SA}-$MoS_{0.5}Se_{1.5}$, and Rh_{SA}-$MoSe_2$.

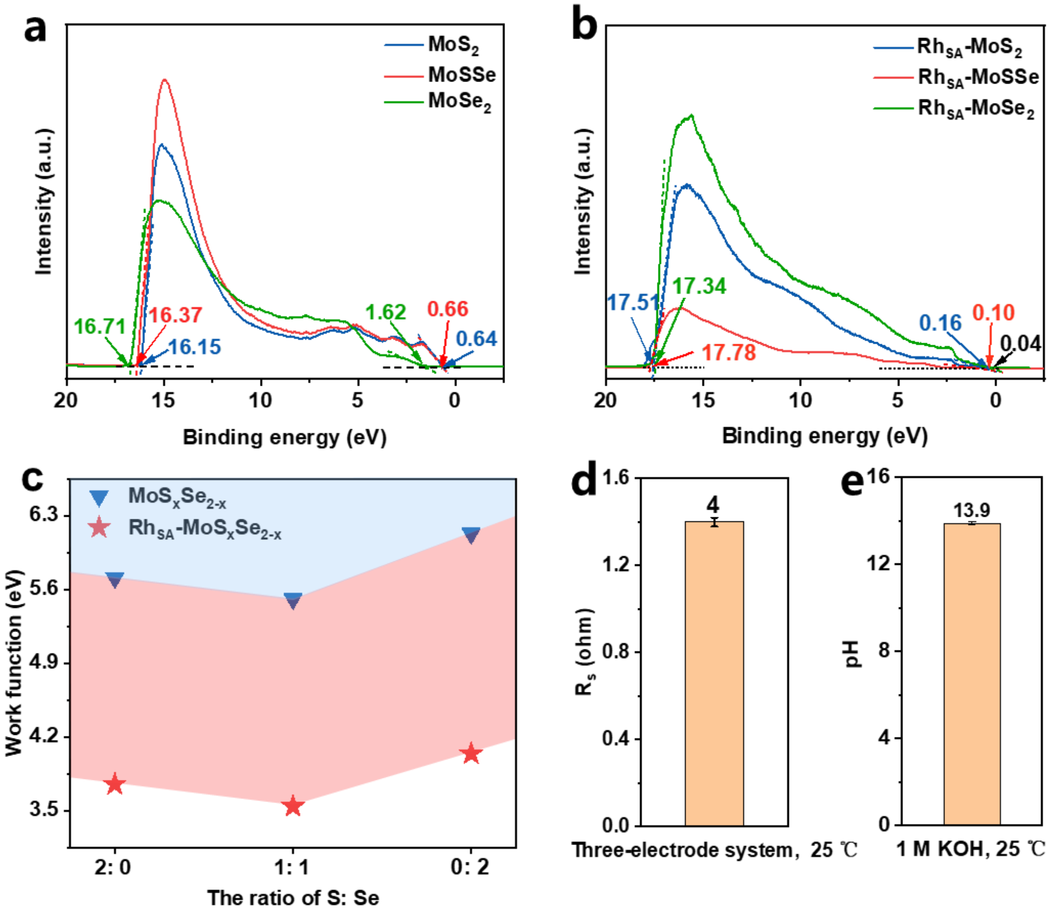

Supplementary Fig. 36. (a) UPS spectra of MoS_2, MoSSe, and $MoSe_2$. (b) UPS spectra of Rh_{SA}-MoS_2, Rh_{SA}-MoSSe, and Rh_{SA}-$MoSe_2$. (c) Dependence of the work function on the S: Se ratio for Rh_{SA}-MoS_xSe_{2-x} and pristine MoS_xSe_{2-x}. (d) The solution resistance (R_s) in a three-electrode system at 25 °C: 4 ± 0.8 ohm, error bars represent SD from two independent measurements. (e) The pH value of 1 M KOH solution at 25 °C: 13.9 ± 0.07, error bars represent SD from two independent test.

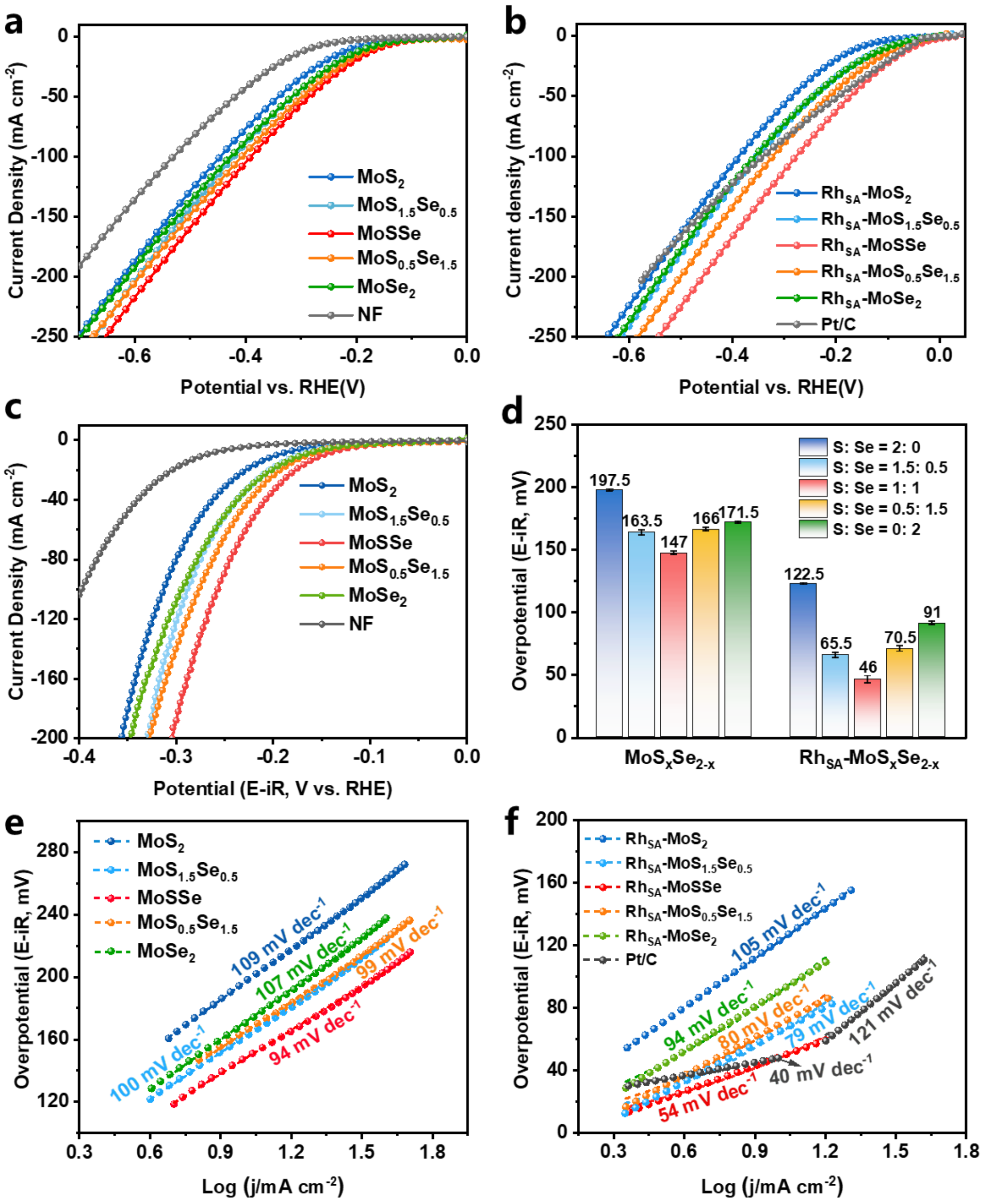

Supplementary Fig. 37. HER polarization curves of (a) MoS_xSe_{2-x} and (b) Rh_{SA}-MoS_xSe_{2-x} without *iR* compensation. (c) HER polarization curves and (e) corresponding Tafel slopes for MoS_xSe_{2-x} and NF catalysts, with 95% *iR* compensation. (d) Overpotentials at current densities of 10 mA cm^{-2} for MoS_xSe_{2-x} and Rh_{SA}-MoS_xSe_{2-x}, error bars represent SD from two independent measurements. (f) Tafel slopes for Rh_{SA}-MoS_xSe_{2-x} and Pt/C catalysts.

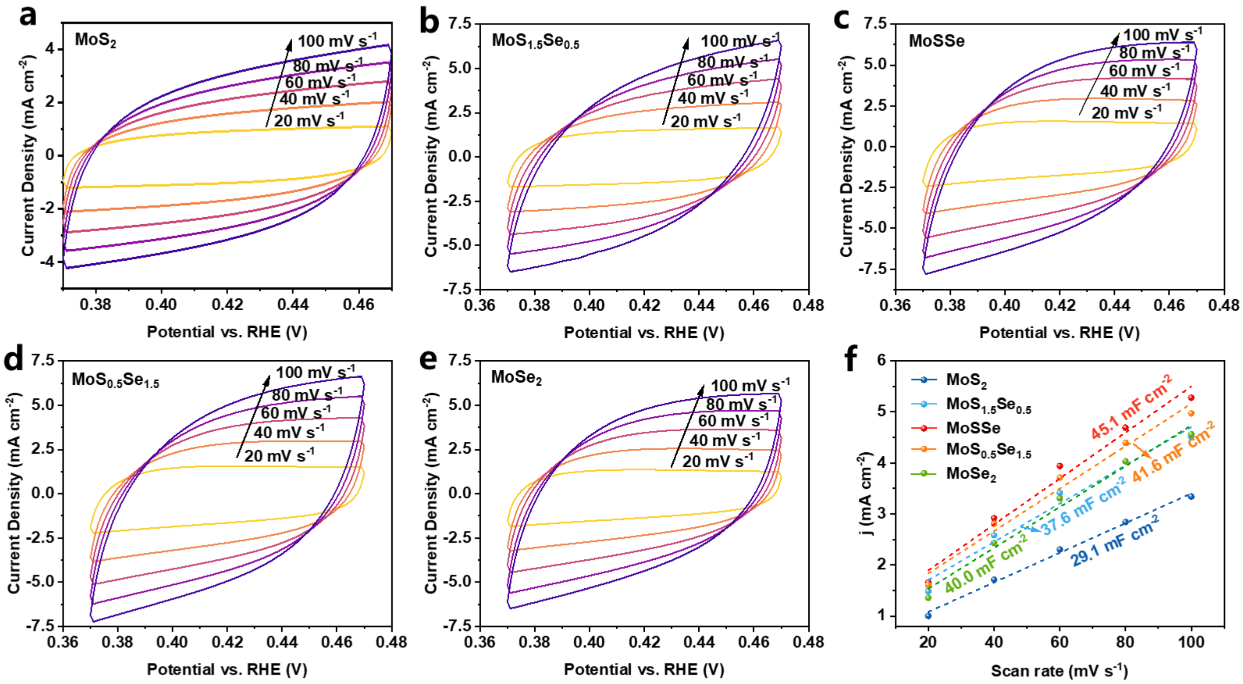

Supplementary Fig. 38. Double-layer capacitance analysis. CVs of (a) MoS_2, (b) $MoS_{1.5}Se_{0.5}$, (c) MoSSe, (d) $MoS_{0.5}Se_{1.5}$, and (e) $MoSe_2$, recorded at scan rates of 20, 40, 60, 80, and 100 mV s^{-1}. (f) Plots of current densities versus scan rate, where Δj represents the difference between anodic and cathodic current densities at 0.42 V vs. RHE. The slopes of the linear fits correspond to twice the electrochemical double-layer capacitance.

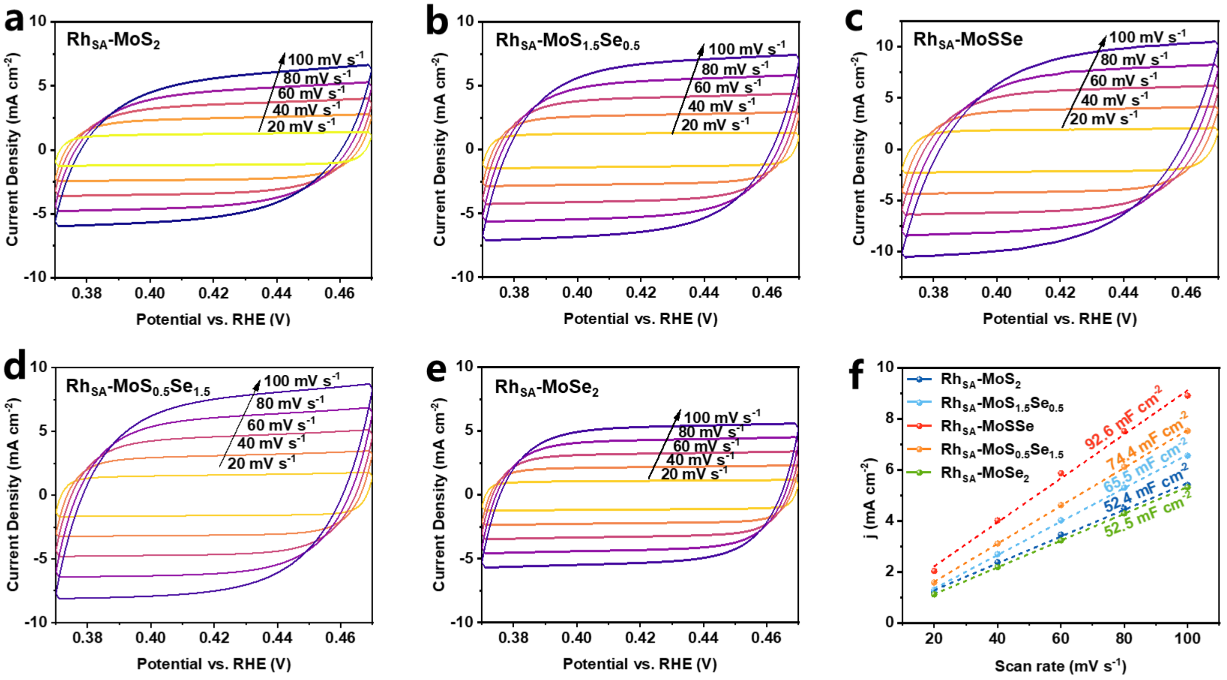

Supplementary Fig. 39. Double-layer capacitance measurements. CVs of (a) Rh$_{SA}$-MoS$_2$, (b) Rh$_{SA}$-MoS$_{1.5}$Se$_{0.5}$, (c) Rh$_{SA}$-MoSSe, (d) Rh$_{SA}$-MoS$_{0.5}$Se$_{1.5}$, and (e) Rh$_{SA}$-MoSe$_2$, recorded at scan rates of 20, 40, 60, 80, and 100 mV s^{-1}. (f) Plots of current density versus scan rate, where Δj represents the difference between anodic and cathodic current densities at 0.42 V vs. RHE. The slopes of the linear fits correspond to twice the electrochemical double-layer capacitance.

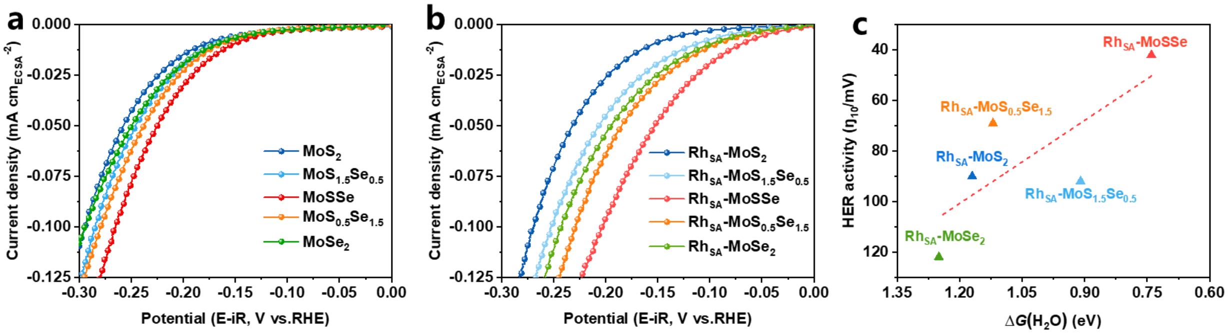

Supplementary Fig. 40. HER activities of (a) MoS_xSe_{2-x} and (b) Rh_{SA}-MoS_xSe_{2-x} catalysts, normalized to ECSA. (c) Relationship of $\Delta G(H_2O)$ and alkaline HER activity of Rh_{SA}-MoS_xSe_{2-x}.

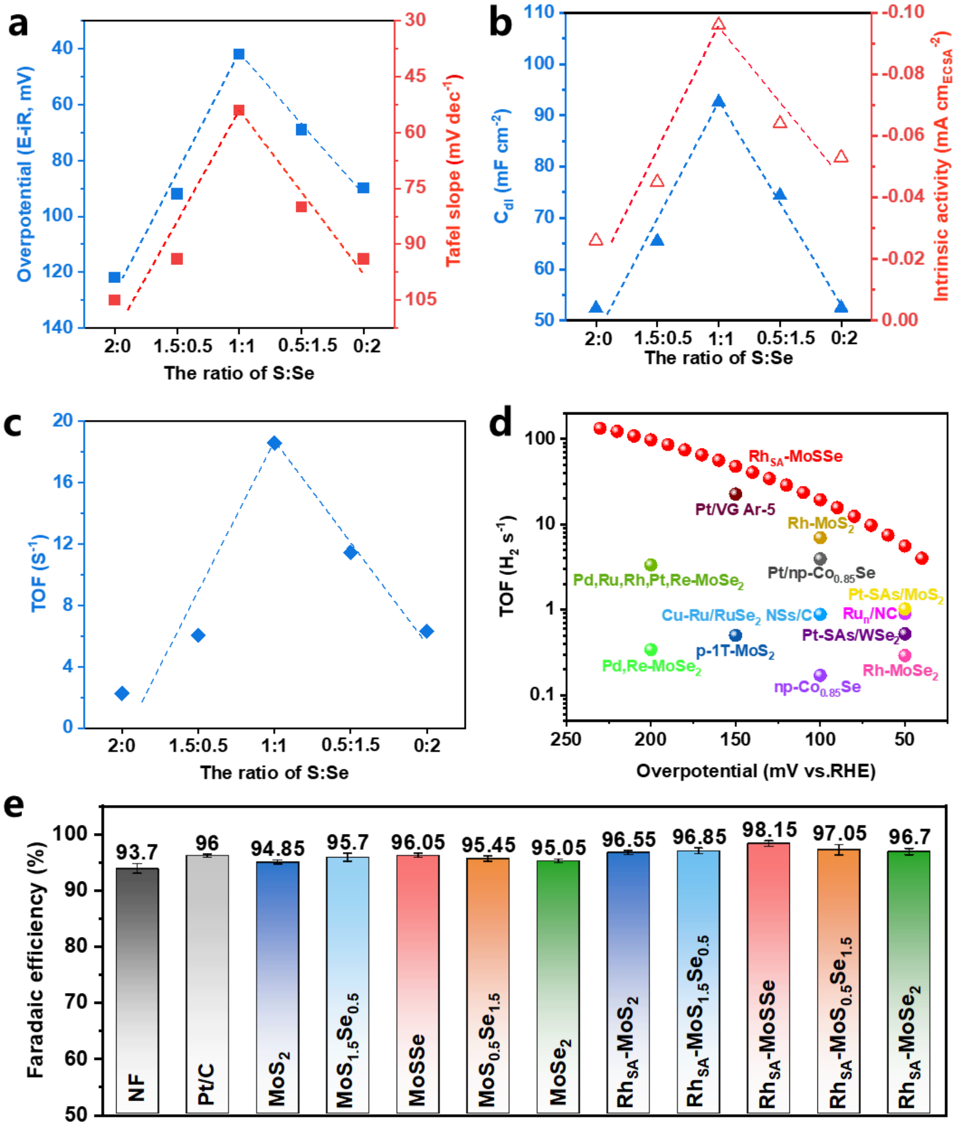

Supplementary Fig. 41. (a) Correlation between the S: Se ratio in Rh$_{SA}$-MoS$_x$Se$_{2-x}$ and overpotential at 10 mA cm^{-2} and Tafel slope. (b) Correlation between the S: Se ratio in Rh$_{SA}$-MoS$_x$Se$_{2-x}$ and C_{dl} and intrinsic activity. (c) Correlation between the S: Se ratio in Rh$_{SA}$-MoS$_x$Se$_{2-x}$ and overpotential at 10 mA cm^{-2}. (d) TOF values of Rh$_{SA}$-MoSSe (red dot) compared with previously reported HER electrocatalysts at -100 mV vs. RHE, the TOF values was only performed once. (e) Faradaic efficiency of MoS$_x$Se$_{2-x}$, Rh$_{SA}$-MoS$_x$Se$_{2-x}$, NF, and Pt/C samples at -0.2 V. Error bars represent SD from two independent measurements.

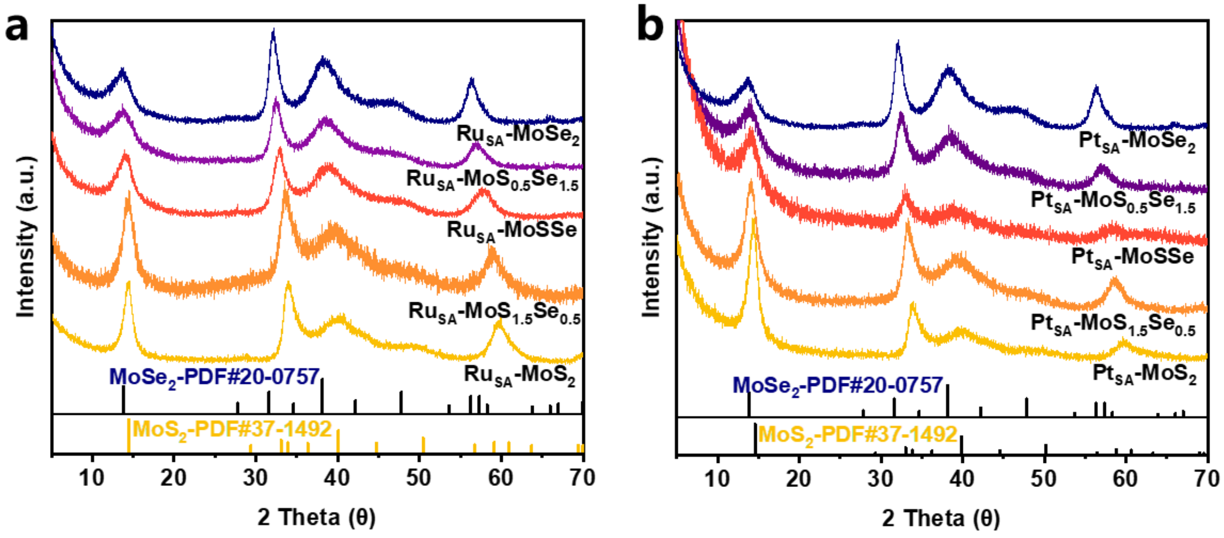

Supplementary Fig. 42. XRD patterns of (a) Ru$_{SA}$-MoS$_x$Se$_{2-x}$ catalysts, and (b) Pt$_{SA}$-MoS$_x$Se$_{2-x}$ catalysts.

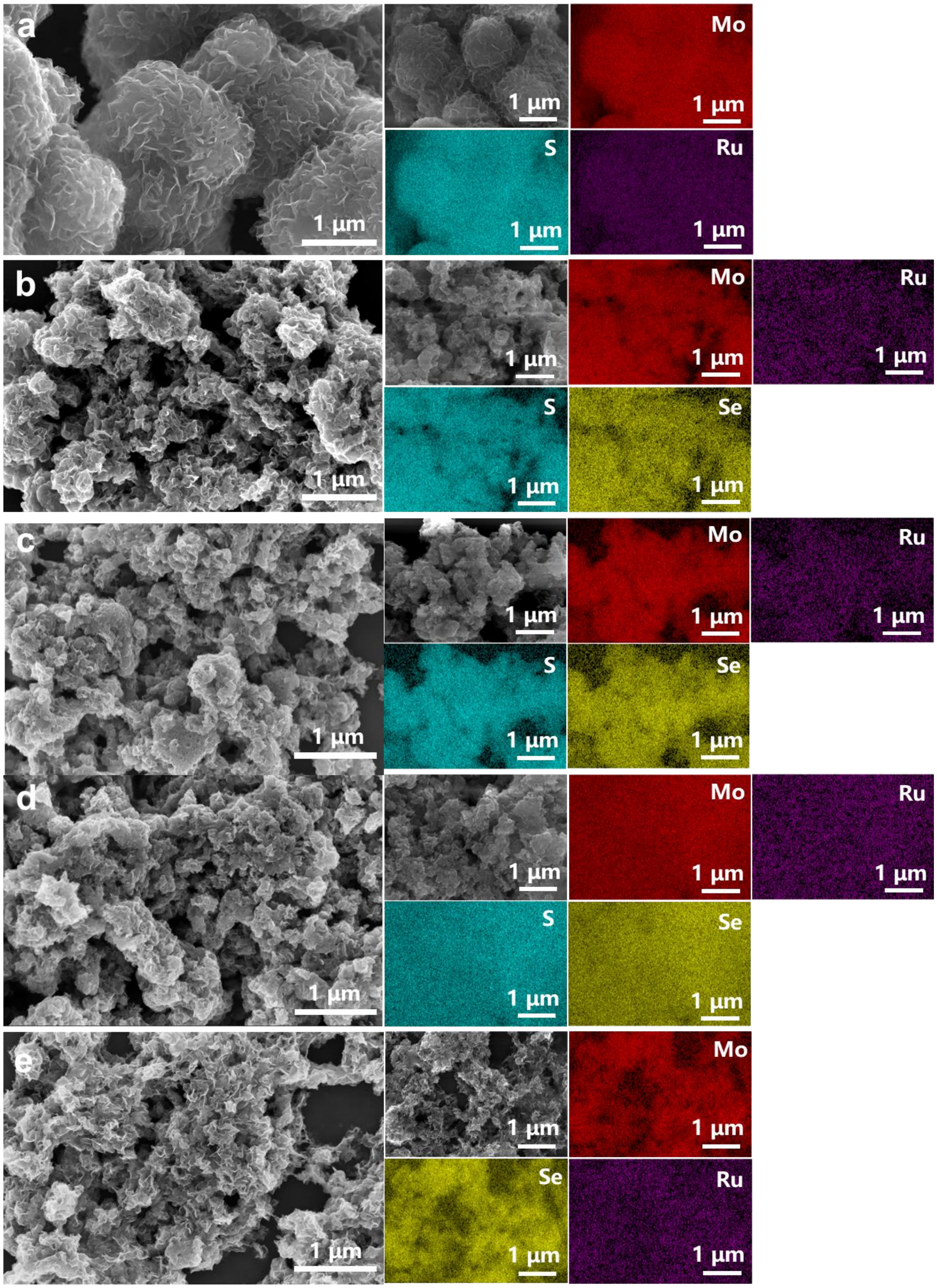

Supplementary Fig. 43. SEM images and corresponding EDS elemental mapping of (a) Ru_{SA}-MoS_2, (b) Ru_{SA}-$MoS_{1.5}Se_{0.5}$, (c) Ru_{SA}-MoSSe, (d) Ru_{SA}-$MoS_{1.5}Se_{0.5}$, and (e) Ru_{SA}-$MoSe_2$.

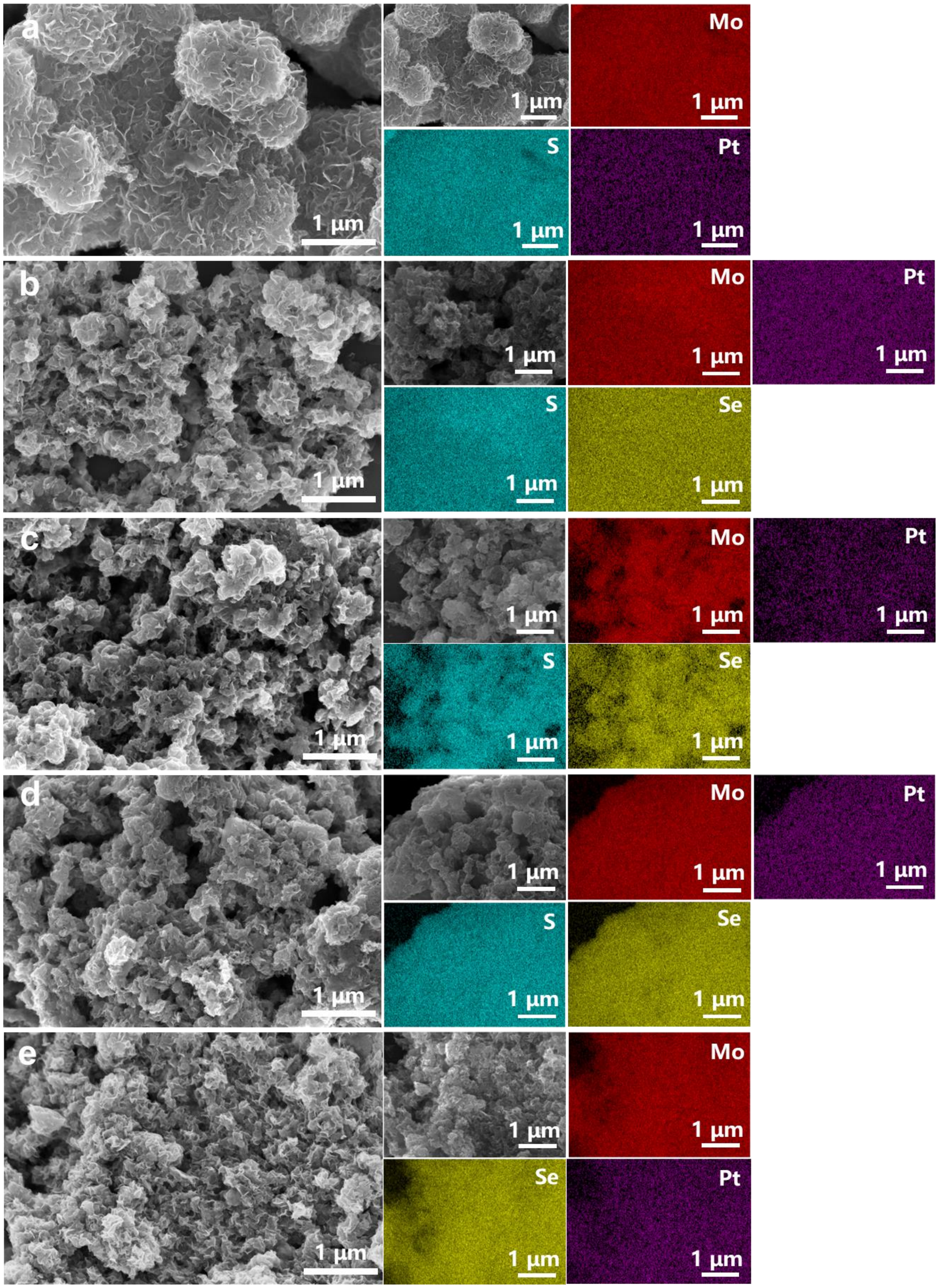

Supplementary Fig. 44. SEM images and corresponding EDS elemental mapping of (a) Pt_{SA}-MoS_2, (b) Pt_{SA}-$MoS_{1.5}Se_{0.5}$, (c) Pt_{SA}-MoSSe, (d) Pt_{SA}-$MoS_{1.5}Se_{0.5}$, and (e) Pt_{SA}-$MoSe_2$.

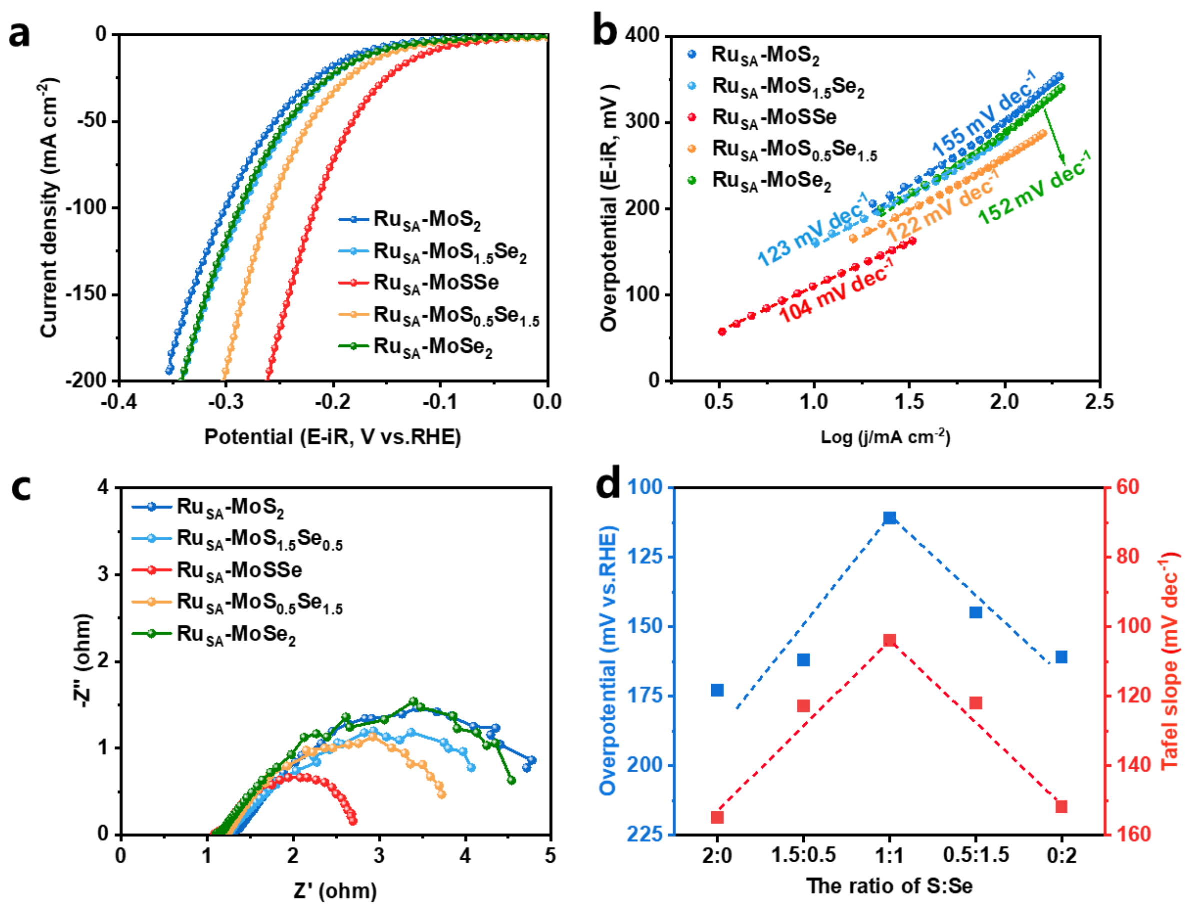

Supplementary Fig. 45. (a) HER polarization curves, (b) Tafel slopes, and (c) Nyquist plots of Ru_{SA}-MoS_xSe_{2-x} at -0.1 V vs. RHE. (d) Correlation between the S: Se ratio in Ru_{SA}-MoS_xSe_{2-x} and overpotential at 10 mA cm^{-2} and Tafel slope.

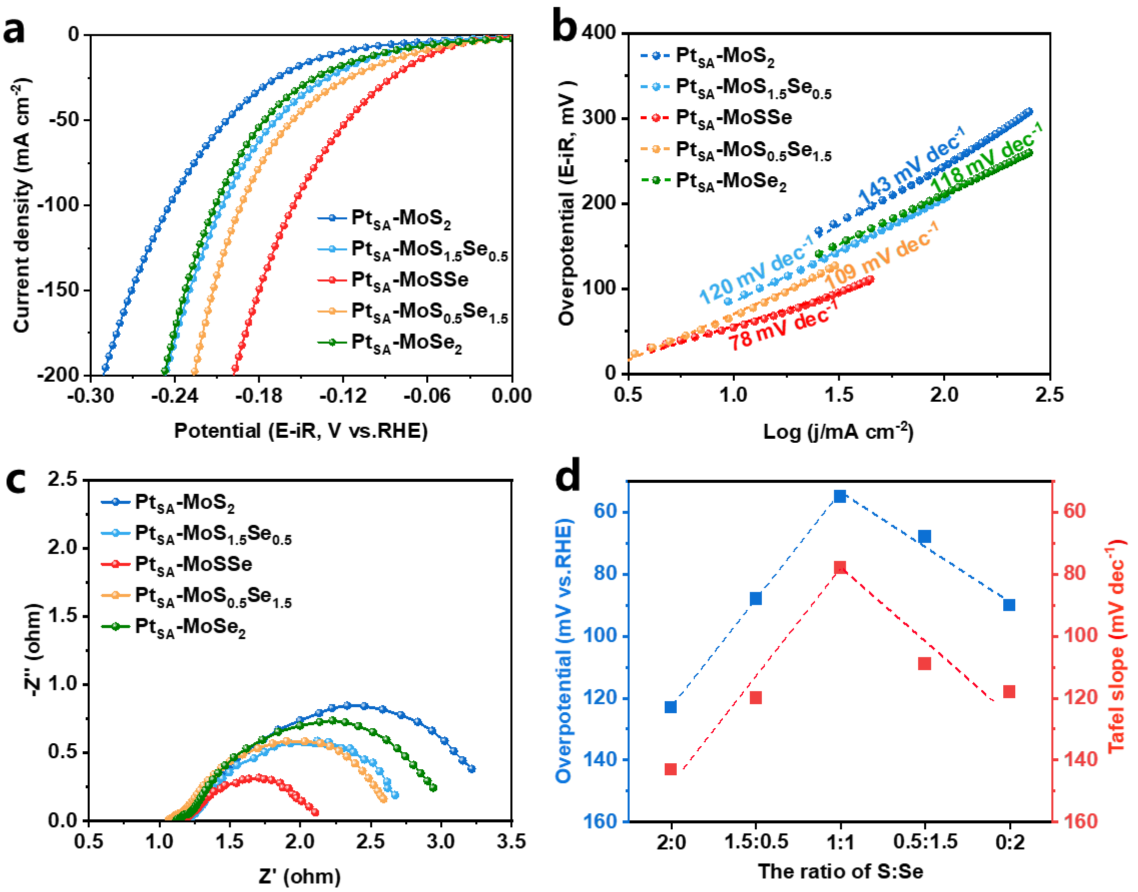

Supplementary Fig. 46. (a) HER polarization curves, and corresponding (b) Tafel slopes of Pt_{SA}-MoS_xSe_{2-x}, with (c) Nyquist plots of Pt_{SA}-MoS_xSe_{2-x} at -0.1 V vs. RHE. (d) (a) Correlation between the S: Se ratio in Pt_{SA}-MoS_xSe_{2-x} and overpotential at 10 mA cm^{-2} and Tafel slope.

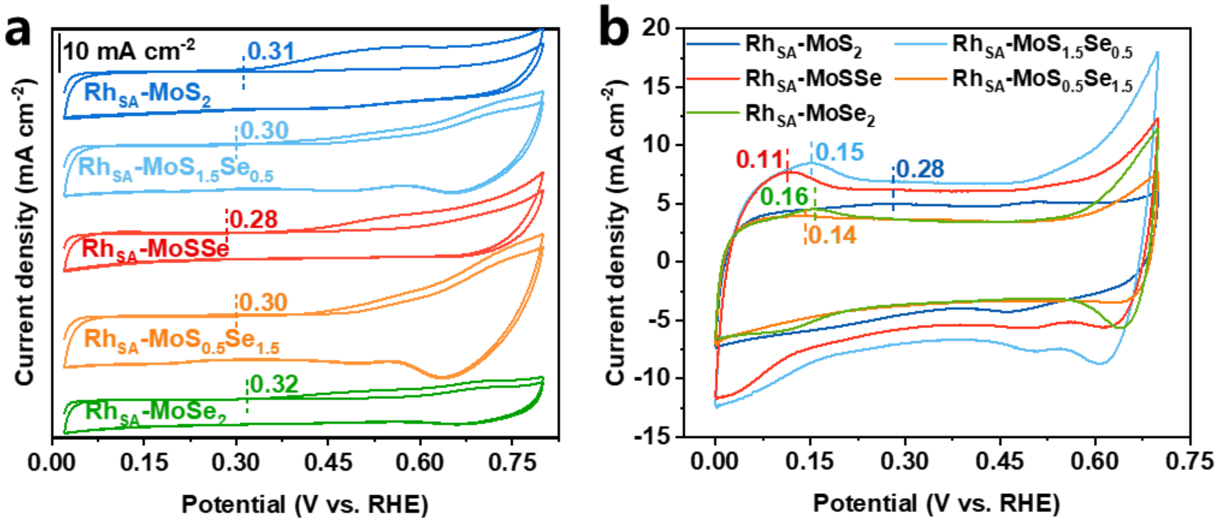

Supplementary Fig. 47. (a) CO-stripping voltammetry curves and (b) CV curves of Rh$_{SA}$-MoS$_2$, Rh$_{SA}$-MoS$_{1.5}$Se$_{0.5}$, Rh$_{SA}$-MoSSe, Rh$_{SA}$-MoS$_{0.5}$Se$_{1.5}$, and Rh$_{SA}$-MoSe$_2$.

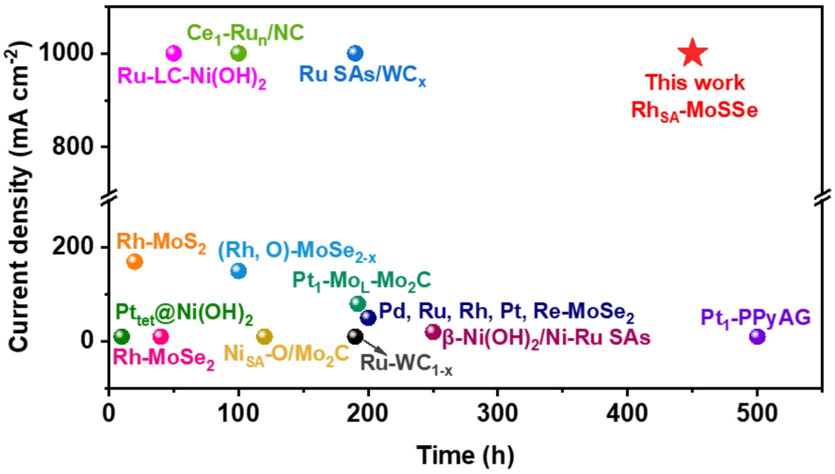

Supplementary Fig. 48. Comparison of HER stability for Rh_{SA}-MoS_xSe_{2-x} with previously reported state-of-the-art noble metal-based and TMDs-based catalysts.

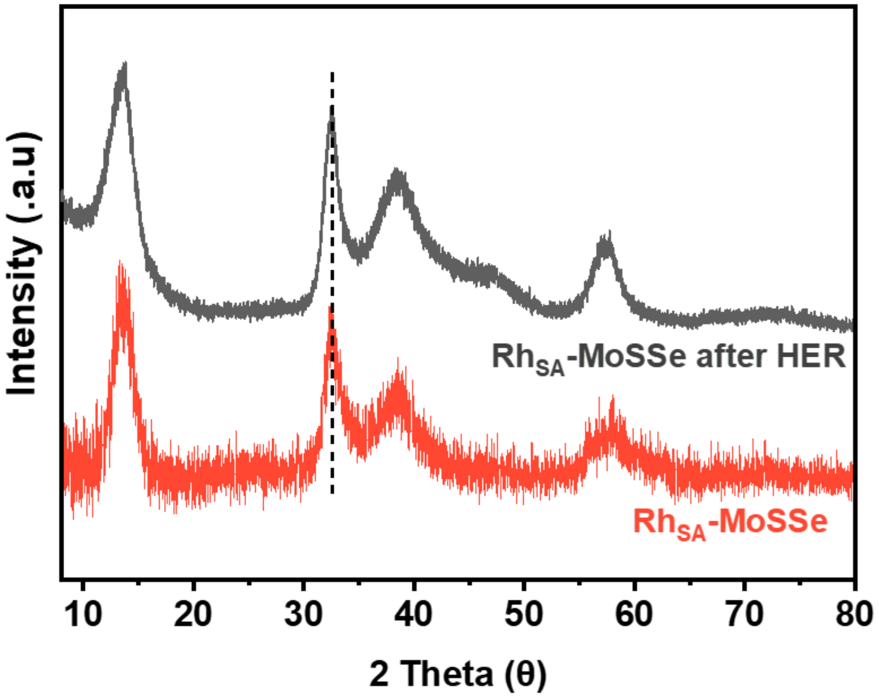

Supplementary Fig. 49. XRD patterns of Rh$_{SA}$-MoSSe before and after long-term HER testing.

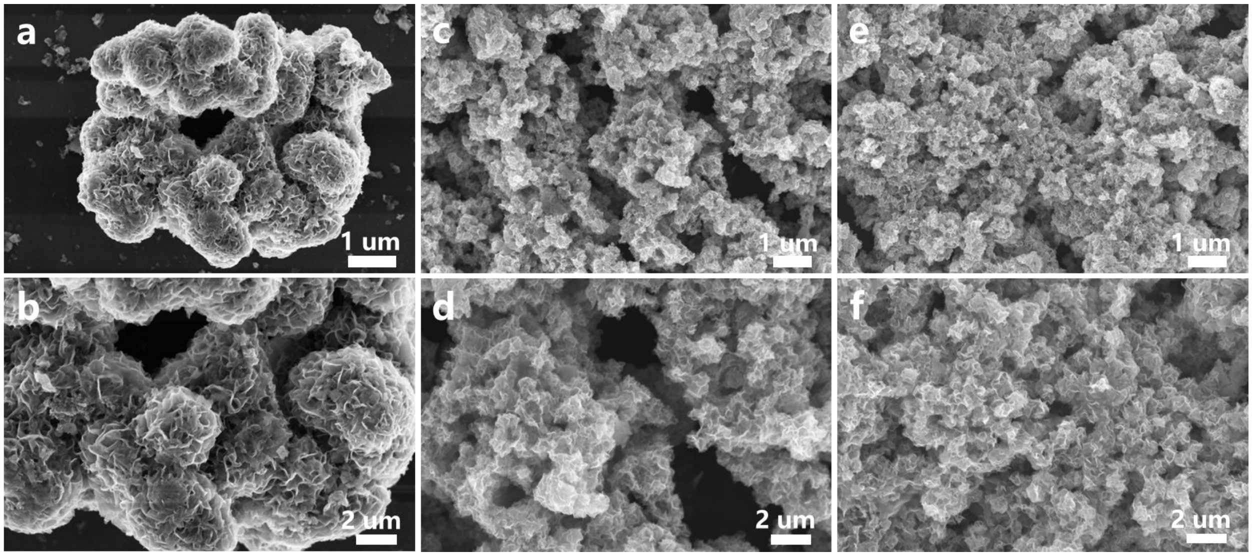

Supplementary Fig. 50. SEM images of (a, b) Rh_{SA}-MoS_2, (c, d) Rh_{SA}-MoSSe, and (e, f) Rh_{SA}-$MoSe_2$ after long-term HER testing.

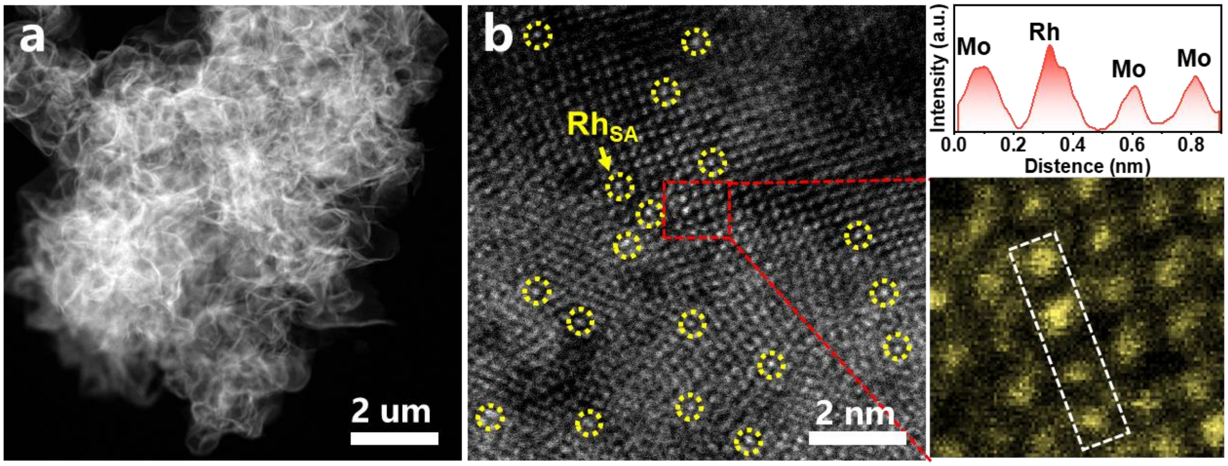

Supplementary Fig. 51. ACTEM images of Rh_{SA}-MoSSe after long-term HER testing.

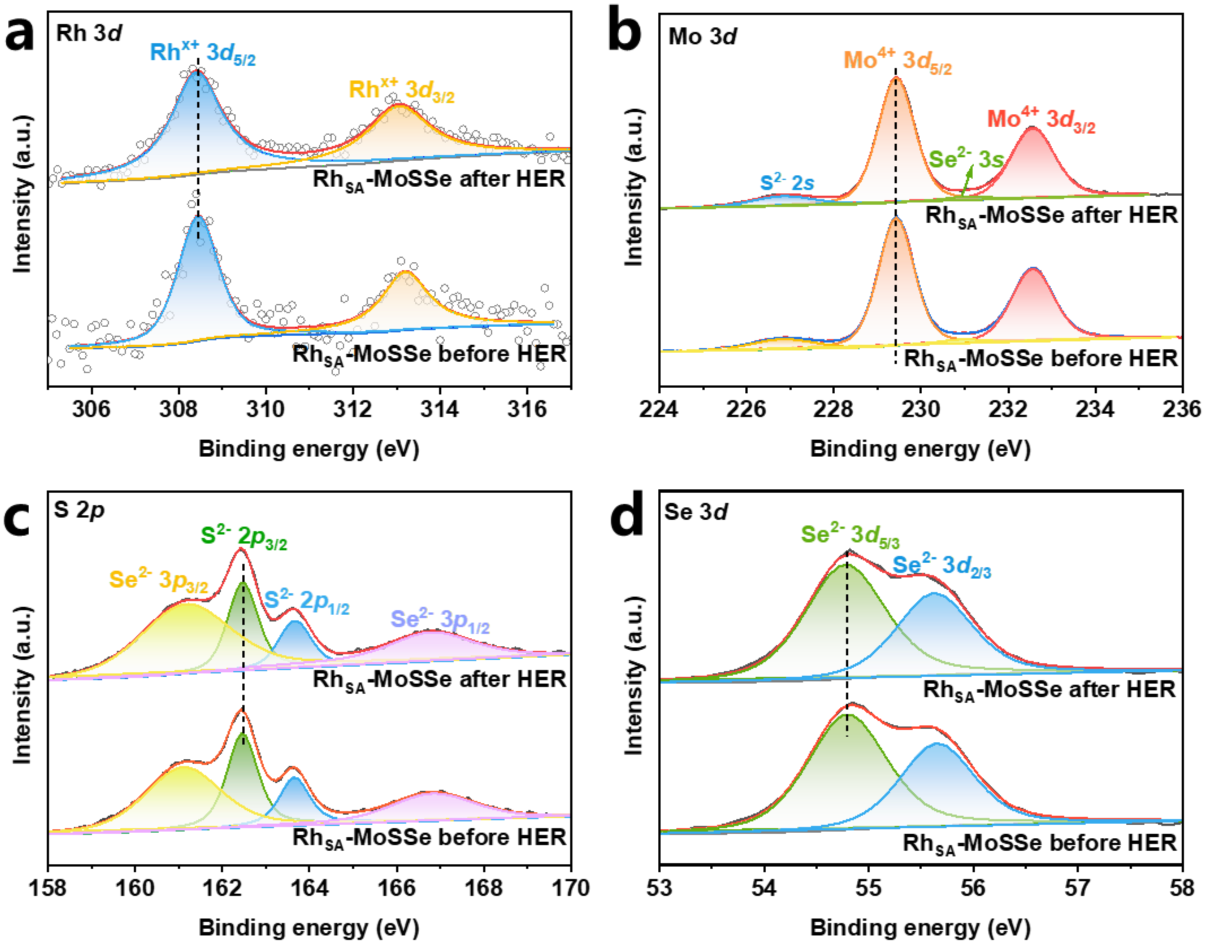

Supplementary Fig. 52. XPS spectra of (a) Rh 3*d*, (b) Mo 3*d*, (c) S 2*p*, and (d) Se 3*d* for Rh_{SA}-MoSSe before and after long-term HER testing.

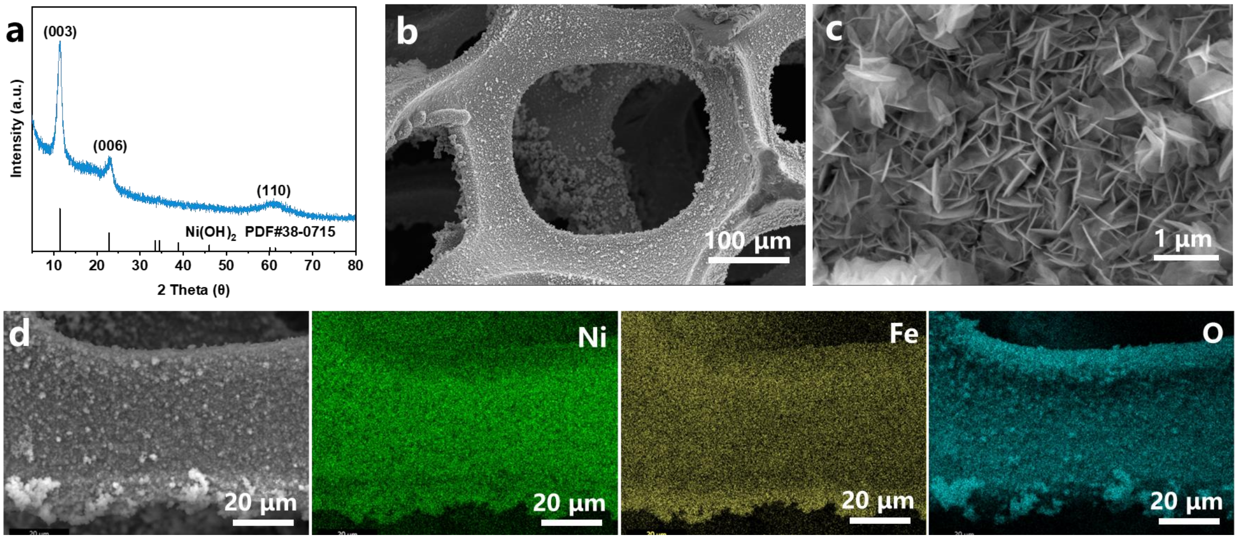

Supplementary Fig. 53. (a) XRD patterns of homemade NiFe LDH@NF. SEM images (b and c) and corresponding EDS elemental mapping (d) of NiFe LDH@NF.

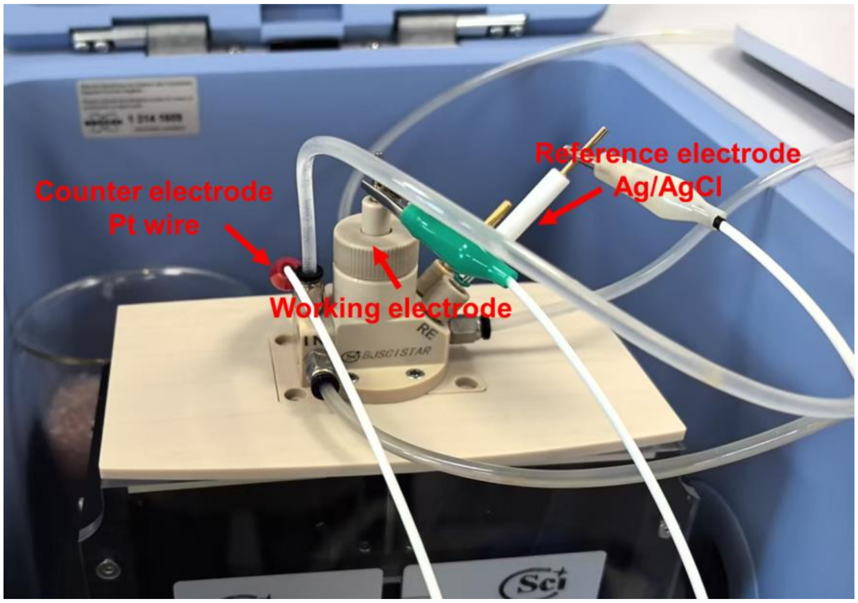

Supplementary Fig. 54. Picture of the custom-built electrochemical cell for operando FTIR spectroscopy. The cell is a flow cell configuration equipped with a single-crystal silicon (Si) crystal serving as the IR-transparent window. A three-electrode setup is used: the working electrode is a catalyst-coated electrode placed in direct contact with the Si crystal, a platinum wire counter electrode, and an Ag/AgCl reference electrode. The electrolyte flows through the cell continuously during measurement. The IR beam enters the Si crystal, undergoes total internal reflection, and interacts with the electrode/electrolyte interface at the evanescent wave region, enabling real-time monitoring of surface species under electrochemical reaction conditions.

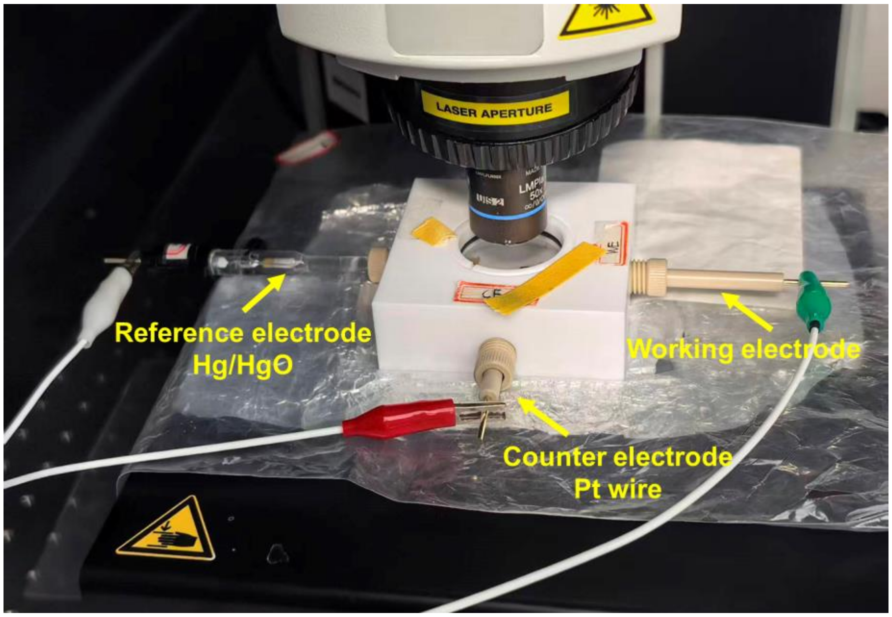

Supplementary Fig. 55. Picture of the custom-built electrochemical cell for operando Raman spectroscopy. The cell consists of a three-electrode configuration: a working electrode placed facing a quartz window to allow laser illumination and Raman signal collection, a platinum wire counter electrode, and a Hg/HgO reference electrode. The electrolyte (20 mL) is contained in a non-sealed cell body. The laser beam focuses on the WE surface. The cell geometry ensures a thin electrolyte layer above the working electrode for optimal Raman signal.

Supplementary Table 1. The best-fitted EXAFS results of Rh_{SA}-MoS_2, Rh_{SA}-MoSSe, and Rh_{SA}-$MoSe_2$.

Sample	Shell	CN	R (Å)	ΔR (Å)	σ^2 (10^{-2} Å^2)	R-factor (%)
Rh_{SA}-MoS_2	Rh-S	3.5	2.32	-0.06	0.7	1.1
Rh_{SA}-MoSSe	Rh-S	1.9	2.24	-0.12	1.3	0.3
Rh_{SA}-MoSSe	Rh-Se	1.6	2.51	-0.05	0.4	0.4
Rh_{SA}-$MoSe_2$	Rh-Se	3.4	2.53	0.03	0.8	1.9

Supplementary Table 2. Comparison of HER activity for Rh_{SA}-MoS_xSe_{2-x} with previously reported state-of-the-art noble metal-based and TMDs-based catalysts.

Catalysts	electrolyte	Mass loading	TOF (S^{-1})	Ref.
Rh_{SA}-MoSSe	1 M KOH	2.5 mg cm^{-2}	19.34@100 mV	This work
Rh_{SA}-MoS_2	1 M KOH	2.5 mg cm^{-2}	2.15@100 mV	This work
Rh_{SA}-$MoS_{1.5}Se_{0.5}$	1 M KOH	2.5 mg cm^{-2}	6.05@100 mV	This work
Rh_{SA}-$MoS_{0.5}Se_{1.5}$	1 M KOH	2.5 mg cm^{-2}	9.89@100 mV	This work
Rh_{SA}-$MoSe_2$	1 M KOH	2.5 mg cm^{-2}	6.97@100 mV	This work
Rh-MoS_2	0.5 M H_2SO_4	0.5 mg cm^{-2}	5.33@100 mV	1
Rh-$MoSe_2$	0.5 M H_2SO_4	0.285 mg cm^{-2}	0.29@50 mV	2
Pt/np-$Co_{0.85}Se$	1.0 M PBS	2.04 mg cm^{-2}	3.93@100 mV	3
np-Co0.85Se	1.0 M PBS	2.04 mg cm^{-2}	0.17@100 mV	3
Ce_1-Ru_n/NC	1 M KOH	/	5.23@50 mV	4
Ru_n/NC	1 M KOH	/	0.9@50 mV	4
Pd, Ru, Rh, Pt, Re-$MoSe_2$	1 M KOH	0.354 mg cm^{-2}	3.34@200 mV	5
Rh, Pd, Re-$MoSe_2$	1 M KOH	0.354 mg cm^{-2}	1.86@200 mV	5
Pd, Re-$MoSe_2$	1 M KOH	0.354 mg cm^{-2}	0.34@200 mV	5
Rh-MoS_2	1 M KOH	0.309 mg cm^{-2}	0.50@50 mV	6
Pt/VG Ar-5	1 M KOH	/	22.6@150 mV	7
Pt-SAs/$MoSe_2$	1 M KOH	0.06 mg cm^{-2}	6.21@50 mV	8
Pt-SAs/MoS_2	1 M KOH	0.07 mg cm^{-2}	1.02@50 mV	8
Pt-SAs/WS_2	1 M KOH	0.08 mg cm^{-2}	0.56@50 mV	8

Pt-SAs/WSe_2	1 M KOH	0.07 mg cm^{-2}	0.52@50 mV	8
Cu-Ru/$RuSe_2$ NSs/C	1 M KOH	/	0.88@100 mV	9
p-1T-MoS_2	0.5 M H_2SO_4	0.14 mg cm^{-2}	0.5@153 mV	10

Supplementary Table 3. Comparison of HER stability for Rh_{SA}-MoS_xSe_{2-x} with previously reported state-of-the-art noble metal-based and TMDs-based catalysts.

Catalysts	electrolyte	Current density	Time	Ref.
Rh_{SA}-MoSSe	1 M KOH	1000 mA cm^{-2}	> 450 h	This work
Rh-$MoSe_2$	0.5 M H_2SO_4	10 mA cm^{-2}	40 h	2
Pt/np-$Co_{0.85}Se$	1.0 M PBS	10 mA cm^{-2}	40 h	3
Ce_1-Ru_n/NC	1 M KOH	1000 mA cm^{-2}	100 h	4
Pd, Ru, Rh, Pt, Re-$MoSe_2$	1 M KOH	50 mA cm^{-2}	200	5
Rh-MoS_2	1 M KOH	170 mA cm^{-2}	20 h	6
Pt_{tet}@$Ni(OH)_2$	1 M KOH	10 mA cm^{-2}	10 h	11
Ru SAs/WC_x	1 M KOH	1000 mA cm^{-2}	190 h	12
Ru-WC_{1-x}	1 M KOH	10 mA cm^{-2}	190 h	13
Pt_1-Mo_L-Mo_2C	1 M KOH	80 mA cm^{-2}	192 h	14
Ru-YNC	1 M KOH	500 mA cm^{-2}	1000 h	15
Ni_{SA}-O/Mo_2C	1 M KOH	10 mA cm^{-2}	120 h	16
Pt_1-PPyAG	1 M KOH	10 mA cm^{-2}	500 h	17
β-$Ni(OH)_2$/Ni-Ru SAs	1 M KOH	20 mA cm^{-2}	250 h	18
Ru-LC-$Ni(OH)_2$	1 M KOH	1000 mA cm^{-2}	50 h	19
2H@1T-MoS_2-Sn_1	0.5 M H_2SO_4	10 mA cm^{-2}	240 h	20
Tm SAs-MoS_2	1 M KOH	10 mA cm^{-2}	27 h	21
(Rh, O)-$MoSe_{2-x}$	1 M KOH	150 mA cm^{-2}	100 h	22
1T p_z-MoS_2	1 M KOH	100 mA cm^{-2}	50 h	23
FeCo-MoS_2	0.5 M H_2SO_4	1000 mA cm^{-2}	350 h	24

Supplementary Table 4. Comparison of cell voltage at a current density of 1 A cm^{-2} and stability time for recently reported catalysts applied in AEMWE

Catalysts	Electrolyte	Temperature (°C)	Cell Voltage (V) @j = 1 A cm^{-2}	Stability Time	Ref.
Rh_{SA}-MoSSe‖NiFe LDH@NF	1 M KOH	80	1.70	500 h@1 A cm^{-2}	This work
Pd, Ru, Rh, Pt, Re-$MoSe_2$‖IrO_2	1 M KOH	80	1.82	500 h@1 A cm^{-2}	5
Ru SAs/WC_x‖$NiFeOH_x$-NF	1 M KOH	80	1.79	190 h@1 A cm^{-2}	12
Ru-WC_{1-x}‖$NiFeO_x$	1 M KOH	70	1.80	100 h@1 A cm^{-2}	13
Ru-YNC‖NiFe LDH	1 M KOH	80	1.87	1000 h@0.5 A cm^{-2}	15
Ru-LC-$Ni(OH)_2$‖FeNi LDH	1 M KOH	80	1.69	500 h@0.5 A cm^{-2}	19
(Rh, O)-$MoSe_{2-x}$‖IrO_2	1 M KOH	80	No.	300 h@1.5 A cm^{-2}	22
1T p_z-MoS_2‖$RuIrO_2$	1 M KOH	80	1.78	200 h@0.5 A cm^{-2}	23
FeCo-MoS_2	0.5 M H_2SO_4	85	2.23	350 h@1 A cm^{-2}	24

$Pt_1/Ni(OH)_2$‖$Ir_1/Co(OH)_2$	1 M KOH	80	2.10	1030 h@0.8 A cm^{-2}	25
Co_n-Pt_1@NPC‖NiFe LDH	1 M KOH	70	2.03	100 h@1 A cm^{-2}	26

Supplementary References

1. Meng, X. *et al.* Distance synergy of MoS_2-confined rhodium atoms for highly efficient hydrogen evolution. *Angew. Chem. Int. Ed.* 59, 10502-10507 (2020).
2. Liu, S. *et al.* Tuning the electronic structure of Se via constructing Rh-$MoSe_2$ nanocomposite to generate high-performance electrocatalysis for hydrogen evolution reaction. *ACS Sustain. Chem. Eng.* 6, 9137-9144 (2018).
3. Jiang, K. *et al.* Single platinum atoms embedded in nanoporous cobalt selenide as electrocatalyst for accelerating hydrogen evolution reaction. *Nat. Commun.* 10, 1743 (2019).
4. Shen, F. *et al.* Oxophilic Ce single atoms-triggered active sites reverse for superior alkaline hydrogen evolution. *Nat. Commun.* 15, 448 (2024).
5. Luo, Z. *et al.* Creating high-entropy single atoms on transition disulfides through substrate-induced redox dynamics for efficient electrocatalytic hydrogen evolution. *Angew. Chem. Int. Ed.* 63, e202405017 (2024).
6. Cheng, Y. *et al.* Rh-MoS_2 nanocomposite catalysts with Pt-like activity for hydrogen evolution reaction. *Adv. Funct. Mater.* 27, 2-7 (2017).
7. Tsounis, C. *et al.* Pt single atom electrocatalysts at graphene edges for efficient alkaline hydrogen evolution. *Adv. Funct. Mater.* 32, 2203067 (2022).
8. Shi, Y. *et al.* Electronic metal-support interaction modulates single-atom platinum catalysis for hydrogen evolution reaction. *Nat. Commun.* 12, 3021 (2021).
9. Wang, K. *et al.* Cu-doped heterointerfaced Ru/$RuSe_2$ nanosheets with optimized H and H_2O adsorption boost hydrogen evolution catalysis. *Adv. Mater.* 35, 2300980 (2023).

10. Yin, Y. *et al.* Contributions of phase, sulfur vacancies, and edges to the hydrogen evolution reaction catalytic activity of porous molybdenum disulfide nanosheets. *J. Am. Chem. Soc.* 138, 7965-7972 (2016).

11. Wan, C. *et al.* Amorphous nickel hydroxide shell tailors local chemical environment on platinum surface for alkaline hydrogen evolution reaction. *Nat. Mater.* 22, 1022-1029 (2023).

12. Lin, X. *et al.* Alleviating OH blockage on the catalyst surface by the puncture effect of single-atom sites to boost alkaline water electrolysis. *J. Am. Chem. Soc.* 146, 4883-4891 (2024)

13. Zhang, H. *et al.* Revealing the correlation of loading-to-performance of single atom catalysts. *Angew. Chem. Int. Ed.* 64, e202506563 (2025).

14. Xu, L. *et al.* Surface single atom alloys for alkaline hydrogen evolution reaction. *Adv. Mater.* 37, 2502989 (2025).

15. Zhang, H. *et al.* Cascade electrocatalysis via integration of ruthenium clusters and yttrium single atoms for a boosted alkaline hydrogen evolution reaction. *Energy Environ. Sci.* 18, 6141-6153 (2025).

16. Hou, M. *et al.* Microenvironment reconstitution of highly active Ni single atoms on oxygen-incorporated Mo_2C for water splitting. *Nat. Commun.* 15, 1342 (2024).

17. Zhang, L. *et al.* Atomically dispersed platinum on conjugated polymers with tailored cation-π interactions and microstructures for hydrogen evolution electrocatalysis. *Adv. Funct. Mater.* 34, 240707 (2024).

18. Yan, P. *et al.* 'One stone five birds' plasma activation strategy synergistic with Ru single atoms doping boosting the hydrogen evolution performance of metal hydroxide. *Adv. Funct. Mater.* 33, 2301343 (2023).

19. Wang, L. *et al.* Manipulating the microenvironment of single atoms by switching support crystallinity for industrial hydrogen evolution. *Angew. Chem. Int. Ed.* 63, e202317220 (2024).

20. Jin, H., *et al*. Atomically dispersed Sn on core-shell MoS_2 nanoreactors as Mott-Schottky phase junctions for efficient electrocatalytic hydrogen evolution. *Adv. Mater.* 37, 2502977 (2025).

21. Li, M. *et al.* Oxophilic Tm-sites in MoS_2 trigger thermodynamic spontaneous water dissociation for enhanced hydrogen evolution. *Adv. Energy Mater.* 14, 2401716 (2024).

22. Luo, Z. *et al.* Synergistic M-O dual-atom pairs induced interfacial water hydrogen bonding network for boosting $MoSe_2$ electrocatalytic performance. *Adv. Funct. Mater.* 34, 2405881 (2024).

23. Dai, L. *et al.* Charge-buffered sulfidation stabilized B δ^- in 1T MoS_2 : Orbital alignment for efficient alkaline hydrogen production. *Adv. Mater.* 37, e09904 (2025).

24. Zhang, Y. *et al.* Synergy of MoS_2-confined Fe and Co atoms enhances hydrogen evolution. *Adv. Funct. Mater.* 35, 2503549 (2025).

25. Cao, D. *et al.* Fabricating lattice-confined Pt single atoms with high electron-deficient state for alkali hydrogen evolution under industrial-current density. *Adv. Mater.* 37, 2414138 (2025).

26. Xu, Y. *et al.* Co nanoparticle: An efficient H-pump for Pt single atoms towards enhanced hydrogen spillover. *Angew. Chem. Int. Ed.* 64, e202502227 (2025).
