# Supplementary material for: Frontier-orbital modulation of rhodium single-atom catalysts for enhanced hydrogen evolution
Source: Nat Commun. 2026 May 18;17:6523. doi: 10.1038/s41467-026-73161-6 (PMC13376774; doi:10.1038/s41467-026-73161-6)
Supplement: Supplementary file 2 — Description of Additional Supplementary Files [file 41467_2026_73161_MOESM2_ESM.pdf]

Description of Additional Supplementary Files

File Name: Supplementary Data 1

Description: CIF of computational models for MoS_2, $MoS_{1.5}Se_{0.5}$, MoSSe, $MoS_{0.5}Se_{1.5}$, $MoSe_2$, Rh_{SA}-MoS_2, Rh_{SA}-$MoS_{1.5}Se_{0.5}$, Rh_{SA}-MoSSe, Rh_{SA}-$MoS_{1.5}Se_{0.5}$, and Rh_{SA}-$MoSe_2$.
