# Supplementary material for: Frontier-orbital modulation of rhodium single-atom catalysts for enhanced hydrogen evolution
Source: Nat Commun. 2026 May 18;17:6523. doi: 10.1038/s41467-026-73161-6 (PMC13376774; doi:10.1038/s41467-026-73161-6)
Supplement: Supplementary file 4 — Transparent Peer Review file [file 41467_2026_73161_MOESM4_ESM.pdf]

nature portfolio

Peer Review File

Frontier-Orbital Modulation of Rhodium Single-Atom Catalysts for Enhanced Hydrogen Evolution

Corresponding Author: Professor Yida Deng

Parts of this Peer Review File have been redacted as indicated to remove third-party material.

This file contains all reviewer reports in order by version, followed by all author rebuttals in order by version.

Version 0:

Reviewer comments:

Reviewer #1

(Remarks to the Author)
The manuscript introduces an anion-engineered single-atom catalyst platform, where Rh single atoms are anchored on MoSxSe2-x supports to tune the d–p orbital hybridization between Rh and chalcogen atoms. By gradually varying the S/Se ratio, the authors claim to establish a volcano-type relationship between the d-band center of Rh and the adsorption energies of H and OH intermediates, thereby achieving both high activity and long-term stability in alkaline HER. The concept of using anion engineering to control metal–support interactions at the orbital level is intriguing and, in principle, of interest to the Nature Communications readership. However, in its current form, the manuscript requires substantial revision before it can be further considered. Specific points are as follows:
1. The electrochemical deposition of Rh single atoms on MoSxSe2-x substrates results in different Rh contents depending on the S/Se ratio. The manuscript does not provide a clear explanation for this phenomenon.
2. The authors present adsorption Gibbs free energies of OH and H, which are certainly important descriptors in alkaline HER. Nevertheless, the crucial step of water dissociation ($H_2O \rightarrow H^* + OH^*$) is not explicitly addressed. Additional calculations showing how the S/Se ratio influences the water dissociation barrier and the preferred active site for this step would significantly strengthen the mechanistic claims.
3. XAFS is indeed a powerful tool to probe local atomic coordination. However, the current EXAFS results are only qualitatively described. Detailed EXAFS fitting data, including coordination numbers, bond distances, and Debye–Waller factors, should be provided along with fitting tables to confirm the proposed Rh–S/Se coordination environment.
4. While the manuscript provides extensive XPS characterization of S/Se ratio effects, the XAFS analysis is insufficient. Given that XAFS directly reveals the coordination environment of single atoms, the dataset should be expanded. This would allow a more convincing correlation between structural evolution and catalytic performance.
5. To demonstrate practical relevance, it is strongly recommended to evaluate the catalyst under realistic device conditions. Specifically, membrane electrode assembly (MEA) or anion exchange membrane water electrolyzer (AEMWE) tests should be provided to validate whether the catalyst can maintain its activity and durability in full-cell configurations at technologically relevant current densities.
6. The manuscript also contains typographical errors that should be carefully corrected. (Fig. 5g).

Reviewer #2

(Remarks to the Author)
This manuscript presents a comprehensive study on rhodium single-atom catalysts supported on molybdenum sulfide selenide (MoSxSe2-x) for hydrogen evolution reactions. The study addresses the precise modulation of metal-support interactions at the orbital level and their impact on catalytic performance. The authors employ a systematic approach by varying the support composition and correlating these electronic effects with HER activity, stability, and intermediate binding energies. However, for practical relevance and commercialization, electrochemical catalysts should ideally be evaluated at the single-cell level. Therefore, the reviewer recommends major revisions, including the addition of AEMWE data, before the manuscript is suitable for publication in this journal.

This reviewer raises the following questions:

1. The authors claim that the d-band center of Rh in the RhSA-MoSxSe2-x series shows a volcano-type dependence with ΔGH* and ΔGOH*. However, according to conventional d-band theory, adsorption energies usually scale linearly with the d-band position, where an upshifted d-band leads to stronger adsorption. In Figure 1c, the center shift due to d-p hybridization is thought to follow a linear trend. This may be considered as a discrepancy between Figures c and d, requiring further explanation for the origin of the volcano-like behavior. The discussion would be further strengthened if a mechanistic basis linking the adsorption energy and the observed volcano-like trend was presented.
2. In Figure 1e, the authors claim that the integration of Rh sites into molybdenum sulfide selenide substantially enhances H and OH adsorption. It would strengthen the manuscript to provide a more detailed discussion on how the ΔGH* and ΔGOH* values are improved, specifically whether these values fall within the optimal range for HER activity for each sample. Including such analysis would help clarify the correlation between the observed adsorption enhancements and the expected catalytic performance.
3. The key point of this work is that the support properties are modulated and the electron density of Rh is tuned through S and Se with higher ionic electronegativity. However, despite their similar electronegativities, the reason why changing the ratio of S and Se affects the properties is not clearly explained. The authors should clarify the underlying mechanism, including the differences in Rh–S and Rh–Se bonding interactions and the relative contributions of π- and σ-bonding, to explain how these changes influence the electronic structure and catalytic behavior.
4. In Figure 3d, it is unclear why the number of active sites differs. If the observed performance difference is attributed to an increase in the number of active sites, the authors should clearly specify the proposed active sites and describe the method used to determine the number of active sites.
5. To demonstrate the practical relevance and support claims of high activity and durability, it is recommended that the authors perform actual AEMWE (Anion Exchange Membrane Water Electrolysis) tests. Such evaluations would provide more realistic insight into the catalyst performance under operating conditions. Including these results would significantly strengthen the manuscript by bridging fundamental electrochemical measurements and practical applicability.
6. We request the following minor corrections:
1) The sample names in Figure 5g should be corrected.
2) On page 4, line 72, the uppercase letter should be changed to lowercase.

Reviewer #3

(Remarks to the Author)
In this work, Jia et al. studied MoSSe with varied S/Se ratios decorated with Rh single atoms. The authors modulated the d-p orbital hybridizations between the Rh and S/Se and studied their influences on the HER activity and stability of the catalysts toward the hydrogen evolution reaction in alkaline media. This is a complete and detailed study, with both experimental and theoretical investigations. However, this work is lacking of novelty and significance in terms of synthetic method, materials innovation and catalytic mechanism, and the findings does not represent significant advancements in the field. The materials of MoS/Se and their modified forms (especially single atom-doped ones) have been widely studied for HER electrocatalysis. In addition, whether the issue of activity-stability trade-off proposed by the authors exists remains unclear and the analyses on how they break this trade-off is insufficient. Overall, this reviewer believes that this work does not meet the high standards of Nature Communications. Some specific comments were provided below for the authors to consider to improve their work.
1. One key message that the authors try to convey is the breakage of the activity-stability trade-off by the Ru-MoSSe catalysts. However, the specific meaning and the fundamental causes behind this trade-off is not clarified nor analyzed.
2. The rationale for choosing Rh single atoms to study the d-p orbital hybridization for enhanced HER should be explicitly provided. Considering that Pt and Ru precious metals are more frequently and suitable for HER electrocatalysis, Pt and Ru single atoms should be studied to demonstrate the generality of the design strategy.
3. For the performance comparison with previous works, the stability as an important parameter should be included and analyzed in detail.
4. The performance of the catalysts was analyzed in conventional three-electrode system. Considering that MoS2 and its modified form as HER catalysts have been extensively reported, their catalytic performance in anion-exchange membrane full cells is recommended to be included to evaluate their potential for practical applications.
5. For the experimental parts, the authors synthesized Rh-MoSSe catalysts with five different Se/S ratios. However, for the theoretical parts, only ratios of 2:0, 1:1, 0:2 were studied. As the authors highlight that the modulation is gradient-continuous, more Se/S ratios should be studied, which additionally help to identify the optimal catalyst composition.
6. For Fig. S11, the peak shifts in the XRD patterns among different catalysts should be explained.
7. For the Pt/C catalyst in Fig. S17, why two Tafel slopes in different potential regions were calculated?
8. What makes MoSSe (with or without Ru) have higher ECSA than both MoS2 and MoSe2?

Version 1:

Reviewer comments:

Reviewer #1

(Remarks to the Author)
The authors adequately responded all reviewers' concerns, and thus it seems ready for publication now.

Reviewer #3

(Remarks to the Author)
The revised manuscript has been improved to the meet the high standard of Nature Communications, and this reviewer recommends its publication.

Reviewer #4

(Remarks to the Author)
The authors have provided thorough, systematic, and compelling responses to the reviewer's concerns. Their revisions, particularly the expansion of theoretical analysis and addition of key computational and experimental data, have significantly strengthened the manuscript. The work now presents a cohesive story that successfully bridges atomistic design principles with macroscopic catalytic performance. From a computational perspective, the theoretical foundation is sound and well-integrated. Therefore, I recommend acceptance of the manuscript but could be better if the following concerns are fixed. Here are several points that should be enhanced:
1. The explanation of differing Rh-S and Rh-Se interactions based on polarizability and atomic size is chemically intuitive. To provide deeper, quantitative insight, the authors could consider including: a) Crystal Orbital Hamiltonian Population (COHP) analysis: To quantify the bonding/antibonding character and strength of Rh-S vs. Rh-Se bonds; b) Electron Localization functon(ELF) or Bader charge analysis: To provide a more detailed picture of charge redistribution and bond covalency/ionicity.
2. Replacing "number of active sites" with "ECSA" is a good correction. However, the discussion should carefully distinguish between electrochemically accessible surface area (related to double-layer capacitance) and the intrinsic activity per catalytic site. The authors' normalization of current to ECSA is the correct approach to isolate intrinsic activity.
3. While the volcano plot is presented, establishing a more quantitative link between the computed adsorption energies/water dissociation barriers and the experimental overpotential/Tafel slope via microkinetic modeling or applying the Brønsted-Evans-Polanyi (BEP) relation would represent a pinnacle of computational electrocatalysis. This could be suggested as a direction for future work if beyond the current scope.

Version 2:

Reviewer comments:

Reviewer #4

(Remarks to the Author)
My concerns have been addressed in this revision, and I recommend the publication of this current version.

Open Access This Peer Review File is licensed under a Creative Commons Attribution 4.0 International License, which permits use, sharing, adaptation, distribution and reproduction in any medium or format, as long as you give appropriate credit to the original author(s) and the source, provide a link to the Creative Commons license, and indicate if changes were made.
In cases where reviewers are anonymous, credit should be given to 'Anonymous Referee' and the source.
The images or other third party material in this Peer Review File are included in the article's Creative Commons license,

unless indicated otherwise in a credit line to the material. If material is not included in the article's Creative Commons license and your intended use is not permitted by statutory regulation or exceeds the permitted use, you will need to obtain permission directly from the copyright holder.
To view a copy of this license, visit https://creativecommons.org/licenses/by/4.0/

Response to the reviewers' comments

We are grateful for the reviewers' in-depth comments and suggestions, which have helped us greatly to improve the quality of this manuscript. We have considered the comments and suggestions seriously, and carried out the related experiments following the suggestions of the reviewers. We have made detailed and **point-to-point response** and revised the manuscript accordingly with all changes being highlighted in yellow in the revised manuscript and Supporting Information.

Reviewer #1 (Remarks to the Author)

The manuscript introduces an anion-engineered single-atom catalyst platform, where Rh single atoms are anchored on MoS_xSe_{2-x} supports to tune the d-p orbital hybridization between Rh and chalcogen atoms. By gradually varying the S/Se ratio, the authors claim to establish a volcano-type relationship between the d-band center of Rh and the adsorption energies of H and OH intermediates, thereby achieving both high activity and long-term stability in alkaline HER. The concept of using anion engineering to control metal-support interactions at the orbital level is intriguing and, in principle, of interest to the Nature Communications readership. However, in its current form, the manuscript requires substantial revision before it can be further considered. Specific points are as follows:

Response: We have made our response point-by-point, and we believe that the quality of our work has been improved by addressing the following concerns.

Comment: 1) *The electrochemical deposition of Rh single atoms on MoS_xSe_{2-x} substrates results in different Rh contents depending on the S/Se ratio. The manuscript does not provide a clear explanation for this phenomenon.*

Response: Thank you very much for the valuable comment. The Rh single-atoms anchored on a series of MoS_xSe_{2-x} were prepared based on a universal electrochemical deposition method. In the interest of rigor, we retested the Rh loadings on MoS_xSe_{2-x} by inductively coupled plasma optical emission spectrometry (ICP-OES) and averaged (Fig. R1). The mass loadings of Rh on MoS_2, $MoS_{1.5}Se_{0.5}$, MoSSe, $MoS_{0.5}Se_{1.5}$, and $MoSe_2$ were 0.37 wt.%, 0.42 wt.%, 0.49 wt.%, 0.44 wt.%, and 0.39 wt.%, respectively. The mass loading of metal species is depended on the concentration of metal precursors, number of scanning cycles, scanning rate, conductivity of substrates, and strong metal-support

interaction (*Nat. Commun.* **11**, 1215 (2020)). In this work, all Rh_{SA}-MoS_xSe_{2-x} were synthesized with identical concentration of Rh precursors, number of scanning cycles, and scanning rate. Therefore, the observed variation in Rh loading is attributed to differences in substrates conductivity and the strong metal-support interactions. The principle is as follows: According to the hard-soft acid-base (HSAB) principle (*Chem. Rev.* **75**, 1–20 (1975); *Angew. Chem. Int. Ed.* **64**, e202514010 (2025); *J. Am. Chem. Soc.* **147**, 20748–20758 (2025)), the soft acid Rh^{3+} preferentially interacts with the softer base Se over S. Consequently, the higher conductivity of Se than S results in a higher Rh mass loading on $MoSe_2$ than MoS_2. Owing to the built-in electric field caused by the electron transfer from Se to S (*J. Phys. Chem. C* **122**, 24520–24526 (2018), *J. Mater. Chem. A* **6**, 2295–2301 (2018)), the MoS_xSe_{2-x} with both S and Se anions exhibits superior conductivity and stronger metal-support interaction than MoS_2 and $MoSe_2$. In addition, the adsorption energies of Rh on MoS_2, MoSSe, and $MoSe_2$ calculated by DFT are -3.66 eV, -3.91 eV, and -3.85 eV, respectively (Fig. R2). Therefore, the mass loading of Rh species on MoS_xSe_{2-x} substrates is in the order of MoSSe > $MoS_{0.5}Se_{1.5}$ ≈ $MoS_{1.5}Se_{0.5}$ > $MoSe_2$ > MoS_2, which are consistent with above experimental basis.

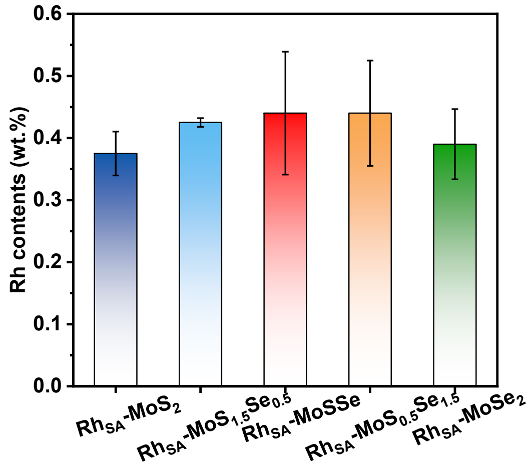

Fig. R1 (Supplementary Fig. 25). The mass loading of Rh species on MoS_xSe_{2-x} substrates.

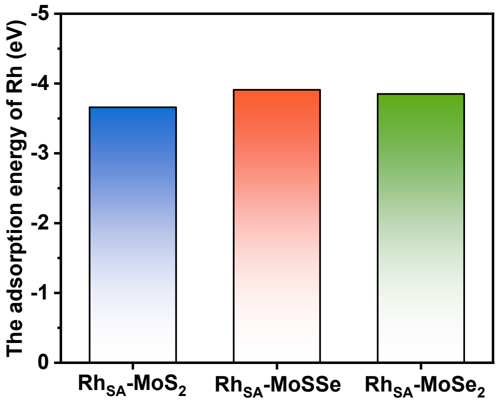

Fig. R2 (Supplementary Fig. 26). The adsorption energy of Rh on MoS_2, MoSSe, and $MoSe_2$.

We have also added these contents to the revised manuscript highlighted in yellow in Line 4-20, Page 8, "The Rh loadings were comparable across the samples (Rh_{SA}-MoS_2: 0.37 wt.%, Rh_{SA}-$MoS_{1.5}Se_{0.5}$: 0.42 wt.%, Rh_{SA}-MoSSe: 0.49 wt.%, Rh_{SA}-$MoS_{0.5}Se_{1.5}$: 0.44 wt.%, and Rh_{SA}-$MoSe_2$: 0.39 wt.%), as determined by inductively coupled plasma optical emission spectrometry (ICP-OES) (Supplementary Fig. 25). Given that all Rh_{SA}-MoS_xSe_{2-x} samples were synthesized using the same Rh precursor concentration, number of scanning cycles, and scanning rate, the observed variation in Rh loading is attributed to differences in substrates conductivity and the strong metal-support interactions[35]. The principle is as follows: According to the hard-soft acid-base (HSAB) principle[36–38], the soft acid Rh^{3+} preferentially interacts with the softer base Se over S. Consequently, the higher conductivity of Se than S results in a higher Rh mass loading on $MoSe_2$ than MoS_2. Owing to the built-in electric field caused by the electron transfer from Se to S[39,40], the MoS_xSe_{2-x} with both S and Se anions exhibits superior conductivity and stronger metal-support interaction than MoS_2 and $MoSe_2$. In addition, the adsorption energies of Rh atoms on MoS_2, MoSSe, and $MoSe_2$ calculated by DFT are -3.66 eV, -3.91 eV, and -3.85 eV, respectively (Supplementary Fig. 26), indicating the stronger metal-support interaction between Rh atom and MoSSe compared to both MoS_2 and $MoSe_2$. Therefore, the mass loading of Rh species on MoS_xSe_{2-x} substrates is in the order of MoSSe > $MoS_{0.5}Se_{1.5}$ ≈ $MoS_{0.5}Se_{1.5}$ > $MoSe_2$ > MoS_2."

Comment: 2) *The authors present adsorption Gibbs free energies of OH and H, which are certainly important descriptors in alkaline HER. Nevertheless, the crucial step of water dissociation (*$H_2O \rightarrow H^* + OH^*$*) is not explicitly addressed. Additional calculations showing how the S/Se ratio influences*

the water dissociation barrier and the preferred active site for this step would significantly strengthen the mechanistic claims.

Response: Thanks for the valuable comment. We have added the calculation about water dissociation barrier at all possible and thermodynamically stable adsorption sites on both pristine MoS_xSe_{2-x} and Rh_{SA}-MoS_xSe_{2-x} (Fig. R3-5). As show in Fig. R6, the water dissociation free energy at Rh sites of Rh_{SA}-MoS_xSe_{2-x} exhibits a volcano-type relationship with the S: Se ratio, with the lowest energy barrier observed at an S: Se ratio of 1:1 (volcano's apex). It is worth nothing that the water dissociation energy at Rh sites can be effectively modulated by tuning the anion composition in the support, synergistically improving both the thermodynamics and kinetics of HER. Moreover, we also calculated the Gibbs free energy differences for the alkaline HER process in Rh_{SA}-MoS_xSe_{2-x} and MoS_xSe_{2-x}. The H_2O adsorption at Rh sites in Rh_{SA}-MoS_xSe_{2-x} is thermodynamically barrier-free and more favorable than at S/Se sites in Rh_{SA}-MoS_xSe_{2-x} or MoS_xSe_{2-x} (Fig. R7). The rate-determining step (RDS) for alkaline HER in both systems is H_2O dissociation. Significantly, the incorporation of Rh single-atom in Rh_{SA}-MoS_xSe_{2-x} lowers the energy barriers for H_2O dissociation, synergistically improving HER thermodynamics and kinetics.

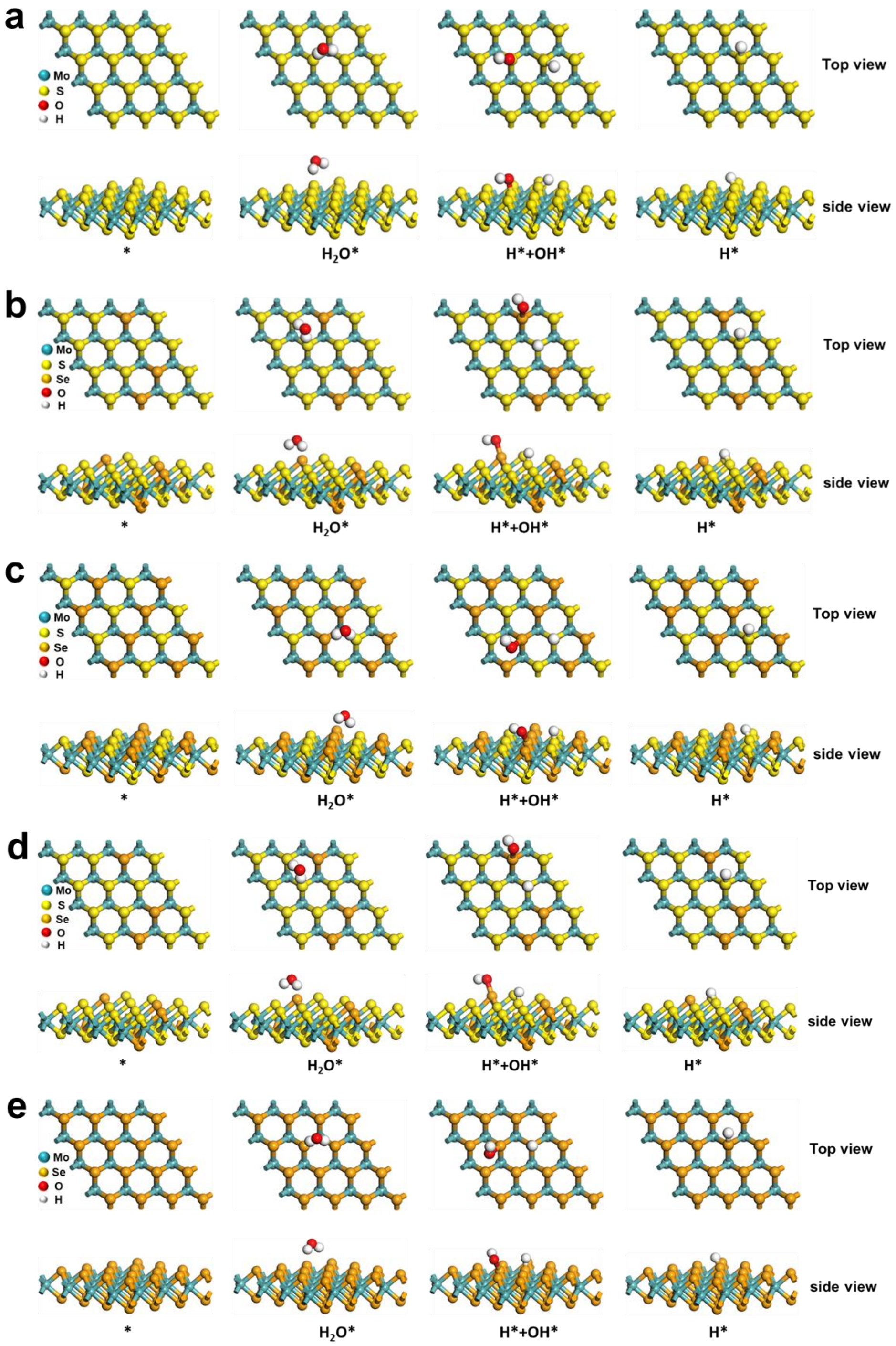

Fig. R3 (Supplementary Figs. 8-12). Diagram illustrating the HER at S/Se sites on (a) MoS_2, (b) $MoS_{1.5}Se_{0.5}$, (c) MoSSe, (d) $MoS_{0.5}Se_{1.5}$, and (e) $MoSe_2$.

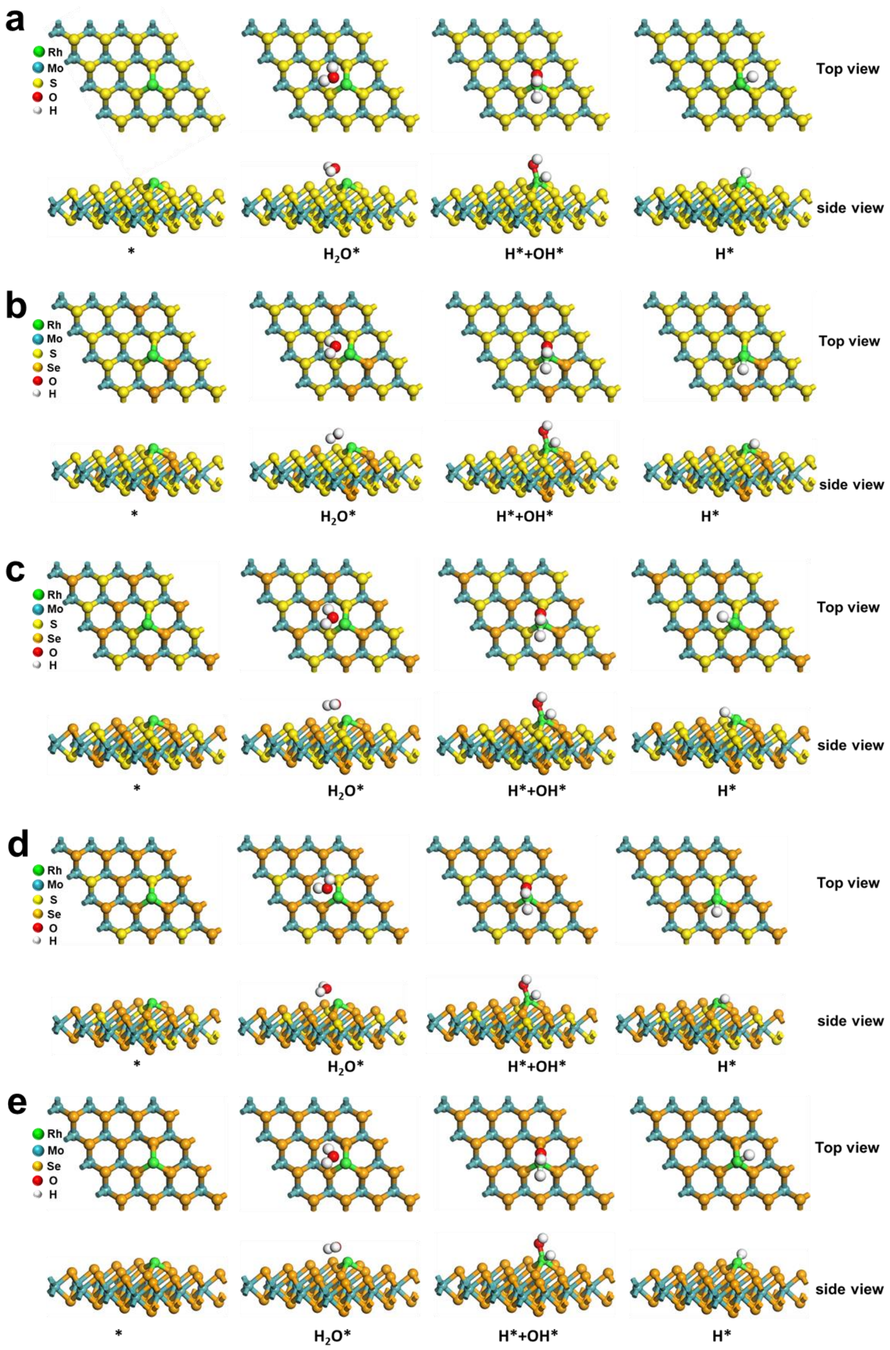

Fig. R4 (Supplementary Figs. 13-17). Diagram illustrating the HER at Rh sites on (a) Rh_{SA}-MoS_2, (b) Rh_{SA}-$MoS_{1.5}Se_{0.5}$, (c) Rh_{SA}-MoSSe, (d) Rh_{SA}-$MoS_{0.5}Se_{1.5}$, and (e) Rh_{SA}-$MoSe_2$.

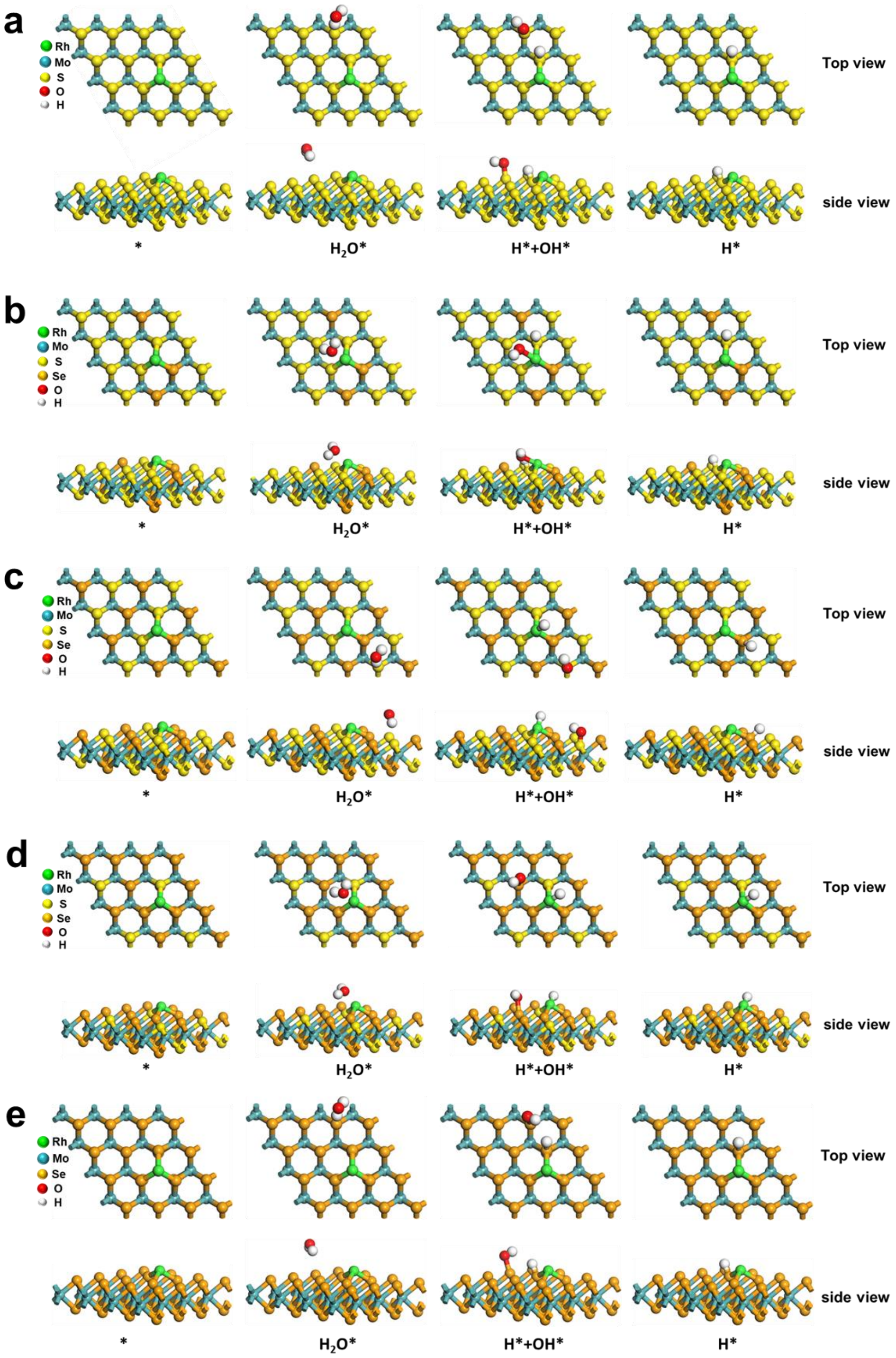

Fig. R5 (Supplementary Figs. 18-22). Diagram illustrating the HER at S/Se sites surrounding Rh single-atom on (a) Rh_{SA}-MoS_2, (b) Rh_{SA}-$MoS_{1.5}Se_{0.5}$, (c) Rh_{SA}-MoSSe, (d) Rh_{SA}-$MoS_{0.5}Se_{1.5}$, and (e) Rh_{SA}-$MoSe_2$.

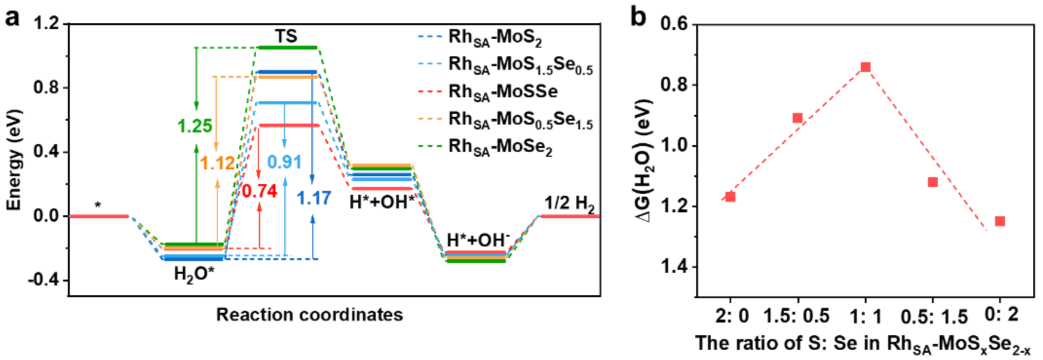

Fig. R6 (Fig. 1e). (a) Gibbs free energy diagram for the HER process on Rh_{SA}-MoS_xSe_{2-x} at Rh sites. (b) Correlation between the S:Se ratio in Rh_{SA}-MoS_xSe_{2-x} and water dissociation free energy at Rh sites.

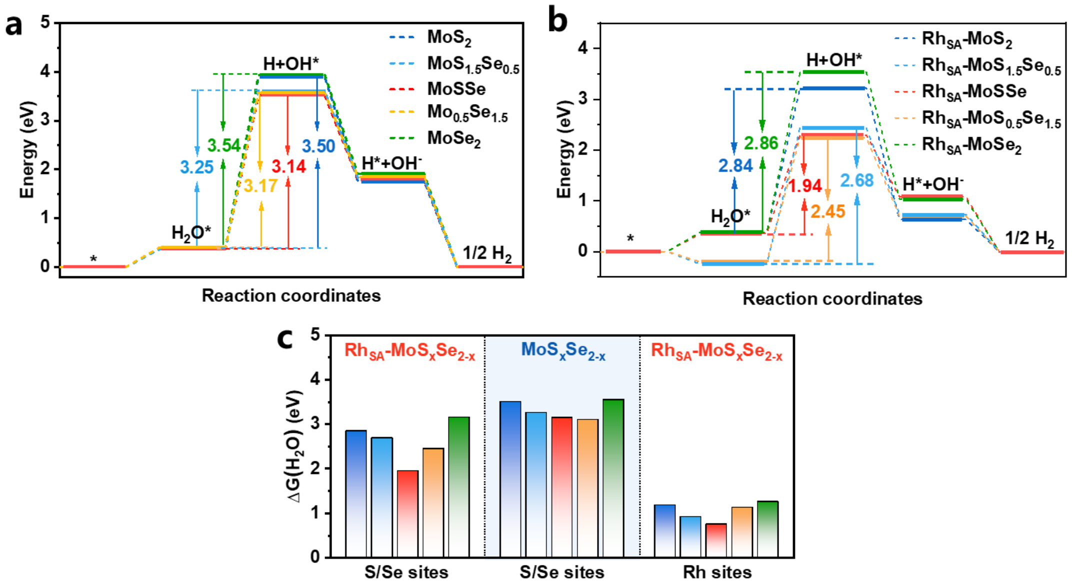

Fig. R7 (Supplementary Fig. 23). Gibbs free energy diagram for the HER process on (a) MoS_xSe_{2-x} at S/Se sites, (b) Rh_{SA}-MoS_xSe_{2-x} at S/Se sites surrounding Rh single-atom. (c) Water dissociation energies at S/Se sites in pristine MoS_xSe_{2-x}, Rh sites, and adjacent S/Se sites in Rh_{SA}-MoS_xSe_{2-x}.

We have also added these contents to the revised manuscript highlighted in yellow in Line 14-21, Page 7, "Gibbs free energy differences for the alkaline HER process in Rh_{SA}-MoS_xSe_{2-x} and MoS_xSe_{2-x} were calculated in Supplementary Figs. 8-23. H_2O adsorption at Rh sites in Rh_{SA}-MoS_xSe_{2-x} is thermodynamically barrier-free and more favorable than at S/Se sites in Rh_{SA}-MoS_xSe_{2-x} or MoS_xSe_{2-x}. The rate-determining step (RDS) for alkaline HER in both systems is H_2O dissociation. Moreover, the water dissociation free energy of Rh_{SA}-MoS_xSe_{2-x} exhibits a volcano-type relationship with the S: Se ratio, with the lowest energy barrier observed at an S: Se ratio of 1: 1 (volcano's apex). Significantly, the incorporation of Rh single-atom in Rh_{SA}-MoS_xSe_{2-x} lowers the energy barriers for H_2O dissociation,

effectively promoting the water dissociation."

Comment: 3) *XAFS is indeed a powerful tool to probe local atomic coordination. However, the current EXAFS results are only qualitatively described. Detailed EXAFS fitting data, including coordination numbers, bond distances, and Debye–Waller factors, should be provided along with fitting tables to confirm the proposed Rh–S/Se coordination environment.*

Response: Thank you very much for the valuable suggestion. We have added the detailed EXAFS fitting data of Rh_{SA}-MoS_2, Rh_{SA}-MoSSe, and Rh_{SA}-$MoSe_2$, including coordination numbers (CN), bond distances (R), bond distances shift (ΔR), Debye-Waller factors ($\sigma^{\wedge 2}$), and R-factor to unambiguously confirm the proposed Rh-S/Se coordination environment. The Fourier transformed extended X-ray absorption fine spectroscopy (FT-EXAFS) spectra of Rh K-edge in Rh_{SA}-MoS_2, Rh_{SA}-MoSSe, and Rh_{SA}-$MoSe_2$ all display a main characteristic peak below 3 Å, matching to the Rh-S/Se coordination (*Nat. Commun.* **14**, 6019 (2023); *Adv. Mater.* **33**, 2105049 (2021)). In contrast to the Rh foil reference, the absence of a metallic Rh-Rh scattering signal rules out the presence of Rh nanoparticles or clusters, confirming the atomic dispersion of Rh species in Rh_{SA}-MoS_2, Rh_{SA}-MoSSe, and Rh_{SA}-$MoSe_2$. The Ru K-edge EXAFS data fitting curves in Fig. R8 and corresponding fitting data in Table R1 unveiled the mean Ru-S/Se coordination number of the three catalysts was about 3.5, which was consistent with theoretical calculation models. Given the higher ionic radius and relative atomic mass of Se^{2-} compared to S^{2-} (*Mater. Res. Bull.* **37**, 1923–1931 (2002); *Mater. Today Commun.* **28**, 102614 (2021); *J. Phys. Chem. Solids* **194**, 112222 (2024)), the bond distance of Rh-S (2.3 Å) is shorter than Rh-Se (2.5 Å).

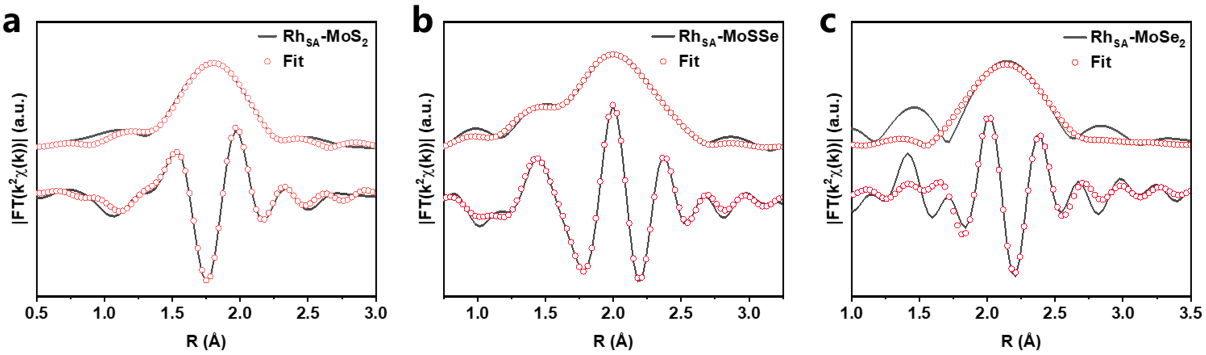

Fig. R8 (Supplementary Fig. 34). The Rh K-edge EXAFS fitting (red dots) for the Rh_{SA}-MoS_2 (a), Rh_{SA}-MoSSe (b), and Rh_{SA}-$MoSe_2$ (c), shown in k^2 weighted R-space.

Table R1 (Supplementary Table 1). The best-fitted EXAFS results of Rh_{SA}-MoS_2, Rh_{SA}-MoSSe, and

Rh_{SA}-$MoSe_2$.

Sample	Shell	CN	R (Å)	Δ R (Å)	$σ^{\wedge 2}$ (10^{-2} $Å^2$)	R-factor (%)
Rh_{SA}-MoS_2	Rh-S	3.5	2.32	-0.06	0.7	1.1
Rh_{SA}-MoSSe	Rh-S	1.9	2.24	-0.12	1.3	0.3
Rh_{SA}-MoSSe	Rh-Se	1.6	2.51	-0.05	0.4	0.4
Rh_{SA}-$MoSe_2$	Rh-Se	3.4	2.53	0.03	0.8	1.9

In the revised manuscript, we have included the detailed XAFS analysis in the main text highlighted in yellow in Line 19-23, Page 10 and Line 1-3, Page 11. "The Rh K-edge EXAFS data fitting curves in Supplementary Fig. 34 and corresponding fitting data in Supplementary Table 1 unveiled the mean Ru-S/Se coordination number of the three catalysts was about 3.5, which was consistent with theoretical calculation models. Given the higher ionic radius and relative atomic mass of Se^{2-} compared to S^{2-}[41,42,45], the bond distance of Rh-S (2.3 Å) is shorter than Rh-Se (2.5 Å). Furthermore, Wavelet transformed EXAFS (WT-EXAFS) contour plots reveal single intensity maxima for Rh in Rh_{SA}-MoS_2, Rh_{SA}-MoSSe, and Rh_{SA}-$MoSe_2$ at approximately 6.7, 8.0, and 9.4 $Å^{-1}$ (Fig. 2h), corresponding to the Rh-S, Rh-S/Se, and Rh-Se coordination paths[34], respectively."

Comment: 4) *While the manuscript provides extensive XPS characterization of S/Se ratio effects, the XAFS analysis is insufficient. Given that XAFS directly reveals the coordination environment of single atoms, the dataset should be expanded. This would allow a more convincing correlation between structural evolution and catalytic performance.*

Response: Thank you for the kind suggestion. In the revised manuscript, we have included the detailed XAFS analysis in the main text highlighted in yellow in Line 14-23, Page 10 and Line 1-3, Page 11. "The Fourier transformed extended X-ray absorption fine spectroscopy (FT-EXAFS) spectra of Rh K-edge in Rh_{SA}-MoS_2, Rh_{SA}-MoSSe, and Rh_{SA}-$MoSe_2$ all display a main characteristic peak below 3 Å (Fig. 2g), matching to the Rh-S/Se coordination[22,33]. In contrast to the Rh foil reference, the absence of a metallic Rh-Rh scattering signal rules out the presence of Rh nanoparticles or clusters, confirming the atomic dispersion of Rh species in Rh_{SA}-MoS_2, Rh_{SA}-MoSSe, and Rh_{SA}-$MoSe_2$. The Rh K-edge EXAFS data fitting curves in Supplementary Fig. 34 and corresponding fitting data in Supplementary Table 1 unveiled the mean Ru-S/Se coordination number of the three catalysts was about 3.5, which was consistent with theoretical calculation models. Given the higher ionic radius and relative atomic

mass of Se^{2-} compared to S^{2-}[41,42,45], the bond distance of Rh-S (2.3 Å) is shorter than Rh-Se (2.5 Å). Furthermore, Wavelet transformed EXAFS (WT-EXAFS) contour plots reveal single intensity maxima for Rh in Rh_{SA}-MoS_2, Rh_{SA}-MoSSe, and Rh_{SA}-$MoSe_2$ at approximately 6.7, 8.0, and 9.4 $Å^{-1}$ (Fig. 2h), corresponding to the Rh-S, Rh-S/Se, and Rh-Se coordination paths[34], respectively."

Comment: 5) *To demonstrate practical relevance, it is strongly recommended to evaluate the catalyst under realistic device conditions. Specifically, membrane electrode assembly (MEA) or anion exchange membrane water electrolyzer (AEMWE) tests should be provided to validate whether the catalyst can maintain its activity and durability in full-cell configurations at technologically relevant current densities.*

Response: Thank you very much for the valuable comment. We have added anion exchange membrane water electrolyzer (AEMWE) tests to further evaluate the application potential of Rh_{SA}-MoSSe catalyst for industrial water splitting. The homemade NiFe LDH@NF as an anode electrocatalyst (Fig. R9) was assembled with Rh_{SA}-MoSSe and operated in 1.0 M KOH electrolyte under 80 °C. The polarization curves showed the Rh_{SA}-MoSSe||NiFe LDH@NF exhibit a cell voltage of 1.58 V and 1.70 V at current densities of 0.5 A cm^{-2} and 1.0 A cm^{-2} for AEMWE, respectively, which is superior to commercial Pt/C||NiFe LDH@NF and MoSSe||NiFe LDH@NF systems (Fig. R10a). Furthermore, the AEMWE of Rh_{SA}-MoSSe||NiFe LDH@NF was continuously operated for over 500 h at a current density of 1 A cm^{-2} (Fig. R10b). The overpotential at 1.0 A cm^{-2} and stability time of were comparable to those of recently reported catalysts for AEMWE (Table R2). The above results confirm the advanced and reliable potential of Rh_{SA}-MoSSe catalyst for industrial water splitting applications.

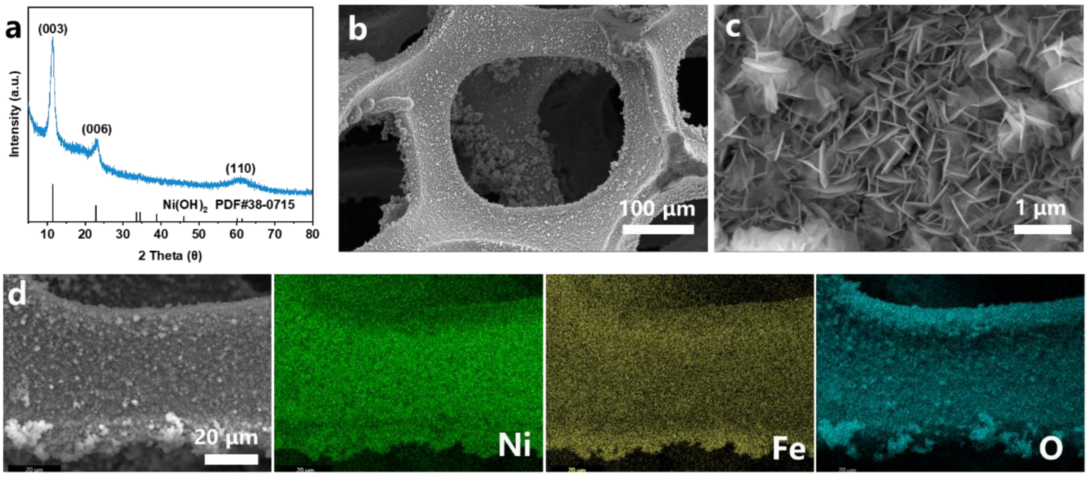

Fig. R9 (Supplementary Fig. 53). (a) XRD patterns of NiFe LDH@NF. SEM images (b and c) and corresponding EDS elemental mapping (d) of NiFe LDH@NF.

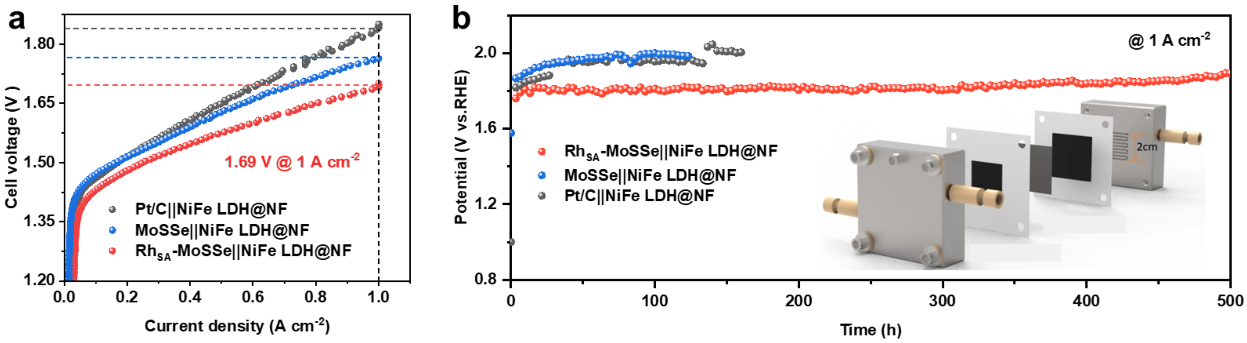

Fig. R10 (Figs. 4g and h). (a) Polarization curve of Rh$_{SA}$-MoSSe||NiFe LDH@NF, MoSSe||NiFe LDH@NF, and Pt/C||NiFe LDH@NF in the AEMWE operated at 80 °C. (b) Stability test of Rh$_{SA}$-MoSSe||NiFe LDH@NF, MoSSe||NiFe LDH@NF, and Pt/C||NiFe LDH@NF in the AEMWE at 1 A·cm^{-2}. Inset: schematic diagram of an AEMWE.

Table R2 (Supplementary Table 4). Comparison of cell voltage at a current density of 1 A cm^{-2} and stability time for recently reported catalysts applied in AEMWE.

Catalysts	electrolyte	Cell Voltage (V) @j = 1 A cm^{-2}	Stability Time	Ref.
Rh$_{SA}$-MoSSe‖NiFe LDH@NF	1 M KOH	1.70	500 h@1 A cm^{-2}	This work
Pd, Ru, Rh, Pt, Re-MoSe$_2$‖IrO$_2$	1 M KOH	1.82	500 h@1 A cm^{-2}	12
Ru SAs/WC$_x$‖NiFeOH$_x$-NF	1 M KOH	1.79	190 h@1 A cm^{-2}	19
Ru-WC$_{1-x}$‖NiFeO$_x$	1 M KOH	1.80	100 h@1 A cm^{-2}	20
Ru-YNC‖NiFe LDH	1 M KOH	1.87	1000 h@0.5 A cm^{-2}	22
Ru-LC-Ni(OH)$_2$‖FeNi LDH	1 M KOH	1.69	500 h@0.5 A cm^{-2}	26
(Rh, O)-MoSe$_{2-x}$‖IrO$_2$	1 M KOH	No.	300 h@1.5 A cm^{-2}	29
1T p$_z$-MoS$_2$‖RuIrO$_2$	1 M KOH	1.78	200 h@0.5 A cm^{-2}	30
FeCo-MoS$_2$	0.5 M H$_2$SO$_4$	2.23	350 h@1 A cm^{-2}	31
Pt$_1$/Ni(OH)$_2$‖Ir$_1$/Co(OH)$_2$	1 M KOH	2.10	1030 h@0.8 A cm^{-2}	32
Co$_n$-Pt$_1$@NPC‖NiFe LDH	1 M KOH	2.03	100 h@1 A cm^{-2}	33

We have also added these contents to the revised manuscript highlighted in yellow in Line 12-23, Page 18, "The anion exchange membrane water electrolyzer (AEMWE) tests were employed to further evaluate the application potential of Rh$_{SA}$-MoSSe catalyst for industrial water splitting. The homemade NiFe LDH@NF as an anode electrocatalyst (Supplementary Fig. 53) was assembled with Rh$_{SA}$-MoSSe

and operated in 1.0 M KOH electrolyte under 80 °C. The polarization curves showed the Rh_{SA}-MoSSe||NiFe LDH@NF exhibit a cell voltage of 1.58 V and 1.70 V at current densities of 0.5 A cm^{-2} and 1.0 A cm^{-2} for AEMWE, respectively, which is superior to commercial Pt/C||NiFe LDH@NF and MoSSe||NiFe LDH@NF systems (Fig. 4g). Furthermore, the AEMWE of Rh_{SA}-MoSSe||NiFe LDH@NF was continuously operated for over 500 h at a current density of 1 A cm^{-2} (Fig. 4h). The overpotential at 1.0 A cm^{-2} and stability time of were comparable to those of recently reported catalysts for AEMWE (Supplementary Table 4). The above results confirm the advanced and reliable potential of Rh_{SA}-MoSSe catalyst for industrial water splitting applications."

We have also added these contents to the revised supporting information highlighted in yellow in Line 12-18, Page 3, "**2.4 Synthesis of NiFe LDH@NF**. The NiFe LDH grown on Ni foam was synthesized through a hydrothermal reaction. Firstly, the solution was prepared by adding 0.5 mmol $Ni(NO_3)_2·6H_2O$, 0.5 mmol $Fe(NO_3)_3·9H_2O$, and 5 mmol $CO(NH_2)_2$ into 30 mL of water and transferred to an autoclave. Subsequently, the cleaned Ni foam was placed into the obtained solution. The hydrothermal reaction was maintained at 80 °C for 4 h. After cooling naturally to room temperature, the sample was washed with ethanol and ultrapure water several times and finally dried in air for further use.", and in Line 20-23, Page 4 and Line 1-7, Page 5, "**4. Anion-exchange membrane water electrolyzer (AEMWE) test.** Anion-exchange membrane water electrolyzer (AEMWE) with serpentine flow channel were used to test practical applications of Rh_{SA}-MoSSe electrocatalysts. The anode electrode was prepared by spraying Rh_{SA}-MoSSe or Pt/C inks on Ni foam, the spraying was controlled to achieve a mass loading of 4.0 mg cm^{-2}. The homemade self-supporting NiFe LDH@NF was used as an anode electrocatalyst. Ni foam was used as a cathode porous current collector. Sustainion®X37-50 Grade 60 anion exchange membrane (Dioxide Materials Corporation) was activated by soaking in a 1.0 M KOH solution for 24 hours under 80 °C before use. The AEMWE test was operated at 80 °C with a peristaltic pump pumping 1.0 M KOH at a flow rate of 40 ml·min^{-1}.

Polarization curves were obtained from 1 to 1.8 V at a stepwise rate of 5 mV s^{-1}. A long-term stability test was conducted at a current density of 1 $A\cdot cm^{-2}$ at 80 °C."

Comment: 6) *The manuscript also contains typographical errors that should be carefully corrected. (Fig. 5g).*

Response: Thanks for the valuable comment. Thanks for the valuable comment. In the revised manuscript, we have corrected the sample names in Figure 5g.

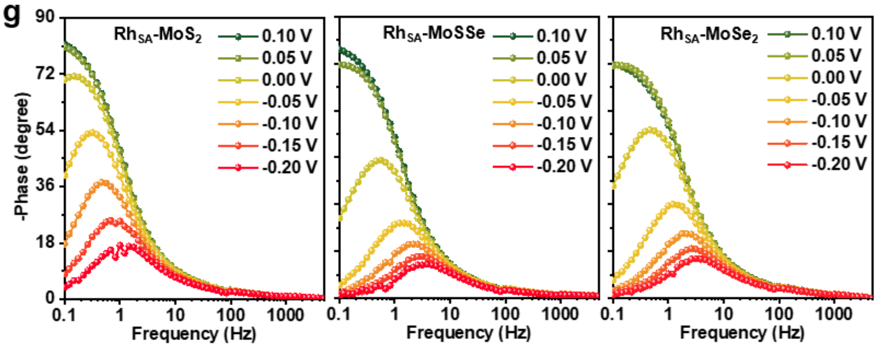

Fig 5 (g) In situ bode plots for Rh_{SA}-MoS_2, Rh_{SA}-MoSSe, and Rh_{SA}-$MoSe_2$.

Reviewer #2 (Remarks to the Author)

This manuscript presents a comprehensive study on rhodium single-atom catalysts supported on molybdenum sulfide selenide (MoS_xSe_{2-x}) for hydrogen evolution reactions. The study addresses the precise modulation of metal-support interactions at the orbital level and their impact on catalytic performance. The authors employ a systematic approach by varying the support composition and correlating these electronic effects with HER activity, stability, and intermediate binding energies. However, for practical relevance and commercialization, electrochemical catalysts should ideally be evaluated at the single-cell level. Therefore, the reviewer recommends major revisions, including the addition of AEMWE data, before the manuscript is suitable for publication in this journal.

Response: Thank you very much for the recommendation of our manuscript for publication, which greatly helps us to improve our manuscript. In the revised manuscript and supporting information, we have updated the important characterizations as suggested by the Reviewer. Please see below our point-by-point responses to the Reviewer's concerns.

This reviewer raises the following questions:

Comment: 1) *The authors claim that the d-band center of Rh in the Rh_{SA}-MoS_xSe_{2-x} series shows a volcano-type dependence with ΔGH* and ΔGOH*. However, according to conventional d-band theory, adsorption energies usually scale linearly with the d-band position, where an upshifted d-band leads to stronger adsorption. In Figure 1c, the center shift due to d-p hybridization is thought to follow a linear trend. This may be considered as a discrepancy between Figures c and d, requiring further explanation for the origin of the volcano-like behavior. The discussion would be further strengthened if a mechanistic basis linking the adsorption energy and the observed volcano-like trend was presented.*

Response: Thank you very much for the valuable and insightful comments. As you pointed out, the volcano-type relationship between the d-band center of Rh in the Rh_{SA}-MoS_xSe_{2-x} series and both ΔG_{H*} and ΔG_{OH*} cannot be fully explained by the conventional d-band theory. Therefore, we have performed additional analysis and significantly expanded our discussion to provide a clear mechanistic basis linking the adsorption energy and the observed volcano-like trend. Up to now, the d-band center theory is widely adopted for metal-based catalysts with continuous energy band structures (*Angew. Chem. Int. Ed.*, **62**, e202310894 (2023); *Adv. Mater.* **35**, 2208034 (2023); *Angew. Chem. Int. Ed.* **62**, e202307808 (2023)). However, the character of discrete energy structures of SACs raises questions about the d-

band theory (*Phys. Rev. Lett.* **76**, 2141-2144 (1996); *J. Am. Chem. Soc.* **147**, 7070−7082 (2025)). Alternatively, frontier molecular orbital (FMO) theory (*Phys. Rev. Lett.* **125**, 156001 (2020); *J. Am. Chem. Soc.* **143**, 11897–11902 (2021); *Nature* **640**, 668–675 (2025)), frequently applied to homogeneous molecular catalysts, has also been advocated to depict M_1-adsorbate orbital interactions in SACs. Therefore, the FMO theory was presented to link the adsorption energy and the observed volcano-like trend. To gain a comprehensive understanding of metal-support frontier orbital interactions, we construct a model system of Rh single-atom catalysts (SACs), in which the highest occupied molecular orbital (HOMO) of the Rh single atoms (Rh_{SA}) can be effectively modulated by tuning the anion composition in the support (Fig. R1). As shown in Fig. R2a, the conversion from energy relative to the Fermi level (E-E_f) to energy relative to the vacuum level (E-E_{vac}) is a fundamental transformation in computational chemistry and materials science that establishes absolute energy references for molecular orbitals. This conversion is governed by the work function (Φ), which represents the minimum energy required to remove an electron from the Fermi level to the vacuum level, mathematically expressed as $\Phi = E_{vac} - E_f$. The fundamental conversion equation is $E - E_{vac} = (E - E_f) - \Phi$, where the work function serves as the critical bridge between these two energy reference systems.

The first principal density functional theory (DFT) calculations indicate that the lowest unoccupied molecular orbital (LUMO) position of MoS_xSe_{2-x} substrates exhibits a volcano-type relationship with the S: Se ratio, with MoSSe located at the volcano apex (Fig. R2). Moreover, the elevated LUMO of MoSSe support narrows the energy gap with the HOMO of Rh atoms, which promotes orbital hybridizations between Rh atoms and support to lead to a high stability. When electrons are transferred from Rh to MoS_xSe_{2-x} substrates, the anchored Rh atoms exhibit an unoccupied or partially occupied state (or LUMO) in Rh_{SA}-MoS_xSe_{2-x}. Therefore, regulating Rh-MoS_xSe_{2-x} orbital coupling would facilitate charge transfer and effectively modulated the LUMO position of anchored Rh atoms. As shown in Fig. R3, the projected density of states (PDOS) analysis demonstrates the LUMO position of Rh atoms exhibits a volcano-type relationship with the S: Se ratio in MoS_xSe_{2-x} substrates. The higher LUMO of Rh atoms on MoSSe exhibits smaller energy gaps (ΔE_1) with the HOMO of OH^- and larger energy gaps (ΔE_2) with the HOMO of H_2 molecules, which could directly alter the adsorption energies of key intermediates through systematic tuning the S: Se ratio in the support. Moreover, the S: Se ratio in Rh_{SA}-MoS_xSe_{2-x} series exhibits a volcano-type relationship with both the free energy of hydrogen

adsorption (ΔG_{H*}) and hydroxide adsorption (ΔG_{OH*}), with Rh_{SA}-MoSSe located at the volcano apex, displaying optimal adsorption strengths for both species (H and OH).

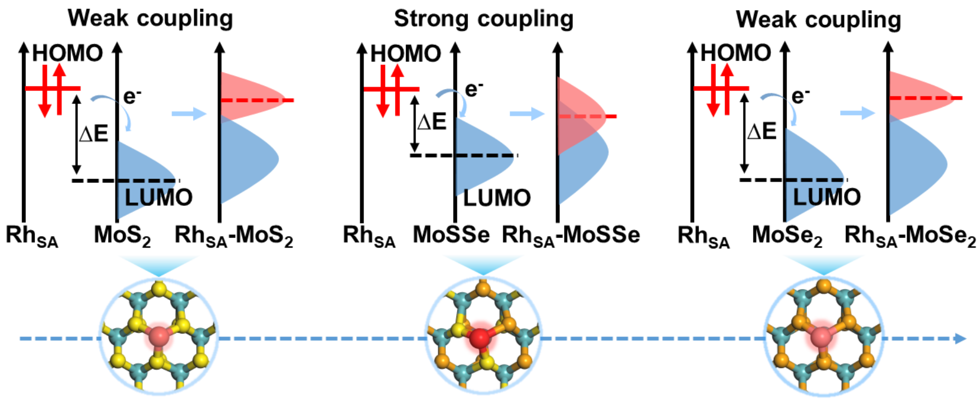

Fig. R1 (Fig. 1a). Schematic illustration of modulating MSIs in Rh_{SA}-MoS_xSe_{2-x} by varying the S: Se ratio of MoS_xSe_{2-x} supports.

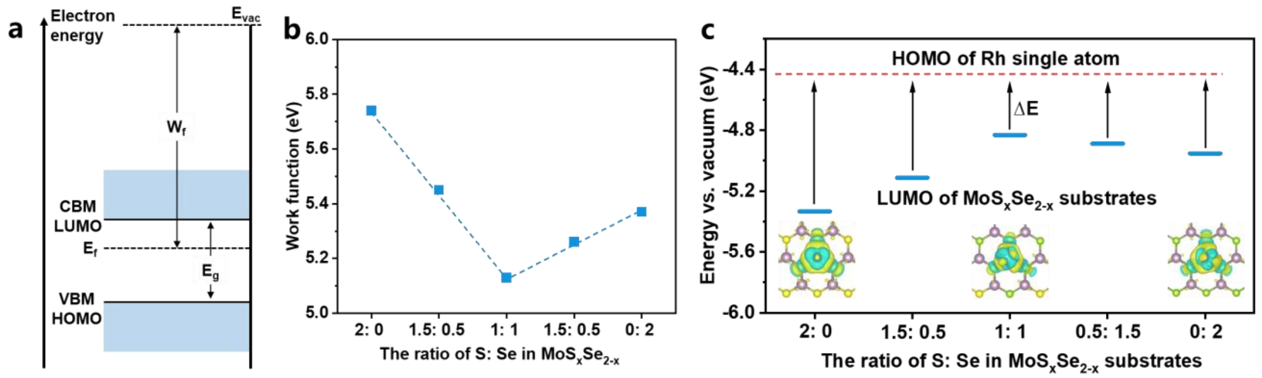

Fig. R2 (Supplementary Fig. 4 and Fig. 1b). (a) Energy diagram of a semiconductor with flat bands to the surface. Band edges (CBM/LUMO and VBM/HOMO), vacuum level E_{vac}, work function W_f, and energy gap E_g are defined. (b) Dependence of the calculated work function on the S: Se ratio in MoS_xSe_{2-x}. (c) Calculated LUMO positions of MoS_xSe_{2-x} substrates with different S: Se ratios and the HOMO of unsupported Rh atom relative to vacuum. Black arrows highlight the energy discrepancies (ΔE) between the MoS_xSe_{2-x} LUMOs and the Rh atom HOMO. The insets show the charge density difference of Rh atoms in Rh_{SA}-MoS_2, Rh_{SA}-MoSSe, and Rh_{SA}-$MoSe_2$, with yellow and blue regions indicating charge accumulation and decrease, respectively.

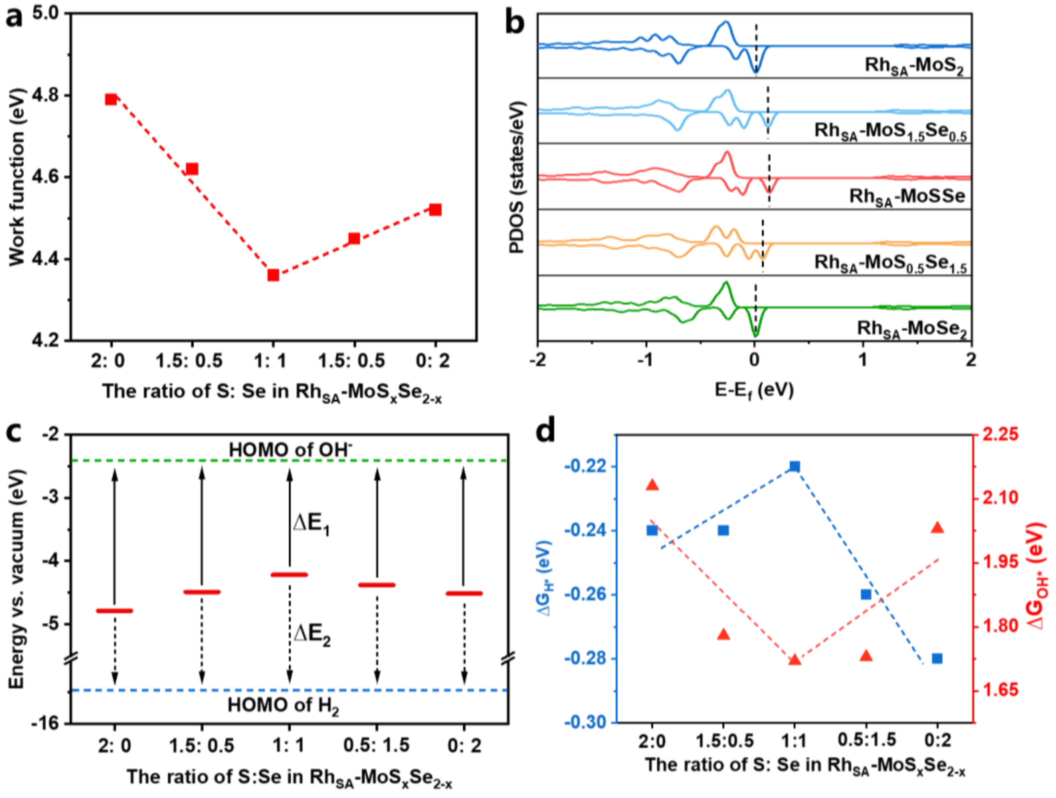

Fig. R3 (Supplementary Fig. 4, Fig. 1c and d). (a) Dependence of the calculated work function on the S: Se ratio in Rh_{SA}-MoS_xSe_{2-x}. (b) Calculated PDOS of anchored Rh atoms in Rh_{SA}-MoS_xSe_{2-x}, where bleak dashed lines denote the LUMO of Rh. (c) Calculated LUMO positions of Rh single atoms anchored on MoS_xSe_{2-x} substrates (red solid line) and the HOMO of OH^- (green dashed line) and H_2 (blue dashed line) relative to vacuum. Black arrows highlight the energy discrepancies (ΔE_1) between the Rh_{SA} LUMOs and the OH^- HOMO. Black dashed arrows highlight the energy discrepancies (ΔE_2) between the Rh_{SA} LUMOs and the H_2 HOMO. (d) Correlation between the S: Se ratio and ΔG_{OH^*}, and ΔG_{H^*} in Rh_{SA}-MoS_xSe_{2-x}.

We have also added these contents to the revised manuscript highlighted in yellow in Line 7-23, Page 5 and Line 5-9, Page 7, "The calculated HOMO position of freestanding Rh atom is located at -4.50 eV, whereas the LUMO of MoS_xSe_{2-x} substrates exhibit a volcano-type relationship with the S: Se ratio, with MoSSe located at the volcano apex (Fig. 1b, Supplementary Fig. 4, and Supplementary Note 1). According to the FMO theory[32–34], the elevated LUMO of MoSSe support narrows the energy gap with the highest occupied molecular orbital (HOMO) of Rh atoms, which promotes orbital

hybridizations between Rh atoms and support to lead to a high stability. Charge density difference analyses reveal distinct charge redistribution between Rh single-atoms and coordinated S/Se atoms, resulting in a marked reduction of electron density around Rh atoms (Fig. 1b (inset)).

When electrons are transferred from Rh to MoS_xSe_{2-x} substrates, the anchored Rh atoms exhibit an unoccupied or partially occupied state (or LUMO) in Rh_{SA}-MoS_xSe_{2-x}. Therefore, regulating Rh-MoS_xSe_{2-x} orbital coupling would facilitate charge transfer and effectively modulated the LUMO position of anchored Rh atoms. As shown in Supplementary Fig. 4c, the projected density of states (PDOS) analysis demonstrates the LUMO position of Rh atoms exhibits a volcano-type relationship with the S: Se ratio in MoS_xSe_{2-x} substrates. The higher LUMO of Rh atoms on MoSSe exhibits smaller energy gaps (ΔE_1) with the HOMO of OH^- and larger energy gaps (ΔE_2) with the HOMO of H_2 molecules (Fig. 1c), which could directly alter the adsorption energies of key intermediates through systematic tuning the S: Se ratio in the support. Moreover, the S: Se ratio in Rh_{SA}-MoS_xSe_{2-x} series exhibits a volcano-type relationship with both the free energy of hydrogen adsorption (ΔG_{H*}) and hydroxide adsorption (ΔG_{OH*}), with Rh_{SA}-MoSSe located at the volcano apex, displaying optimal adsorption strengths for both species (H and OH) (Fig. 1d and Supplementary Figs. 5 and 6).".

Comment: 2) *In Figure 1e, the authors claim that the integration of Rh sites into molybdenum sulfide selenide substantially enhances H and OH adsorption. It would strengthen the manuscript to provide a more detailed discussion on how the ΔGH* and ΔGOH* values are improved, specifically whether these values fall within the optimal range for HER activity for each sample. Including such analysis would help clarify the correlation between the observed adsorption enhancements and the expected catalytic performance.*

Response: Thank you for the comment. The frontier molecular orbital (FMO) theory, frequently applied to homogeneous molecular catalysts, has also been advocated to depict M_1-adsorbate orbital interactions in SACs. Therefore, the FMO theory was presented to link the adsorption energy and the observed volcano-like trend. To gain a comprehensive understanding of metal-support frontier orbital interactions, we construct a model system of Rh single-atom catalysts (SACs), in which the highest occupied molecular orbital (HOMO) of the Rh single atoms (Rh_{SA}) can be effectively modulated by tuning the anion composition in the support (Fig. R4). When electrons are transferred from Rh to

MoS_xSe_{2-x} substrates, the anchored Rh atoms exhibit an unoccupied or partially occupied state (or LUMO) in Rh_{SA}-MoS_xSe_{2-x}. Therefore, regulating Rh-MoS_xSe_{2-x} orbital coupling would facilitate charge transfer and effectively modulated the LUMO position of anchored Rh atoms. As shown in Fig. R5, the projected density of states (PDOS) analysis demonstrates the LUMO position of Rh atoms exhibits a volcano-type relationship with the S: Se ratio in MoS_xSe_{2-x} substrates. The higher LUMO of Rh atoms on MoSSe exhibits smaller energy gaps (ΔE_1) with the HOMO of OH^- and larger energy gaps (ΔE_2) with the HOMO of H_2 molecules, which could directly alter the adsorption energies of key intermediates through systematic tuning the S: Se ratio in the support. Moreover, the S: Se ratio in Rh_{SA}-MoS_xSe_{2-x} series exhibits a volcano-type relationship with both the free energy of hydrogen adsorption (ΔG_{H*}) and hydroxide adsorption (ΔG_{OH*}), with Rh_{SA}-MoSSe located at the volcano apex, displaying optimal adsorption strengths for both species (H and OH).

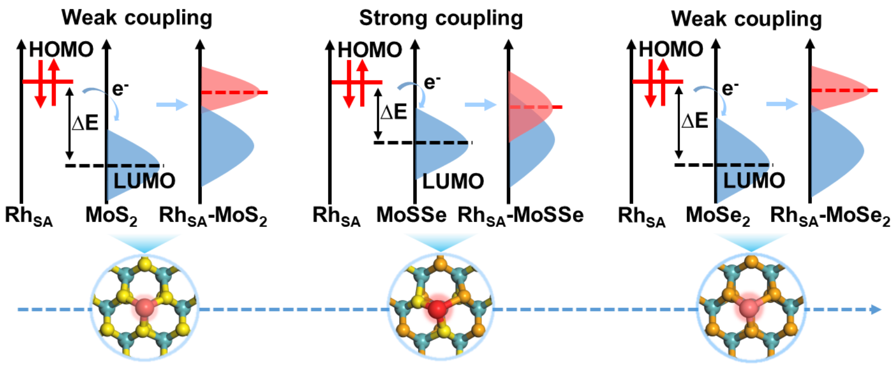

Fig. R4 (Fig. 1a). Schematic illustration of modulating MSIs in Rh_{SA}-MoS_xSe_{2-x} by varying the S: Se ratio of MoS_xSe_{2-x} supports.

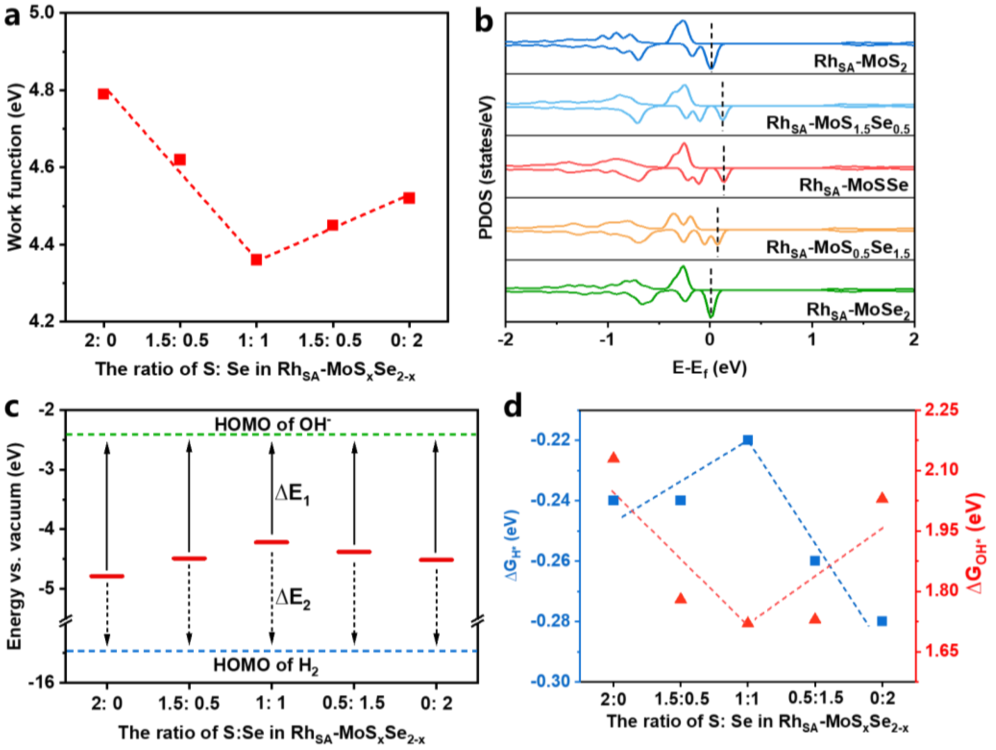

Fig. R5 (Supplementary Fig. 4, Fig. 1c and d). (a) Dependence of the calculated work function on the S: Se ratio in Rh$_{SA}$-MoS$_x$Se$_{2-x}$. (b) Calculated PDOS of anchored Rh atoms in Rh$_{SA}$-MoS$_x$Se$_{2-x}$, where bleak dashed lines denote the LUMO of Rh. (c) Calculated LUMO positions of Rh single atoms anchored on MoS$_x$Se$_{2-x}$ substrates (red solid line) and the HOMO of OH$^-$ (green dashed line) and H$_2$ (blue dashed line) relative to vacuum. Black arrows highlight the energy discrepancies (ΔE_1) between the Rh$_{SA}$ LUMOs and the OH$^-$ HOMO. Black dashed arrows highlight the energy discrepancies (ΔE_2) between the Rh$_{SA}$ LUMOs and the H$_2$ HOMO. (d) Correlation between the S: Se ratio and ΔG_{OH^*}, and ΔG_{H^*} in Rh$_{SA}$-MoS$_x$Se$_{2-x}$.

We have also added these contents to the revised manuscript highlighted in yellow in Line 16-23, Page 5 and Line 5-9, Page 7, "When electrons are transferred from Rh to MoS$_x$Se$_{2-x}$ substrates, the anchored Rh atoms exhibit an unoccupied or partially occupied state (or LUMO) in Rh$_{SA}$-MoS$_x$Se$_{2-x}$. Therefore, regulating Rh-MoS$_x$Se$_{2-x}$ orbital coupling would facilitate charge transfer and effectively modulated the LUMO position of anchored Rh atoms. As shown in Supplementary Fig. 4c, the projected density of states (PDOS) analysis demonstrates the LUMO position of Rh atoms exhibits a volcano-type relationship with the S: Se ratio in MoS$_x$Se$_{2-x}$ substrates. The higher LUMO of Rh atoms on MoSSe exhibits smaller energy gaps (ΔE_1) with the HOMO of OH$^-$ and larger energy gaps (ΔE_2) with the HOMO of H$_2$ molecules (Fig. 1c), which could directly alter the adsorption energies of key

intermediates through systematic tuning the S: Se ratio in the support. Moreover, the S: Se ratio in Rh_{SA}-MoS_xSe_{2-x} series exhibits a volcano-type relationship with both the free energy of hydrogen adsorption (ΔG_{H*}) and hydroxide adsorption (ΔG_{OH*}), with Rh_{SA}-MoSSe located at the volcano apex, displaying optimal adsorption strengths for both species (H and OH) (Fig. 1d and Supplementary Figs. 5 and 6).".

Comment: ***3)*** *The key point of this work is that the support properties are modulated and the electron density of Rh is tuned through S and Se with higher ionic electronegativity. However, despite their similar electronegativities, the reason why changing the ratio of S and Se affects the properties is not clearly explained. The authors should clarify the underlying mechanism, including the differences in Rh–S and Rh–Se bonding interactions and the relative contributions of π- and σ-bonding, to explain how these changes influence the electronic structure and catalytic behavior.*

Response: Thanks for the kind suggestion. While the similar electronegativity of S and Se, changing the S and Se ratio significantly affects the properties of Rh single atoms, with the fundamental mechanism originating from significant differences in polarizability, atomic size, and orbital characteristics. Specifically, Se features a higher polarizability volume than S, along with larger atomic and covalent radii (*Mater. Res. Bull.* **37**, 1923–1931 (2002); *Mater. Today Commun.* **28**, 102614 (2021); *J. Phys. Chem. Solids* **194**, 112222 (2024)). Therefore, the closer size match between S 3p and Rh 4d orbitals and the shorter Rh-S bond facilitates more effective orbital overlap, resulting in strong σ-bonding and weak π-back bonding in Rh-S bond. Moreover, the higher polarizability and more diffuse 4p orbitals of Se enhance its π-acceptor capacity, leading to stronger π-back bonding and weak σ-bonding in Rh-Se bond (Fig. R6). This differential σ/π bonding mechanism directly modulates the Rh d-band electronic structure, optimizing the adsorption strength of key intermediates and thereby controlling both the activity and selectivity of the catalyst.

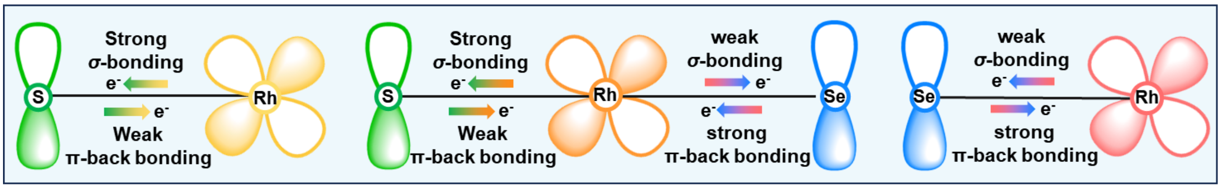

Fig. R6 (Fig. 3a). Schematic depiction of electronic interactions among Rh, S, and Se in Rh_{SA}-MoS_xSe_{2-x}.

We have also added these contents to the revised manuscript highlighted in yellow in Line 12-16, Page 11, "Owing to the higher polarizability volume and larger covalent radii of Se than S[41,42,45], the closer size match between S 3p and Rh 4d orbitals and the shorter Rh-S bond facilitates more effective orbital overlap, resulting in strong σ-bonding and weak π-back bonding in Rh-S bond. Moreover, the higher polarizability and more diffuse 4p orbitals of Se enhance its π-acceptor capacity, leading to stronger π-back bonding and weak σ-bonding in Rh-Se bond."

***Comment:* 4)** *In Figure 3d, it is unclear why the number of active sites differs. If the observed performance difference is attributed to an increase in the number of active sites, the authors should clearly specify the proposed active sites and describe the method used to determine the number of active sites.*

Response: Thanks for the valuable comment. The number of active sites is derived from the electrochemical active surface area (ECSA). For clarity and precision, the descriptor "number of active sites" has been replaced with "electrochemical active surface area" throughout the main text. The observed performance difference is attributed to the increase of ECSA and the enhancement of intrinsic activity. In the main text, the polarization curves of Rh_{SA}-MoS_xSe_{2-x} and MoS_xSe_{2-x} samples, normalized to ECSA, confirm the superior intrinsic activities of Rh_{SA}-MoSSe (Figs. R7a and b). Moreover, the S: Se ratio in Rh_{SA}-MoS_xSe_{2-x} exhibits a volcano-type relationship with both ECSA and intrinsic activity, with Rh_{SA}-MoSSe at the volcano's apex displaying optimal overall HER performance (Fig. R7c). The electrochemical active surface area (ECSA) was determined by measuring the electrochemical double-layer capacitance (C_{dl}). Cyclic voltammetry (CV) was conducted in a non-Faradaic region (0.37 ~ 0.47 V vs. RHE) at scan rates ranging from 20 to 100 mV s^{-1}. The C_{dl} was calculated by plotting the current density difference (ΔJ) between anodic and cathodic sweeps at a fixed potential against the scan rate, where the slope represents C_{dl}. The ECSA was calculated as ECSA = C_{dl}/C_s, where C_s is the specific capacitance of the material. A general specific capacitances value of C_s = 40 uF cm^{-2} in 1 M KOH was used, based on typical commonly reported values for surface area estimation.

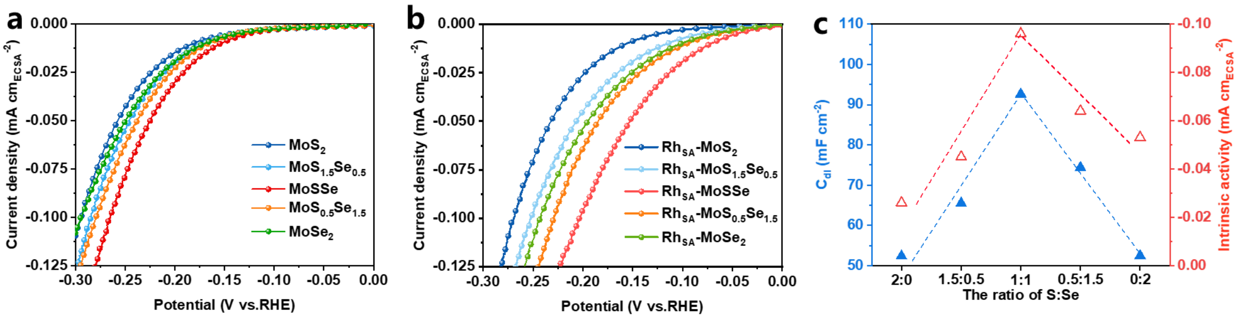

Fig. R7 (Supplementary Fig. 40 and 41b). HER activities of (a) MoS_xSe_{2-x} and (b) Rh_{SA}-MoS_xSe_{2-x} catalysts, normalized to ECSA. (c) Correlation between the S: Se ratio in Rh_{SA}-MoS_xSe_{2-x} and C_{dl} and intrinsic activity.

Comment: 5) *To demonstrate the practical relevance and support claims of high activity and durability, it is recommended that the authors perform actual AEMWE (Anion Exchange Membrane Water Electrolysis) tests. Such evaluations would provide more realistic insight into the catalyst performance under operating conditions. Including these results would significantly strengthen the manuscript by bridging fundamental electrochemical measurements and practical applicability.*

Response: Thank you for the suggestion. We have added anion exchange membrane water electrolyzer (AEMWE) tests to further evaluate the application potential of Rh_{SA}-MoSSe catalyst for industrial water splitting. The homemade NiFe LDH@NF as an anode electrocatalyst (Fig. R8) was assembled with Rh_{SA}-MoSSe and operated in 1.0 M KOH electrolyte under 80 °C. The polarization curves showed the Rh_{SA}-MoSSe||NiFe LDH@NF exhibit a cell voltage of 1.58 V and 1.70 V at current densities of 0.5 A cm^{-2} and 1.0 A cm^{-2} for AEMWE, respectively, which is superior to commercial Pt/C||NiFe LDH@NF and MoSSe||NiFe LDH@NF systems (Fig. R9a). Furthermore, the AEMWE of Rh_{SA}-MoSSe||NiFe LDH@NF was continuously operated for over 500 h at a current density of 1 A cm^{-2} (Fig. R9b). The cell voltage at 1.0 A cm^{-2} and stability time were comparable to those of recently reported catalysts for AEMWE (Table R1). The above results confirm the advanced and reliable potential of Rh_{SA}-MoSSe catalyst for industrial water splitting applications.

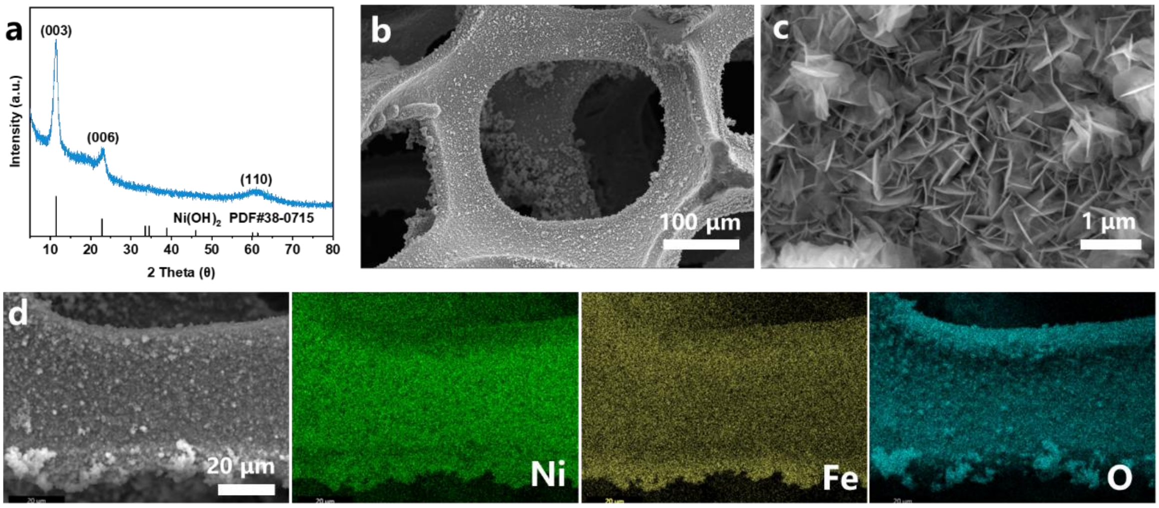

Fig. R8 (Supplementary Fig. 53). (a) XRD patterns of NiFe LDH@NF. (b and c) SEM images and (d) corresponding EDS elemental mapping of NiFe LDH@NF.

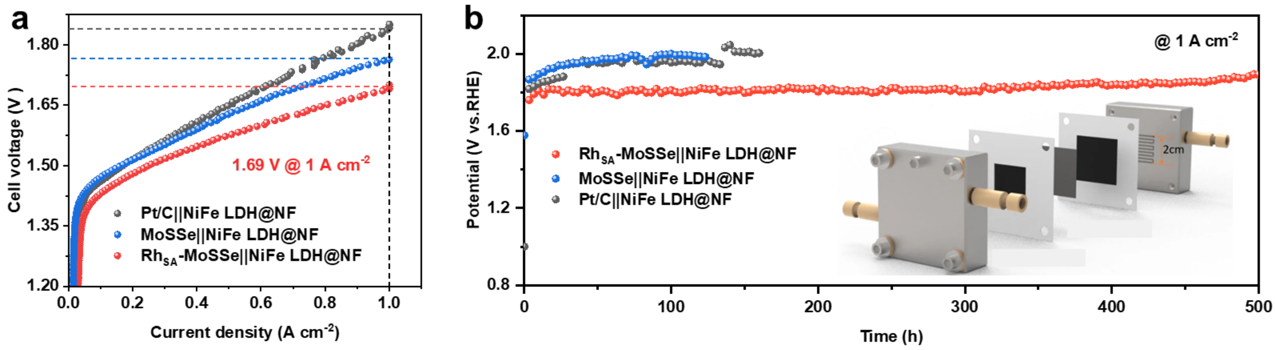

Fig. R9 (Figs. 4g and h). (a) Polarization curve of Rh_{SA}-MoSSe||NiFe LDH@NF, MoSSe||NiFe LDH@NF, and Pt/C||NiFe LDH@NF in the AEMWE operated at 80 °C. (b) Stability test of Rh_{SA}-MoSSe||NiFe LDH@NF, MoSSe||NiFe LDH@NF, and Pt/C||NiFe LDH@NF in the AEMWE at 1 A·cm^{-2}. Inset: schematic diagram of an AEMWE.

Table R1 (Supplementary Table 4). Comparison of cell voltage at a current density of 1 A cm^{-2} and stability time for recently reported catalysts applied in AEMWE.

Catalysts	electrolyte	Cell Voltage (V) @j = 1 A cm^{-2}	Stability Time	Ref.
Rh_{SA}-MoSSe||NiFe LDH@NF	1 M KOH	1.70	500 h@1 A cm^{-2}	This work
Pd, Ru, Rh, Pt, Re-$MoSe_2$||IrO_2	1 M KOH	1.82	500 h@1 A cm^{-2}	12
Ru SAs/WC_x||$NiFeOH_x$-NF	1 M KOH	1.79	190 h@1 A cm^{-2}	19

Ru-WC_{1-x}‖$NiFeO_x$	1 M KOH	1.80	100 h@1 A cm^{-2}	20
Ru-YNC‖NiFe LDH	1 M KOH	1.87	1000 h@0.5 A cm^{-2}	22
Ru-LC-$Ni(OH)_2$‖FeNi LDH	1 M KOH	1.69	500 h@0.5 A cm^{-2}	26
(Rh, O)-$MoSe_{2-x}$‖IrO_2	1 M KOH	No.	300 h@1.5 A cm^{-2}	29
1T p_z-MoS_2‖$RuIrO_2$	1 M KOH	1.78	200 h@0.5 A cm^{-2}	30
FeCo-MoS_2	0.5 M H_2SO_4	2.23	350 h@1 A cm^{-2}	31
$Pt_1/Ni(OH)_2$‖$Ir_1/Co(OH)_2$	1 M KOH	2.10	1030 h@0.8 A cm^{-2}	32
Co_n-Pt_1@NPC‖NiFe LDH	1 M KOH	2.03	100 h@1 A cm^{-2}	33

We have also added these contents to the revised manuscript highlighted in yellow in Line 12-23, Page 18, "The anion exchange membrane water electrolyzer (AEMWE) tests were employed to further evaluate the application potential of Rh_{SA}-MoSSe catalyst for industrial water splitting. The homemade NiFe LDH@NF as an anode electrocatalyst (Supplementary Fig. 53) was assembled with Rh_{SA}-MoSSe and operated in 1.0 M KOH electrolyte under 80 °C. The polarization curves showed the Rh_{SA}-MoSSe‖NiFe LDH@NF exhibit a cell voltage of 1.58 V and 1.70 V at current densities of 0.5 A cm^{-2} and 1.0 A cm^{-2} for AEMWE, respectively, which is superior to commercial Pt/C‖NiFe LDH@NF and MoSSe‖NiFe LDH@NF systems (Fig. 4g). Furthermore, the AEMWE of Rh_{SA}-MoSSe‖NiFe LDH@NF was continuously operated for over 500 h at a current density of 1 A cm^{-2} (Fig. 4h). The overpotential at 1.0 A cm^{-2} and stability time of were comparable to those of recently reported catalysts for AEMWE (Supplementary Table 4). The above results confirm the advanced and reliable potential of Rh_{SA}-MoSSe catalyst for industrial water splitting applications."

We have also added these contents to the revised supporting information highlighted in yellow in Line 12-18, Page 3, "**2.4 Synthesis of NiFe LDH@NF**. The NiFe LDH grown on Ni foam was synthesized through a hydrothermal reaction. Firstly, the solution was prepared by adding 0.5 mmol $Ni(NO_3)_2 \cdot 6H_2O$, 0.5 mmol $Fe(NO_3)_3 \cdot 9H_2O$, and 5 mmol $CO(NH_2)_2$ into 30 mL of water and transferred to an autoclave. Subsequently, the cleaned Ni foam was placed into the obtained solution. The hydrothermal reaction was maintained at 80 °C for 4 h. After cooling naturally to room temperature, the sample was washed with ethanol and ultrapure water several times and finally dried in air for further use.", and in Line 20-23, Page 4 and Line 1-7, Page 5, "**4. Anion-exchange**

membrane water electrolyzer (AEMWE) test. Anion-exchange membrane water electrolyzer (AEMWE) with serpentine flow channel were used to test practical applications of Rh_{SA}-MoSSe electrocatalysts. The anode electrode was prepared by spraying Rh_{SA}-MoSSe or Pt/C inks on Ni foam, the spraying was controlled to achieve a mass loading of 4.0 mg cm^{-2}. The homemade self-supporting NiFe LDH@NF was used as an anode electrocatalyst. Ni foam was used as a cathode porous current collector. Sustainion®X37-50 Grade 60 anion exchange membrane (Dioxide Materials Corporation) was activated by soaking in a 1.0 M KOH solution for 24 hours under 80 °C before use. The AEMWE test was operated at 80 °C with a peristaltic pump pumping 1.0 M KOH at a flow rate of 40 ml·min^{-1}. Polarization curves were obtained from 1 to 1.8 V at a stepwise rate of 5 mV s^{-1}. A long-term stability test was conducted at a current density of 1 A·cm^{-2} at 80 °C."

Comment: 6) *We request the following minor corrections:*

1) The sample names in Figure 5g should be corrected.

2) On page 4, line 72, the uppercase letter should be changed to lowercase.

Response: Thanks for the valuable comment. In the revised manuscript, we have corrected the sample names in Figure 5g and changed the uppercase letter to lowercase.

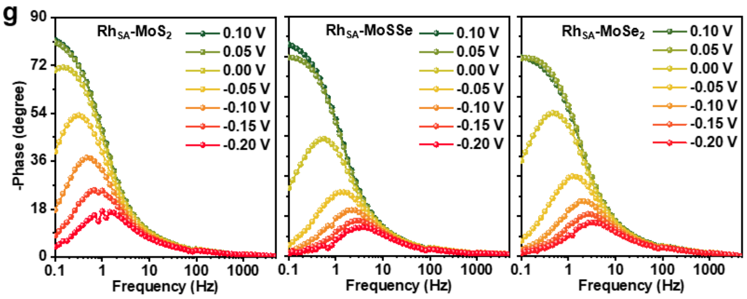

Fig. 5. (g) In situ bode plots for Rh_{SA}-MoS_2, Rh_{SA}-MoSSe, and Rh_{SA}-$MoSe_2$.

Reviewer #3 (Remarks to the Author):

In this work, Jia et al. studied MoSSe with varied S/Se ratios decorated with Rh single atoms. The authors modulated the d-p orbital hybridizations between the Rh and S/Se and studied their influences on the HER activity and stability of the catalysts toward the hydrogen evolution reaction in alkaline media. This is a complete and detailed study, with both experimental and theoretical investigations. However, this work is lacking of novelty and significance in terms of synthetic method, materials innovation and catalytic mechanism, and the findings does not represent significant advancements in the field. The materials of MoS/Se and their modified forms (especially single atom-doped ones) have been widely studied for HER electrocatalysis. In addition, whether the issue of activity-stability trade-off proposed by the authors exists remains unclear and the analyses on how they break this trade-off is insufficient. Overall, this reviewer believes that this work does not meet the high standards of Nature Communications. Some specific comments were provided below for the authors to consider to improve their work.

Response: We sincerely thank the reviewer for the insightful and constructive feedback. We have carefully addressed each point raised in the comments and have revised the manuscript accordingly. We carefully examined our work and further emphasized the novelty of our study, particularly in comparison with the materials of MoS/Se and their modified forms (especially single atom-doped ones). Moreover, we have added detailed mechanistic explanation on how break activity-stability trade-off. Below, we provide a detailed explanation of the key innovations in our work and the underlying mechanism of the activity-stability trade-off.

1. The catalytic mechanism novelty of our study.

Most works about transition metal dichalcogenides (TMDs) and their modified forms (especially single atom-doped ones) have primarily focused on the higher catalytic activity than non-modified ones towards HER due to the pair of metal-adsorbate and metal-support interactions (*Angew. Chem. Int. Ed.* **59**, 10502–10507; *Adv. Funct. Mater.* **27**, 1700359 (2017); *Nat. Commun.* **10**, 1743 (2019)). However, achieving precise metal-support interactions control through atomic-level compositional gradients remains challenging, impeding a clear understanding of structure-performance relationships. Notably, the role of metal-support frontier orbital hybridization in anion-engineered supports remains largely unexplored, representing a critical gap in the orbital-level design of SACs. While Zhang et al. (*Nature*, **640**, 668 (2025)) highlighted the influence of support LUMO levels on frontier orbital

interactions in Pd-based single-atom catalysts (SACs) for thermal catalysis, their approach remains confined to HOMO-LUMO alignment within oxide semiconductors. Our work extends this frontier orbital framework to electrochemical systems by introducing anion-gradient engineering as a generalizable strategy for precisely tuning HOMO-LUMO hybridization between Rh single atoms and compositionally engineered chalcogenide supports (MoS_xSe_{2-x}) (Fig. R1). We demonstrate that the elevated lowest unoccupied molecular orbital (LUMO) of MoS_xSe_{2-x} support narrows the energy gap with the highest occupied molecular orbital (HOMO) of Rh atoms, which promotes orbital hybridizations between Rh atoms and support to lead to a high stability and further amends the LUMO of anchored Rh atoms to optimize both the hydroxide (OH) and hydrogen (H) adsorption for high activity (Figs. R2 and R3). Our work offers fundamental insights into the metal-support frontier orbital interaction in SACs and establishes a rational design framework for high activity and stability electrocatalysis through anion-mediated metal-support interactions.

2. The underlying mechanism that breaks the activity-stability trade-off.

To gain a comprehensive understanding of metal-support frontier orbital interactions, we construct a model system of Rh single-atom catalysts (SACs), in which the HOMO of the Rh single atoms (Rh_{SA}) can be effectively modulated by tuning the anion composition in the support (Fig. R1). The first principal density functional theory (DFT) calculations indicate that the lowest unoccupied molecular orbital (LUMO) position of MoS_xSe_{2-x} substrates exhibits a volcano-type relationship with the S: Se ratio, with MoSSe located at the volcano apex (Fig. R2). Moreover, the elevated LUMO of MoSSe support narrows the energy gap with the HOMO of Rh atoms, which promotes orbital hybridizations between Rh atoms and support to lead to a high stability. When electrons are transferred from Rh to MoS_xSe_{2-x} substrates, the anchored Rh atoms exhibit an unoccupied or partially occupied state (or LUMO) in Rh_{SA}-MoS_xSe_{2-x}. Therefore, regulating Rh-MoS_xSe_{2-x} orbital coupling would facilitate charge transfer and effectively modulated the LUMO position of anchored Rh atoms. As shown in Fig. R3a and b, the projected density of states (PDOS) analysis demonstrates the LUMO position of Rh atoms exhibits a volcano-type relationship with the S: Se ratio in MoS_xSe_{2-x} substrates. The higher LUMO of Rh atoms on MoSSe exhibits smaller energy gaps (ΔE_1) with the HOMO of OH^- and larger energy gaps (ΔE_2) with the HOMO of H_2 molecules, which could directly alter the adsorption energies of key intermediates through systematic tuning the S: Se ratio in the support. Moreover, the S: Se ratio in Rh_{SA}-MoS_xSe_{2-x} series exhibits a volcano-type relationship with both the free energy of hydrogen

adsorption (ΔG_{H*}) and hydroxide adsorption (ΔG_{OH*}), with Rh_{SA}-MoSSe located at the volcano apex, displaying optimal adsorption strengths for both species (H and OH). In addition, the electrochemical measurements have demonstrated that the S: Se ratio in Rh_{SA}-MoS_xSe_{2-x} exhibits a volcano-type relationship with HER activity, Tafel slope, C_{dl}, intrinsic activity, and TOF, with Rh_{SA}-MoSSe at the volcano's apex displaying optimal overall HER performance. Moreover, we also added the chronopotentiometry (CP) tests of commercial Pt/C, MoSSe, Rh_{SA}-MoS_2, and Rh_{SA}-$MoSe_2$ for the stability comparison with Rh_{SA}-MoSSe. The CP studies reveal that the Rh_{SA}-MoSSe catalysts exhibit negligible potential decay over 450 h at a high current density of 1 A cm^{-2}, outperforming the commercial Pt/C, MoSSe, Rh_{SA}-MoS_2, and Rh_{SA}-$MoSe_2$ (Fig. R4). The experimental findings are fully consistent with the trends predicted by DFT calculations. Therefore, the Rh_{SA}-MoSSe with optimal HOMO-LUMO hybridization achieves superior HER activity and exceptional stability simultaneously, thereby breaking the activity-stability trade-off.

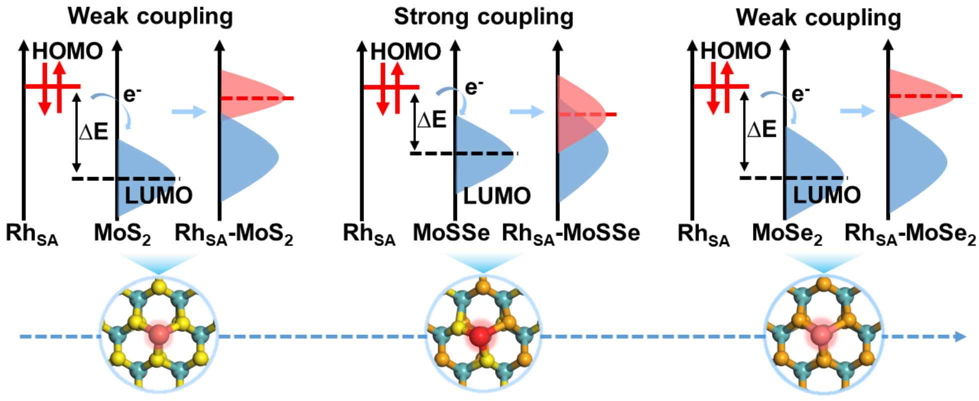

Fig. R1 (Fig. 1a). Schematic illustration of modulating MSIs in Rh_{SA}-MoS_xSe_{2-x} by varying the S: Se ratio of MoS_xSe_{2-x} supports.

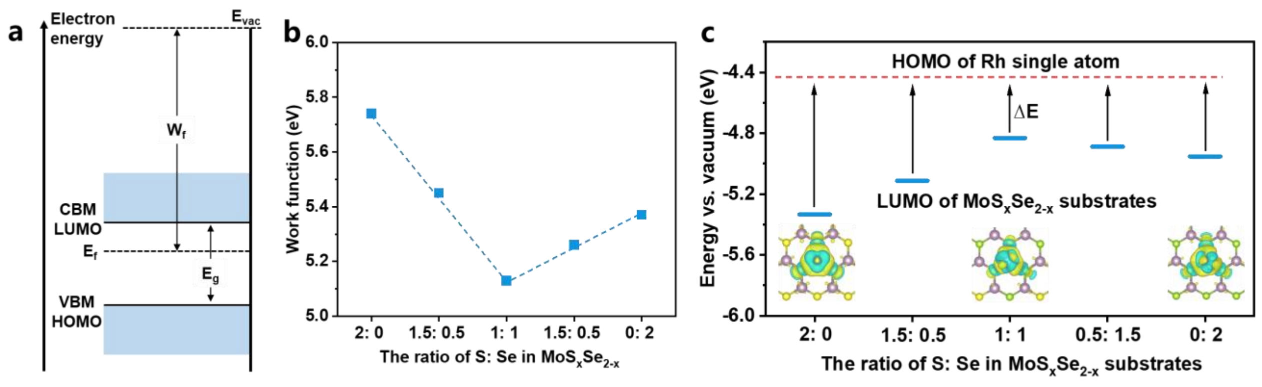

Fig. R2 (Supplementary Fig. 4 and Fig. 1b). (a) Energy diagram of a semiconductor with flat bands to the surface. Band edges (CBM/LUMO and VBM/HOMO), vacuum level E_{vac}, work function W_f,

and energy gap E_g are defined. (b) Dependence of the calculated work function on the S: Se ratio in MoS_xSe_{2-x}. (c) Calculated LUMO positions of MoS_xSe_{2-x} substrates with different S: Se ratios and the HOMO of unsupported Rh atom relative to vacuum. Black arrows highlight the energy discrepancies (ΔE) between the MoS_xSe_{2-x} LUMOs and the Rh atom HOMO. The insets show the charge density difference of Rh atoms in Rh_{SA}-MoS_2, Rh_{SA}-MoSSe, and Rh_{SA}-$MoSe_2$, with yellow and blue regions indicating charge accumulation and decrease, respectively.

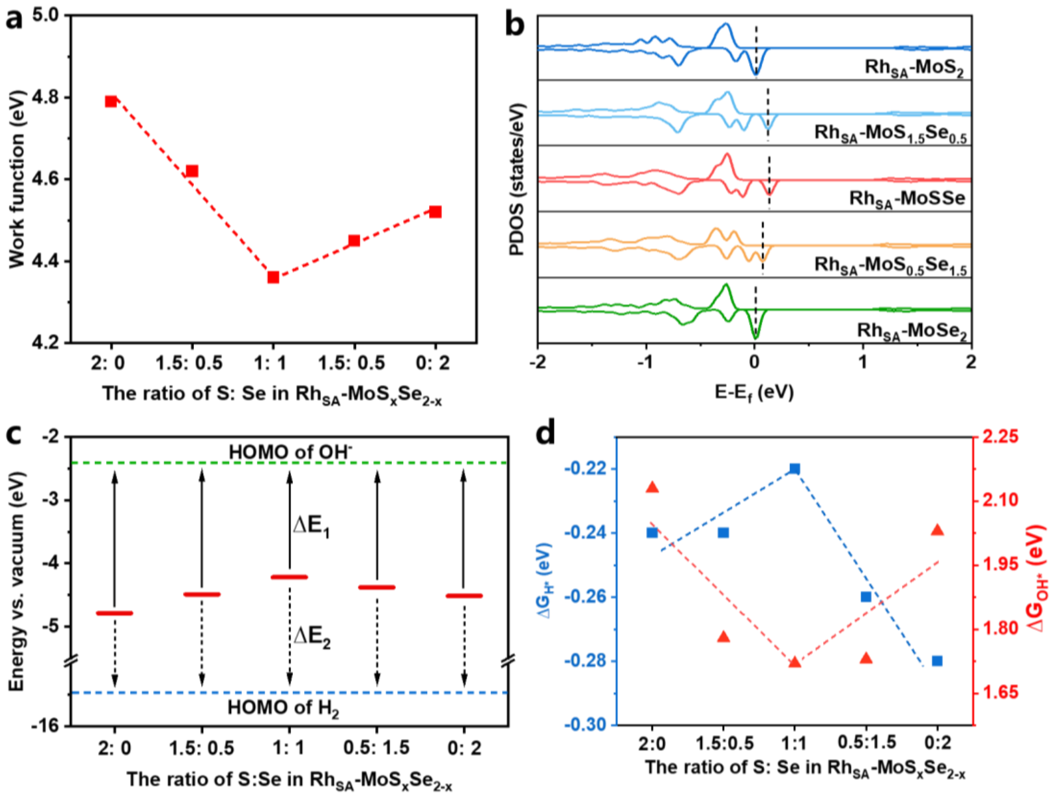

Fig. R3 (Supplementary Fig. 4, Fig. 1c and d). (a) Dependence of the calculated work function on the S: Se ratio in Rh_{SA}-MoS_xSe_{2-x}. (b) Calculated PDOS of anchored Rh atoms in Rh_{SA}-MoS_xSe_{2-x}, where bleak dashed lines denote the LUMO of Rh. (c) Calculated LUMO positions of Rh single atoms anchored on MoS_xSe_{2-x} substrates (red solid line) and the HOMO of OH^- (green dashed line) and H_2 (blue dashed line) relative to vacuum. Black arrows highlight the energy discrepancies (ΔE_1) between the Rh_{SA} LUMOs and the OH^- HOMO. Black dashed arrows highlight the energy discrepancies (ΔE_2) between the Rh_{SA} LUMOs and the H_2 HOMO. (d) Correlation between the S: Se ratio and ΔG_{OH*}, and ΔG_{H*} in Rh_{SA}-MoS_xSe_{2-x}.

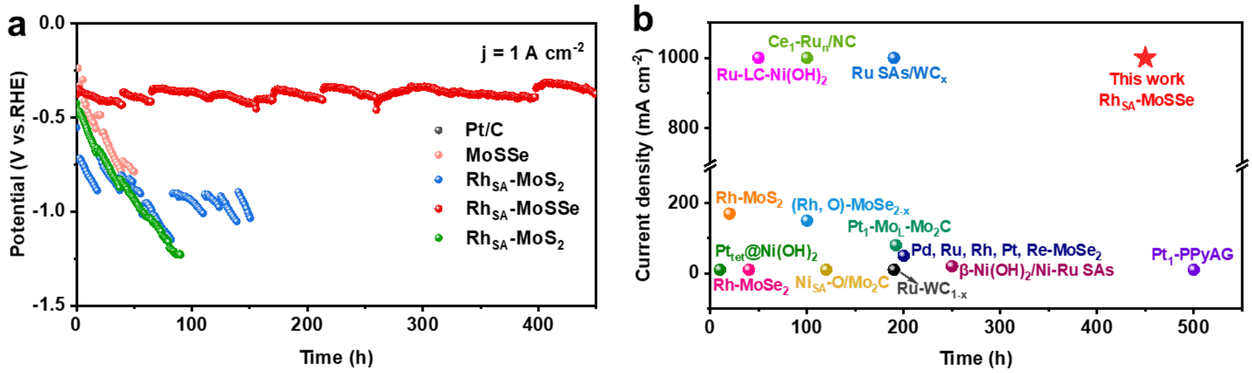

Fig. R4 (Supplementary Fig. 48 and Fig. 4f). (a) Chronoamperometric (CP) curves of Pt/C, MoSSe, Rh_{SA}-MoS_2, Rh_{SA}-MoSSe, and Rh_{SA}-$MoSe_2$ at a current density of 1 A cm^{-2}. (b) Comparison of HER stability for RhSA-MoSxSe2-x with previously reported state-of-the-art noble metal-based and TMDs-based catalysts.

***Comment: 1)** One key message that the authors try to convey is the breakage of the activity-stability trade-off by the Ru-MoSSe catalysts. However, the specific meaning and the fundamental causes behind this trade-off is not clarified nor analyzed.*

Response: Thank you for the valuable comment and acknowledge that the manuscript would benefit from a deeper mechanistic discussion of the activity-stability trade-off. While SACs maximize atom utilization, their performance is plagued by the universal activity-stability trade-off (*Chem. Soc. Rev.* **49**, 2215–2264 (2020); *Nat. Commun.* **10**, 1743 (2019); *Adv. Mater.* **33**, 2008151 (2021)). It has been widely demonstrated that the precise control of MSIs is essential for enhancing catalyst stability and optimizing the binding energies of reactants and intermediates, which govern the catalytic performance of SACs (*J. Am. Chem. Soc.* **144**, 9271–9279 (2022); *Nat. Commun.* **13**, 2473 (2022); *J. Am. Chem. Soc.* **145**, 23659–23669 (2023)). To gain a comprehensive understanding of metal-support frontier orbital interactions, we construct a model system of Rh single-atom catalysts (SACs), in which the HOMO of the Rh single atoms (Rh_{SA}) can be effectively modulated by tuning the anion composition in the support (Fig. R5). The first principal density functional theory (DFT) calculations indicate that the lowest unoccupied molecular orbital (LUMO) position of MoS_xSe_{2-x} substrates exhibits a volcano-type relationship with the S: Se ratio, with MoSSe located at the volcano apex (Fig. R6). Moreover, the elevated LUMO of MoSSe support narrows the energy gap with the HOMO of Rh atoms, which promotes orbital hybridizations between Rh atoms and support to lead to a high stability. When

electrons are transferred from Rh to MoS_xSe_{2-x} substrates, the anchored Rh atoms exhibit an unoccupied or partially occupied state (or LUMO) in Rh_{SA}-MoS_xSe_{2-x}. Therefore, regulating Rh-MoS_xSe_{2-x} orbital coupling would facilitate charge transfer and effectively modulated the LUMO position of anchored Rh atoms. As shown in Fig. R7a and b, the projected density of states (PDOS) analysis demonstrates the LUMO position of Rh atoms exhibits a volcano-type relationship with the S: Se ratio in MoS_xSe_{2-x} substrates. The higher LUMO of Rh atoms on MoSSe exhibits smaller energy gaps (ΔE_1) with the HOMO of OH^- and larger energy gaps (ΔE_2) with the HOMO of H_2 molecules, which could directly alter the adsorption energies of key intermediates through systematic tuning the S: Se ratio in the support. Moreover, the S: Se ratio in Rh_{SA}-MoS_xSe_{2-x} series exhibits a volcano-type relationship with both the free energy of hydrogen adsorption (ΔG_{H*}) and hydroxide adsorption (ΔG_{OH*}), with Rh_{SA}-MoSSe located at the volcano apex, displaying optimal adsorption strengths for both species (H and OH). In addition, the electrochemical measurements have demonstrated that the S: Se ratio in Rh_{SA}-MoS_xSe_{2-x} exhibits a volcano-type relationship with HER activity, Tafel slope, C_{dl}, intrinsic activity, and TOF, with Rh_{SA}-MoSSe at the volcano's apex displaying optimal overall HER performance. Moreover, we also added the chronopotentiometry (CP) tests of commercial Pt/C, MoSSe, Rh_{SA}-MoS_2, and Rh_{SA}-$MoSe_2$ for the stability comparison with Rh_{SA}-MoSSe. The CP studies reveal that the Rh_{SA}-MoSSe catalysts exhibit negligible potential decay over 450 h at a high current density of 1 A cm^{-2}, outperforming the commercial Pt/C, MoSSe, Rh_{SA}-MoS_2, and Rh_{SA}-$MoSe_2$ (Fig. R8). The experimental findings are fully consistent with the trends predicted by DFT calculations. Therefore, the Rh_{SA}-MoSSe with optimal HOMO-LUMO hybridization achieves superior HER activity and exceptional stability simultaneously, thereby breaking the activity-stability trade-off.

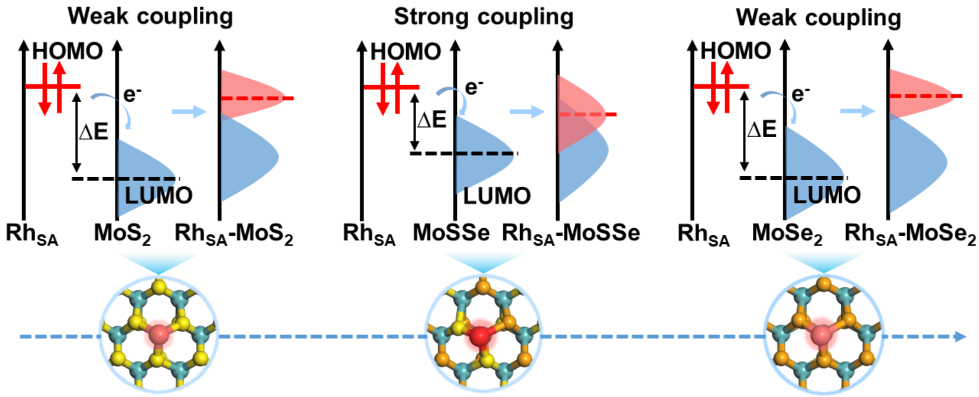

Fig. R5 (Fig. 1a). Schematic illustration of modulating MSIs in Rh_{SA}-MoS_xSe_{2-x} by varying the S: Se ratio of MoS_xSe_{2-x} supports.

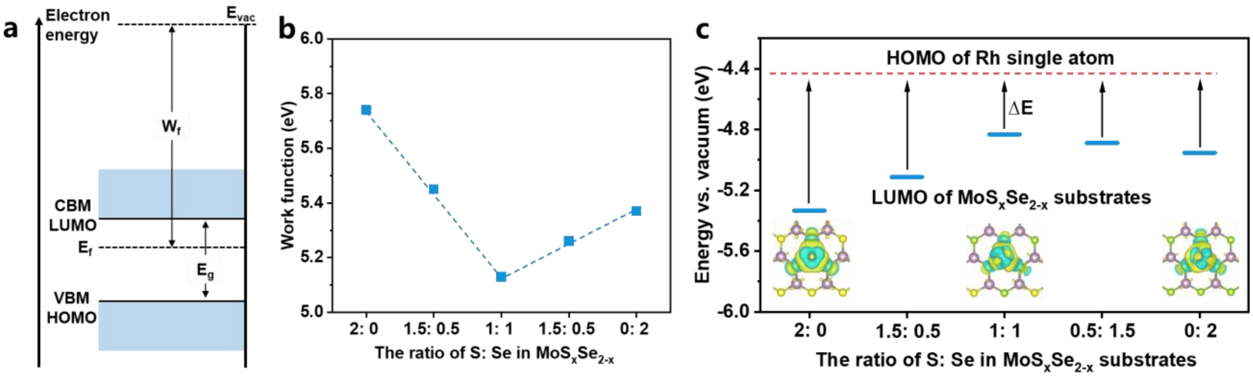

Fig. R6 (Supplementary Fig. 4 and Fig. 1b). (a) Energy diagram of a semiconductor with flat bands to the surface. Band edges (CBM/LUMO and VBM/HOMO), vacuum level E_{vac}, work function W_f, and energy gap E_g are defined. (b) Dependence of the calculated work function on the S: Se ratio in MoS_xSe_{2-x}. (c) Calculated LUMO positions of MoS_xSe_{2-x} substrates with different S: Se ratios and the HOMO of unsupported Rh atom relative to vacuum. Black arrows highlight the energy discrepancies (ΔE) between the MoS_xSe_{2-x} LUMOs and the Rh atom HOMO. The insets show the charge density difference of Rh atoms in Rh_{SA}-MoS_2, Rh_{SA}-MoSSe, and Rh_{SA}-$MoSe_2$, with yellow and blue regions indicating charge accumulation and decrease, respectively.

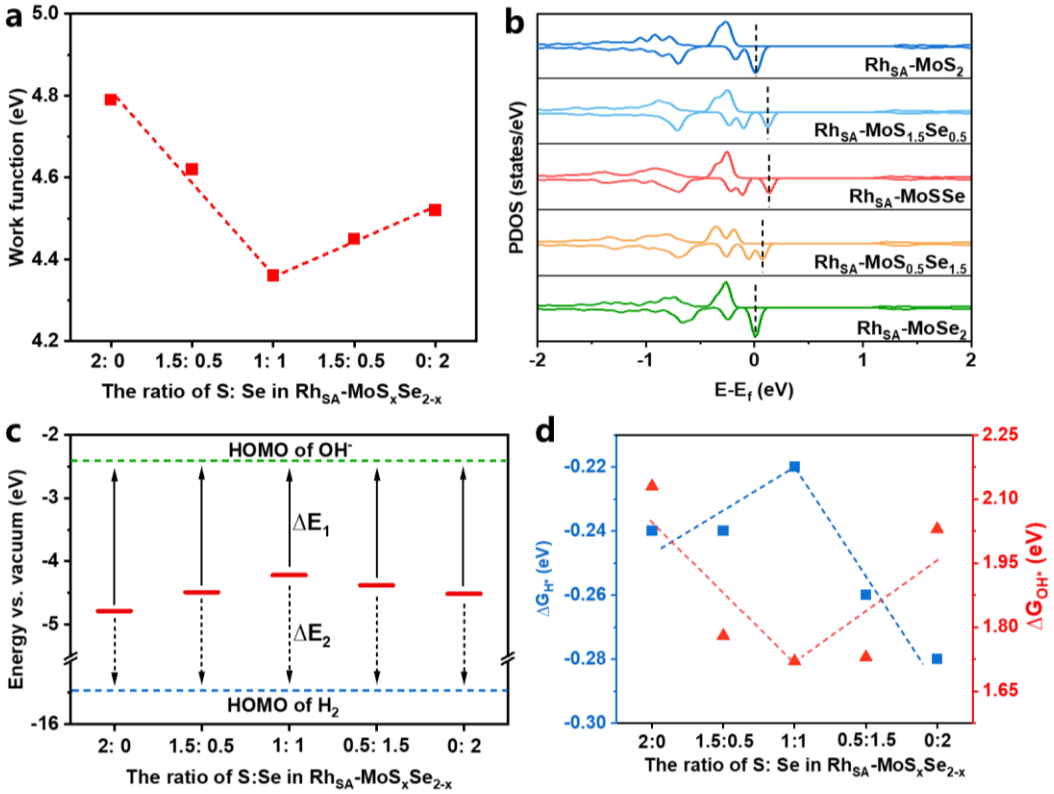

Fig. R7 (Supplementary Fig. 4, Fig. 1c and d). (a) Dependence of the calculated work function on the S: Se ratio in Rh_{SA}-MoS_xSe_{2-x}. (b) Calculated PDOS of anchored Rh atoms in Rh_{SA}-MoS_xSe_{2-x},

where bleak dashed lines denote the LUMO of Rh. (c) Calculated LUMO positions of Rh single atoms anchored on MoS_xSe_{2-x} substrates (red solid line) and the HOMO of OH^- (green dashed line) and H_2 (blue dashed line) relative to vacuum. Black arrows highlight the energy discrepancies (ΔE_1) between the Rh_{SA} LUMOs and the OH^- HOMO. Black dashed arrows highlight the energy discrepancies (ΔE_2) between the Rh_{SA} LUMOs and the H_2 HOMO. (d) Correlation between the S: Se ratio and ΔG_{OH^*}, and ΔG_{H^*} in Rh_{SA}-MoS_xSe_{2-x}.

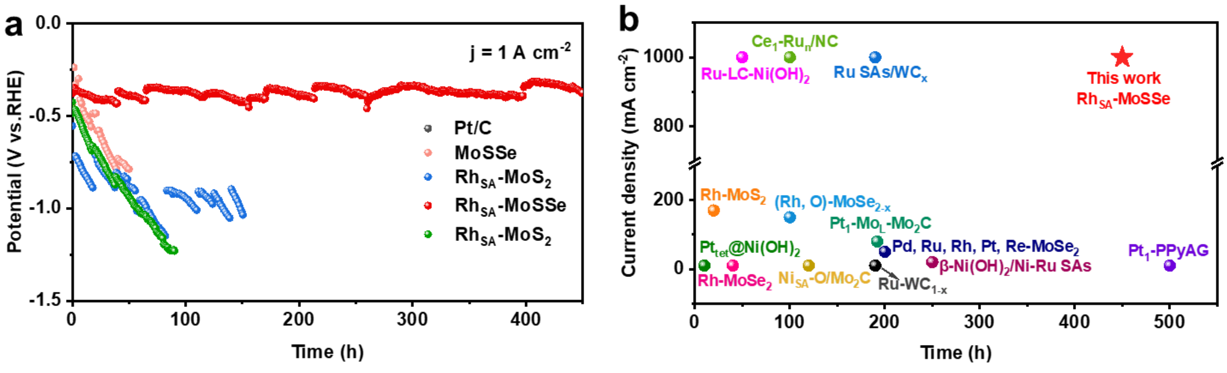

Fig. R8 (Supplementary Fig. 48 and Fig. 4f). (a) Chronoamperometric (CP) curves of Pt/C, MoSSe, Rh_{SA}-MoS_2, Rh_{SA}-MoSSe, and Rh_{SA}-$MoSe_2$ at a current density of 1 A cm^{-2}. (b) Comparison of HER stability for RhSA-MoSxSe2-x with previously reported state-of-the-art noble metal-based and TMDs-based catalysts.

We have also added these contents to the revised manuscript highlighted in yellow in Line 7-23, Page 5 and Line 5-9, Page 7, "The calculated HOMO position of freestanding Rh atom is located at -4.50 eV, whereas the LUMO of MoS_xSe_{2-x} substrates exhibit a volcano-type relationship with the S: Se ratio, with MoSSe located at the volcano apex (Fig. 1b, Supplementary Fig. 4, and Supplementary Note 1). According to the FMO theory[31–33], the elevated LUMO of MoSSe support narrows the energy gap with the highest occupied molecular orbital (HOMO) of Rh atoms, which promotes orbital hybridizations between Rh atoms and support to lead to a high stability. Charge density difference analyses reveal distinct charge redistribution between Rh single-atoms and coordinated S/Se atoms, resulting in a marked reduction of electron density around Rh atoms (Fig. 1b (inset)).

When electrons are transferred from Rh to MoS_xSe_{2-x} substrates, the anchored Rh atoms exhibit an unoccupied or partially occupied state (or LUMO) in Rh_{SA}-MoS_xSe_{2-x}. Therefore, regulating Rh-MoS_xSe_{2-x} orbital coupling would facilitate charge transfer and effectively modulated the LUMO position of anchored Rh atoms. As shown in Supplementary Fig. 4c, the projected density of states

(PDOS) analysis demonstrates the LUMO position of Rh atoms exhibits a volcano-type relationship with the S: Se ratio in MoS_xSe_{2-x} substrates. The higher LUMO of Rh atoms on MoSSe exhibits smaller energy gaps (ΔE_1) with the HOMO of OH^- and larger energy gaps (ΔE_2) with the HOMO of H_2 molecules (Fig. 1c), which could directly alter the adsorption energies of key intermediates through systematic tuning the S: Se ratio in the support. Moreover, the S: Se ratio in Rh_{SA}-MoS_xSe_{2-x} series exhibits a volcano-type relationship with both the free energy of hydrogen adsorption (ΔG_{H^*}) and hydroxide adsorption (ΔG_{OH^*}), with Rh_{SA}-MoSSe located at the volcano apex, displaying optimal adsorption strengths for both species (H and OH) (Fig. 1d and Supplementary Figs. 5 and 6).".and in Line 8-14, Page 16, "Long-term durability of Rh_{SA}-MoSSe was assessed via chronopotentiometry, demonstrating negligible potential decay over 450 h at a high current density of 1 A cm^{-2}, outperforming the commercial Pt/C, MoSSe, Rh_{SA}-MoS_2, and Rh_{SA}-$MoSe_2$ (Supplementary Fig. 48). The experimental findings are fully consistent with the trends predicted by the above DFT calculations. Moreover, the stability time of Rh_{SA}-MoSSe at 1 A cm^{-2} surpasses most reported noble metal-based and transition metal dichalcogenide (TMD) catalysts (Fig. 4f and Supplementary Table 3)[13,17,47–50,55–67]."

***Comment:* 2)** *The rationale for choosing Rh single atoms to study the d-p orbital hybridization for enhanced HER should be explicitly provided. Considering that Pt and Ru precious metals are more frequently and suitable for HER electrocatalysis, Pt and Ru single atoms should be studied to demonstrate the generality of the design strategy.*

Response: Thank you for the kind suggestion. The HER generally exhibits much poorer kinetics in alkaline electrolytes than in acidic electrolytes due to the sluggish water dissociation and the poor proton supply rate. It was demonstrated that the rate of alkaline hydrogen evolution exhibits a volcano-type relationship with the hydroxide adsorption strength (ΔG_{OH^*}). The rate-determining step (RDS) on the too-weak binding side of the ΔG_{OH^*} volcano curve is the water dissociation, whereas the RDS on the too-strong binding side is the hydroxide desorption (*Nat. Energy* **5**, 891-899 (2020); *Nat. Commun.* **13**, 5497 (2022); *J. Am. Chem. Soc.* **146**, 4883-4891 (2024)). Moreover, the alkaline HER rate can also be correlated to the calculated hydrogen binding energy (HBE) on the metal surfaces (*Nat. Commun.* **6**, 5848 (2015); *Energy Environ. Sci.* **6**, 1509–1512 (2013)). Therefore, developing electrocatalysts with both optimized hydrogen binding strength and hydroxide binding strength is of paramount

importance to enhance the alkaline HER activity. In the past decades, Rh-based catalysts have shown great potential to substitute Pt/C for alkaline HER by virtue of the much lower energy barrier for water dissociation and approximate HBE with Pt (*Angew. Chem. Int. Ed.* **59**, 10502–10507 (2020); *Adv. Funct. Mater.* **27**, 1700359 (2017); *Nat. Commun.* **7**, 12272 (2016)). Therefore, we chose Rh single atoms to study the d-p orbital hybridization for enhanced HER.

Moreover, we studied Pt and Ru single atoms anchored on MoS_xSe_{2-x} to demonstrate the generality of the design strategy. The XRD of Ru_{SA}-MoSSe and Pt_{SA}-MoS_xSe_{2-x} was shown in Fig. R9, confirming no diffraction peaks of Ru or Pt nanoparticles were observed. Additionally, the SEM and corresponding elemental mapping results reveals the uniform distribution of every element (Fig. R10 and R11). Notably, the S: Se ratio in both Ru_{SA}-MoS_xSe_{2-x} and Pt_{SA}-MoS_xSe_{2-x} exhibits a volcano-type relationship with HER activity and Tafel slope, with Ru_{SA}-MoSSe and Pt_{SA}-MoSSe at the volcano's apex displaying optimal overall HER performance (Figs. R12 and 13).

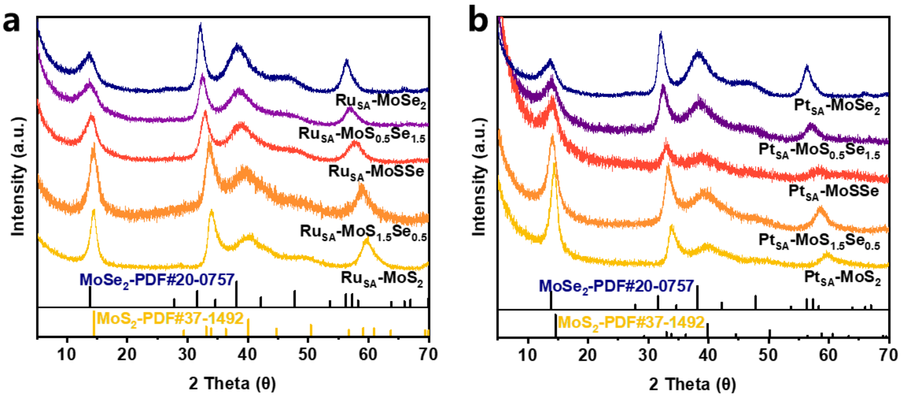

Fig. R9 (Supplementary Fig. 42). XRD patterns of (a) Ru_{SA}-MoS_xSe_{2-x} catalysts, and (b) Pt_{SA}-MoS_xSe_{2-x} catalysts.

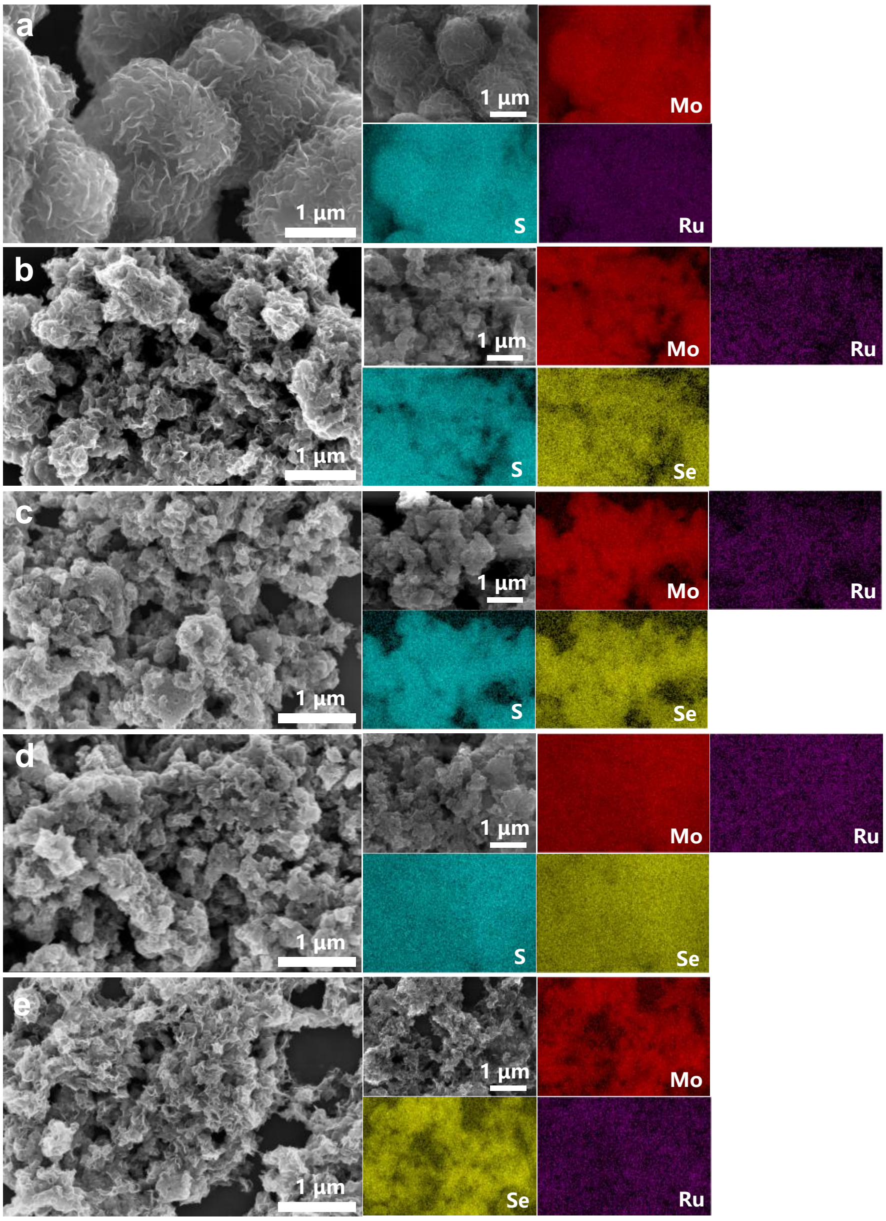

Fig. R10 (Supplementary Fig. 43). SEM images and corresponding EDS elemental mapping of (a) Ru_{SA}-MoS_2, (b) Ru_{SA}-$MoS_{1.5}Se_{0.5}$, (c) Ru_{SA}-MoSSe, (d) Ru_{SA}-$MoS_{1.5}Se_{0.5}$, and (e) Ru_{SA}-$MoSe_2$.

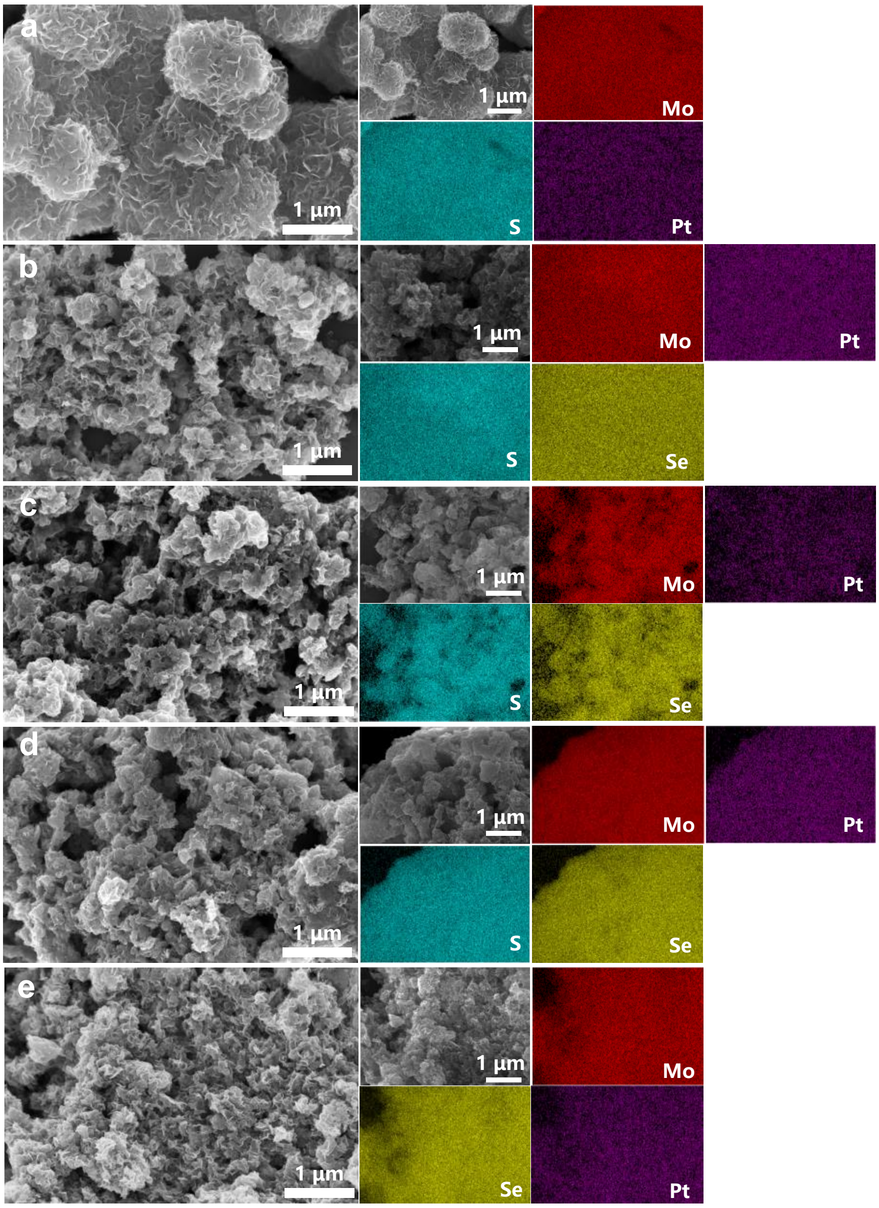

Fig. R11 (Supplementary Fig. 44). SEM images and corresponding EDS elemental mapping of (a) Pt_{SA}-MoS_2, (b) Pt_{SA}-$MoS_{1.5}Se_{0.5}$, (c) Pt_{SA}-MoSSe, (d) Pt_{SA}-$MoS_{1.5}Se_{0.5}$, and (e) Pt_{SA}-$MoSe_2$.

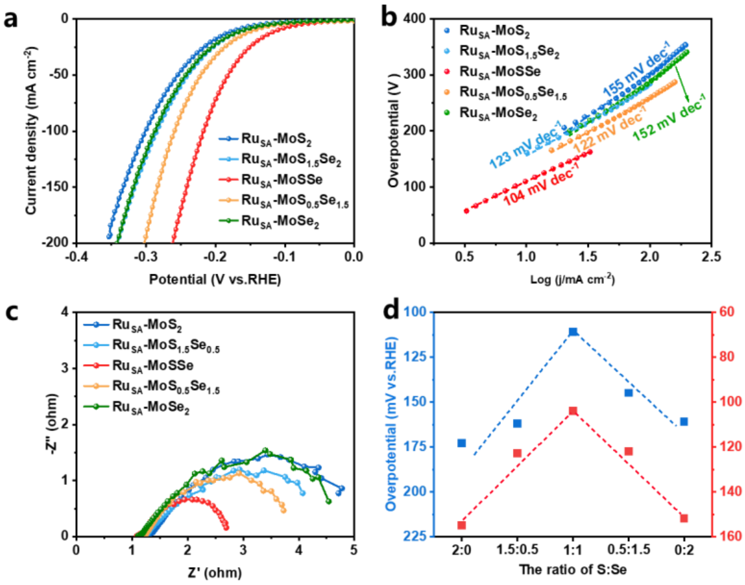

Fig. R12 (Supplementary Fig. 45). (a) HER polarization curves, (b) Tafel slopes, and (c) Nyquist plots of Ru_{SA}-MoS_xSe_{2-x} at -0.1 V vs. RHE. (d) (a) Correlation between the S:Se ratio in Ru_{SA}-MoS_xSe_{2-x} and overpotential at 10 mA cm^{-2} and Tafel slope.

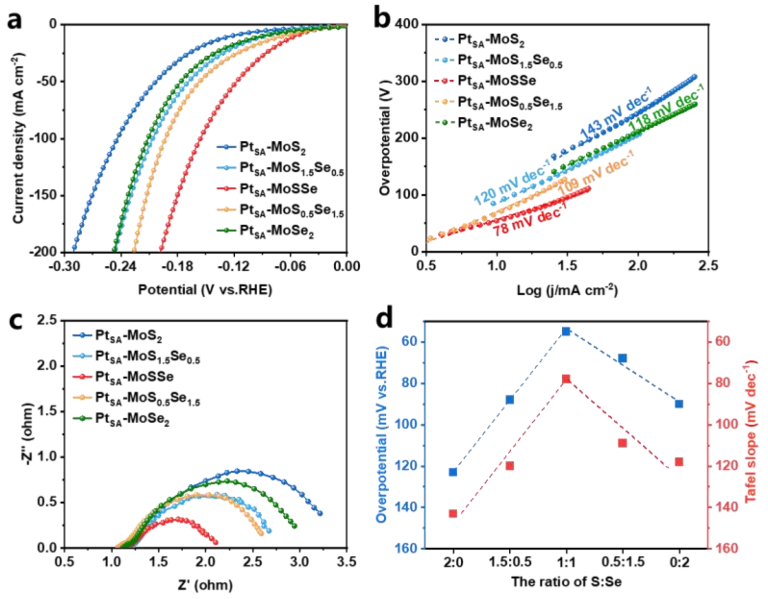

Fig. R13 (Supplementary Fig. 46). (a) HER polarization curves, (b) Tafel slopes, and (c) Nyquist plots of Pt_{SA}-MoS_xSe_{2-x} at -0.1 V vs. RHE. (d) (a) Correlation between the S:Se ratio in Pt_{SA}-MoS_xSe_{2-x} and overpotential at 10 mA cm^{-2} and Tafel slope.

We have also added these contents to the revised manuscript highlighted in yellow in Line 6-9, Page 3, "In the past decades, Rh-based catalysts have shown great potential to substitute Pt/C for alkaline HER by virtue of the much lower energy barrier for water dissociation and approximate H adsorption energy with Pt[12–14], but their scarcity and high cost greatly hindered the large-scale applications.", and

in Line 8-16, Page 15, "Moreover, the Pt and Ru single atoms anchored on MoS_xSe_{2-x} were also successfully synthesized to demonstrate the generality of the design strategy. The XRD of Ru_{SA}-MoSSe and Pt_{SA}-MoS_xSe_{2-x} was shown in Supplementary Fig. 42, confirming no diffraction peaks of Ru or Pt nanoparticles were observed. Additionally, the SEM and corresponding elemental mapping results reveals the uniform distribution of every element (Supplementary Fig. 43 and 44). Notably, the S: Se ratio in both Ru_{SA}-MoS_xSe_{2-x} and Pt_{SA}-MoS_xSe_{2-x} exhibits a volcano-type relationship with HER activity and Tafel slope, with Ru_{SA}-MoSSe and Pt_{SA}-MoSSe at the volcano's apex displaying optimal overall HER performance (Supplementary Figs. 45 and 46)."

***Comment:* 3)** *For the performance comparison with previous works, the stability as an important parameter should be included and analyzed in detail.*

Response: Thank you very much for the critical and valuable comment. We fully agree that long-term stability is a paramount metric for assessing the practical viability of electrocatalysts, often even more crucial than initial activity. The first principal density functional theory (DFT) calculations indicate that the lowest unoccupied molecular orbital (LUMO) position of MoS_xSe_{2-x} substrates exhibits a volcano-type relationship with the S: Se ratio, with MoSSe located at the volcano apex (Fig. R14). Moreover, the elevated LUMO of MoSSe support narrows the energy gap with the HOMO of Rh atoms, which promotes orbital hybridizations between Rh atoms and support to lead to a high stability. Moreover, we have added the chronopotentiometry (CP) tests of commercial Pt/C, MoSSe, Rh_{SA}-MoS_2, and Rh_{SA}-$MoSe_2$ for the stability comparison with Rh_{SA}-MoSSe. The CP studies reveal that the Rh_{SA}-MoSSe catalysts exhibit negligible potential decay over 450 h at a high current density of 1 A cm^{-2}, outperforming the commercial Pt/C, MoSSe, Rh_{SA}-MoS_2, and Rh_{SA}-$MoSe_2$ (Fig. R15a). The experimental findings are fully consistent with the trends predicted by the above DFT calculations. Moreover, the stability time of Rh_{SA}-MoSSe at 1 A cm^{-2} surpasses most reported noble metal-based and transition metal dichalcogenide (TMD) catalysts (Fig. R15b and Table R1). The experimental findings are fully consistent with the trends predicted by the above DFT calculations.

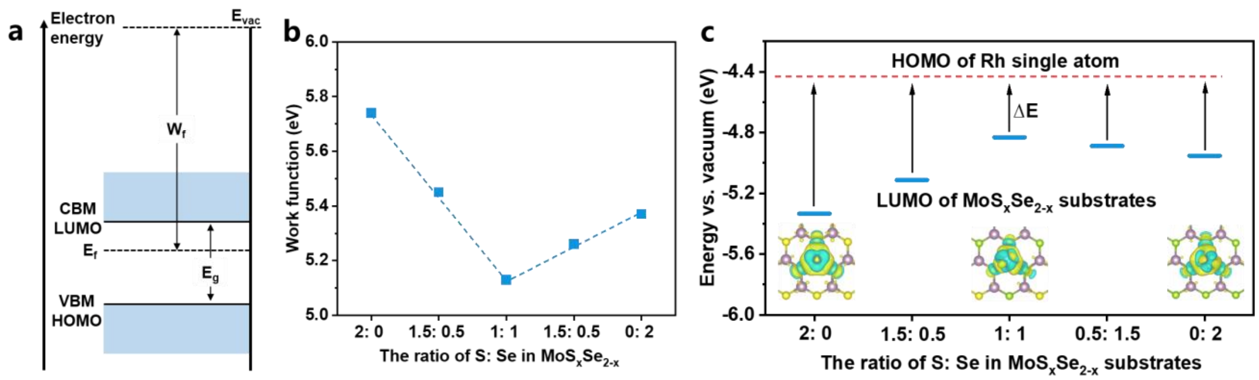

Fig. R14 (Supplementary Fig. 4 and Fig. 1b). (a) Energy diagram of a semiconductor with flat bands to the surface. Band edges (CBM/LUMO and VBM/HOMO), vacuum level E_{vac}, work function W_f, and energy gap E_g are defined. (b) Dependence of the calculated work function on the S: Se ratio in MoS_xSe_{2-x}. (c) Calculated LUMO positions of MoS_xSe_{2-x} substrates with different S: Se ratios and the HOMO of unsupported Rh atom relative to vacuum. Black arrows highlight the energy discrepancies (ΔE) between the MoS_xSe_{2-x} LUMOs and the Rh atom HOMO. The insets show the charge density difference of Rh atoms in Rh_{SA}-MoS_2, Rh_{SA}-MoSSe, and Rh_{SA}-$MoSe_2$, with yellow and blue regions indicating charge accumulation and decrease, respectively.

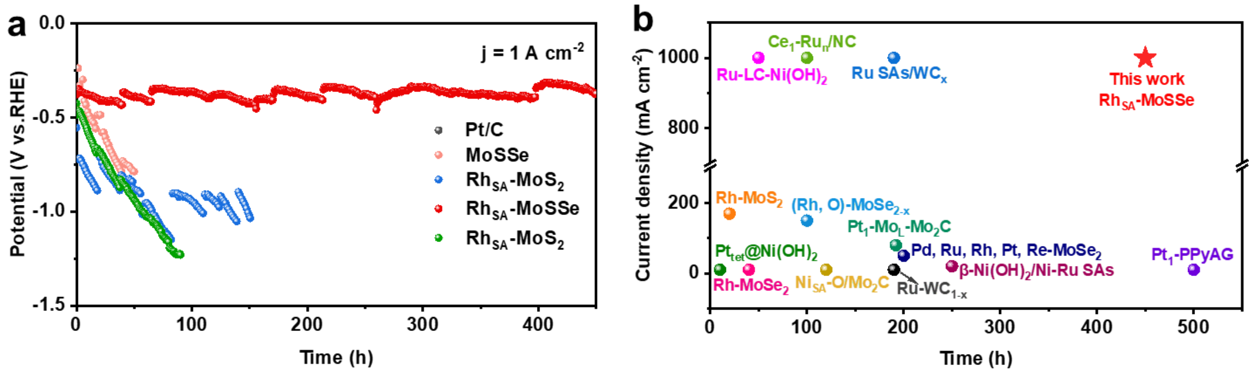

Fig. R15 (Supplementary Fig. 48 and Fig. 4f). (a) Chronoamperometric (CP) curves of Pt/C, MoSSe, Rh_{SA}-MoS_2, Rh_{SA}-MoSSe, and Rh_{SA}-$MoSe_2$ at a current density of 1 A cm^{-2}. (b) Comparison of HER stability for RhSA-MoSxSe2-x with previously reported state-of-the-art noble metal-based and TMDs-based catalysts.

Table R1 (Supplementary Table 3). Comparison of HER stability for Rh_{SA}-MoS_xSe_{2-x} with previously reported state-of-the-art noble metal-based and TMDs-based catalysts.

Catalysts	electrolyte	Current density	Time	Ref.
Rh_{SA}-MoSSe	1 M KOH	1000 mA cm^{-2}	> 450 h	This work

Rh-$MoSe_2$	0.5 M H_2SO_4	10 mA cm^{-2}	40 h	9
Pt/np-$Co_{0.85}Se$	1.0 M PBS	10 mA cm^{-2}	40 h	10
Ce_1-Ru_n/NC	1 M KOH	1000 mA cm^{-2}	100 h	11
Pd, Ru, Rh, Pt, Re-$MoSe_2$	1 M KOH	50 mA cm^{-2}	200	12
Rh-MoS_2	1 M KOH	170 mA cm^{-2}	20 h	13
Pt_{tet}@$Ni(OH)_2$	1 M KOH	10 mA cm^{-2}	10 h	18
Ru SAs/WC_x	1 M KOH	1000 mA cm^{-2}	190 h	19
Ru-WC_{1-x}	1 M KOH	10 mA cm^{-2}	190 h	20
Pt_1-Mo_L-Mo_2C	1 M KOH	80 mA cm^{-2}	192 h	21
Ru-YNC	1 M KOH	500 mA cm^{-2}	1000 h	22
Ni_{SA}-O/Mo_2C	1 M KOH	10 mA cm^{-2}	120 h	23
Pt_1-PPyAG	1 M KOH	10 mA cm^{-2}	500 h	24
β-$Ni(OH)_2$/Ni-Ru SAs	1 M KOH	20 mA cm^{-2}	250 h	25
Ru-LC-$Ni(OH)_2$	1 M KOH	1000 mA cm^{-2}	50 h	26
2H@1T-MoS_2-Sn_1	0.5 M H_2SO_4	10 mA cm^{-2}	240 h	27
Tm SAs-MoS_2	1 M KOH	10 mA cm^{-2}	27 h	28
(Rh, O)-$MoSe_{2-x}$	1 M KOH	150 mA cm^{-2}	100 h	29
1T p_z-MoS_2	1 M KOH	100 mA cm^{-2}	50 h	30
FeCo-MoS_2	0.5 M H_2SO_4	1000 mA cm^{-2}	350 h	31

We have also added these contents to the revised manuscript highlighted in yellow in Line 8-14, Page 16, "Long-term durability of Rh_{SA}-MoSSe was assessed via chronopotentiometry, demonstrating negligible potential decay over 450 h at a high current density of 1 A cm^{-2}, outperforming the commercial Pt/C, MoSSe, Rh_{SA}-MoS_2, and Rh_{SA}-$MoSe_2$ (Supplementary Fig. 48). The experimental findings are fully consistent with the trends predicted by the above DFT calculations. Moreover, the stability time of Rh_{SA}-MoSSe at 1 A cm^{-2} surpasses most reported noble metal-based and transition metal dichalcogenide (TMD) catalysts (Fig. 4f and Supplementary Table 3)[13,17,47–50,55–67]."

***Comment:* 4)** *The performance of the catalysts was analyzed in conventional three-electrode system. Considering that MoS_2 and its modified form as HER catalysts have been extensively reported, their catalytic performance in anion-exchange membrane full cells is recommended to be included to evaluate their potential for practical applications.*

Response: Thanks for the valuable comment. We have added anion exchange membrane water

electrolyzer (AEMWE) tests to further evaluate the application potential of Rh_{SA}-MoSSe catalyst for industrial water splitting. The homemade NiFe LDH@NF as an anode electrocatalyst (Fig. R16) was assembled with Rh_{SA}-MoSSe and operated in 1.0 M KOH electrolyte under 80 °C. The polarization curves showed the Rh_{SA}-MoSSe||NiFe LDH@NF exhibit a cell voltage of 1.58 V and 1.70 V at current densities of 0.5 A cm^{-2} and 1.0 A cm^{-2} for AEMWE, respectively, which is superior to commercial Pt/C||NiFe LDH@NF and MoSSe||NiFe LDH@NF systems (Fig. R17a). Furthermore, the AEMWE of Rh_{SA}-MoSSe||NiFe LDH@NF was continuously operated for over 500 h at a current density of 1 A cm^{-2}, which is much better than commercial Pt/C||NiFe LDH@NF and MoSSe||NiFe LDH@NF systems (Fig. R17b). The overpotential at 1.0 A cm^{-2} and stability time of were comparable to those of recently reported catalysts for AEMWE (Table R2). The above results confirm the advanced and reliable potential of Rh_{SA}-MoSSe catalyst for industrial water splitting applications.

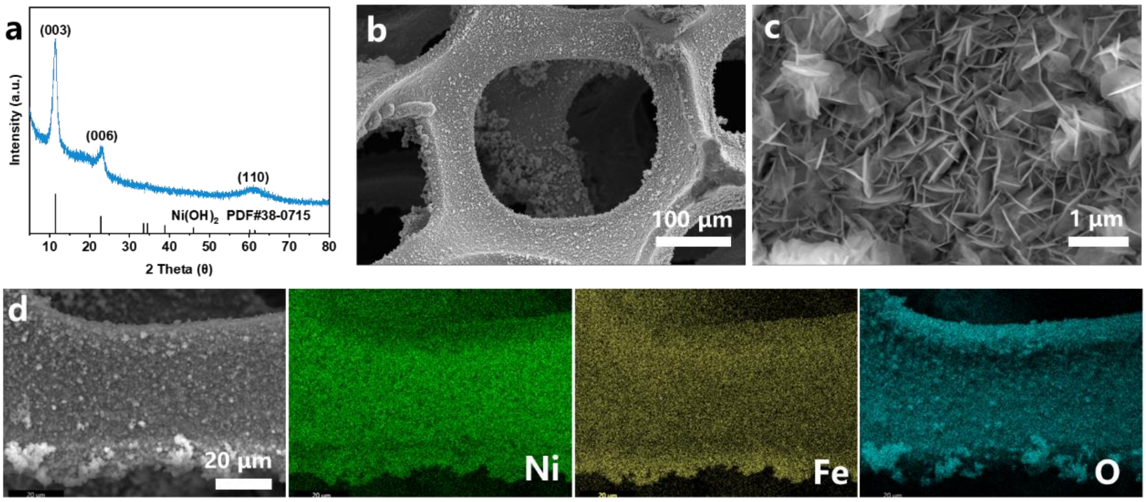

Fig. R16 (Supplementary Fig. 53). (a) XRD patterns of NiFe LDH@NF. SEM images (b and c) and corresponding EDS elemental mapping (d) of NiFe LDH@NF.

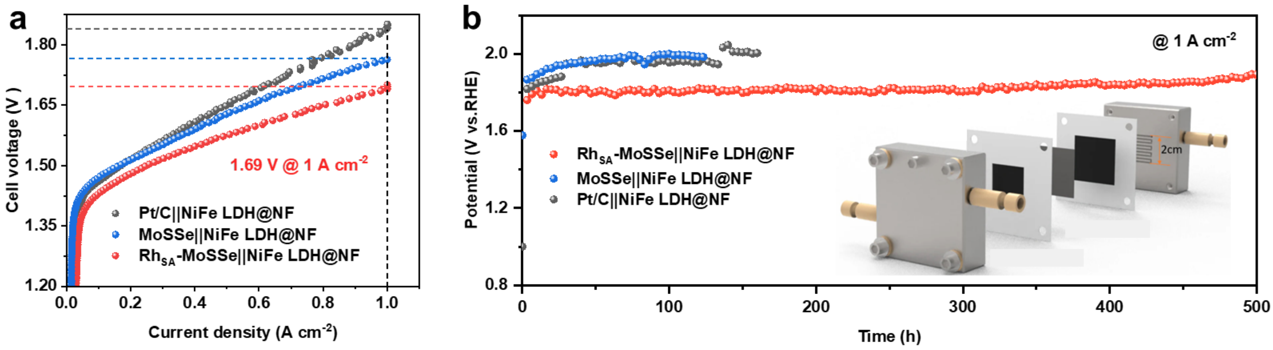

Fig. R17 (Fig. 4g and h). (a) Polarization curve of Rh_{SA}-MoSSe||NiFe LDH@NF, MoSSe||NiFe

LDH@NF, and Pt/C||NiFe LDH@NF in the AEMWE operated at 80 °C. (b) Stability test of Rh_{SA}-MoSSe||NiFe LDH@NF, MoSSe||NiFe LDH@NF, and Pt/C||NiFe LDH@NF in the AEMWE at 1 A·cm^{-2}. Inset: schematic diagram of an AEMWE.

Table R2 (Supplementary Table 4). Comparison of cell voltage at a current density of 1 A cm^{-2} and stability time for recently reported catalysts applied in AEMWE

Catalysts	electrolyte	Cell Voltage (V) @j = 1 A cm^{-2}	Stability Time	Ref.
Rh_{SA}-MoSSe||NiFe LDH@NF	1 M KOH	1.70	500 h@1 A cm^{-2}	This work
Pd, Ru, Rh, Pt, Re-$MoSe_2$||IrO_2	1 M KOH	1.82	500 h@1 A cm^{-2}	12
Ru SAs/WC_x||$NiFeOH_x$-NF	1 M KOH	1.79	190 h@1 A cm^{-2}	19
Ru-WC_{1-x}||$NiFeO_x$	1 M KOH	1.80	100 h@1 A cm^{-2}	20
Ru-YNC||NiFe LDH	1 M KOH	1.87	1000 h@0.5 A cm^{-2}	22
Ru-LC-$Ni(OH)_2$||FeNi LDH	1 M KOH	1.69	500 h@0.5 A cm^{-2}	26
(Rh, O)-$MoSe_{2-x}$||IrO_2	1 M KOH	No.	300 h@1.5 A cm^{-2}	29
1T p_z-MoS_2||$RuIrO_2$	1 M KOH	1.78	200 h@0.5 A cm^{-2}	30
FeCo-MoS_2	0.5 M H_2SO_4	2.23	350 h@1 A cm^{-2}	31
$Pt_1/Ni(OH)_2$||$Ir_1/Co(OH)_2$	1 M KOH	2.10	1030 h@0.8 A cm^{-2}	32
Co_n-Pt_1@NPC||NiFe LDH	1 M KOH	2.03	100 h@1 A cm^{-2}	33

We have also added these contents to the revised manuscript highlighted in yellow in Line 12-23, Page 18, "The anion exchange membrane water electrolyzer (AEMWE) tests were employed to further evaluate the application potential of Rh_{SA}-MoSSe catalyst for industrial water splitting. The homemade NiFe LDH@NF as an anode electrocatalyst (Supplementary Fig. 53) was assembled with Rh_{SA}-MoSSe and operated in 1.0 M KOH electrolyte under 80 °C. The polarization curves showed the Rh_{SA}-MoSSe||NiFe LDH@NF exhibit a cell voltage of 1.58 V and 1.70 V at current densities of 0.5 A cm^{-2} and 1.0 A cm^{-2} for AEMWE, respectively, which is superior to commercial Pt/C||NiFe LDH@NF and MoSSe||NiFe LDH@NF systems (Fig. 4g). Furthermore, the AEMWE of Rh_{SA}-MoSSe||NiFe LDH@NF was continuously operated for over 500 h at a current density of 1 A cm^{-2} (Fig. 4h). The overpotential at 1.0 A cm^{-2} and stability time of were comparable to those of recently reported catalysts for AEMWE (Supplementary Table 4). The above results confirm the advanced and reliable potential of Rh_{SA}-MoSSe catalyst for industrial water splitting applications."

We have also added these contents to the revised supporting information highlighted in yellow in Line 12-18, Page 3, "**2.4 Synthesis of NiFe LDH@NF**. The NiFe LDH grown on Ni foam was synthesized through a hydrothermal reaction. Firstly, the solution was prepared by adding 0.5 mmol $Ni(NO_3)_2 \cdot 6H_2O$, 0.5 mmol $Fe(NO_3)_3 \cdot 9H_2O$, and 5 mmol $CO(NH_2)_2$ into 30 mL of water and transferred to an autoclave. Subsequently, the cleaned Ni foam was placed into the obtained solution. The hydrothermal reaction was maintained at 80 °C for 4 h. After cooling naturally to room temperature, the sample was washed with ethanol and ultrapure water several times and finally dried in air for further use.", and in Line 20-23, Page 4 and Line 1-7, Page 5, "**4. Anion-exchange membrane water electrolyzer (AEMWE) test.** Anion-exchange membrane water electrolyzer (AEMWE) with serpentine flow channel were used to test practical applications of Rh_{SA}-MoSSe electrocatalysts. The anode electrode was prepared by spraying Rh_{SA}-MoSSe or Pt/C inks on Ni foam, the spraying was controlled to achieve a mass loading of 4.0 mg cm^{-2}. The homemade self-supporting NiFe LDH@NF was used as an anode electrocatalyst. Ni foam was used as a cathode porous current collector. Sustainion®X37-50 Grade 60 anion exchange membrane (Dioxide Materials Corporation) was activated by soaking in a 1.0 M KOH solution for 24 hours under 80 ℃ before use. The AEMWE test was operated at 80 °C with a peristaltic pump pumping 1.0 M KOH at a flow rate of 40 $ml \cdot min^{-1}$. Polarization curves were obtained from 1 to 1.8 V at a stepwise rate of 5 mV s^{-1}. A long-term stability test was conducted at a current density of 1 $A \cdot cm^{-2}$ at 80 °C."

Comment: 5) *For the experimental parts, the authors synthesized Rh-MoSSe catalysts with five different Se/S ratios. However, for the theoretical parts, only ratios of 2:0, 1:1, 0:2 were studied. As the authors highlight that the modulation is gradient-continuous, more Se/S ratios should be studied, which additionally help to identify the optimal catalyst composition.*

Response: We sincerely thank the reviewer for this insightful suggestion. In response, we have now

performed comprehensive density functional theory (DFT) calculations for the additional S/Se ratios of 1.5: 0.5 and 0.5: 1.5, thereby creating a complete and continuous theoretical dataset that fully aligns with our experimentally synthesized catalysts. The MoS_xSe_{2-x} and Rh_{SA}-MoS_xSe_{2-x} ($0 \leq x \leq 2$) structure models are shown in Figs. R18 and R19. The density of states (DOS) reveals that the incorporation of Rh single-atoms significantly increases the DOS of MoS_xSe_{2-x} near the Fermi level (Fig. R20). Moreover, the band gap of Rh_{SA}-MoSSe (0.85 eV) is notably narrower than those of Rh_{SA}-MoS_2, Rh_{SA}-$MoS_{1.5}Se_{0.5}$, $MoS_{0.5}Se_{1.5}$, Rh_{SA}-$MoSe_2$, and MoS_xSe_{2-x} substrates, thereby promoting accelerated electron transfer and optimizing the adsorption and desorption of reaction intermediates. Moreover, the S:Se ratio in Rh_{SA}-MoS_xSe_{2-x} series exhibits a volcano-type relationship with both the free energy of hydrogen adsorption (ΔG_{H*}) and hydroxide adsorption (ΔG_{OH*}), with Rh_{SA}-MoSSe located at the volcano apex, displaying optimal adsorption strengths for both species (H and OH). We also evaluated ΔG_{H*} and ΔG_{OH*} at all possible and thermodynamically stable adsorption sites on both pristine MoS_xSe_{2-x} and regions surrounding the Rh atoms in Rh_{SA}-MoS_xSe_{2-x} (Fig. R21 and 22). It is worth noting that the introduction of Rh single-atom substantially enhances the adsorption of H and OH at Rh sites, synergistically improving both the thermodynamics and kinetics of HER (Fig. R23). Gibbs free energy differences for the alkaline HER process in Rh_{SA}-MoS_xSe_{2-x} and MoS_xSe_{2-x} were calculated in Figs. R24-27. H_2O adsorption at Rh sites in Rh_{SA}-MoS_xSe_{2-x} is thermodynamically barrier-free and more favorable than at S/Se sites in Rh_{SA}-MoS_xSe_{2-x} or MoS_xSe_{2-x}. The rate-determining step (RDS) for alkaline HER in both systems is H_2O dissociation. The water dissociation free energy of Rh_{SA}-MoS_xSe_{2-x} exhibits a volcano-type relationship with the S: Se ratio, with the lowest energy barrier observed at an S: Se ratio of 1: 1 (volcano's apex). Moreover, the introduction of Rh single-atoms substantially lowers the water dissociation energy barrier at Rh sites and surrounding S/Se sites in Rh_{SA}-MoS_xSe_{2-x}, thereby accelerating the water dissolution kinetics (Fig. R28).

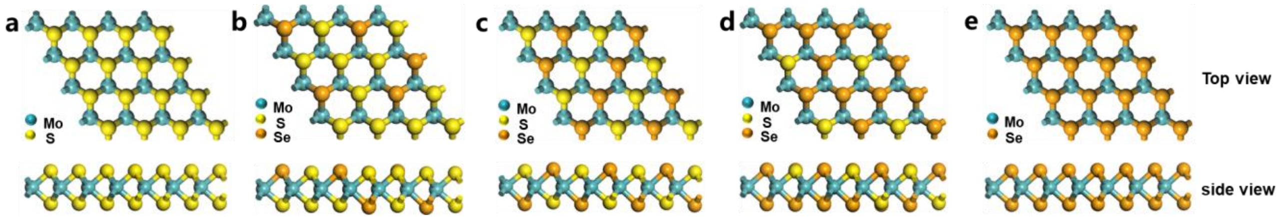

Fig. R18 (Supplementary Fig. 1). Structure models of MoS_xSe_{2-x} ($0 \leq x \leq 2$). Structure models of (a) MoS_2, (b) $MoS_{1.5}Se_{0.5}$, (c) MoSSe, (d) $MoS_{0.5}Se_{1.5}$, and (e) $MoSe_2$, depicted in top and side view. Blue,

yellow, and orange spheres represent molybdenum (Mo), sulfur (S), and selenium (Se) atoms, respectively.

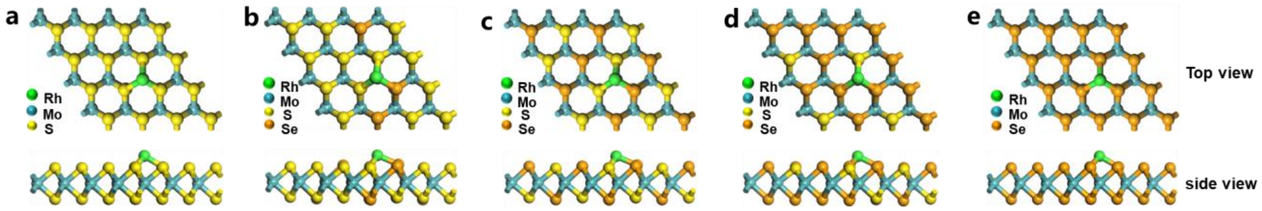

Fig. R19 (Supplementary Fig. 2). Structure models of Rh_{SA}-MoS_xSe_{2-x} ($0 \leq x \leq 2$). Structure models of (a) Rh_{SA}-MoS_2, (b) Rh_{SA}-$MoS_{1.5}Se_{0.5}$, (c) Rh_{SA}-MoSSe, (d) Rh_{SA}-$MoS_{1.5}Se_{0.5}$, and (e) Rh_{SA}-$MoSe_2$, presented in top and side view. Green, blue, yellow, and orange spheres represent rhodium (Rh), Mo, S, and Se atoms, respectively.

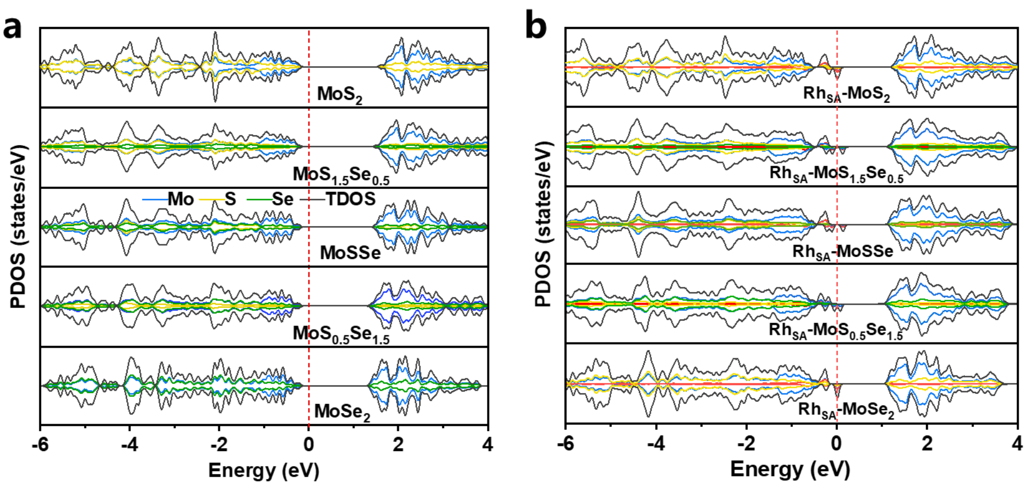

Fig. R20 (Supplementary Fig. 3). (a) Calculated density of states (DOS) for MoS_2, $MoS_{1.5}Se_{0.5}$, MoSSe, $MoS_{0.5}Se_{1.5}$, and $MoSe_2$. (b) Calculated DOS for Rh single-atom anchored systems: Rh_{SA}-MoS_2, Rh_{SA}-$MoS_{1.5}Se_{0.5}$, Rh_{SA}-MoSSe, Rh_{SA}-$MoS_{0.5}Se_{1.5}$, and Rh_{SA}-$MoSe_2$.

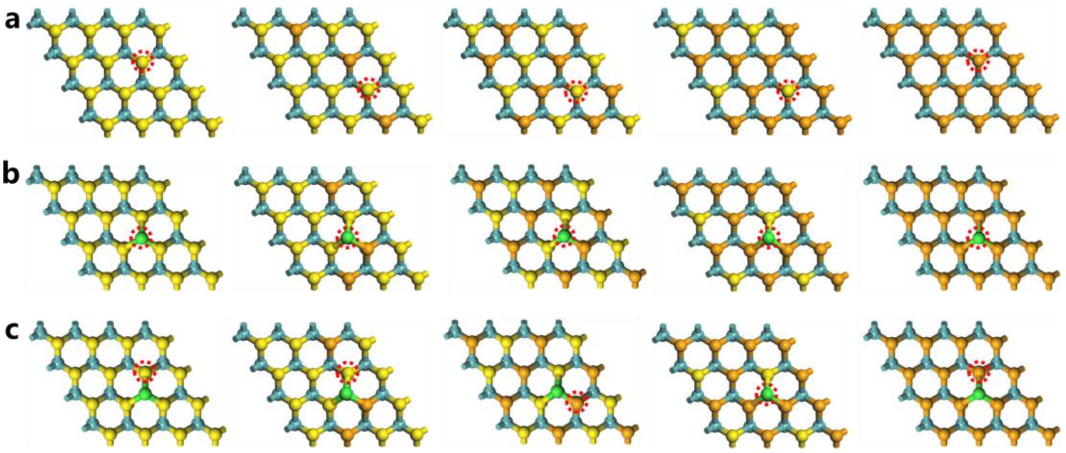

Fig. R21 (Supplementary Fig. 5). (a) S/Se sites for H adsorption in MoS_xSe_{2-x}. (b) Rh sites and (c) surrounding S/Se sites for H adsorption in Rh_{SA}-MoS_xSe_{2-x}.

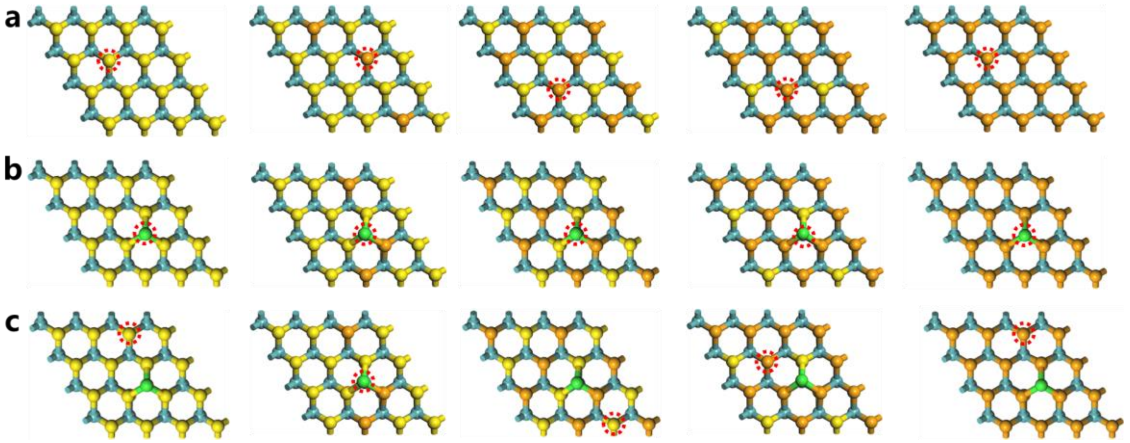

Fig. R22 (Supplementary Fig. 6). (a) S/Se sites for OH adsorption in MoS_xSe_{2-x}. (b) Rh sites and (c) surrounding S/Se sites for OH adsorption in Rh_{SA}-MoS_xSe_{2-x}.

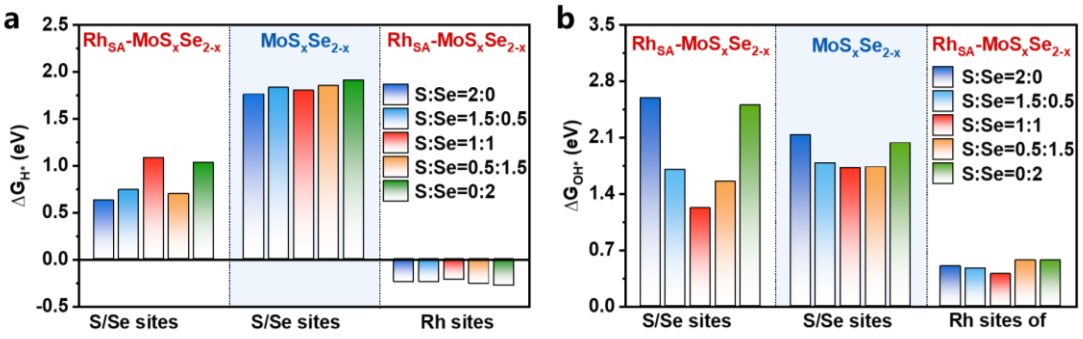

Fig. R23 (Supplementary Fig. 7). (a) H adsorption strength and (b) OH adsorption strength at S/Se sites in pristine MoS_xSe_{2-x}, Rh sites, and adjacent S/Se sites in Rh_{SA}-MoS_xSe_{2-x}.

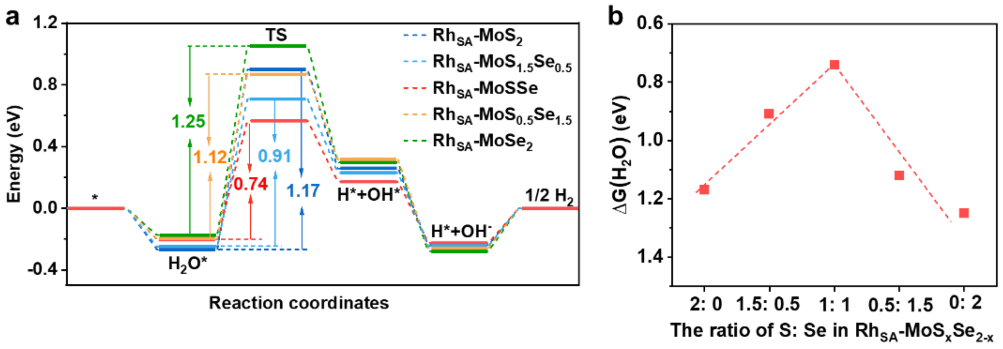

Fig. R24 (Fig. 1e). (a) Gibbs free energy diagram for the HER process on Rh_{SA}-MoS_xSe_{2-x} at Rh sites. (b) Correlation between the S:Se ratio in Rh_{SA}-MoS_xSe_{2-x} and water dissociation free energy at Rh sites.

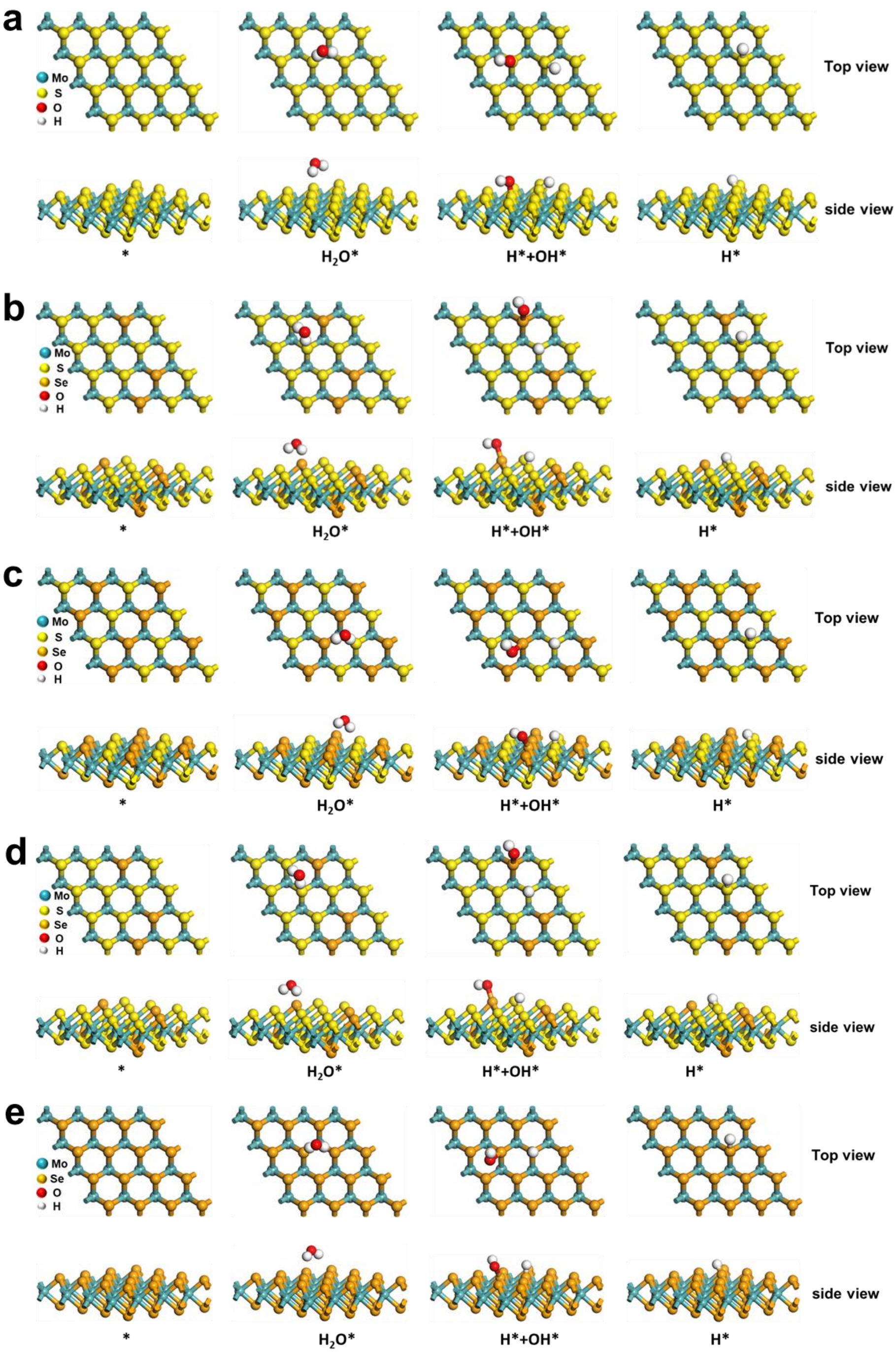

Fig. R25 (Supplementary Fig. 8-12). Diagram illustrating the HER at S/Se sites on (a) MoS_2, (b) $MoS_{1.5}Se_{0.5}$, (c) MoSSe, (d) $MoS_{0.5}Se_{1.5}$, and (e) $MoSe_2$.

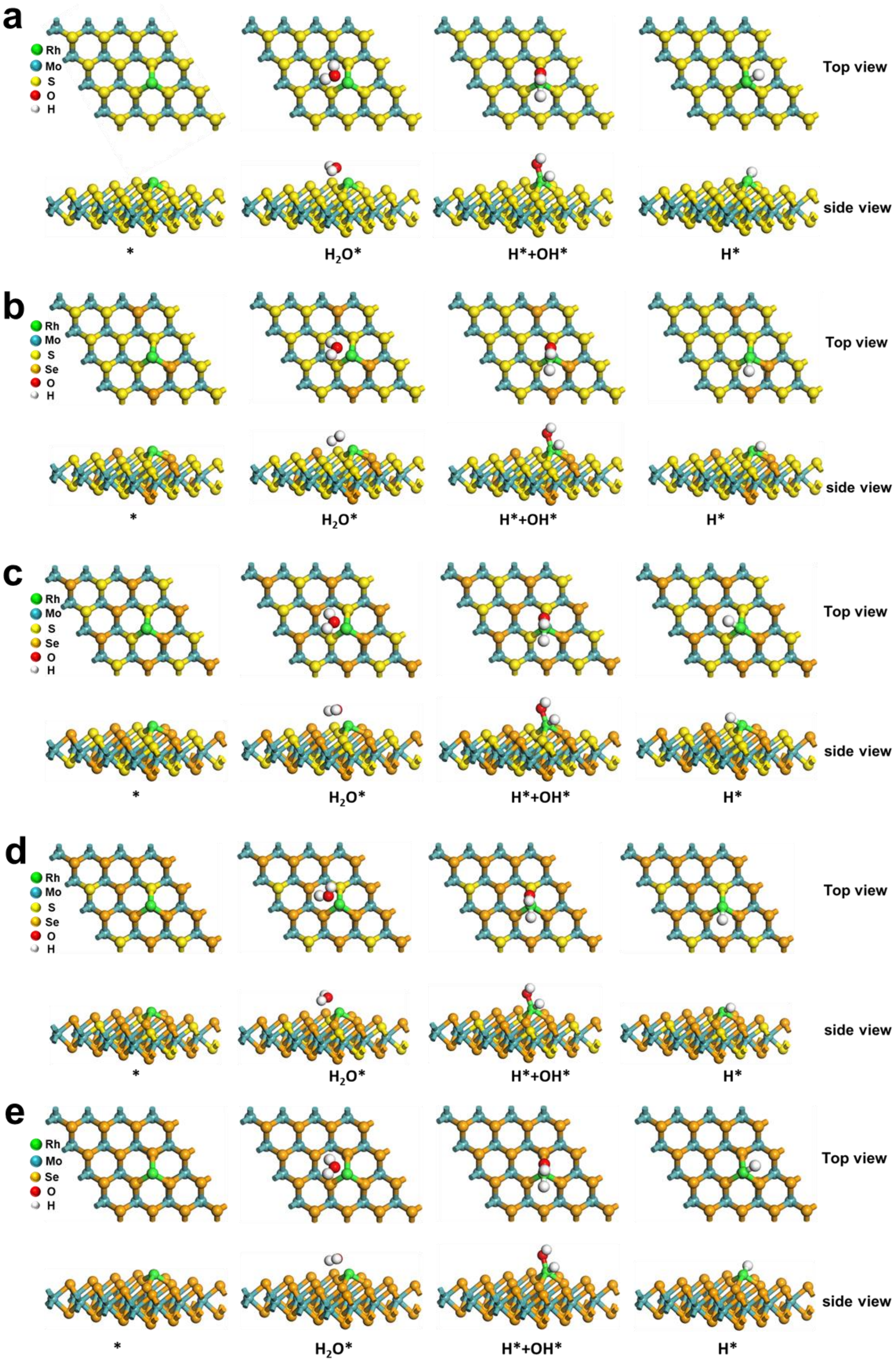

Fig. R26 (Supplementary Fig. 13-17). Diagram illustrating the HER at Rh sites on (a) Rh_{SA}-MoS_2, (b) Rh_{SA}-$MoS_{1.5}Se_{0.5}$, (c) Rh_{SA}-MoSSe, (d) Rh_{SA}-$MoS_{0.5}Se_{1.5}$, and (e) Rh_{SA}-$MoSe_2$.

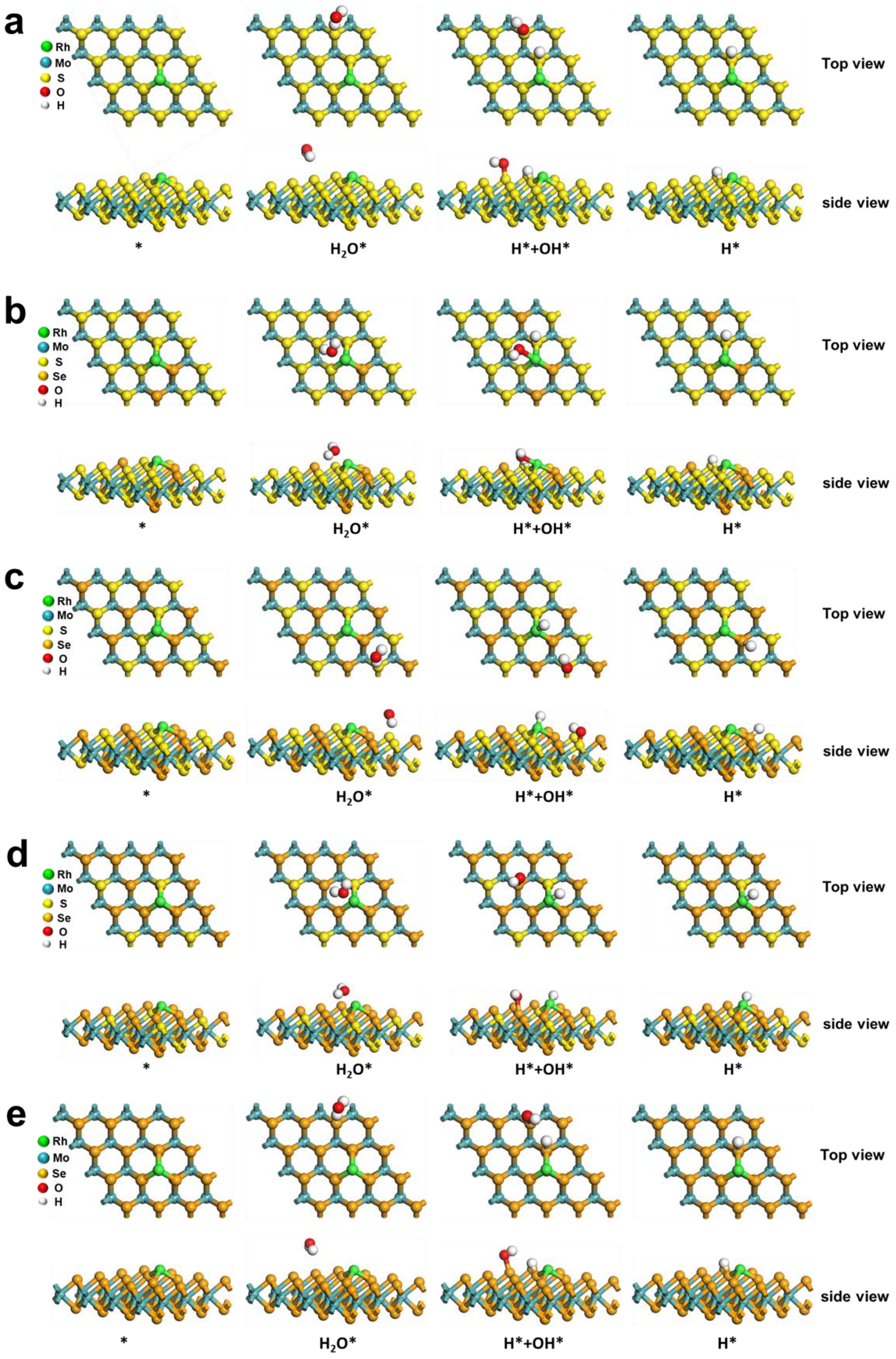

Fig. R27 (Supplementary Fig. 18-22). Diagram illustrating the HER at S/Se sites surrounding Rh single-atom on (a) Rh_{SA}-MoS_2, (b) Rh_{SA}-$MoS_{1.5}Se_{0.5}$, (c) Rh_{SA}-MoSSe, (d) Rh_{SA}-$MoS_{0.5}Se_{1.5}$, and (e) Rh_{SA}-$MoSe_2$.

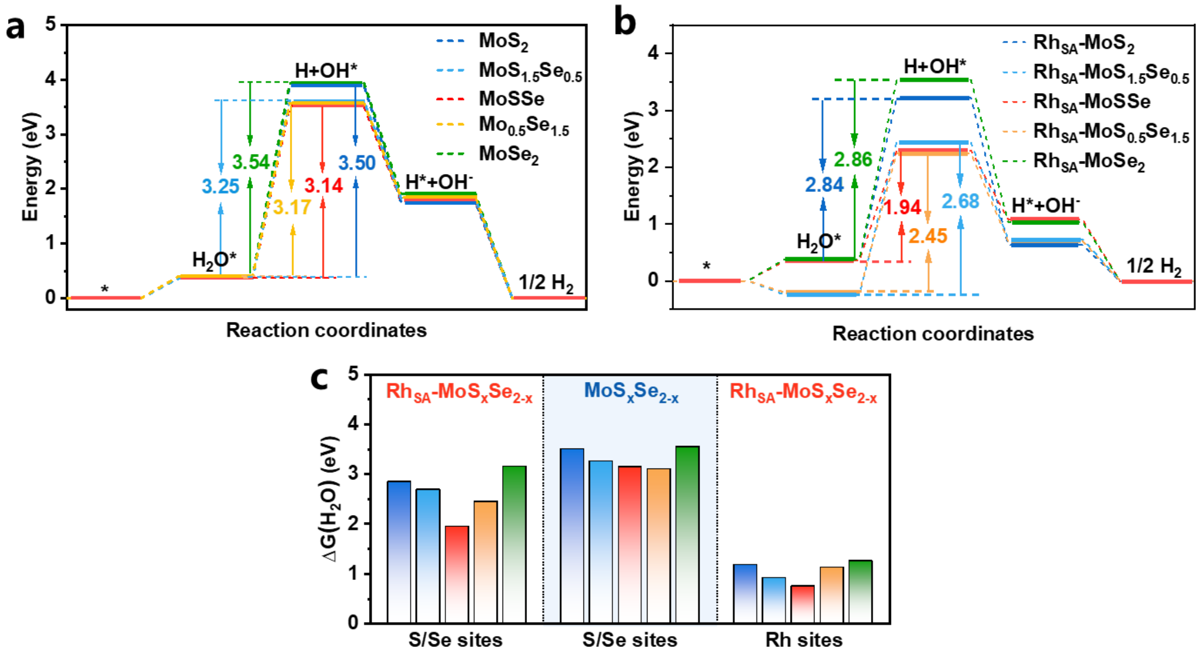

Fig. R28 (Supplementary Fig. 23). Gibbs free energy diagram for the HER process on (a) MoS_xSe_{2-x} at S/Se sites, (b) Rh_{SA}-MoS_xSe_{2-x} at S/Se sites surrounding Rh single-atom. (c) Water dissociation energies at S/Se sites in pristine MoS_xSe_{2-x}, Rh sites, and adjacent S/Se sites in Rh_{SA}-MoS_xSe_{2-x}.

We have also added these contents to the revised manuscript highlighted in yellow in Line 23, Page 4, and Line 1-7, Page 5, "First principal density functional theory (DFT) calculations were conducted to investigate the MSIs effects based on the MoS_xSe_{2-x} and Rh_{SA}-MoS_xSe_{2-x} ($0 \leq x \leq 2$) structure models (Supplementary Figs. 1 and 2). The density of states (DOS) reveals that the incorporation of Rh single-atoms significantly increases the DOS of MoS_xSe_{2-x} near the Fermi level (Supplementary Fig. 3). Moreover, the band gap of Rh_{SA}-MoSSe (0.68 eV) is notably narrower than those of Rh_{SA}-MoS_2 (0.88 eV), Rh_{SA}-$MoS_{1.5}Se_{0.5}$ (0.73 eV), Rh_{SA}-$MoS_{0.5}Se_{1.5}$ (0.70 eV), and Rh_{SA}-$MoSe_2$ (0.73 eV), and MoS_xSe_{2-x} substrates, thereby promoting accelerated electron transfer and optimizing the adsorption and desorption of reaction intermediates", and in Line 5-21, Page 7, "Moreover, the S: Se ratio in Rh_{SA}-MoS_xSe_{2-x} series exhibits a volcano-type relationship with both the free energy of hydrogen adsorption (ΔG_{H^*}) and hydroxide adsorption (ΔG_{OH^*}), with Rh_{SA}-MoSSe located at the volcano apex, displaying optimal adsorption strengths for both species (H and OH) (Fig. 1d and Supplementary Figs. 5 and 6). To further elucidate the impact of MSIs, we evaluated ΔG_{H^*} and ΔG_{OH^*} at all possible and thermodynamically stable adsorption sites on both pristine MoS_xSe_{2-x} and regions surrounding the Rh atoms in Rh_{SA}-MoS_xSe_{2-x}. It is worth noting that the introduction of Rh single-atom substantially enhances the adsorption of H and OH at Rh sites, synergistically improving

both the thermodynamics and kinetics of HER (Supplementary Fig. 7). Gibbs free energy differences for the alkaline HER process in Rh_{SA}-MoS_xSe_{2-x} and MoS_xSe_{2-x} were calculated in Supplementary Figs. 8-23. H_2O adsorption at Rh sites in Rh_{SA}-MoS_xSe_{2-x} is thermodynamically barrier-free and more favorable than at S/Se sites in Rh_{SA}-MoS_xSe_{2-x} or MoS_xSe_{2-x}. The rate-determining step (RDS) for alkaline HER in both systems is H_2O dissociation. Moreover, the water dissociation free energy of Rh_{SA}-MoS_xSe_{2-x} exhibits a volcano-type relationship with the S: Se ratio, with the lowest energy barrier observed at an S: Se ratio of 1: 1 (volcano's apex). Significantly, the incorporation of Rh single-atom in Rh_{SA}-MoS_xSe_{2-x} lowers the energy barriers for H_2O dissociation, effectively promoting the water dissociation."

***Comment:* 6)** *For Fig. S11, the peak shifts in the XRD patterns among different catalysts should be explained.*

Response: Thanks for the valuable comments. In the revised manuscript, we have added detailed explanation of the peak shift in the XRD patterns among different catalysts in the main text highlighted in yellow in Line 11-12, Page 9, and Line 1-4, Page 10. "As shown in Supplementary Figs. 30 and 31, the X-ray diffraction (XRD) patterns and corresponding Raman spectra indicate that all MoS_xSe_{2-x} samples maintain the same crystal structures to their parent structures of 2H phase MoS_2 (PDF#37-1492) and $MoSe_2$ (PDF#20-0757) without the impurity phase. Moreover, all main diffraction peaks of MoS_xSe_{2-x} are shifted toward a small angle with the Se concentration increasing, which is due to the gradual expansion of unit cells upon the substitution of S atoms by larger Se atoms[41,42]."

***Comment:* 7)** *For the Pt/C catalyst in Fig. S17, why two Tafel slopes in different potential regions were calculated?*

Response: Thank you very much for the valuable comments. The observed transition in the Tafel slope for the Pt/C catalyst is governed by the change of surface hydrogen coverage and mass transfer limitation as the overpotential increases, which is a common phenomenon with Pt/C catalysts (Fig. R29) (*Nat. Mater.* **22**, 1022-1029 (2023); *ACS Cent. Sci.* 10, 658-665 (2024); *Sci Rep* 5, 13801 (2015)).

[FIGURE REDACTED]

Fig. R29. (a) Tafel slopes of Pt_{tet}@$Ni(OH)_2$, Pt_{tet} and Pt/C pH 0 and 14, respectively. (b) Comparison of the Tafel slopes of Pt_{tet}@$Ni(OH)_2$ with the state-of-the-art alkaline HER catalysts. The dotted lines represent the Tafel slopes determined by three distinct rate-determining steps (rds): Volmer step (blue); Heyrovsky step (green) and Tafel step (red). All previous studies of Pt or modified Pt catalysts show a Tafel slope of ~40 mV per decade or above in alkaline electrolytes, consistent with a Volmer step or Heyrovsky limited mechanism, while the Tafel slope achieved with our Pt_{tet}@$Ni(OH)_2$ catalysts is below 27.3 ± 1.8 mV per decade, comparable to the typical values observed in the acidic electrolyte, suggesting abundant proton supply near the Pt surface in our unique design of Pt_{tet}@$Ni(OH)_2$ catalysts despite the bulk alkaline electrolyte. (c) Tafel plot of Pt at 5 $\mu g_{Pt}/cm^2$ loading. The Tafel slopes increase with an increased overpotential. (d) Simulated behavior of the Tafel relation for the hydrogen evolution reaction.

***Comment:** 8) What makes MoSSe (with or without Ru) have higher ECSA than both MoS_2 and $MoSe_2$?*

Response: Thank you for the valuable comments. Owing to the built-in electric field and in-plane dipole moment caused by the electron transfer from Se to S, the MoS_xSe_{2-x} with both S and Se anions exhibits superior conductivity and hydrophilic than MoS_2 and $MoSe_2$. Moreover, the introduction of

Rh increases the number of active sites and enhances charge transfer efficiency. Therefore, MoSSe (with or without Rh) have higher ECSA than both MoS_2 and $MoSe_2$.

In the revised manuscript, we have added explanation the descriptions for the higher ECSA of MoSSe (with or without Ru) than both MoS_2 and $MoSe_2$ in the main text highlighted in yellow in Line 4-8, Page 14. "Owing to the built-in electric field and in-plane dipole moment caused by the electron transfer from Se to S[39,40], the MoS_xSe_{2-x} with both S and Se anions exhibits superior conductivity and hydrophilic than MoS_2 and $MoSe_2$. Moreover, the introduction of Rh increases the number of active sites and enhances charge transfer efficiency."

Response to the reviewers' comments

We are grateful for the reviewers' in-depth comments and suggestions, which have helped us greatly to improve the quality of this manuscript. We have considered the comments and suggestions seriously, and carried out the related experiments following the suggestions of the reviewers. We have made detailed and **point-to-point response** and revised the manuscript accordingly with all changes being highlighted in yellow in the revised manuscript and Supporting Information.

Reviewer #1 (Remarks to the Author)

The authors adequately responded all reviewers' concerns, and thus it seems ready for publication now.

Response: Thanks for your positive comments and support for our work. We sincerely appreciate the time and effort you have dedicated to reviewing our manuscript. Your thoughtful suggestions are incredibly helpful and have served as an excellent guide for both my thesis and my overall research.

Reviewer #3 (Remarks to the Author)

The revised manuscript has been improved to the meet the high standard of Nature Communications, and this reviewer recommends its publication.

Response: We are truly grateful for your positive comments and support. Thank you once again for the time and care you've put into reviewing our work. Your suggestions have helped us to improve the quality of our manuscript and will guide us in our future work.

Reviewer #4 (Remarks to the Author):

The authors have provided thorough, systematic, and compelling responses to the reviewer's concerns. Their revisions, particularly the expansion of theoretical analysis and addition of key computational and experimental data, have significantly strengthened the manuscript. The work now presents a cohesive story that successfully bridges atomistic design principles with macroscopic catalytic performance. From a computational perspective, the theoretical foundation is sound and well-integrated. Therefore, I recommend acceptance of the manuscript but could be better if the following concerns are fixed.

Response: We are writing to express our sincere gratitude for your insightful feedback on our manuscript and your positive recommendation for its publication. In the revised manuscript and

supporting information, we have updated the important characterizations as suggested by the Reviewer. Please see below our point-by-point responses to the Reviewer's concerns.

Here are several points that should be enhanced:

Comment: 1) *The explanation of differing Rh-S and Rh-Se interactions based on polarizability and atomic size is chemically intuitive. To provide deeper, quantitative insight, the authors could consider including: a) Crystal Orbital Hamiltonian Population (COHP) analysis: To quantify the bonding/antibonding character and strength of Rh-S vs. Rh-Se bonds; b) Electron Localization function (ELF) or Bader charge analysis: To provide a more detailed picture of charge redistribution and bond covalency/ionicity.*

Response: Thank you very much for the valuable suggestion. We have added the COHP analysis and Bader charge analysis to provide deeper and quantitative insight of the Rh-S and Rh-Se interactions. The bonding characteristics and strength of the Rh-S and Rh-Se bonds in Rh_{SA}-MoS_xSe_{2-x} were quantitatively assessed using crystal orbital Hamilton population (COHP) analysis and the corresponding integrated COHP (ICOHP) values (Fig. R1). In all three Rh_{SA}-MoS_xSe_{2-x} systems, COHP curves show dominant bonding states below the Fermi level, verifying the stability of Rh-S and Rh-Se bonds (*Angew. Chem. Int. Ed.* **64**, e202419595 (2025); *Angew. Chem. Int. Ed.* **64**, e202505587 (2025)), with ICOHP values of -1.16 (Rh_{SA}-MoS_2), -1.21 (Rh_{SA}-MoSSe), and -1.17 (Rh_{SA}-$MoSe_2$). Notably, the slightly more negative ICOHP for Rh-MoSSe indicates a stronger covalent interaction, which originates from the asymmetric structure that polarizes the electron density and enhances orbital overlap. However, the three systems exhibit slightly different anti-bonding characteristics near the Fermi level. Rh_{SA}-MoS_2 displays more pronounced anti-bonding features suggestive of partial occupation of destabilizing states than Rh_{SA}-$MoSe_2$, while Rh_{SA}-MoSSe exhibits an intermediate level that reflects its asymmetric chalcogen environment. The nature of the interaction between the isolated Rh single atom and the MoS_xSe_{2-x} substrates was qualitatively and quantitatively analyzed through charge density difference (CDD) mappings and Bader charge analysis (Fig. R2) (*Angew. Chem. Int. Ed.* **64**, e202503608 (2025); *Angew. Chem. Int. Ed.*, **64**, e202510383 (2025)). CDD analyses reveal distinct charge redistribution between Rh single-atoms and coordinated S/Se atoms, resulting in a marked reduction of electron density around Rh atoms. For Rh-MoS_2, significant charge accumulation appears between Rh and S atoms, indicating polar covalent bonding with ionic character. For Rh-

$MoSe_2$, minimal charge redistribution is observed, suggesting weak covalent interaction with negligible ionicity. Bader charge analysis quantifies such electron transfer, affording positive values of 0.22 |e| (Rh-MoS_2), 0.14 |e| (Rh-MoSSe), and 0.01 |e| (Rh-$MoSe_2$), which unambiguously confirm the electron donation from Rh to the substrates. The substantial transfer in Rh-MoS_2 (0.22 |e|) signifies strong ionic character, while the negligible transfer in Rh-$MoSe_2$ (0.01 |e|) confirms a nearly non-ionic interaction. Thus, the decreasing Bader charges quantitatively capture the transition from polar ionic-covalent bonding in Rh-MoS_2 to metallic/weak covalent bonding in Rh-$MoSe_2$, fully consistent with the CDD observations.

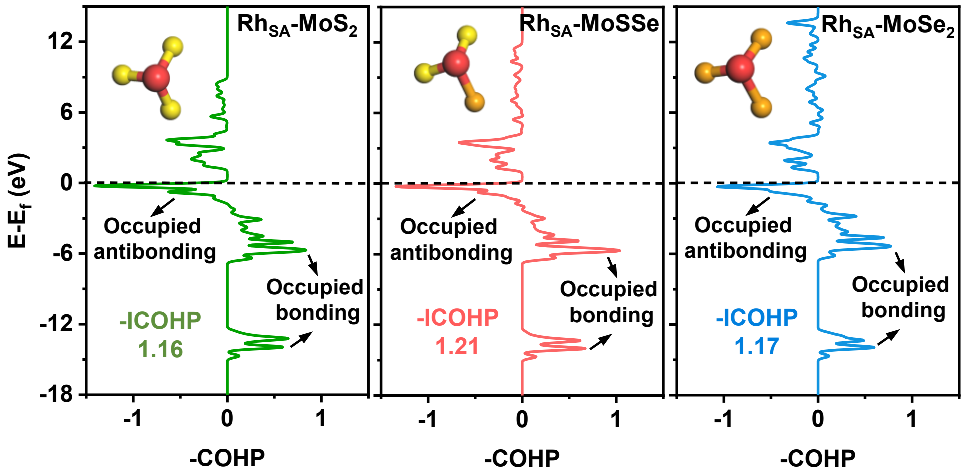

Fig. R1 (Fig. 3b). The COHP analysis of Rh-S, Rh-S/Se, and Rh-Se bonds in Rh$_{SA}$-MoS_2 (left), Rh$_{SA}$-MoSSe (middle), and Rh$_{SA}$-$MoSe_2$ (right).

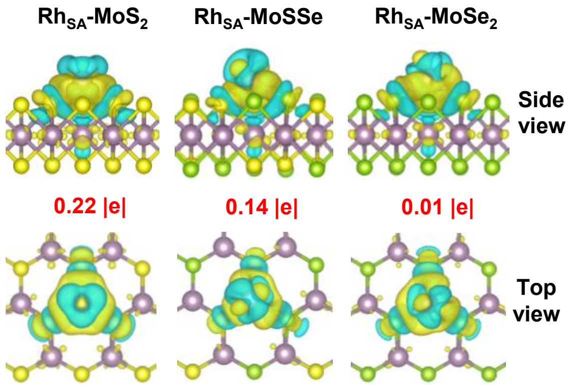

Fig. R2 (Supplementary Fig. 35a). The charge density difference of Rh atoms in Rh$_{SA}$-MoS_2, Rh$_{SA}$-MoSSe, and Rh$_{SA}$-$MoSe_2$, with yellow and blue regions indicating charge accumulation and decrease, respectively.

In the revised manuscript, we have also added the COHP and Bader charge analysis highlighted in

yellow in Line 14-23, Page 11 and Line 1-10, Page 12, "The bonding characteristics and strength of the Rh-S and Rh-Se bonds in Rh_{SA}-MoS_xSe_{2-x} were investigated using crystal orbital Hamilton population (COHP) and integrated COHP (ICOHP) analysis (Fig. 3b). The COHP curves of All three systems show dominant bonding states below the Fermi level, confirming stable Rh-S and Rh-Se bonds, with ICOHP values of -1.16 (Rh_{SA}-MoS_2), -1.21 (Rh_{SA}-MoSSe), and -1.17 (Rh_{SA}-$MoSe_2$). Notably, the more negative ICOHP value of Rh-MoSSe indicates a stronger covalent interaction due to the asymmetric structure that polarizes the electron density and enhances orbital overlap. However, the three systems exhibit slightly different anti-bonding characteristics near the Fermi level. Rh_{SA}-MoS_2 displays more pronounced anti-bonding features suggestive of partial occupation of destabilizing states than Rh_{SA}-$MoSe_2$, while Rh_{SA}-MoSSe exhibits an intermediate level that reflects its asymmetric chalcogen environment. Charge density difference (CDD) analyses reveal charge redistribution between Rh single-atoms and coordinated S/Se atoms, resulting in a marked reduction of electron density around Rh atoms (Supplementary Fig. 35a). Bader charge analysis quantifies such electron transfer, affording positive values of 0.22 |e| (Rh-MoS_2), 0.14 |e| (Rh-MoSSe), and 0.01 |e| (Rh-$MoSe_2$), which further confirm the electron donation from Rh to the substrates. The substantial transfer in Rh-MoS_2 (0.22 |e|) signifies strong ionic character, while the negligible transfer in Rh-$MoSe_2$ (0.01 |e|) confirms a nearly non-ionic interaction. Therefore, the decreasing Bader charges quantitatively capture the transition from polar ionic-covalent bonding in Rh-MoS_2 to weak covalent bonding in Rh-$MoSe_2$, fully consistent with the CDD observations.".

Comment: 2) *Replacing "number of active sites" with "ECSA" is a good correction. However, the discussion should carefully distinguish between electrochemically accessible surface area (related to double-layer capacitance) and the intrinsic activity per catalytic site. The authors' normalization of current to ECSA is the correct approach to isolate intrinsic activity.*

Response: We are particularly grateful for your thoughtful comment on distinguishing between electrochemically accessible surface area and intrinsic catalytic activity. The catalysts mass activity is fundamentally determined by the product of the electrochemically active surface area (ECSA, normalized by mass) and the specific activity (SA, catalytic current normalized by ECSA) (*Nat. Catal.* **2**, 495-503 (2019); *Nat. Mater.* **20**, 208-213 (2021); *Science* **354**, 1414-1419 (2016)). In contrast to the geometrical surface area, the ECSA represents the actual surface area accessible to the electrolyte

(*Chem. Soc. Rev.*, 48, 2518-2534 (2019); *Energy Environ. Sci.*, **11**, 744-771 (2018)). Normalizing activity by ECSA thus offers a more accurate and meaningful metric for evaluating the catalytic properties of diverse electrocatalysts, accounting for variations in size, shape, morphology, topography, and porosity. Therefore, we also normalized the polarization curves of Rh_{SA}-MoS_xSe_{2-x} and MoS_xSe_{2-x} samples by ECSA to confirm the superior intrinsic activities of Rh_{SA}-MoSSe.

In the revised manuscript, we have also added the discussion between the electrochemically accessible surface area and the intrinsic activity per catalytic site highlighted in yellow in Line 10-13, Page 15, "The catalysts mass activity is fundamentally determined by the product of the electrochemically active surface area (ECSA, normalized by mass) and the specific activity (SA, catalytic current normalized by ECSA). In contrast to the geometrical surface area, the ECSA represents the actual surface area accessible to the electrolyte.", and Line 21-23, Page 15, "Normalizing activity by ECSA thus offers a more accurate and meaningful metric for evaluating the catalytic properties of diverse electrocatalysts, accounting for variations in size, shape, morphology, topography, and porosity.".

Comment: 3) *While the volcano plot is presented, establishing a more quantitative link between the computed adsorption energies/water dissociation barriers and the experimental overpotential/Tafel slope via microkinetic modeling or applying the Brønsted-Evans-Polanyi (BEP) relation would represent a pinnacle of computational electrocatalysis. This could be suggested as a direction for future work if beyond the current scope.*

Response: Thanks for the insightful and valuable suggestion. Given that computational results indicate water dissociation is the rate-determining step, we added the relationship between the computed water dissociation barriers ($\Delta G(H_2O)$) and experimental overpotential required to achieve a current density of 10 mA cm^{-2} (η_{10}) based on the Brønsted-Evans-Polanyi (BEP) relationship (*Nat Energy* **5**, 891-899 (2020)). As shown in Fig. R3, a lower $\Delta G(H_2O)$ corresponds to a smaller overpotential and thus significantly enhanced alkaline HER activity, revealing the critical role of water dissociation kinetics in determining the overall alkaline HER performance. In the present work, constrained by the modulation range of the support effect derived from the electronic metal-support interaction (EMSI) in TMD-based materials, the water dissociation energy barrier of Rh_{SA}-MoS_xSe_{2-x} is limited to a record-low value of 0.74 eV. Accordingly, the rational design of high-performance alkaline HER

electrocatalysts should focus on constructing catalytic sites with intrinsically low water dissociation energy and precisely tuning the electronic structure to further reduce $\Delta G(H_2O)$, thereby accelerating water dissociation kinetics and achieving superior alkaline HER activity.

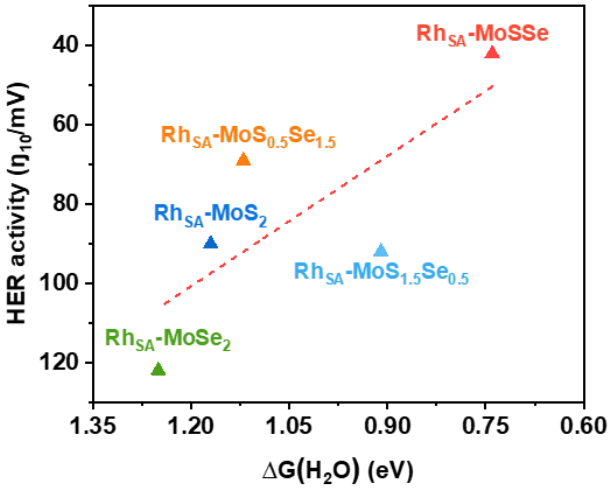

Fig. R3 (Supplementary Fig. 40c). (a) Relationship of $\Delta G(H_2O)$ and alkaline HER activity of Rh_{SA}-MoS_xSe_{2-x}.

In the revised manuscript, we have also added the discussion highlighted in yellow in Line 22-23, Page 14 and Line 1-9, Page 15, "Moreover, we have added the relationship between the computed $\Delta G(H_2O)$ and experimental overpotential required to achieve a current density of 10 mA cm^{-2} (η_{10}) based on the Brønsted-Evans-Polanyi (BEP) relationship. As shown in Supplementary Fig. 40c, a lower $\Delta G(H_2O)$ corresponds to a smaller overpotential and thus significantly enhanced alkaline HER activity, revealing the critical role of water dissociation kinetics in determining the overall alkaline HER performance. In this work, the water dissociation barrier of Rh_{SA}-MoS_xSe_{2-x} is limited to 0.74 eV due to the modulation scope of EMSI in TMD-based supports. Therefore, future high-performance alkaline HER electrocatalysts should be rationally designed by constructing active sites with intrinsically low water dissociation energy and optimizing electronic structures to further reduce $\Delta G(H_2O)$, thus promoting water dissociation kinetics and enhancing alkaline HER activity.".
